# Effect of tetracycline on nitrogen removal in Moving Bed Biofilm Reactor (MBBR) System

**DOI:** 10.1371/journal.pone.0261306

**Published:** 2022-01-10

**Authors:** Yan Shu, Donghui Liang

**Affiliations:** 1 School of Environment and Energy, South China University of Technology, Guangzhou Higher Education Mega Centre, Guangzhou, China; 2 The Key Lab of Pollution Control and Ecosystem Restoration in Industry Clusters, Ministry of Education, South China University of Technology, Guangzhou Higher Education Mega Centre, Guangzhou, China; Guangzhou University, CHINA

## Abstract

The effect of tetracycline (TC) on nitrogen removal in wastewater treatment plants has become a new problem. This study investigated the effects of TC on nitrogen removal using a Moving Bed Biofilm Reactor system. The results showed that there was no significant effect on nitrogen removal performance when the concentration of TC was 5 mg/L, and that the total nitrogen (TN) removal efficiency could reach 75–77%. However, when the concentration of TC increased to 10 mg/L, the denitrification performance was affected and the TN removal efficiency decreased to 58%. The abundance of denitrifying bacteria such as those in the genus Thauera decreased, and TC-resistant bacteria gradually became dominant. At a TC concentration of 10 mg/L, there were also increases and decreases, respectively, in the abundance of resistance and denitrification functional genes. The inhibitory effect of TC on denitrification was achieved mainly by the inhibition of nitrite-reducing bacteria.

## Introduction

The presence of residual antibiotics in water, of which tetracycline (TC) is a typical representative, are a new environmental problem. TC is widely used as a broad-spectrum antibiotic in the treatment of human diseases, animal husbandry, and aquaculture. TC production and usage in China is second and first, respectively, in the world [1,2]. TC is difficult to degrade and can inhibit the synthesis of bacterial proteins from the 30S units from bacterial ribosomes by connecting to a-tRNA binding site A, which inhibits the growth and reproduction of bacteria [3]. Traditional wastewater treatment processes cannot degrade TC effectively, and its presence has become an important issue for the stable operation and maintenance of sewage treatment plants [4]. In one study, the effluent concentration of TC in a sewage treatment plant was detected in the range of μg/L to mg/L [5]. In pharmaceutical wastewater, the concentration of TC can reach 32 mg/L [6]. In addition, the continuous accumulation of residual TC might produce TC-resistant genes in bacterial bacteria [7]. These resistant bacteria spread to humans through the food chain [8]. Over time, the high selective pressure from TC may integrate genetic resistance into the bacterial genome [9]. Therefore, an increasing number of researchers are paying attention to environmental problems caused by TC [10].

In traditional wastewater treatment plants, ammonia nitrogen (ammonia-N) in sewage is removed mainly by microorganisms, and TC may affect their community structure and abundance during the nitrogen removal process [11]. A previous study [12] has shown that when the concentration of TC was 20 μg/L, there was no obvious effect on the diversity and abundance of microbial denitrifiers in the short term; however, long-term exposure changed the structure of the microbial community, with a decrease in the abundance of *Lysobacter*, *Dechloromonas*, and *Flavobacterium*. Under TC-related stress, microorganisms that cannot adapt to the presence of TC are replaced by microorganisms with stronger adaptability. Functional microorganisms such as nitrobacteria and denitrifiers are also affected by TC; denitrifying bacteria, for example, are more sensitive to TC than other heterotrophic bacteria [13]. It has been reported that the abundance of denitrifying bacteria increased when the TC concentration was 5 mg/L, and that the denitrification effect was enhanced [14]. A low TC concentration can promote microorganisms to form larger flocs, which stabilizes the living environment of the microbial community inside the floc and causes cellular responses similar to the function of “signal molecules.” However, high TC concentrations can cause denitrifying bacteria to separate from sludge substrates [14]. The cellular responses caused by TC differ for different types of functional bacteria [15,16]. Researchers found that under TC stress, the relative abundance of *Nitrospira* and *Nitrobacter* increased via the auto-induction of TC, which promoted the expression of the group effect [17]. However, there is currently still a lack of systematic research on the effect of TC on nitrogen removal. The mechanism of nitrogen removal under TC stress remains unclear, although there are some reports on the changes in microbial communities or resistance genes under antibiotic stress [18–22], The expression of key denitrification genes and the dynamic structural changes to bacteria need to be investigated further.

In this study the nitrogen removal performance, evolution of the microbial community structure, effect of functional denitrification genes, and resistance genes were systematically explored via an Moving Bed Biofilm Reactor (MBBR) system using high-throughput sequencing and fluorescent quantitative Polymerase Chain Reaction (PCR) technology. The inhibitory effect of TC on denitrification and the spread and accumulation of resistance genes was also investigated. Furthermore, the balance of nitrogen in the MBBR system and the nitrogen removal kinetics of the latter were discussed, and the mechanism of nitrogen removal in the MBBR system under TC stress was clarified. This study provides a theoretical basis for the removal of nitrogen from sewage containing TC.

## Materials and methods

### Synthetic wastewater and inoculated sludge

The inoculated sludge was collected from the active sludge in the outer ring of an integrated concentric circular aerobic reactor in a sewage treatment station in Guangzhou. The sludge parameters were: Mixed Liquor Suspended Solids (MLSS) = 4900–6400 mg/L, Mixed Liquid Volatile Suspended Solids (MLVSS) = 2300–2800 mg/L, MLVSS/MLSS = 0.44–0.49.

The synthetic wastewater used in the experiment were made up by medicines including CH_3_COONa (0.5 g/L), NH_4_Cl (0.1 g/L), KH_2_PO_4_ (0.1 g/L), MgSO_4_·7H_2_O (0.05 g/L), CaCl_2_ (0.05 g/L), NaCl (0.5 g/L), TC (5 mg/L), and microelement (2 mL/L), and the pH was 7.0.

### Constructing and operating the MBBR

The MBBR system constructed for this study consisted of the following parts: a suction sump, water suction pump, biological carrier, gas pump, and reaction tank with an effective cubage of 5 L. Biological carrier chosen to form the biofilm was YuLong biological filler, and the important parameters included as follows: ɸ 25×10 mm of size; 960 m^2^/m^3^ of working surface; 0.94 g/cm^3^ of density and void ratio > 80%. The filling ratio was 40%. The water suction pump pumped synthetic wastewater into the reactor. Biofilm formation was carried out as follows: 1 L of activated sludge was poured into the reactor with a sodium acetate carbon source and ammonia-Nammonia-N nitrogen source. The distinction in the parameter at three periods was the initial concentration of TC, and the time period was set to 20 d (periods I, II, and III). The concentrations of normal pollutants, including nitrate nitrogen (nitrate-N), ammonia-N, nitrite nitrogen (nitrite-N), and TN were recorded every day.

### The evolution of microbial community

In order to study the effects of TC on the succession of microbial communities in MBBR, high-throughput sequencing analysis was adopted. The samples of three period were collected respectively (Sample ID: B1, B2, B3). After extracting the DNA with the kit, the DNA was amplified by PCR (primer sequence: F: ACTCCTACGGGAGGCAGCA, R: GGACTACHVGGGTWTCTAAT) and then sent to BioMaker Company (Beijing) for sequencing.

### Determination of functional genes and TC resistance genes

The samples were collected from the filler in three period of MBBR. Four TC concentration resistance genes (tetX, tetW, tetM, and tetA), and two functional genes (nirS, nirK) were detected by the real-time PCR detection system. DNA was extracted using kit and amplified by PCR. The amplified samples were sent to Beijing BioMaker Company for determination. The primers used were showed in **Table 1**.

**Table 1:** PCR primers of the tetracycline resistance genes and denitrification functional genes.

Target gene	Primer	Primer sequences
***tet*X**	*tet*X -F	AGCCTTACCAATGGGTGTAAA
	*tet*X -R	TTCTTACCTTGGACATCCCG
***tet*W**	*tet*W -F	GAGAGCCTGCTATATGCCAGC
	*tet*W -R	GGGCGTATCCACAATGTTAAC
***tet*M**	*tet*M -F	ACAGAAAGCTTATTATATAAC
	*tet*M -R	TGGCGTGTCTATGATGTTCAC
***tet*A**	*tet*A -F	GCTACATCCTGCTTGCCTTC
	*tet*A -R	CATAGATCGCCGTGAAGAGG
***nir*S**	*nir*S-F	GASTTCGGRTGSGTCTTGA
	*nir*S-R	GTSAACGTSAAGGARACSGG
***nir*K**	*nir*K-F	GGAATGGTGCCCTGGCA
	*nir*K-R	GCCTCGATCAGATTATGG

### Analytical methods

TN, Ammonia-N, nitrate-N and nitrite-N of the samples were detected by the ultraviolet spectrophotometer (DR5000, America). The detection method of sewage indicators adopted the standard methods. pH was detected by pH detector (PHB-4, China). The total organic carbon (TOC) was detected by TOC analyzer. DO was detected by dissolved oxygen meter (HQ30d, America).

### Data analysis

All the experiments were repeated for three times and the average value was taken as the result. The experimental data and the error analysis were calculated using the software Microsoft Excel and Origin 2018, and then drew diagrams.

#### Analysis of nitrogen balance

After the stable operation of stages I and III, samples were centrifuged and the concentrations of ammonia-N, nitrate-N, nitrite-N and total nitrogen in the supernatant were measured. The same samples were crushed using a sonic cell pulverizer and then filtered through a filter membrane to measure the concentrations of nitrogen and TN in the supernatant, The nitrogen balance was calculated–based on the changes in the concentrations of nitrogen by using the following equation [23].


Organic‐N=TN‐nitrate‐N‐nitrite‐N‐ammonia‐N (1)

Intracellular‐N=TN‐totalsoluble‐N (2)

Gaseous‐N=TN‐nitrate‐N‐nitrite‐N‐ammonia‐N‐organic‐N‐intracellular‐N (3)

#### Calculation method of carbon balance and electron flow distribution

The MBBR system was operated with sodium acetate as the sole carbon source; TC was not added. The effluent supernatant of MBBR was collected and the ammonia-N, nitrate-N, nitrite-N, TN, and Total Organic Carbon (TOC) concentrations were tested after centrifugation. The electron flow distribution was calculated–based on the changes in the concentrations of ammonia-N, nitrate-N, nitrite-N, TN, and TOC of the MBBR effluent–using the following equation [23]:

$$Aerobic\;respiration:\;C_{2}H_{3}O_{2}^{-}+2O_{2}\to CO_{2}+HCO_{3}^{-}+H_{2}O$$ (4)

$$Denitrification:\;C_{2}H_{3}O_{2}^{-}+8NO_{3}^{-}\to 3CO_{3}^{2-}+7HCO_{3}^{-}+4N_{2}+4H_{2}O$$ (5)

## Results and discussion

### Nitrogen removal performance

**Fig 1:**
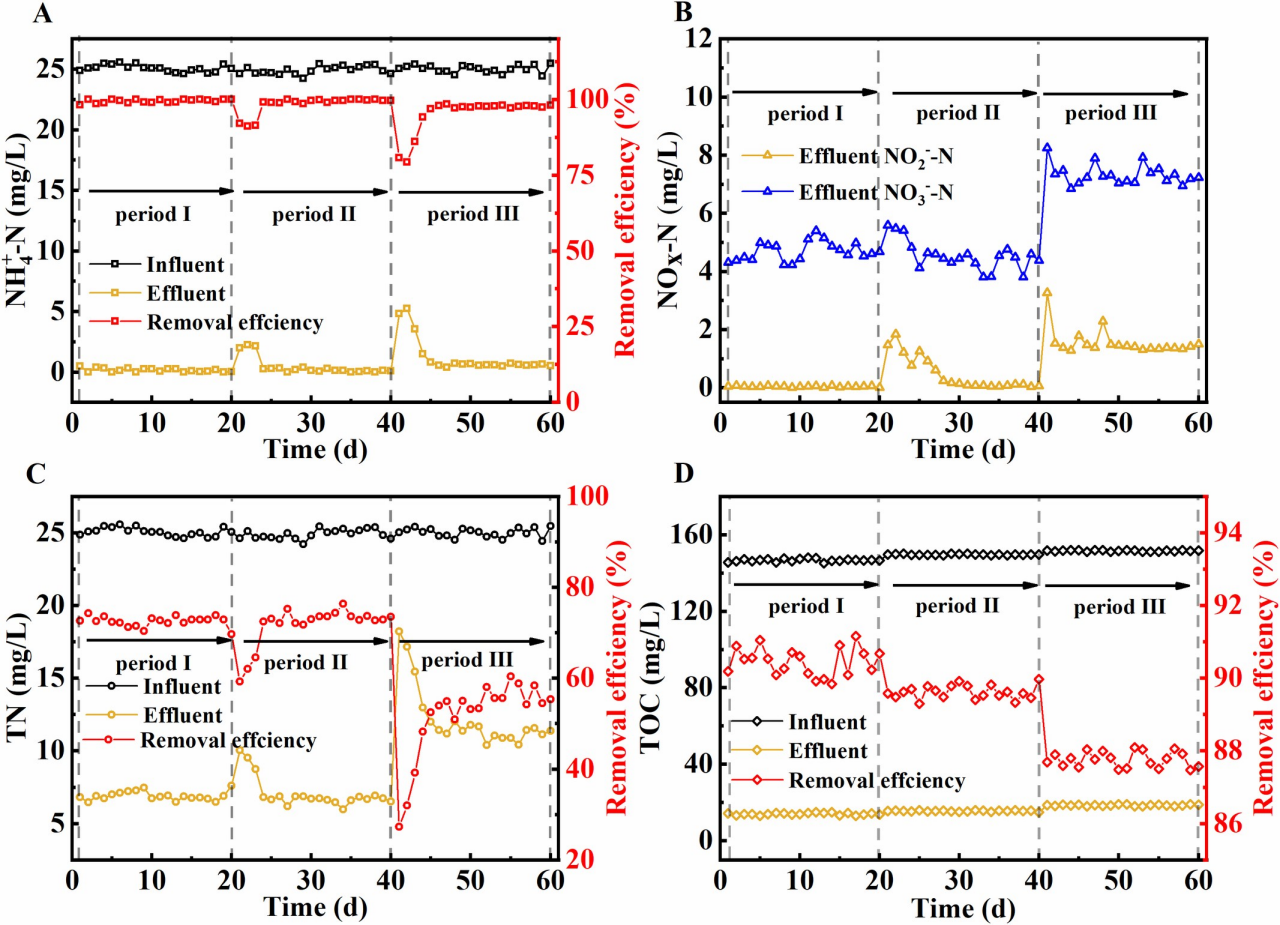
Concentration and removal efficiency of NH_4_^+^-N (A), TN (C) and TOC (D) in influent and effluent and NOx-N in effluent of MBBR(B).

The biofilms in the filler grew and matured gradually over three weeks. Then the investigation of the influence of TC on nitrogen removal performance under different TC concentrations began and lasted for 60 d, with periods I, II, and III consisting of 20 d each. As shown in **Fig 1**, when the TC concentration rose gradually from 0 (period I) to 10 mg/L (period III), the concentration of ammonia-N in the effluent was less than 0.5 mg/L on average, and the removal rate of the latter was stable above 98%. This result showed that there the ammonia-N removal efficiency is high in an MBBR system when the TC concentration remained in the range of 0–10 mg/L. The nitrification of the MBBR system was not affected by TC concentrations below 10 mg/L, this could be caused by the high tolerance of nitrifying bacteria to TC below 10mg/L [11]. The average concentration of nitrate and nitrite in effluent changed from 4.73 mg/L and 0.04 mg/L to 4.62 mg/L and 0.07 mg/L, respectively, when the TC concentration increased from 0 to 5 mg/L. However, the average concentration of nitrate and nitrite in effluent increased to 7.42 mg/L and 1.43 mg/L, respectively, when the TC concentration rose to 10 mg/L (period III). The TN removal efficiency decreased gradually from 77.12% to 58.41% when the TC concentration increased from 5 mg/L (period II) to 10 mg/L (period III). These results indicated that the MBBR system had a high TN removal efficiency when the TC concentration was maintained at 0–5 mg/L, at which level the bacteria had a high TC tolerance. However, the denitrification of the system was inhibited when the TC concentration reached 10 mg/L; the TOC removal rate stabilized at approximately 90%. The results showed that the TOC removal efficiency was high in the MBBR system when the TC concentration was maintained at 0–10 mg/L. The TOC removal rate was not affected by TC concentrations below 10 mg/L.

### The evolution of microbial community

#### The evolution of microbial community diversity

Generally, ACE and Chao indexes were used to measure the relative abundance of species, and the Shannon and Simpson indexes were used to measure the diversity of species. The Alpha diversity in each period of MBBR system was shown in **Table 2**. The OTU, ACE and Shannon of the microbial community changed from 556, 660.881 and 5.2119 to 573, 637.374 and 5.6943, respectively, when TC concentration rose from 0 to 5 mg/L. It showed that there was a high diversity of microbial community in MBBR system when TC concentration stayed around a range (0–5 mg/L). Moreover, the OUT ACE, and Shannon of the microbial community increased from 573, 637.374 and 5.6943 to 755, 813.747 and 6.707, respectively, when TC concentration increased from 5 mg/L to 10 mg/L. These consequences indicated that diversity of microbial community in MBBR system was not influenced by TC concentration below 10 mg/L.

**Table 2:** Alpha diversity index of MBBR system.

Period	OTU	ACE	Chao1	Simpson	Shannon	Coverage
TC = 0 mg/L	566	660.881	664.1	0.9274	5.2119	0.9983
TC = 5 mg/L	573	637.374	636.984	0.9508	5.6943	0.9983
TC = 10mg/L	755	813.747	829.75	0.9529	6.707	0.9985

#### The evolution of microbial community structures

**Fig 2:**
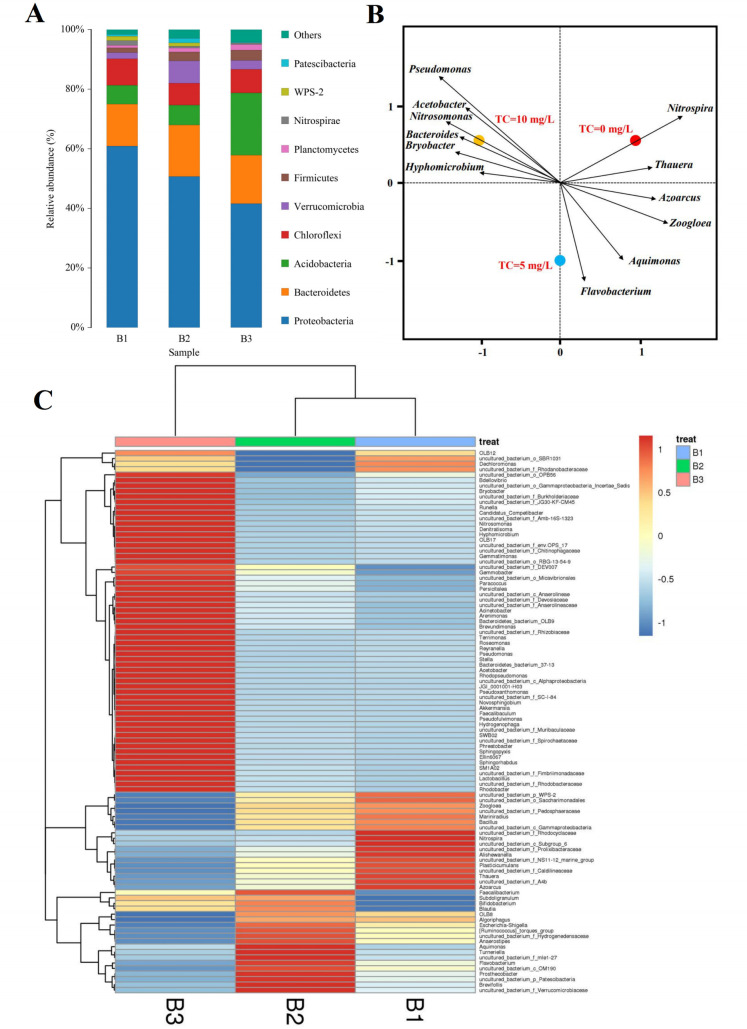
Classification of microorganisms in the MBBR: bacterial community at phylum-level (A); heatmap of genus level (B); RDA ordination analysis of the correlation between frequent species dynamics and the samples (C).

The initial concentration of TC had effect on microbial community structure in MBBR system. It was found that microbial community structure in MBBR system influenced removal efficiency of the nitrogen. Therefore, it was necessary to analysis the evolution of microbial community structure in MBBR system, as shown in **Fig 2**.

**Fig 2A** showed the microbial community structures at phylum level. The major phyla were *Proteobacteria*, *Bacteroidetes*, *Acidobacteria*, *Chloroflexi*, *Verrucomicrobia*, *Firmicutes*, and *Nitrospirae*. When the TC concentration increased from 0 to 10 mg/L, the relative abundances of *Proteobacteria* and *Chloroflexi* decreased from 61% to 42% and 9% to 6%, respectively. The relative abundance of *Bacteroidetes* remained consistent at 14%–16%. The relative abundance of *Acidobacteria* was the highest when the TC concentration was 10 mg/L. *Proteobacteria* and *Bacteroidetes* are closely related to nitrogen removal [24]; the former has a significant effect on the denitrification process [25]. These results indicated that *Proteobacteria* have a poor tolerance to TC. *Bacteroidetes* is a common major phylum in sewage treatment plants and its relative abundance did not change significantly under TC stress conditions, indicating that *Bacteroidetes* was not affected by TC. *Acidobacteria* influenced the degradation of organics [26], and its relative abundance increased when the TC concentration rose to 10 mg/L. This indicated that *Acidobacteria* may have a certain degrading effect on TC.

**Fig 2B** showed the structures of the microbial community in genus level. The relative abundance of Azoarcus, Thauera, Nitrospira, and Zoogloea were higher than those of other genera of bacteria. Azoarcus influenced the denitrification process [27]. *Thauera* has been reported as a genus of bacteria involved in both nitrification and denitrification [28]. and *Zoogloea* is a denitrifier [29]. The relative abundances of *Azoarcus*, *Thauera*, and *Zoogloea* decreased as the TC concentration increased, indicating that these genera had poor tolerance to TC. However, when the TC concentration increased from 0 to 5 mg/L, the relative abundance of some genera that affected nitrogen removal–such as *Flavobacterium*–increased while the relative abundance of *Aquimonas*, which has been reported to degrade antibiotics [30], decreased. In addition, when the TC concentration rose to 10 mg/L, the relative abundances of *Bryobacter*, *Hyphomicrobium*, *Novosphingobium*, *Sphingorhabdus*, and *Pseudomonas* were higher than those of other genera. *Hyphomicrobium* and *Bryobacter* are tolerant to antibiotics [31,32]. *Novosphingobium* degrades aromatic compounds [33]. and *Pseudomonas* are reported as an aerobic denitrifying bacterium [34].

**Fig 2C** showed the RDA analysis of the correlation between species at the genus level and samples. When the concentration of TC was in the range of 0–10 mg/L, the increase in the concentration of TC led to changes in the microbial community at the genus level. Denitrification functions such as *Azoarcus*, *Thaurea*, *Nitrospira*, *Zoogloea*, etc. Bacteria and tetracycline concentration are negatively correlated, and the abundance decreased with the increasing of TC concentration. However, *Aquimonas*, *Hyphomicrobium*, *Bacteroides*, *Bryobacter* and other strains that were tolerant to TC were positively correlated with TC concentration, and their abundance increased with the increasing of TC concentration [35]. At the same time, we found that the concentration of tetracycline did not affect the abundance of nitrifying bacteria such as *Nitrospira* and *Nitrosomonas*, which indicated that it had no inhibitory effect on the nitrification reaction, when the concentration of TC was in the range of 0–10 mg/L.

Based on the above results, we found that when the TC concentration rose into 5 mg/L, the relative abundance of some denitrifying bacteria which could not live under the TC stress decreased, but the relative abundance of several denitrobacteria which had tolerant to TC increased, so the removal of nitrogen in the MBBR systems was maintained at the same level. But, the relative abundance of denitrification bacteria decreased when TC concentration rose from 5 mg/L to 10 mg/L, it led to the inhibition of the denitrification in the MBBR system. This was consistent with the previous conclusion.

### Change of denitrification functional genes

**Fig 3:**
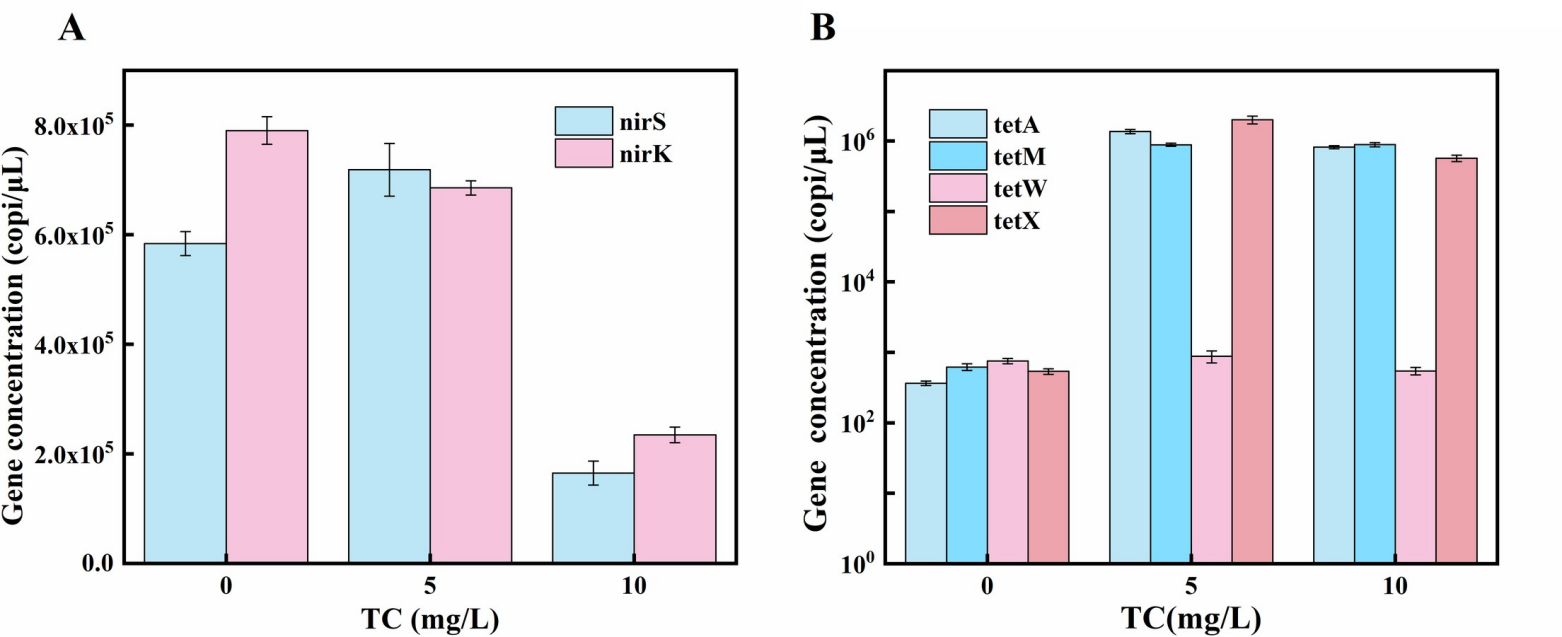
The change of denitrifications gene (A) and resistance gene (B) under TC stress.

The functional genes nirS and nirK are important for the conversion of NO_2_ to NO during denitrification [36]. As shown in **Fig 3A**. The abundance of nirS and nirK remained consisted when the TC concentration was increased to 5 mg/L. These results indicated that a TC concentration of 5 mg/L did not significantly affect the functional expression of denitrifying bacteria in the MBBR. However, the abundance of nirS and nirK decreased to one-third of their previous values when the TC concentration increased to 10 mg/L, which indicates that 10 mg/L of TC would severely inhibit denitrification genes and lead to the accumulation of nitrite-N and nitrate-N.

### Change of TC resistance gene

When TC accumulates in sewage, the accumulation and spread of antibiotic resistance genes (ARGs) is a common phenomenon. Therefore, it was necessary to detect the abundance of ARGs in the MBBR system. Four TC resistance genes (tetA, tetM, tetW, and tetX) were detected during the three study periods [37]. The abundance of the resistance genes in each period collecting from the MBBR system were showed in **Fig 3B**. The abundance of the four TC resistance genes was maintained at 10^2^–10^3^ copi/μL. When the TC concentration rose to 5 mg/L, the abundances of tetA, tetM, and tetX increased to 1.3 × 10^6^ copi/μL, 8.8 × 10^5^ copi/μL, and 1.9 × 10^6^ copi/μL, respectively; these are markedly higher levels than before. The results indicate that bacterial resistance increased under TC stress, which led to the evolution of prerequisite genes encoding drug resistance to form genomic TC resistance genes [38]. When the concentration of TC increased to 10 mg/L, however, tetA, tetM, and tetX levels did not continue to increase. This result indicated that different TC-resistant bacteria had different tolerances and responses to TC stress. The concentration of tetW remained at 10^2^–10^3^ copies/μL throughout the study period, indicating that a TC below 10 mg/L had little effect on tetW in the microbial community.

### Nitrogen balance in MBBR

**Fig 4:**
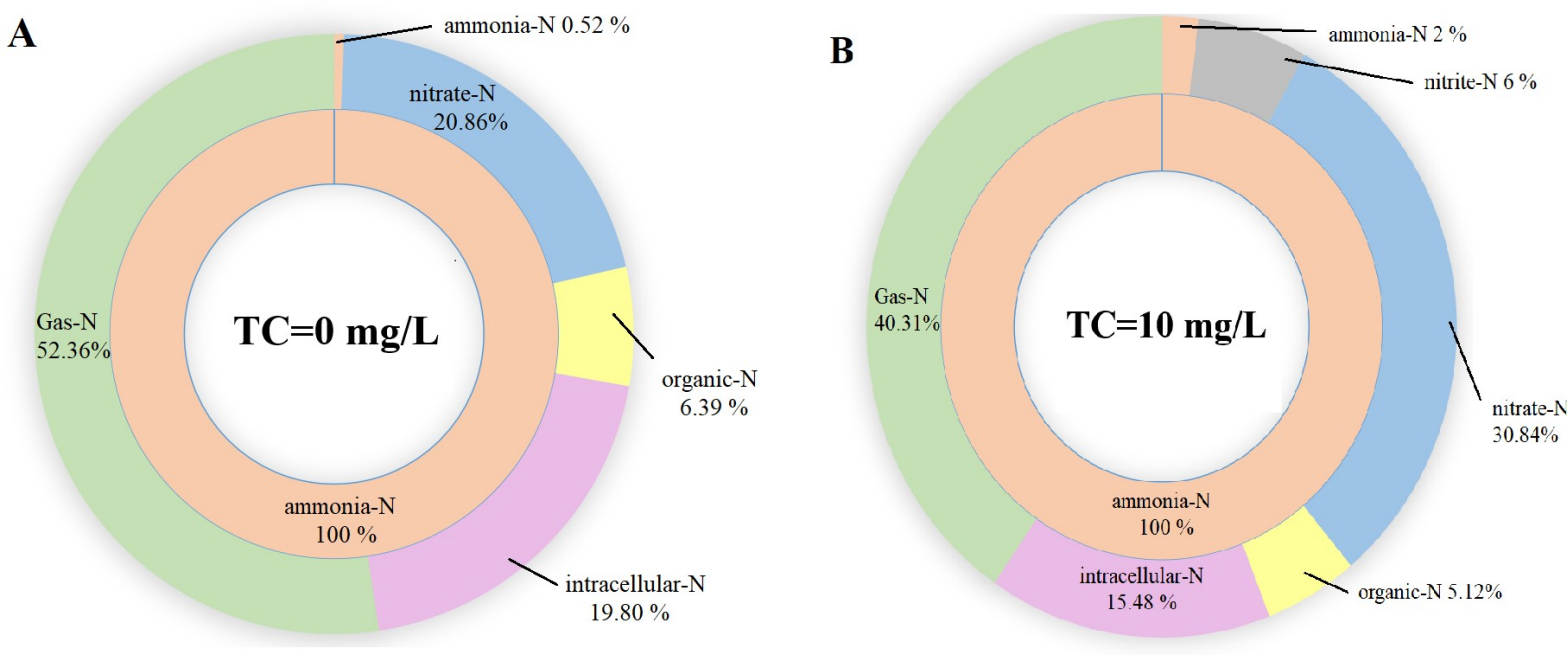
The balance of nitrogen in MBBR under TC = 0 mg/L (A) and TC = 10 mg/L (B).

The balance of nitrogen was showed in **Fig 4** and **Table 3**. The final TN remaining in the system–including ammonia-N, nitrite-N, nitrate-N, gaseous nitrogen, organic nitrogen, and intracellular nitrogen–approached the initial TN in the MBBR system; therefore, the balance of nitrogen was calculated accurately. When no TC was added to the MBBR, 19.8% of nitrogen was assimilated into intracellular nitrogen by bacteria, with a concentration of 4.89 mg/L. Nitrogen (6.39%) was converted into organic nitrogen and was present in the MBBR system. The concentrations of ammonia and nitrite in the effluent were close to 0 mg/L, while 5.15 mg/L of the nitrate was not further utilized due to the lack of electron donors and 52.36% of the nitrogen was converted into gaseous nitrogen. Following the increase in TC concentration to 10 mg/L, 15.48%, 5.12%, and 30.84% of the nitrogen was converted into intracellular nitrogen, organic nitrogen, and nitrate-N, respectively, and accumulated in the MBBR system, of which the concentration was 7.53 mg/L; 40.31% of nitrogen was converted into gaseous nitrogen. When the TC concentration was 10 mg/L, the concentrations of intracellular nitrogen and organic nitrogen decreased to lower than before. These results indicated that TC inhibitfed the growth and metabolic activity of these bacteria. Denitrification was inhibited in the MBBR system when the TC concentration was 10 mg/L, leading to the accumulation of nitrate nitrogen and nitrous nitrogen and a decrease in gaseous nitrogen. At different TC concentrations, the proportion of gaseous nitrogen was higher than that of intracellular nitrogen, indicating that the removal of nitrogen in the MBBR system was accomplished mainly by nitrification and denitrification, not assimilation.

**Table 3:** Nitrogen balance analysis of the nitrogen removal in MBBR.

TC (mg/L)	Nitrogen source	Influent NH_4_^+^-N (mg/L)	Effluent nitrogen (mg/L)					
			NH_4_^+^-N	NO_2_^-^-N	NO_3_^-^-N	Intracellular nitrogen	Organic nitrogen	Gaseous nitrogen
0	NH_4_^+^-N	24.69	0.13	0.01	5.15	4.89	1.58	1.25
10		24.41	0.51	1.5	7.53	3.78	1.25	9.84

### Carbon balance and electron flow distribution in MBBR

Part of the carbon source (C_5_H_7_NO_2_) was used for biosynthesis and the other was used as an electron donor. In the absence of TC, no nitrogen accumulated in the MBBR system. Assuming that the nitrogen removal process of the MBBR system involved complete nitrification and denitrification, the gaseous product was N_2_. The electron transfer, based on the measured TOC content and nitrogen balance calculations, was calculated as follows:

$$Biological\;charcoal=\frac{biological\;nirtrogen}{14}\times 5=1.75\;mmol/L$$ (6)

Forelectrondonor=(ΔTOC−biologicalcharcoal)×4=37.17mmol/L (7)

$$electron\;for\;nitate=\frac{N_{2}}{28}\times 5=2.31mmol/L$$ (8)

electronforoxygen=37.17−2.31=34.86mmol/L (9)

The result of the calculation indicated that 1.75 mmol/L carbon (C_5_H_7_NO_2_) was used for biosynthesis. The oxidation reaction of sodium acetate released 37.17 mmol/L electrons, of which 2.31 mmol/L was used to reduce nitrate nitrogen, 34.86 mmol/L for biological oxidation and ATP synthesis through the triphosphate cycle, leaving about 9.29% of the carbon unused. Fewer electrons were used in the reduction of nitrate nitrogen, possibly because the denitrification process requires more complex enzymes, such as nitrate reductase and sub-enzymes, than aerobic respiration that results in a low substrate conversion rate.

### Kinetics for the removal of different nitrogen types

**Fig 5:**
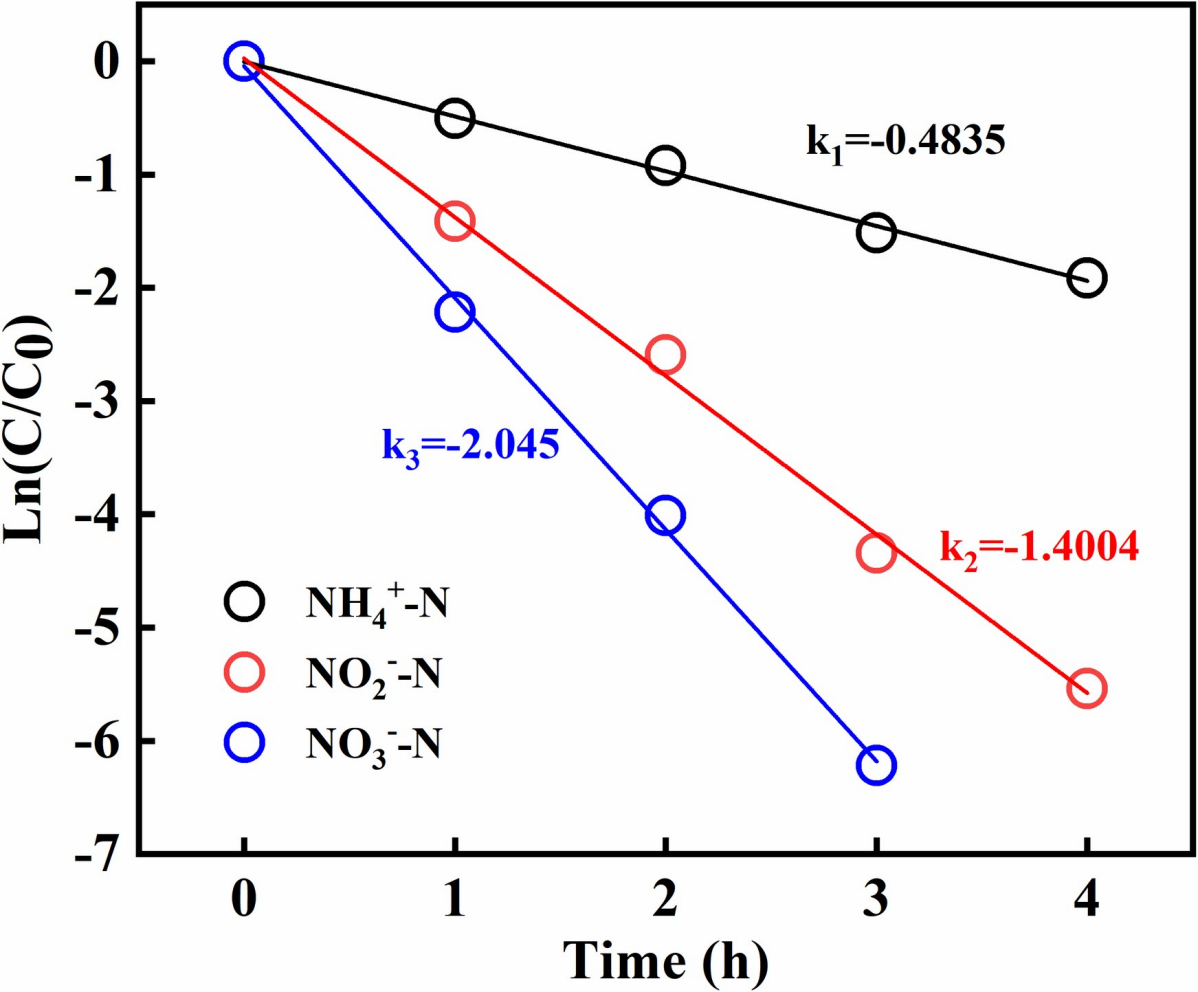
Kinetic analysis of different nitrogen sources.

In order to study the apparent kinetics with different nitrogen sources including ammonia, nitrite and nitrate, the result of experiment was showed in **Fig 5**, The apparent kinetic constants of ammonia-N, nitrate-N, and nitrite-N were 0.4835 (k_1_), 1.4004 (k_2_), and 2.045 (k_3_); the values of k_2_ and k_3_ were much higher than that of k_1_, showing that the removal of the ammonia-N was the limiting step in process of nitrogen removal in the MBBR system. Furthermore, the nitrate removal rate was faster than the nitrite removal rate, a common phenomenon, because of the higher redox potential of the former [39]. As nitrite-N was more rapidly consumed than nitrate, the former did not accumulate in the MBBR system when the nitrogen source was ammonia-N.

### Nitrogen removal mechanism under TC stress

**Fig 6:**
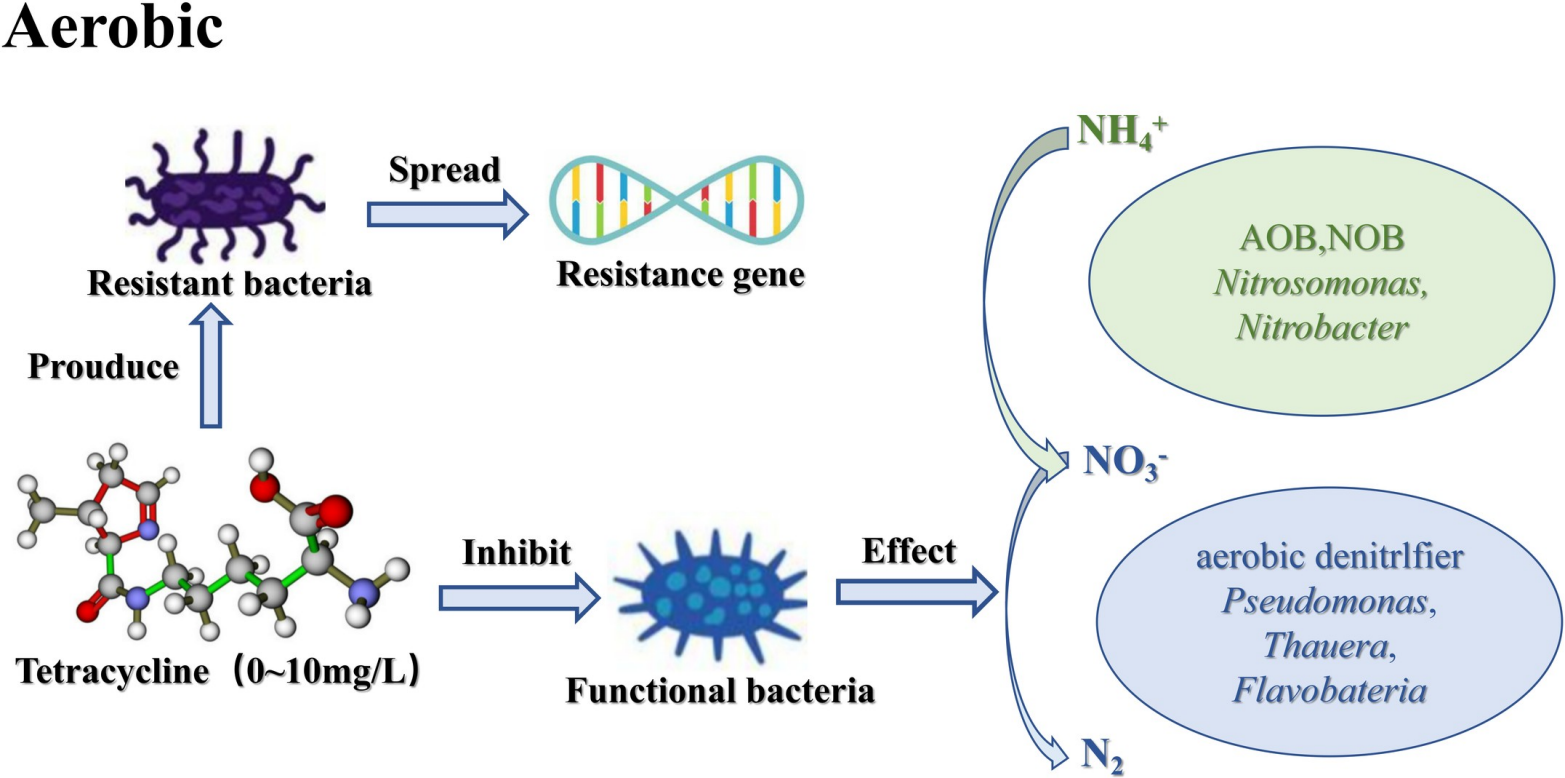
Mechanism diagram of nitrogen removal under TC stress.

The results above show TC can reduce the abundance of denitrifying bacteria by inhibiting their growth and reproduction while also reducing the abundance of the functional denitrification genes nirS and nirK. Inhibiting the expression of nitrite-reducing bacteria is an important method by which TC inhibits denitrification. The increased abundance of TC resistance genes indicates that the presence of TC will lead to the spread of the former. Based on this, the MBBR biological denitrification mechanism diagram under tetracycline stress was shown in **Fig 6**.

## Conclusions

In this study, there was no significant effect on the removal performance of nitrogen in MBBR when the concentration of TC was 5 mg/L. But when the concentration of TC increased to 10 mg/L, the denitrification effect of MBBR would be affected significantly. It was found that 5 mg/L and 10 mg/L of TC would not do harm to the diversity of microbial communities in the biofilm system. when the TC concentration was increased to 10 mg/L, the abundance of denitrifying bacteria (*Azoarcus*, *Thauera* and *Zoogloea*) was greatly reduced. The functional denitrification genes (nirS and nirK) did not change significantly when the concentration of TC was 5 mg/L, and dropped to one-third of the initial value when the concentration of TC increased to 10 mg/L. The inhibition of denitrification that occurred when the TC concentration was 10 mg/L TC occurred mainly through the inhibition of the functional expression of nitrite-reducing bacteria.

## Supporting information

S1 Data (XLS)

S2 Data (ZIP)

S3 Data (XLSX)

S4 Data (XLS)
