# Supplementary material for: Effect of tetracycline on nitrogen removal in Moving Bed Biofilm Reactor (MBBR) System
Source: PLoS One. 2022 Jan 10;17(1):e0261306. doi: 10.1371/journal.pone.0261306 (PMC8746769; doi:10.1371/journal.pone.0261306)
Supplement: S2 Data — (ZIP) [file pone.0261306.s002.zip › index.html]

微生物多样性分析结题报告

微生物多样性分析结题报告

## 微生物多样性分析结题报告

### 摘要

3 个样品测序共获得 231,654 对 Reads， 双端 Reads 质控、拼接后共产生 230,641 条 Clean Reads，每个样品至少产生 71,614 条 Clean Reads ，平均产生 76,880 条 Clean Reads。

### 1 背景介绍

微生物多样性是基于 Illumina HiSeq 测序平台，利用双末端测序（Paired-End）的方法， 构建小片段文库进行测序。通过对 Reads 拼接过滤，聚类或去噪，并进行物种注释及丰度分析，可以揭示样品的物种构成； 进一步进行α多样性分析（Alpha Diversity）、β多样性分析（Beta Diversity）、显著物种差异分析、相关性分析、功能预测分析等等，可以挖掘样品之间的差异。

目前，微生物多样性研究主要是于编码核糖体RNA的核酸序列保守区进行的。细菌主要是基于16S区，真菌主要基于18S区或ITS区（内转录间区），16S rDNA 是编码原核生物核糖体小亚基rRNA（16S rRNA）的DNA序列，18S rDNA是编码真核生物核糖体小亚基rRNA（18S rRNA）的DNA序列，ITS是编码真核生物核糖体小亚基rRNA的DNA内转录间隔区序列。这些序列中既有保守区又有可变区，保守序列区域反映了生物物种间的亲缘关系，而高变序列区域则能体现物种间的差异。由于18S rDNA在进化速率上比较保守，在系统发育研究中较适用于种级以上阶元的分类。常用作微生物分类研究的ITS分为ITS1和ITS2两种。ITS1位于真核生物核糖体rDNA序列的18S和5.8S之间，ITS2位于真核生物核糖体rDNA序列5.8S和28S之间。由于ITS区在核糖体RNA加工过程中被剪切掉，不发挥功能作用，在进化过程中选择压力较小，进化速率约为18S rDNA的10倍，属于中度保守的区域，利用它可研究种及种以下的分类阶元。另外，也可通过选择引物同时扩增18S rDNA和ITS，通过分析18S rDNA序列，先在较高级别上确定样品的归属，然后根据ITS 序列，将真菌归类到种或亚种水平。

### 2 项目概况

#### 2.1 结果概述

本项目中，微生物多样性分析包括以下操作：

1、对原始测序序列进行质控，包括低质量过滤、长度过滤，得到高质量序列；

2、将高质量序列进行聚类/去噪，划分OTUs/ASVs（后面统一称之为Feature），并根据Feature的序列组成得到其物种分类；

3、基于特征(Feature)分析结果，对样品在各个分类水平上进行分类学分析，获得各样品在门、纲、目、科、属、种分类学水平上的群落结构图、物种聚类热图、属分类学水平系统进化发生树及分类学树状图；

4、通过Alpha多样性分析研究单个样品内部的物种多样性，统计了各样品的Ace、Chao1、Shannon及Simpson指数，绘制了样品稀释曲线及等级丰度曲线；

5、通过 Beta多样性分析来比较不同样品在物种多样性方面（群落组成及结构）存在的差异大小。根据距离矩阵获得相应距离下的样品层次聚类（UPGMA）树、NMDS分析、样品聚类热图及样品PCA、PCoA图（有分组信息）、基于多种距离的箱线图等；

6、通过相关性与关联分析研究菌群与环境因子之间的关系；

7、通过功能预测分析，对样品进行基因功能或表型预测并计算功能基因或者表型丰度。

#### 2.2 样品基本信息

下表展示了各样品对应的基本信息：

表格1 样品编号对应表

| Sample ID | Sequenced Region | treat |
| --- | --- | --- |
| B1 | 16s,v3+v4\_b | B1 |
| B2 | 16s,v3+v4\_b | B2 |
| B3 | 16s,v3+v4\_b | B3 |

Sample ID：样品编号；Sequenced Region：测序区域；treat:客户提供的分组信息。

### 3 工作流程

#### 3.1 工作流程图

图1 工作流程图

注：若合同中未签某种分析或样品不适合做某种分析，则结题报告及结果文件中不会提供相应的结果。

#### 3.2 实验流程

建库测序：提取样品总DNA后，根据保守区设计得到引物，在引物末端加上测序接头，进行PCR扩增并对其产物进行纯化、定量和均一化形成测序文库，建好的文库先进行文库质检，质检合格的文库用Illumina HiSeq 2500进行测序。高通量测序（如Illumina HiSeq等测序平台）得到的原始图像数据文件，经碱基识别（Base Calling）分析转化为原始测序序列（Sequenced Reads），结果以FASTQ（简称为fq）文件格式存储，其中包含测序序列（Reads）的序列信息以及其对应的测序质量信息。

实验原理如下：

图2 实验原理图

#### 3.3 信息分析流程

数据预处理：主要有如下3个步骤：

1)Raw reads过滤：首先使用 Trimmomatic v0.33软件，对测序得到的 Raw Reads进行过滤；然后使用cutadapt 1.9.1软件进行引物序列的识别与去除，得到不包含引物序列的高质量Reads;

2)高质量Reads拼接：使用 FLASH v1.2.7软件，通过overlap对每个样品高质量的 Reads 进行拼接，得到的拼接序列即Clean Reads；

3)去除嵌合体：使用 UCHIME v4.2软件，鉴定并去除嵌合体序列，得到最终有效数据(Effective Reads)。

信息分析内容：划分Feature(OTUs、ASVs)、多样性分析、差异分析、相关性分析及功能预测分析（具体见分析结果）。

### 4 分析结果

#### 4.1 测序数据质量评估

通过统计数据处理各阶段样品序列数目，评估数据质量。主要通过统计各阶段的序列数，序列长度等参数对数据进行评估。

各样品测序数据评估结果如下表所示：Sample ID为样品名称；Raw Reads为测序得到的原始reads数；Clean Reads为原始序列质控及拼接后得到的高质量reads数；Effective Reads 为Clean Reads 过滤嵌合体后的有效序列数；AvgLen (bp)为样品平均序列长度；GC(%)为样品GC含量，即G和C类型的碱基占总碱基的百分比；Q20(%)为质量值大于等于20的碱基占总碱基数的百分比；Q30(%)为质量值大于等于30的碱基占总碱基数的百分比；Effective(%)为Effective Reads占Raw Reads的百分比。

表格2 样品测序数据处理结果统计

| Sample ID | Raw Reads | Clean Reads | Effective Reads | AvgLen(bp) | GC(%) | Q20(%) | Q30(%) | Effective(%) |
| --- | --- | --- | --- | --- | --- | --- | --- | --- |
| B2 | 71,916 | 71,614 | 66,184 | 423 | 54.47 | 99.06 | 96.08 | 92.03 |
| B1 | 79,928 | 79,564 | 74,467 | 424 | 54.97 | 99.07 | 96.14 | 93.17 |
| B3 | 79,810 | 79,463 | 74,743 | 417 | 54.67 | 99.13 | 96.33 | 93.65 |

长度分布图统计了质控过滤后，各样品中相应长度范围内的序列数。序列长度分布图如下：

图3 序列长度分布图

注：横坐标为长度范围，纵坐标为Reads数。

结果文件：data\_assement

#### 4.2 OTU/ASV分析

OTU即分类操作单元，是在系统发生学研究或群体遗传学研究中，为了便于进行分析，人为给某一个分类单元（品系，种，属，分组等）设置的同一标志。可以根据不同的相似度水平，对所有序列进行OTU划分，每个OTU对应于一种代表序列。

使用Usearch软件[1]对Reads在97.0%的相似度水平下进行聚类、获得OTU。下图显示了通过聚类得到各样品OTU的个数：柱子上面的数字即为相应样品的OTU数目。

图4 各样品特征个数分布图

注：横坐标为样品名称，纵坐标为特征的数目。

结果文件：feature\_stat.png

利用Venn[2] 图可以展示样品（数目2到5）之间共有、特有特征 数目，直观地表现出样品间特征的重合情况。结合特征所代表的物种，可以找出不同环境中的共有微生物。

根据不同分组绘制的各样本Venn如下图：不同样品用不同颜色表示，不同颜色图形之间交叠部分数字为两个样品之间共有的特征个数。

图5 特征 Venn 图

注：多个颜色图形之间交叠部分数字为多个样品之间共有特征个数，非交叠部分为各样品特有特征个数。

结果文件：venn

#### 4.3 物种注释及分类学分析

以SILVA为参考数据库使用朴素贝叶斯分类器对特征序列进行分类学注释,可得到每个特征对应的物种分类信息，进而在各水平（phylum，class，order，family，genus，species）统计各样品群落组成，利用QIIME软件生成不同分类水平上的物种丰度表，再利用R语言工具绘制成样品各分类学水平下的群落结构图。

##### 4.3.1 聚类结果展示及说明

原始特征表中可能含有极低丰度的特征（物种丰度小于0.005%），将低丰度特征过滤后得到最终的特征列表并统计出各样品中各等级的注释到物种的tags数。结果见下表：Kindom、Phylum、Class、Order、Family、Genus、Species分别代表分类学水平的7个等级，具体可到特征表中查找。下表为样品各等级Reads统计表，其中的值代表该样品该等级下所涵盖的总Reads数量：

表格3 样品各等级Reads统计表

| Sample | Kindom | Phylum | Class | Order | Family | Genus | Species |
| --- | --- | --- | --- | --- | --- | --- | --- |
| B2 | 54,707 | 54,707 | 54,707 | 54,707 | 54,707 | 54,707 | 54,707 |
| B1 | 63,847 | 63,847 | 63,847 | 63,847 | 63,847 | 63,847 | 63,847 |
| B3 | 60,017 | 60,017 | 60,017 | 60,017 | 60,017 | 60,017 | 60,017 |

注：Sample列为样品名称；Kindom、Phylum、Class、Order、Family、Genus、Species分别代表界、门、纲、目、科、属 、种。

下表为样品各等级物种统计表，展示了各样品中各等级的物种类型数目：

表格4 样品各等级物种统计表

| Sample | Kindom | Phylum | Class | Order | Family | Genus | Species |
| --- | --- | --- | --- | --- | --- | --- | --- |
| B2 | 1 | 22 | 49 | 109 | 177 | 308 | 323 |
| B1 | 1 | 21 | 47 | 105 | 176 | 298 | 311 |
| B3 | 1 | 24 | 51 | 125 | 208 | 369 | 392 |
| Total | 1 | 26 | 58 | 133 | 219 | 389 | 413 |

注：Sample列为样品名称；Kindom、Phylum、Class、Order、Family、Genus、Species分别代表界、门、纲、目、科、属 、种。

结果文件：otus

##### 4.3.2 物种分布柱状(饼)图

当只有1个样品或分组时，物种分布以饼图展示，否则以柱状图展示。下图是各水平物种分布柱状(饼)图：从左至右依次为门、纲、目、科、属、种水平；一种颜色代表一个物种，色块长度（柱状图）或色块面积（饼图）表示物种所占相对丰度比例；为使视图效果最佳，只显示丰度水平前十的物种，并将其他物种合并为 Others 在图中显示，Unclassified代表未得到分类学注释的物种，具体物种信息可在相应分类等级中的物种丰度表中查找。

图6 物种分布图

注：横坐标为样品名称；纵坐标为相对丰度百分比。

结果文件：sample\_taxa

##### 4.3.3 物种丰度聚类热图

Heatmap是以颜色梯度来代表数据矩阵中数值的大小并根据物种或样品丰度相似性进行聚类的一种图形展示方式。将高丰度和低丰度的物种分块聚集，通过颜色梯度及相似程度来反映多个样品群落组成的相似性和差异性。根据每个样品的物种组成和相对丰度进行物种热图分析，提取每个分类学水平上的物种，利用R语言工具进行作图，分别在门、纲、目、科、属、种分类水平上进行Heatmap聚类分析。热图聚类结果中，颜色代表物种丰度；纵向聚类表示不同物种在各样品间丰度的相似情况，两物种间距离越近，枝长越短，说明这两个物种在各样品间的丰度越相似；横向聚类表示不同样品的各物种丰度的相似情况，与纵向聚类一样，两样品间距离越近，枝长越短，说明这两个样品的各物种丰度越相似。

样品各分类学水平下的物种丰度聚类热图如下：从左至右依次为门、纲、目、科、属、种水平；热图对应的值为每一行物种的相对丰度经过标准化处理后得到的Z值，颜色梯度由蓝色到红色表示相对丰度由低到高；如有样品分组信息，图中前两行为样品分组信息（如只有一种分组情况，则只有一行），颜色与图列对应。

图7 物种丰度聚类热图

注：横向聚类为样品信息，纵向聚类为物种信息；左侧聚类树为物种聚类树，上方的聚类树为样品聚类树；中间为热图。

结果文件：sample\_taxa

##### 4.3.4 系统进化树

用QIIME软件挑选出属分类学水平上丰度最高的特征的序列作为代表序列，进行多重序列比对并构建系统进化树，然后通过Python语言工具绘制图形。进化树中每条树枝代表一个物种，树枝长度为两个物种间的进化距离，即物种的差异程度。

下图为特征在属分类学水平上的系统进化树：环形图为物种进化树，相同颜色属名代表所属于相同的门。

图8 物种系统进化树

注：右上角图例为门水平物种名称。

结果文件：genus.rep.png

##### 4.3.5 MEGAN分类学树状图

根据NCBI提供的已有微生物物种的分类学数据库，使用MEGAN[3]软件将测序得到的物种丰度信息回归至数据库的分类学系统关系树中，从整个分类系统上全面了解样品中所有微生物的进化关系和丰度差异。具体可分为单样品分类学分析和多样品分类学分析，单样品分析可以了解单个样品中的序列在各个分类学水平上的分布情况，多样品分析可以比较多个样品在不同分类学分支上序列丰度的差异。

单样品、多样品分类学树状图示例如下：

1）多样品分类学树状图比对不同组或样品在某分支上的序列丰度差异，通过带颜色的饼状图呈现，饼状图的面积越大，说明在分支处的序列丰度越大，不同的颜色代表不同组或样品；某颜色的扇形面积越大，说明在该分支上，其对应组或样品的序列数比其他组或样品多。

2）单样品分类学树状图中不同的层次反映NCBI不同的分类学水平；分支处的圆面积代表了分布在该分类学水平且无法继续往下级水平比对的序列数量，面积越大，说明此类序列越多。

图9 MEGAN分类学树状图示例图

注：左图为多样品分类学树状图示例；右图为单样品分类学树状图示例。

结果文件：Taxa tree

##### 4.3.6 KRONA物种注释

使用KRONA 对物种注释结果进行可视化展示, 展示结果中，圆圈从内到外依次代表不同的分类级别，扇形的大小代表不同分类注释结果的相对比例。示例图如下所示：

图10 KRONA示例图

结果文件：KRONA

#### 4.4 Alpha多样性分析

Alpha多样性（Alpha diversity）反映的是单个样品物种丰度（richness）及物种多样性（diversity），有多种衡量指标：Chao1、Ace、Shannon、Simpson、Coverage、PD\_whole\_tree。Chao1和Ace指数衡量物种丰度即物种数量的多少。Shannon和Simpson指数用于衡量物种多样性，受样品群落中物种丰度和物种均匀度（Community evenness）的影响。相同物种丰度的情况下，群落中各物种具有越大的均匀度，则认为群落具有越大的多样性，Shannon指数和Simpson指数值越大，说明样品的物种多样性越高[4]。另外还统计了覆盖率（Coverage），其数值越高，则样本中物种被测出的概率越高，而没有被测出的概率越低。该指数反映本次测序结果是否代表了样本中微生物的真实情况。

##### 4.4.1 Alpha多样性指数统计

使用QIIME2软件，对样品Alpha多样性指数进行评估。各样品Alpha多样性指数值统计如下表所示：

表格5 Alpha多样性指数统计

| Sample ID | Feature | ACE | Chao1 | Simpson | Shannon | PD\_whole\_tree | Coverage |
| --- | --- | --- | --- | --- | --- | --- | --- |
| B1 | 566 | 660.881 | 664.1 | 0.9274 | 5.2119 | 41.1561 | 0.9983 |
| B2 | 573 | 637.3739 | 636.9844 | 0.9508 | 5.6943 | 40.3222 | 0.9983 |
| B3 | 755 | 813.7472 | 829.75 | 0.9529 | 6.707 | 48.1124 | 0.9985 |

注：Sample ID为样品名称；Feature为特征（OTUs或ASVs）的个数；Chao1、Ace、Shannon、Simpson、PD\_whole\_tree分别表示各个指数；Coverage是样本文库的覆盖率。

##### 4.4.2 稀释性曲线(Rarefaction Curve)

稀释性曲线[5](Rarefaction Curve)从样本中随机抽取一定数量的序列，统计这些序列所代表的物种数目，并以序列数与物种数来构建曲线，用于验证测序数据量是否足以反映样品中的物种多样性，并间接反映样品中物种的丰富程度。下图反映了持续抽样下新特征（新物种）出现的速率：在一定范围内，随着测序条数的加大，若曲线表现为急剧上升则表示群落中有大量物种被发现；当曲线趋于平缓，则表示此环境中的物种并不会随测序数量的增加而显著增多。稀释曲线可以作为对各样本测序量是否充分的判断,曲线急剧上升表明测序量不足，需要增加序列条数；反之，则表明样品序列充分，可以进行数据分析。

图11 样品稀释曲线

注：横坐标为随机抽取的测序条数，纵坐标为基于该测序条数得到的Feature数量,每条曲线代表一个样品，用不同颜色标记。

结果文件：rarefaction.curve

##### 4.4.3 香农指数曲线(Shannon Index)

利用 Mothur 软件和R语言工具依据各样品的测序量在不同测序深度时的Shannon指数（反映样品中微生物多样性的指数）绘制Shannon多样性指数稀释曲线，以此反映各样本在不同测序数量时的微生物多样性。Shannon index越大则物种种类越多，物种越丰富，表明样品中已涵盖绝大多数的微生物物种信息。当曲线趋向平坦时，说明测序数据量足够大，特征种类不会在随测序量增加而增长；如果曲线没有趋于平坦，则表明不饱和，增加数据量可以发现更多特征。结果见下图：每条曲线代表一个样品，用不同颜色标记。

图12 样品Shannon Index曲线

注：横坐标为从某个样品中随机抽取的测序条数，纵坐标为 Shannon index 指数。随着测序量增加发现的物种增多，直到物种饱和后，增加抽样条数也并不能发现新的特征。

结果文件：shannon.index

##### 4.4.4 等级丰度曲线(Rank Abundance Curve)

等级丰度曲线[6] (Rank Abundance Curve) 是将各样品的 Feature 丰度按大小排序并基于其相对丰度绘制的曲线图，主要用于同时解释样品所含物种的丰富度和均匀度，物种的丰富度由曲线在横轴上的长度来反映，曲线越宽，表示物种的组成越丰富；物种组成的均匀度由曲线的形状来反映，曲线越平坦，表示物种组成的均匀程度越高，如下图：每条曲线对应一个样品，用不同颜色标记。

图13 样品Rank-Abundance曲线图

注：横坐标为按Features丰度排序的序号，纵坐标为对应的Features的相对丰度。

结果文件：Rank-Abundance

#### 4.5 Beta多样性分析

使用QIIME软件进行 Beta 多样性(Beta diversity)分析，比较不同样品在物种多样性方面存在的相似程度。Beta多样性分析主要采用 binary jaccard、 bray curtis、 weighted unifrac(限细菌)、 unweighted unifrac (限细菌)等4种算法计算样品间的距离从而获得样本间的β值。这四个算法主要分为两大类：加权（Bray-Curtis和Weighted Unifrac）与非加权（Jaccard和Unweightde Unifrac）。利用非加权的计算方法，主要比较的是物种的有无，如果两个群体的β多样性越小,则说明两个群体的物种类型越相似。而加权方法，则需要同时考虑物种有无和物种丰度两个问题。

图14 常见微生物β多样性算法分类

从上图分类可以看出，binary jaccard和bray curtis算法仅基于样品所包含的序列特征比较物种差异（即基于Feature进行比较），而UniFrac [7]则利用了系统进化的信息（即基于系统发生树进行比较），考虑了物种间的进化距离来比较样品间的物种群落差异（限细菌）。此时基于binary jaccard和bray curtis的计算方式认为各Feature序列之间是相互独立的不存在进化关系（各Feature间关系平等）；而基于系统发生树的计算方法，会根据16S的序列信息对Feature进行进化树分类（目前仅支持16S测序数据的系统发生树距离计算，ITS系统发育信息不完善），此时不同Feature之间的距离实际上有“远近”之分。

建议：在微生物多样性分析中，由于环境中的微生物复杂多样，各环境间物种的组成差异更为剧烈，所以通常采用非加权方法进行分析。但如果要研究对照与实验处理组之间的关系采用非加权分析比较不出明显差异，则更推荐加权分析方式。两类分析方法实际上是没有好坏之分，主要依据不同的研究目的选用更为适合的方法进行数据分析，Beta多样性分析采用多种算法进行分析，为您提供了全面的分析结果，您可以在分析结果中挑选最能解释生物学问题的方法即可。

以下是基于这4种距离矩阵展开的Beta多样性分析，主要有以下几点：

1）PCA与PCoA分析——通过一系列的特征值和特征向量进行排序，选择主要的前几位特征值，采取降维的思想，找到距离矩阵中最主要的坐标，从而观察个体或群体间的差异。

2）NMDS分析——将对象间的相似性或相异性数据看成点间距离的单调函数，在保持原始数据次序关系的基础上，用新的相同次序的数据列替换原始数据进行度量型多维尺度分析，用于比对样本组之间的差异。

3）UPGMA分析——基于各样品序列间的进化信息的差异来计算样品间的差异，可以反映样品在进化树中是否有显著的微生物群落差异。

4）组间物种差异热图——基于Feature-Table和上述4种距离矩阵进行物种聚类，从聚类中可以了解样品之间的相似性以及各分类水平上的群落构成相似性。

##### 4.5.1 PCoA分析

主坐标分析法[8] (Principal coordinates analysis,PCoA)是一种与PCA类似的降维排序方法，原理是假设对N个样品有衡量它们之间差异或距离的数据，就可以用此方法找出一个直角坐标系，将N个样品表示成N个点，而使点间的欧式距离的平方正好等于原来的差异数据，实现定性数据的定量转换，从多维数据中提取出最主要的元素和结构。通过主坐标分析可以实现多个样品的分类，进一步展示样品间物种多样性差异。

基于Beta多样性分析得到的四种距离矩阵，使用R语言工具分别绘制的PCoA分析结果如下图：坐标图上距离越近的样品，相似性越大。

图15 PCoA分析图

注：点分别表示各样品；不同颜色代表不同分组；横、纵坐标为导致样品间差异最大的两个特征值，以百分数的形式体现主要影响程度。

结果文件：pcoa

##### 4.5.2 UPGMA分析

UPGMA（Unweighted Pair-group Method with Arithmetic Mean）即：非加权组平均法，也可理解为样品层次聚类，是一种常用的聚类分析方法。其原理是：假定的前条件是在进化过程中，每一世系发生趋异的次数相同，即核苷酸或氨基酸的替换速率是均等且恒定的。通过UPGMA法所产生的系统发生树可以说是物种树的简单体现，在每一次趋异发生后，从共同祖先节点到2个特征间的支的长度一样。因此，这种方法较多地用于物种树的重建。

基于Beta多样性分析得到的四种距离矩阵，通过R语言工具采用非加权配对平均法（UPGMA）对样品进行层次聚类，以判断各样品间物种组成的相似性。样品层次聚类树如下图：样品越靠近，枝长越短，说明两个样品的物种组成越相似。

图16 样品UPGMA聚类树

注：不同颜色代表不同分组。

结果文件：upgma

##### 4.5.3 UPGMA聚类树与柱状图结合绘图

UPGMA聚类树与柱状图结合绘图是将聚类树与丰度柱状图结合起来展示的一种分析手段。左图为样品聚类树（同UPGMA）：基于Beta多样性分析得到的四种距离矩阵，通过Python语言工具对样品进行层次聚类，用以判断各样品间物种组成的相似性；右图为各样品属水平的丰度柱状图，用以判断各样品间物种丰度的相似性。

样品层次聚类树如下图：样品聚类树——样品越靠近，枝长越短，说明两个样品的物种组成越相似；丰度柱状图——根据各色块所占比例比较各样品的物种多样性高低、丰度相似性以及优势物种。

图17 聚类树柱状图组合图

注：左下方图注颜色代表聚类树样品所在分组的颜色，右上方图注代按表物种丰度排名前10的物种，其他归为Others，未注释到的物种归为Unclassified。

结果文件：ClusterTree\_bar

##### 4.5.4 样品热图分析

Heatmap是基于距离算法（binary、bray、weighted、unweighted）得到样品间的距离矩阵，通过R语言工具绘制样品热图，可根据颜色梯度的变化直观看出两两样品间的差异性。

样品聚类热图如下：颜色梯度由蓝色到红色表示样品间距离由近到远。

图18 样品丰度热图

注：如有样品分组信息，图中前两行为样品分组信息（如只有一种分组情况，则只有一行），颜色与图列对应。

结果文件：heatmap

#### 4.6 相关性与关联分析

##### 4.6.1 相关性网络分析

网络图是相关性分析的一种表现形式，根据各个物种在各个样品中的丰度以及变化情况，进行斯皮尔曼(Spearman )秩相关分析并筛选相关性大于0.1且p值小于0.05的数据构建相关性网络。基于网络图的分析，可以获得物种在环境样本中的共存关系，得到物种在同一环境下的相互作用的情况及重要的模式信息，进一步解释样本间表型差异的形成机制。

网络通常由边和节点构成，一条边由两个节点连接而成，边属性统计结果见下表：

表格6 边属性表

| Source | Target | weight | color |
| --- | --- | --- | --- |
| Zoogloea | OLB17 | 1.0 | negative |
| Zoogloea | Plasticicumulans | 1.0 | positive |
| Zoogloea | Thauera | 1.0 | positive |
| Zoogloea | Azoarcus | 1.0 | positive |
| Zoogloea | Nitrosomonas | 1.0 | negative |
| Zoogloea | Bacteroidetes\_bacterium\_OLB9 | 1.0 | negative |
| Zoogloea | Nitrospira | 1.0 | positive |
| Zoogloea | Hydrogenophaga | 1.0 | negative |
| Zoogloea | SWB02 | 1.0 | negative |
| Zoogloea | SM1A02 | 1.0 | negative |
| Zoogloea | Rhodobacter | 1.0 | negative |
| Zoogloea | Brevundimonas | 1.0 | negative |
| Zoogloea | Pseudoxanthomonas | 1.0 | negative |
| Zoogloea | Bacillus | 1.0 | positive |
| Zoogloea | Ellin6067 | 1.0 | negative |
| Zoogloea | Pseudofulvimonas | 1.0 | negative |
| Zoogloea | Bacteroidetes\_bacterium\_37-13 | 1.0 | negative |
| Zoogloea | Akkermansia | 1.0 | negative |
| Zoogloea | Sphingorhabdus | 1.0 | negative |
| Zoogloea | Paracoccus | 1.0 | negative |
| Zoogloea | Terrimonas | 1.0 | negative |
| Zoogloea | Mariniradius | 1.0 | positive |
| Zoogloea | Sphingopyxis | 1.0 | negative |
| Zoogloea | Phreatobacter | 1.0 | negative |
| Zoogloea | Gemmobacter | 1.0 | negative |
| Zoogloea | Bdellovibrio | 1.0 | negative |
| Zoogloea | Lactobacillus | 1.0 | negative |
| Zoogloea | Novosphingobium | 1.0 | negative |
| Zoogloea | Faecalibaculum | 1.0 | negative |
| Zoogloea | Acinetobacter | 1.0 | negative |
| Zoogloea | Arenimonas | 1.0 | negative |
| Zoogloea | JGI\_0001001-H03 | 1.0 | negative |
| Zoogloea | Alishewanella | 1.0 | positive |
| Zoogloea | Reyranella | 1.0 | negative |
| Zoogloea | Persicitalea | 1.0 | negative |
| Zoogloea | Agathobacter | 1.0 | negative |
| Zoogloea | Hirschia | 1.0 | negative |
| Zoogloea | Sediminibacterium | 1.0 | negative |
| Zoogloea | Sphingosinicella | 1.0 | negative |
| Zoogloea | Streptococcus | 1.0 | negative |
| Zoogloea | Methyloversatilis | 1.0 | positive |
| Zoogloea | Longilinea | 1.0 | negative |
| Zoogloea | OLB13 | 1.0 | positive |
| Zoogloea | Lentimicrobium | 1.0 | negative |
| Zoogloea | Clostridium\_sensu\_stricto\_1 | 1.0 | negative |
| Zoogloea | Acidithiobacillus | 1.0 | negative |
| Zoogloea | [Eubacterium]\_coprostanoligenes\_group | 1.0 | negative |
| OLB17 | Plasticicumulans | 1.0 | negative |
| OLB17 | Thauera | 1.0 | negative |
| OLB17 | Azoarcus | 1.0 | negative |
| OLB17 | Nitrosomonas | 1.0 | positive |
| OLB17 | Bacteroidetes\_bacterium\_OLB9 | 1.0 | positive |
| OLB17 | Nitrospira | 1.0 | negative |
| OLB17 | Hydrogenophaga | 1.0 | positive |
| OLB17 | SWB02 | 1.0 | positive |
| OLB17 | SM1A02 | 1.0 | positive |
| OLB17 | Rhodobacter | 1.0 | positive |
| OLB17 | Brevundimonas | 1.0 | positive |
| OLB17 | Pseudoxanthomonas | 1.0 | positive |
| OLB17 | Bacillus | 1.0 | negative |
| OLB17 | Ellin6067 | 1.0 | positive |
| OLB17 | Pseudofulvimonas | 1.0 | positive |
| OLB17 | Bacteroidetes\_bacterium\_37-13 | 1.0 | positive |
| OLB17 | Akkermansia | 1.0 | positive |
| OLB17 | Sphingorhabdus | 1.0 | positive |
| OLB17 | Paracoccus | 1.0 | positive |
| OLB17 | Terrimonas | 1.0 | positive |
| OLB17 | Mariniradius | 1.0 | negative |
| OLB17 | Sphingopyxis | 1.0 | positive |
| OLB17 | Phreatobacter | 1.0 | positive |
| OLB17 | Gemmobacter | 1.0 | positive |
| OLB17 | Bdellovibrio | 1.0 | positive |
| OLB17 | Lactobacillus | 1.0 | positive |
| OLB17 | Novosphingobium | 1.0 | positive |
| OLB17 | Faecalibaculum | 1.0 | positive |
| OLB17 | Acinetobacter | 1.0 | positive |
| OLB17 | Arenimonas | 1.0 | positive |
| OLB17 | JGI\_0001001-H03 | 1.0 | positive |
| OLB17 | Alishewanella | 1.0 | negative |
| OLB17 | Reyranella | 1.0 | positive |
| OLB17 | Persicitalea | 1.0 | positive |
| OLB17 | Agathobacter | 1.0 | positive |
| OLB17 | Hirschia | 1.0 | positive |
| OLB17 | Sediminibacterium | 1.0 | positive |
| OLB17 | Sphingosinicella | 1.0 | positive |
| OLB17 | Streptococcus | 1.0 | positive |
| OLB17 | Methyloversatilis | 1.0 | negative |
| OLB17 | Longilinea | 1.0 | positive |
| OLB17 | OLB13 | 1.0 | negative |
| OLB17 | Lentimicrobium | 1.0 | positive |
| OLB17 | Clostridium\_sensu\_stricto\_1 | 1.0 | positive |
| OLB17 | Acidithiobacillus | 1.0 | positive |
| OLB17 | [Eubacterium]\_coprostanoligenes\_group | 1.0 | positive |
| Plasticicumulans | Thauera | 1.0 | positive |
| Plasticicumulans | Azoarcus | 1.0 | positive |
| Plasticicumulans | Nitrosomonas | 1.0 | negative |
| Plasticicumulans | Bacteroidetes\_bacterium\_OLB9 | 1.0 | negative |
| Plasticicumulans | Nitrospira | 1.0 | positive |
| Plasticicumulans | Hydrogenophaga | 1.0 | negative |
| Plasticicumulans | SWB02 | 1.0 | negative |
| Plasticicumulans | SM1A02 | 1.0 | negative |
| Plasticicumulans | Rhodobacter | 1.0 | negative |
| Plasticicumulans | Brevundimonas | 1.0 | negative |
| Plasticicumulans | Pseudoxanthomonas | 1.0 | negative |
| Plasticicumulans | Bacillus | 1.0 | positive |
| Plasticicumulans | Ellin6067 | 1.0 | negative |
| Plasticicumulans | Pseudofulvimonas | 1.0 | negative |
| Plasticicumulans | Bacteroidetes\_bacterium\_37-13 | 1.0 | negative |
| Plasticicumulans | Akkermansia | 1.0 | negative |
| Plasticicumulans | Sphingorhabdus | 1.0 | negative |
| Plasticicumulans | Paracoccus | 1.0 | negative |
| Plasticicumulans | Terrimonas | 1.0 | negative |
| Plasticicumulans | Mariniradius | 1.0 | positive |
| Plasticicumulans | Sphingopyxis | 1.0 | negative |
| Plasticicumulans | Phreatobacter | 1.0 | negative |
| Plasticicumulans | Gemmobacter | 1.0 | negative |
| Plasticicumulans | Bdellovibrio | 1.0 | negative |
| Plasticicumulans | Lactobacillus | 1.0 | negative |
| Plasticicumulans | Novosphingobium | 1.0 | negative |
| Plasticicumulans | Faecalibaculum | 1.0 | negative |
| Plasticicumulans | Acinetobacter | 1.0 | negative |
| Plasticicumulans | Arenimonas | 1.0 | negative |
| Plasticicumulans | JGI\_0001001-H03 | 1.0 | negative |
| Plasticicumulans | Alishewanella | 1.0 | positive |
| Plasticicumulans | Reyranella | 1.0 | negative |
| Plasticicumulans | Persicitalea | 1.0 | negative |
| Plasticicumulans | Agathobacter | 1.0 | negative |
| Plasticicumulans | Hirschia | 1.0 | negative |
| Plasticicumulans | Sediminibacterium | 1.0 | negative |
| Plasticicumulans | Sphingosinicella | 1.0 | negative |
| Plasticicumulans | Streptococcus | 1.0 | negative |
| Plasticicumulans | Methyloversatilis | 1.0 | positive |
| Plasticicumulans | Longilinea | 1.0 | negative |
| Plasticicumulans | OLB13 | 1.0 | positive |
| Plasticicumulans | Lentimicrobium | 1.0 | negative |
| Plasticicumulans | Clostridium\_sensu\_stricto\_1 | 1.0 | negative |
| Plasticicumulans | Acidithiobacillus | 1.0 | negative |
| Plasticicumulans | [Eubacterium]\_coprostanoligenes\_group | 1.0 | negative |
| Thauera | Azoarcus | 1.0 | positive |
| Thauera | Nitrosomonas | 1.0 | negative |
| Thauera | Bacteroidetes\_bacterium\_OLB9 | 1.0 | negative |
| Thauera | Nitrospira | 1.0 | positive |
| Thauera | Hydrogenophaga | 1.0 | negative |
| Thauera | SWB02 | 1.0 | negative |
| Thauera | SM1A02 | 1.0 | negative |
| Thauera | Rhodobacter | 1.0 | negative |
| Thauera | Brevundimonas | 1.0 | negative |
| Thauera | Pseudoxanthomonas | 1.0 | negative |
| Thauera | Bacillus | 1.0 | positive |
| Thauera | Ellin6067 | 1.0 | negative |
| Thauera | Pseudofulvimonas | 1.0 | negative |
| Thauera | Bacteroidetes\_bacterium\_37-13 | 1.0 | negative |
| Thauera | Akkermansia | 1.0 | negative |
| Thauera | Sphingorhabdus | 1.0 | negative |
| Thauera | Paracoccus | 1.0 | negative |
| Thauera | Terrimonas | 1.0 | negative |
| Thauera | Mariniradius | 1.0 | positive |
| Thauera | Sphingopyxis | 1.0 | negative |
| Thauera | Phreatobacter | 1.0 | negative |
| Thauera | Gemmobacter | 1.0 | negative |
| Thauera | Bdellovibrio | 1.0 | negative |
| Thauera | Lactobacillus | 1.0 | negative |
| Thauera | Novosphingobium | 1.0 | negative |
| Thauera | Faecalibaculum | 1.0 | negative |
| Thauera | Acinetobacter | 1.0 | negative |
| Thauera | Arenimonas | 1.0 | negative |
| Thauera | JGI\_0001001-H03 | 1.0 | negative |
| Thauera | Alishewanella | 1.0 | positive |
| Thauera | Reyranella | 1.0 | negative |
| Thauera | Persicitalea | 1.0 | negative |
| Thauera | Agathobacter | 1.0 | negative |
| Thauera | Hirschia | 1.0 | negative |
| Thauera | Sediminibacterium | 1.0 | negative |
| Thauera | Sphingosinicella | 1.0 | negative |
| Thauera | Streptococcus | 1.0 | negative |
| Thauera | Methyloversatilis | 1.0 | positive |
| Thauera | Longilinea | 1.0 | negative |
| Thauera | OLB13 | 1.0 | positive |
| Thauera | Lentimicrobium | 1.0 | negative |
| Thauera | Clostridium\_sensu\_stricto\_1 | 1.0 | negative |
| Thauera | Acidithiobacillus | 1.0 | negative |
| Thauera | [Eubacterium]\_coprostanoligenes\_group | 1.0 | negative |
| OLB8 | Candidatus\_Competibacter | 1.0 | negative |
| OLB8 | Brevifollis | 1.0 | positive |
| OLB8 | Flavobacterium | 1.0 | positive |
| OLB8 | Algoriphagus | 1.0 | positive |
| OLB8 | OLB12 | 1.0 | negative |
| OLB8 | Prosthecobacter | 1.0 | positive |
| OLB8 | Denitratisoma | 1.0 | negative |
| OLB8 | Gemmatimonas | 1.0 | negative |
| OLB8 | Escherichia-Shigella | 1.0 | positive |
| OLB8 | Bryobacter | 1.0 | negative |
| OLB8 | Pseudomonas | 1.0 | negative |
| OLB8 | Turneriella | 1.0 | positive |
| OLB8 | Runella | 1.0 | negative |
| OLB8 | Acetobacter | 1.0 | negative |
| OLB8 | Hyphomicrobium | 1.0 | negative |
| OLB8 | [Ruminococcus]\_torques\_group | 1.0 | positive |
| OLB8 | Roseomonas | 1.0 | negative |
| OLB8 | Anaerostipes | 1.0 | positive |
| OLB8 | Rhodopseudomonas | 1.0 | negative |
| OLB8 | Stella | 1.0 | negative |
| OLB8 | Romboutsia | 1.0 | positive |
| OLB8 | Sphingomonas | 1.0 | negative |
| Candidatus\_Competibacter | Brevifollis | 1.0 | negative |
| Candidatus\_Competibacter | Flavobacterium | 1.0 | negative |
| Candidatus\_Competibacter | Algoriphagus | 1.0 | negative |
| Candidatus\_Competibacter | OLB12 | 1.0 | positive |
| Candidatus\_Competibacter | Prosthecobacter | 1.0 | negative |
| Candidatus\_Competibacter | Denitratisoma | 1.0 | positive |
| Candidatus\_Competibacter | Gemmatimonas | 1.0 | positive |
| Candidatus\_Competibacter | Escherichia-Shigella | 1.0 | negative |
| Candidatus\_Competibacter | Bryobacter | 1.0 | positive |
| Candidatus\_Competibacter | Pseudomonas | 1.0 | positive |
| Candidatus\_Competibacter | Turneriella | 1.0 | negative |
| Candidatus\_Competibacter | Runella | 1.0 | positive |
| Candidatus\_Competibacter | Acetobacter | 1.0 | positive |
| Candidatus\_Competibacter | Hyphomicrobium | 1.0 | positive |
| Candidatus\_Competibacter | [Ruminococcus]\_torques\_group | 1.0 | negative |
| Candidatus\_Competibacter | Roseomonas | 1.0 | positive |
| Candidatus\_Competibacter | Anaerostipes | 1.0 | negative |
| Candidatus\_Competibacter | Rhodopseudomonas | 1.0 | positive |
| Candidatus\_Competibacter | Stella | 1.0 | positive |
| Candidatus\_Competibacter | Romboutsia | 1.0 | negative |
| Candidatus\_Competibacter | Sphingomonas | 1.0 | positive |
| Brevifollis | Flavobacterium | 1.0 | positive |
| Brevifollis | Algoriphagus | 1.0 | positive |
| Brevifollis | OLB12 | 1.0 | negative |
| Brevifollis | Prosthecobacter | 1.0 | positive |
| Brevifollis | Denitratisoma | 1.0 | negative |
| Brevifollis | Gemmatimonas | 1.0 | negative |
| Brevifollis | Escherichia-Shigella | 1.0 | positive |
| Brevifollis | Bryobacter | 1.0 | negative |
| Brevifollis | Pseudomonas | 1.0 | negative |
| Brevifollis | Turneriella | 1.0 | positive |
| Brevifollis | Runella | 1.0 | negative |
| Brevifollis | Acetobacter | 1.0 | negative |
| Brevifollis | Hyphomicrobium | 1.0 | negative |
| Brevifollis | [Ruminococcus]\_torques\_group | 1.0 | positive |
| Brevifollis | Roseomonas | 1.0 | negative |
| Brevifollis | Anaerostipes | 1.0 | positive |
| Brevifollis | Rhodopseudomonas | 1.0 | negative |
| Brevifollis | Stella | 1.0 | negative |
| Brevifollis | Romboutsia | 1.0 | positive |
| Brevifollis | Sphingomonas | 1.0 | negative |
| Flavobacterium | Algoriphagus | 1.0 | positive |
| Flavobacterium | OLB12 | 1.0 | negative |
| Flavobacterium | Prosthecobacter | 1.0 | positive |
| Flavobacterium | Denitratisoma | 1.0 | negative |
| Flavobacterium | Gemmatimonas | 1.0 | negative |
| Flavobacterium | Escherichia-Shigella | 1.0 | positive |
| Flavobacterium | Bryobacter | 1.0 | negative |
| Flavobacterium | Pseudomonas | 1.0 | negative |
| Flavobacterium | Turneriella | 1.0 | positive |
| Flavobacterium | Runella | 1.0 | negative |
| Flavobacterium | Acetobacter | 1.0 | negative |
| Flavobacterium | Hyphomicrobium | 1.0 | negative |
| Flavobacterium | [Ruminococcus]\_torques\_group | 1.0 | positive |
| Flavobacterium | Roseomonas | 1.0 | negative |
| Flavobacterium | Anaerostipes | 1.0 | positive |
| Flavobacterium | Rhodopseudomonas | 1.0 | negative |
| Flavobacterium | Stella | 1.0 | negative |
| Flavobacterium | Romboutsia | 1.0 | positive |
| Flavobacterium | Sphingomonas | 1.0 | negative |
| Azoarcus | Nitrosomonas | 1.0 | negative |
| Azoarcus | Bacteroidetes\_bacterium\_OLB9 | 1.0 | negative |
| Azoarcus | Nitrospira | 1.0 | positive |
| Azoarcus | Hydrogenophaga | 1.0 | negative |
| Azoarcus | SWB02 | 1.0 | negative |
| Azoarcus | SM1A02 | 1.0 | negative |
| Azoarcus | Rhodobacter | 1.0 | negative |
| Azoarcus | Brevundimonas | 1.0 | negative |
| Azoarcus | Pseudoxanthomonas | 1.0 | negative |
| Azoarcus | Bacillus | 1.0 | positive |
| Azoarcus | Ellin6067 | 1.0 | negative |
| Azoarcus | Pseudofulvimonas | 1.0 | negative |
| Azoarcus | Bacteroidetes\_bacterium\_37-13 | 1.0 | negative |
| Azoarcus | Akkermansia | 1.0 | negative |
| Azoarcus | Sphingorhabdus | 1.0 | negative |
| Azoarcus | Paracoccus | 1.0 | negative |
| Azoarcus | Terrimonas | 1.0 | negative |
| Azoarcus | Mariniradius | 1.0 | positive |
| Azoarcus | Sphingopyxis | 1.0 | negative |
| Azoarcus | Phreatobacter | 1.0 | negative |
| Azoarcus | Gemmobacter | 1.0 | negative |
| Azoarcus | Bdellovibrio | 1.0 | negative |
| Azoarcus | Lactobacillus | 1.0 | negative |
| Azoarcus | Novosphingobium | 1.0 | negative |
| Azoarcus | Faecalibaculum | 1.0 | negative |
| Azoarcus | Acinetobacter | 1.0 | negative |
| Azoarcus | Arenimonas | 1.0 | negative |
| Azoarcus | JGI\_0001001-H03 | 1.0 | negative |
| Azoarcus | Alishewanella | 1.0 | positive |
| Azoarcus | Reyranella | 1.0 | negative |
| Azoarcus | Persicitalea | 1.0 | negative |
| Azoarcus | Agathobacter | 1.0 | negative |
| Azoarcus | Hirschia | 1.0 | negative |
| Azoarcus | Sediminibacterium | 1.0 | negative |
| Azoarcus | Sphingosinicella | 1.0 | negative |
| Azoarcus | Streptococcus | 1.0 | negative |
| Azoarcus | Methyloversatilis | 1.0 | positive |
| Azoarcus | Longilinea | 1.0 | negative |
| Azoarcus | OLB13 | 1.0 | positive |
| Azoarcus | Lentimicrobium | 1.0 | negative |
| Azoarcus | Clostridium\_sensu\_stricto\_1 | 1.0 | negative |
| Azoarcus | Acidithiobacillus | 1.0 | negative |
| Azoarcus | [Eubacterium]\_coprostanoligenes\_group | 1.0 | negative |
| Nitrosomonas | Bacteroidetes\_bacterium\_OLB9 | 1.0 | positive |
| Nitrosomonas | Nitrospira | 1.0 | negative |
| Nitrosomonas | Hydrogenophaga | 1.0 | positive |
| Nitrosomonas | SWB02 | 1.0 | positive |
| Nitrosomonas | SM1A02 | 1.0 | positive |
| Nitrosomonas | Rhodobacter | 1.0 | positive |
| Nitrosomonas | Brevundimonas | 1.0 | positive |
| Nitrosomonas | Pseudoxanthomonas | 1.0 | positive |
| Nitrosomonas | Bacillus | 1.0 | negative |
| Nitrosomonas | Ellin6067 | 1.0 | positive |
| Nitrosomonas | Pseudofulvimonas | 1.0 | positive |
| Nitrosomonas | Bacteroidetes\_bacterium\_37-13 | 1.0 | positive |
| Nitrosomonas | Akkermansia | 1.0 | positive |
| Nitrosomonas | Sphingorhabdus | 1.0 | positive |
| Nitrosomonas | Paracoccus | 1.0 | positive |
| Nitrosomonas | Terrimonas | 1.0 | positive |
| Nitrosomonas | Mariniradius | 1.0 | negative |
| Nitrosomonas | Sphingopyxis | 1.0 | positive |
| Nitrosomonas | Phreatobacter | 1.0 | positive |
| Nitrosomonas | Gemmobacter | 1.0 | positive |
| Nitrosomonas | Bdellovibrio | 1.0 | positive |
| Nitrosomonas | Lactobacillus | 1.0 | positive |
| Nitrosomonas | Novosphingobium | 1.0 | positive |
| Nitrosomonas | Faecalibaculum | 1.0 | positive |
| Nitrosomonas | Acinetobacter | 1.0 | positive |
| Nitrosomonas | Arenimonas | 1.0 | positive |
| Nitrosomonas | JGI\_0001001-H03 | 1.0 | positive |
| Nitrosomonas | Alishewanella | 1.0 | negative |
| Nitrosomonas | Reyranella | 1.0 | positive |
| Nitrosomonas | Persicitalea | 1.0 | positive |
| Nitrosomonas | Agathobacter | 1.0 | positive |
| Nitrosomonas | Hirschia | 1.0 | positive |
| Nitrosomonas | Sediminibacterium | 1.0 | positive |
| Nitrosomonas | Sphingosinicella | 1.0 | positive |
| Nitrosomonas | Streptococcus | 1.0 | positive |
| Nitrosomonas | Methyloversatilis | 1.0 | negative |
| Nitrosomonas | Longilinea | 1.0 | positive |
| Nitrosomonas | OLB13 | 1.0 | negative |
| Nitrosomonas | Lentimicrobium | 1.0 | positive |
| Nitrosomonas | Clostridium\_sensu\_stricto\_1 | 1.0 | positive |
| Nitrosomonas | Acidithiobacillus | 1.0 | positive |
| Nitrosomonas | [Eubacterium]\_coprostanoligenes\_group | 1.0 | positive |
| Bacteroidetes\_bacterium\_OLB9 | Nitrospira | 1.0 | negative |
| Bacteroidetes\_bacterium\_OLB9 | Hydrogenophaga | 1.0 | positive |
| Bacteroidetes\_bacterium\_OLB9 | SWB02 | 1.0 | positive |
| Bacteroidetes\_bacterium\_OLB9 | SM1A02 | 1.0 | positive |
| Bacteroidetes\_bacterium\_OLB9 | Rhodobacter | 1.0 | positive |
| Bacteroidetes\_bacterium\_OLB9 | Brevundimonas | 1.0 | positive |
| Bacteroidetes\_bacterium\_OLB9 | Pseudoxanthomonas | 1.0 | positive |
| Bacteroidetes\_bacterium\_OLB9 | Bacillus | 1.0 | negative |
| Bacteroidetes\_bacterium\_OLB9 | Ellin6067 | 1.0 | positive |
| Bacteroidetes\_bacterium\_OLB9 | Pseudofulvimonas | 1.0 | positive |
| Bacteroidetes\_bacterium\_OLB9 | Bacteroidetes\_bacterium\_37-13 | 1.0 | positive |
| Bacteroidetes\_bacterium\_OLB9 | Akkermansia | 1.0 | positive |
| Bacteroidetes\_bacterium\_OLB9 | Sphingorhabdus | 1.0 | positive |
| Bacteroidetes\_bacterium\_OLB9 | Paracoccus | 1.0 | positive |
| Bacteroidetes\_bacterium\_OLB9 | Terrimonas | 1.0 | positive |
| Bacteroidetes\_bacterium\_OLB9 | Mariniradius | 1.0 | negative |
| Bacteroidetes\_bacterium\_OLB9 | Sphingopyxis | 1.0 | positive |
| Bacteroidetes\_bacterium\_OLB9 | Phreatobacter | 1.0 | positive |
| Bacteroidetes\_bacterium\_OLB9 | Gemmobacter | 1.0 | positive |
| Bacteroidetes\_bacterium\_OLB9 | Bdellovibrio | 1.0 | positive |
| Bacteroidetes\_bacterium\_OLB9 | Lactobacillus | 1.0 | positive |
| Bacteroidetes\_bacterium\_OLB9 | Novosphingobium | 1.0 | positive |
| Bacteroidetes\_bacterium\_OLB9 | Faecalibaculum | 1.0 | positive |
| Bacteroidetes\_bacterium\_OLB9 | Acinetobacter | 1.0 | positive |
| Bacteroidetes\_bacterium\_OLB9 | Arenimonas | 1.0 | positive |
| Bacteroidetes\_bacterium\_OLB9 | JGI\_0001001-H03 | 1.0 | positive |
| Bacteroidetes\_bacterium\_OLB9 | Alishewanella | 1.0 | negative |
| Bacteroidetes\_bacterium\_OLB9 | Reyranella | 1.0 | positive |
| Bacteroidetes\_bacterium\_OLB9 | Persicitalea | 1.0 | positive |
| Bacteroidetes\_bacterium\_OLB9 | Agathobacter | 1.0 | positive |
| Bacteroidetes\_bacterium\_OLB9 | Hirschia | 1.0 | positive |
| Bacteroidetes\_bacterium\_OLB9 | Sediminibacterium | 1.0 | positive |
| Bacteroidetes\_bacterium\_OLB9 | Sphingosinicella | 1.0 | positive |
| Bacteroidetes\_bacterium\_OLB9 | Streptococcus | 1.0 | positive |
| Bacteroidetes\_bacterium\_OLB9 | Methyloversatilis | 1.0 | negative |
| Bacteroidetes\_bacterium\_OLB9 | Longilinea | 1.0 | positive |
| Bacteroidetes\_bacterium\_OLB9 | OLB13 | 1.0 | negative |
| Bacteroidetes\_bacterium\_OLB9 | Lentimicrobium | 1.0 | positive |
| Bacteroidetes\_bacterium\_OLB9 | Clostridium\_sensu\_stricto\_1 | 1.0 | positive |
| Bacteroidetes\_bacterium\_OLB9 | Acidithiobacillus | 1.0 | positive |
| Bacteroidetes\_bacterium\_OLB9 | [Eubacterium]\_coprostanoligenes\_group | 1.0 | positive |
| Nitrospira | Hydrogenophaga | 1.0 | negative |
| Nitrospira | SWB02 | 1.0 | negative |
| Nitrospira | SM1A02 | 1.0 | negative |
| Nitrospira | Rhodobacter | 1.0 | negative |
| Nitrospira | Brevundimonas | 1.0 | negative |
| Nitrospira | Pseudoxanthomonas | 1.0 | negative |
| Nitrospira | Bacillus | 1.0 | positive |
| Nitrospira | Ellin6067 | 1.0 | negative |
| Nitrospira | Pseudofulvimonas | 1.0 | negative |
| Nitrospira | Bacteroidetes\_bacterium\_37-13 | 1.0 | negative |
| Nitrospira | Akkermansia | 1.0 | negative |
| Nitrospira | Sphingorhabdus | 1.0 | negative |
| Nitrospira | Paracoccus | 1.0 | negative |
| Nitrospira | Terrimonas | 1.0 | negative |
| Nitrospira | Mariniradius | 1.0 | positive |
| Nitrospira | Sphingopyxis | 1.0 | negative |
| Nitrospira | Phreatobacter | 1.0 | negative |
| Nitrospira | Gemmobacter | 1.0 | negative |
| Nitrospira | Bdellovibrio | 1.0 | negative |
| Nitrospira | Lactobacillus | 1.0 | negative |
| Nitrospira | Novosphingobium | 1.0 | negative |
| Nitrospira | Faecalibaculum | 1.0 | negative |
| Nitrospira | Acinetobacter | 1.0 | negative |
| Nitrospira | Arenimonas | 1.0 | negative |
| Nitrospira | JGI\_0001001-H03 | 1.0 | negative |
| Nitrospira | Alishewanella | 1.0 | positive |
| Nitrospira | Reyranella | 1.0 | negative |
| Nitrospira | Persicitalea | 1.0 | negative |
| Nitrospira | Agathobacter | 1.0 | negative |
| Nitrospira | Hirschia | 1.0 | negative |
| Nitrospira | Sediminibacterium | 1.0 | negative |
| Nitrospira | Sphingosinicella | 1.0 | negative |
| Nitrospira | Streptococcus | 1.0 | negative |
| Nitrospira | Methyloversatilis | 1.0 | positive |
| Nitrospira | Longilinea | 1.0 | negative |
| Nitrospira | OLB13 | 1.0 | positive |
| Nitrospira | Lentimicrobium | 1.0 | negative |
| Nitrospira | Clostridium\_sensu\_stricto\_1 | 1.0 | negative |
| Nitrospira | Acidithiobacillus | 1.0 | negative |
| Nitrospira | [Eubacterium]\_coprostanoligenes\_group | 1.0 | negative |
| Hydrogenophaga | SWB02 | 1.0 | positive |
| Hydrogenophaga | SM1A02 | 1.0 | positive |
| Hydrogenophaga | Rhodobacter | 1.0 | positive |
| Hydrogenophaga | Brevundimonas | 1.0 | positive |
| Hydrogenophaga | Pseudoxanthomonas | 1.0 | positive |
| Hydrogenophaga | Bacillus | 1.0 | negative |
| Hydrogenophaga | Ellin6067 | 1.0 | positive |
| Hydrogenophaga | Pseudofulvimonas | 1.0 | positive |
| Hydrogenophaga | Bacteroidetes\_bacterium\_37-13 | 1.0 | positive |
| Hydrogenophaga | Akkermansia | 1.0 | positive |
| Hydrogenophaga | Sphingorhabdus | 1.0 | positive |
| Hydrogenophaga | Paracoccus | 1.0 | positive |
| Hydrogenophaga | Terrimonas | 1.0 | positive |
| Hydrogenophaga | Mariniradius | 1.0 | negative |
| Hydrogenophaga | Sphingopyxis | 1.0 | positive |
| Hydrogenophaga | Phreatobacter | 1.0 | positive |
| Hydrogenophaga | Gemmobacter | 1.0 | positive |
| Hydrogenophaga | Bdellovibrio | 1.0 | positive |
| Hydrogenophaga | Lactobacillus | 1.0 | positive |
| Hydrogenophaga | Novosphingobium | 1.0 | positive |
| Hydrogenophaga | Faecalibaculum | 1.0 | positive |
| Hydrogenophaga | Acinetobacter | 1.0 | positive |
| Hydrogenophaga | Arenimonas | 1.0 | positive |
| Hydrogenophaga | JGI\_0001001-H03 | 1.0 | positive |
| Hydrogenophaga | Alishewanella | 1.0 | negative |
| Hydrogenophaga | Reyranella | 1.0 | positive |
| Hydrogenophaga | Persicitalea | 1.0 | positive |
| Hydrogenophaga | Agathobacter | 1.0 | positive |
| Hydrogenophaga | Hirschia | 1.0 | positive |
| Hydrogenophaga | Sediminibacterium | 1.0 | positive |
| Hydrogenophaga | Sphingosinicella | 1.0 | positive |
| Hydrogenophaga | Streptococcus | 1.0 | positive |
| Hydrogenophaga | Methyloversatilis | 1.0 | negative |
| Hydrogenophaga | Longilinea | 1.0 | positive |
| Hydrogenophaga | OLB13 | 1.0 | negative |
| Hydrogenophaga | Lentimicrobium | 1.0 | positive |
| Hydrogenophaga | Clostridium\_sensu\_stricto\_1 | 1.0 | positive |
| Hydrogenophaga | Acidithiobacillus | 1.0 | positive |
| Hydrogenophaga | [Eubacterium]\_coprostanoligenes\_group | 1.0 | positive |
| Algoriphagus | OLB12 | 1.0 | negative |
| Algoriphagus | Prosthecobacter | 1.0 | positive |
| Algoriphagus | Denitratisoma | 1.0 | negative |
| Algoriphagus | Gemmatimonas | 1.0 | negative |
| Algoriphagus | Escherichia-Shigella | 1.0 | positive |
| Algoriphagus | Bryobacter | 1.0 | negative |
| Algoriphagus | Pseudomonas | 1.0 | negative |
| Algoriphagus | Turneriella | 1.0 | positive |
| Algoriphagus | Runella | 1.0 | negative |
| Algoriphagus | Acetobacter | 1.0 | negative |
| Algoriphagus | Hyphomicrobium | 1.0 | negative |
| Algoriphagus | [Ruminococcus]\_torques\_group | 1.0 | positive |
| Algoriphagus | Roseomonas | 1.0 | negative |
| Algoriphagus | Anaerostipes | 1.0 | positive |
| Algoriphagus | Rhodopseudomonas | 1.0 | negative |
| Algoriphagus | Stella | 1.0 | negative |
| Algoriphagus | Romboutsia | 1.0 | positive |
| Algoriphagus | Sphingomonas | 1.0 | negative |
| SWB02 | SM1A02 | 1.0 | positive |
| SWB02 | Rhodobacter | 1.0 | positive |
| SWB02 | Brevundimonas | 1.0 | positive |
| SWB02 | Pseudoxanthomonas | 1.0 | positive |
| SWB02 | Bacillus | 1.0 | negative |
| SWB02 | Ellin6067 | 1.0 | positive |
| SWB02 | Pseudofulvimonas | 1.0 | positive |
| SWB02 | Bacteroidetes\_bacterium\_37-13 | 1.0 | positive |
| SWB02 | Akkermansia | 1.0 | positive |
| SWB02 | Sphingorhabdus | 1.0 | positive |
| SWB02 | Paracoccus | 1.0 | positive |
| SWB02 | Terrimonas | 1.0 | positive |
| SWB02 | Mariniradius | 1.0 | negative |
| SWB02 | Sphingopyxis | 1.0 | positive |
| SWB02 | Phreatobacter | 1.0 | positive |
| SWB02 | Gemmobacter | 1.0 | positive |
| SWB02 | Bdellovibrio | 1.0 | positive |
| SWB02 | Lactobacillus | 1.0 | positive |
| SWB02 | Novosphingobium | 1.0 | positive |
| SWB02 | Faecalibaculum | 1.0 | positive |
| SWB02 | Acinetobacter | 1.0 | positive |
| SWB02 | Arenimonas | 1.0 | positive |
| SWB02 | JGI\_0001001-H03 | 1.0 | positive |
| SWB02 | Alishewanella | 1.0 | negative |
| SWB02 | Reyranella | 1.0 | positive |
| SWB02 | Persicitalea | 1.0 | positive |
| SWB02 | Agathobacter | 1.0 | positive |
| SWB02 | Hirschia | 1.0 | positive |
| SWB02 | Sediminibacterium | 1.0 | positive |
| SWB02 | Sphingosinicella | 1.0 | positive |
| SWB02 | Streptococcus | 1.0 | positive |
| SWB02 | Methyloversatilis | 1.0 | negative |
| SWB02 | Longilinea | 1.0 | positive |
| SWB02 | OLB13 | 1.0 | negative |
| SWB02 | Lentimicrobium | 1.0 | positive |
| SWB02 | Clostridium\_sensu\_stricto\_1 | 1.0 | positive |
| SWB02 | Acidithiobacillus | 1.0 | positive |
| SWB02 | [Eubacterium]\_coprostanoligenes\_group | 1.0 | positive |
| SM1A02 | Rhodobacter | 1.0 | positive |
| SM1A02 | Brevundimonas | 1.0 | positive |
| SM1A02 | Pseudoxanthomonas | 1.0 | positive |
| SM1A02 | Bacillus | 1.0 | negative |
| SM1A02 | Ellin6067 | 1.0 | positive |
| SM1A02 | Pseudofulvimonas | 1.0 | positive |
| SM1A02 | Bacteroidetes\_bacterium\_37-13 | 1.0 | positive |
| SM1A02 | Akkermansia | 1.0 | positive |
| SM1A02 | Sphingorhabdus | 1.0 | positive |
| SM1A02 | Paracoccus | 1.0 | positive |
| SM1A02 | Terrimonas | 1.0 | positive |
| SM1A02 | Mariniradius | 1.0 | negative |
| SM1A02 | Sphingopyxis | 1.0 | positive |
| SM1A02 | Phreatobacter | 1.0 | positive |
| SM1A02 | Gemmobacter | 1.0 | positive |
| SM1A02 | Bdellovibrio | 1.0 | positive |
| SM1A02 | Lactobacillus | 1.0 | positive |
| SM1A02 | Novosphingobium | 1.0 | positive |
| SM1A02 | Faecalibaculum | 1.0 | positive |
| SM1A02 | Acinetobacter | 1.0 | positive |
| SM1A02 | Arenimonas | 1.0 | positive |
| SM1A02 | JGI\_0001001-H03 | 1.0 | positive |
| SM1A02 | Alishewanella | 1.0 | negative |
| SM1A02 | Reyranella | 1.0 | positive |
| SM1A02 | Persicitalea | 1.0 | positive |
| SM1A02 | Agathobacter | 1.0 | positive |
| SM1A02 | Hirschia | 1.0 | positive |
| SM1A02 | Sediminibacterium | 1.0 | positive |
| SM1A02 | Sphingosinicella | 1.0 | positive |
| SM1A02 | Streptococcus | 1.0 | positive |
| SM1A02 | Methyloversatilis | 1.0 | negative |
| SM1A02 | Longilinea | 1.0 | positive |
| SM1A02 | OLB13 | 1.0 | negative |
| SM1A02 | Lentimicrobium | 1.0 | positive |
| SM1A02 | Clostridium\_sensu\_stricto\_1 | 1.0 | positive |
| SM1A02 | Acidithiobacillus | 1.0 | positive |
| SM1A02 | [Eubacterium]\_coprostanoligenes\_group | 1.0 | positive |
| OLB12 | Prosthecobacter | 1.0 | negative |
| OLB12 | Denitratisoma | 1.0 | positive |
| OLB12 | Gemmatimonas | 1.0 | positive |
| OLB12 | Escherichia-Shigella | 1.0 | negative |
| OLB12 | Bryobacter | 1.0 | positive |
| OLB12 | Pseudomonas | 1.0 | positive |
| OLB12 | Turneriella | 1.0 | negative |
| OLB12 | Runella | 1.0 | positive |
| OLB12 | Acetobacter | 1.0 | positive |
| OLB12 | Hyphomicrobium | 1.0 | positive |
| OLB12 | [Ruminococcus]\_torques\_group | 1.0 | negative |
| OLB12 | Roseomonas | 1.0 | positive |
| OLB12 | Anaerostipes | 1.0 | negative |
| OLB12 | Rhodopseudomonas | 1.0 | positive |
| OLB12 | Stella | 1.0 | positive |
| OLB12 | Romboutsia | 1.0 | negative |
| OLB12 | Sphingomonas | 1.0 | positive |
| Rhodobacter | Brevundimonas | 1.0 | positive |
| Rhodobacter | Pseudoxanthomonas | 1.0 | positive |
| Rhodobacter | Bacillus | 1.0 | negative |
| Rhodobacter | Ellin6067 | 1.0 | positive |
| Rhodobacter | Pseudofulvimonas | 1.0 | positive |
| Rhodobacter | Bacteroidetes\_bacterium\_37-13 | 1.0 | positive |
| Rhodobacter | Akkermansia | 1.0 | positive |
| Rhodobacter | Sphingorhabdus | 1.0 | positive |
| Rhodobacter | Paracoccus | 1.0 | positive |
| Rhodobacter | Terrimonas | 1.0 | positive |
| Rhodobacter | Mariniradius | 1.0 | negative |
| Rhodobacter | Sphingopyxis | 1.0 | positive |
| Rhodobacter | Phreatobacter | 1.0 | positive |
| Rhodobacter | Gemmobacter | 1.0 | positive |
| Rhodobacter | Bdellovibrio | 1.0 | positive |
| Rhodobacter | Lactobacillus | 1.0 | positive |
| Rhodobacter | Novosphingobium | 1.0 | positive |
| Rhodobacter | Faecalibaculum | 1.0 | positive |
| Rhodobacter | Acinetobacter | 1.0 | positive |
| Rhodobacter | Arenimonas | 1.0 | positive |
| Rhodobacter | JGI\_0001001-H03 | 1.0 | positive |
| Rhodobacter | Alishewanella | 1.0 | negative |
| Rhodobacter | Reyranella | 1.0 | positive |
| Rhodobacter | Persicitalea | 1.0 | positive |
| Rhodobacter | Agathobacter | 1.0 | positive |
| Rhodobacter | Hirschia | 1.0 | positive |
| Rhodobacter | Sediminibacterium | 1.0 | positive |
| Rhodobacter | Sphingosinicella | 1.0 | positive |
| Rhodobacter | Streptococcus | 1.0 | positive |
| Rhodobacter | Methyloversatilis | 1.0 | negative |
| Rhodobacter | Longilinea | 1.0 | positive |
| Rhodobacter | OLB13 | 1.0 | negative |
| Rhodobacter | Lentimicrobium | 1.0 | positive |
| Rhodobacter | Clostridium\_sensu\_stricto\_1 | 1.0 | positive |
| Rhodobacter | Acidithiobacillus | 1.0 | positive |
| Rhodobacter | [Eubacterium]\_coprostanoligenes\_group | 1.0 | positive |
| Prosthecobacter | Denitratisoma | 1.0 | negative |
| Prosthecobacter | Gemmatimonas | 1.0 | negative |
| Prosthecobacter | Escherichia-Shigella | 1.0 | positive |
| Prosthecobacter | Bryobacter | 1.0 | negative |
| Prosthecobacter | Pseudomonas | 1.0 | negative |
| Prosthecobacter | Turneriella | 1.0 | positive |
| Prosthecobacter | Runella | 1.0 | negative |
| Prosthecobacter | Acetobacter | 1.0 | negative |
| Prosthecobacter | Hyphomicrobium | 1.0 | negative |
| Prosthecobacter | [Ruminococcus]\_torques\_group | 1.0 | positive |
| Prosthecobacter | Roseomonas | 1.0 | negative |
| Prosthecobacter | Anaerostipes | 1.0 | positive |
| Prosthecobacter | Rhodopseudomonas | 1.0 | negative |
| Prosthecobacter | Stella | 1.0 | negative |
| Prosthecobacter | Romboutsia | 1.0 | positive |
| Prosthecobacter | Sphingomonas | 1.0 | negative |
| Brevundimonas | Pseudoxanthomonas | 1.0 | positive |
| Brevundimonas | Bacillus | 1.0 | negative |
| Brevundimonas | Ellin6067 | 1.0 | positive |
| Brevundimonas | Pseudofulvimonas | 1.0 | positive |
| Brevundimonas | Bacteroidetes\_bacterium\_37-13 | 1.0 | positive |
| Brevundimonas | Akkermansia | 1.0 | positive |
| Brevundimonas | Sphingorhabdus | 1.0 | positive |
| Brevundimonas | Paracoccus | 1.0 | positive |
| Brevundimonas | Terrimonas | 1.0 | positive |
| Brevundimonas | Mariniradius | 1.0 | negative |
| Brevundimonas | Sphingopyxis | 1.0 | positive |
| Brevundimonas | Phreatobacter | 1.0 | positive |
| Brevundimonas | Gemmobacter | 1.0 | positive |
| Brevundimonas | Bdellovibrio | 1.0 | positive |
| Brevundimonas | Lactobacillus | 1.0 | positive |
| Brevundimonas | Novosphingobium | 1.0 | positive |
| Brevundimonas | Faecalibaculum | 1.0 | positive |
| Brevundimonas | Acinetobacter | 1.0 | positive |
| Brevundimonas | Arenimonas | 1.0 | positive |
| Brevundimonas | JGI\_0001001-H03 | 1.0 | positive |
| Brevundimonas | Alishewanella | 1.0 | negative |
| Brevundimonas | Reyranella | 1.0 | positive |
| Brevundimonas | Persicitalea | 1.0 | positive |
| Brevundimonas | Agathobacter | 1.0 | positive |
| Brevundimonas | Hirschia | 1.0 | positive |
| Brevundimonas | Sediminibacterium | 1.0 | positive |
| Brevundimonas | Sphingosinicella | 1.0 | positive |
| Brevundimonas | Streptococcus | 1.0 | positive |
| Brevundimonas | Methyloversatilis | 1.0 | negative |
| Brevundimonas | Longilinea | 1.0 | positive |
| Brevundimonas | OLB13 | 1.0 | negative |
| Brevundimonas | Lentimicrobium | 1.0 | positive |
| Brevundimonas | Clostridium\_sensu\_stricto\_1 | 1.0 | positive |
| Brevundimonas | Acidithiobacillus | 1.0 | positive |
| Brevundimonas | [Eubacterium]\_coprostanoligenes\_group | 1.0 | positive |
| Denitratisoma | Gemmatimonas | 1.0 | positive |
| Denitratisoma | Escherichia-Shigella | 1.0 | negative |
| Denitratisoma | Bryobacter | 1.0 | positive |
| Denitratisoma | Pseudomonas | 1.0 | positive |
| Denitratisoma | Turneriella | 1.0 | negative |
| Denitratisoma | Runella | 1.0 | positive |
| Denitratisoma | Acetobacter | 1.0 | positive |
| Denitratisoma | Hyphomicrobium | 1.0 | positive |
| Denitratisoma | [Ruminococcus]\_torques\_group | 1.0 | negative |
| Denitratisoma | Roseomonas | 1.0 | positive |
| Denitratisoma | Anaerostipes | 1.0 | negative |
| Denitratisoma | Rhodopseudomonas | 1.0 | positive |
| Denitratisoma | Stella | 1.0 | positive |
| Denitratisoma | Romboutsia | 1.0 | negative |
| Denitratisoma | Sphingomonas | 1.0 | positive |
| Pseudoxanthomonas | Bacillus | 1.0 | negative |
| Pseudoxanthomonas | Ellin6067 | 1.0 | positive |
| Pseudoxanthomonas | Pseudofulvimonas | 1.0 | positive |
| Pseudoxanthomonas | Bacteroidetes\_bacterium\_37-13 | 1.0 | positive |
| Pseudoxanthomonas | Akkermansia | 1.0 | positive |
| Pseudoxanthomonas | Sphingorhabdus | 1.0 | positive |
| Pseudoxanthomonas | Paracoccus | 1.0 | positive |
| Pseudoxanthomonas | Terrimonas | 1.0 | positive |
| Pseudoxanthomonas | Mariniradius | 1.0 | negative |
| Pseudoxanthomonas | Sphingopyxis | 1.0 | positive |
| Pseudoxanthomonas | Phreatobacter | 1.0 | positive |
| Pseudoxanthomonas | Gemmobacter | 1.0 | positive |
| Pseudoxanthomonas | Bdellovibrio | 1.0 | positive |
| Pseudoxanthomonas | Lactobacillus | 1.0 | positive |
| Pseudoxanthomonas | Novosphingobium | 1.0 | positive |
| Pseudoxanthomonas | Faecalibaculum | 1.0 | positive |
| Pseudoxanthomonas | Acinetobacter | 1.0 | positive |
| Pseudoxanthomonas | Arenimonas | 1.0 | positive |
| Pseudoxanthomonas | JGI\_0001001-H03 | 1.0 | positive |
| Pseudoxanthomonas | Alishewanella | 1.0 | negative |
| Pseudoxanthomonas | Reyranella | 1.0 | positive |
| Pseudoxanthomonas | Persicitalea | 1.0 | positive |
| Pseudoxanthomonas | Agathobacter | 1.0 | positive |
| Pseudoxanthomonas | Hirschia | 1.0 | positive |
| Pseudoxanthomonas | Sediminibacterium | 1.0 | positive |
| Pseudoxanthomonas | Sphingosinicella | 1.0 | positive |
| Pseudoxanthomonas | Streptococcus | 1.0 | positive |
| Pseudoxanthomonas | Methyloversatilis | 1.0 | negative |
| Pseudoxanthomonas | Longilinea | 1.0 | positive |
| Pseudoxanthomonas | OLB13 | 1.0 | negative |
| Pseudoxanthomonas | Lentimicrobium | 1.0 | positive |
| Pseudoxanthomonas | Clostridium\_sensu\_stricto\_1 | 1.0 | positive |
| Pseudoxanthomonas | Acidithiobacillus | 1.0 | positive |
| Pseudoxanthomonas | [Eubacterium]\_coprostanoligenes\_group | 1.0 | positive |
| Bacillus | Ellin6067 | 1.0 | negative |
| Bacillus | Pseudofulvimonas | 1.0 | negative |
| Bacillus | Bacteroidetes\_bacterium\_37-13 | 1.0 | negative |
| Bacillus | Akkermansia | 1.0 | negative |
| Bacillus | Sphingorhabdus | 1.0 | negative |
| Bacillus | Paracoccus | 1.0 | negative |
| Bacillus | Terrimonas | 1.0 | negative |
| Bacillus | Mariniradius | 1.0 | positive |
| Bacillus | Sphingopyxis | 1.0 | negative |
| Bacillus | Phreatobacter | 1.0 | negative |
| Bacillus | Gemmobacter | 1.0 | negative |
| Bacillus | Bdellovibrio | 1.0 | negative |
| Bacillus | Lactobacillus | 1.0 | negative |
| Bacillus | Novosphingobium | 1.0 | negative |
| Bacillus | Faecalibaculum | 1.0 | negative |
| Bacillus | Acinetobacter | 1.0 | negative |
| Bacillus | Arenimonas | 1.0 | negative |
| Bacillus | JGI\_0001001-H03 | 1.0 | negative |
| Bacillus | Alishewanella | 1.0 | positive |
| Bacillus | Reyranella | 1.0 | negative |
| Bacillus | Persicitalea | 1.0 | negative |
| Bacillus | Agathobacter | 1.0 | negative |
| Bacillus | Hirschia | 1.0 | negative |
| Bacillus | Sediminibacterium | 1.0 | negative |
| Bacillus | Sphingosinicella | 1.0 | negative |
| Bacillus | Streptococcus | 1.0 | negative |
| Bacillus | Methyloversatilis | 1.0 | positive |
| Bacillus | Longilinea | 1.0 | negative |
| Bacillus | OLB13 | 1.0 | positive |
| Bacillus | Lentimicrobium | 1.0 | negative |
| Bacillus | Clostridium\_sensu\_stricto\_1 | 1.0 | negative |
| Bacillus | Acidithiobacillus | 1.0 | negative |
| Bacillus | [Eubacterium]\_coprostanoligenes\_group | 1.0 | negative |
| Bifidobacterium | Blautia | 1.0 | positive |
| Bifidobacterium | Aquimonas | 1.0 | positive |
| Bifidobacterium | Dechloromonas | 1.0 | negative |
| Bifidobacterium | Subdoligranulum | 1.0 | positive |
| Bifidobacterium | Faecalibacterium | 1.0 | positive |
| Bifidobacterium | Fusicatenibacter | 1.0 | positive |
| Bifidobacterium | Enterobacter | 1.0 | positive |
| Bifidobacterium | Erysipelotrichaceae\_UCG-003 | 1.0 | positive |
| Gemmatimonas | Escherichia-Shigella | 1.0 | negative |
| Gemmatimonas | Bryobacter | 1.0 | positive |
| Gemmatimonas | Pseudomonas | 1.0 | positive |
| Gemmatimonas | Turneriella | 1.0 | negative |
| Gemmatimonas | Runella | 1.0 | positive |
| Gemmatimonas | Acetobacter | 1.0 | positive |
| Gemmatimonas | Hyphomicrobium | 1.0 | positive |
| Gemmatimonas | [Ruminococcus]\_torques\_group | 1.0 | negative |
| Gemmatimonas | Roseomonas | 1.0 | positive |
| Gemmatimonas | Anaerostipes | 1.0 | negative |
| Gemmatimonas | Rhodopseudomonas | 1.0 | positive |
| Gemmatimonas | Stella | 1.0 | positive |
| Gemmatimonas | Romboutsia | 1.0 | negative |
| Gemmatimonas | Sphingomonas | 1.0 | positive |
| Ellin6067 | Pseudofulvimonas | 1.0 | positive |
| Ellin6067 | Bacteroidetes\_bacterium\_37-13 | 1.0 | positive |
| Ellin6067 | Akkermansia | 1.0 | positive |
| Ellin6067 | Sphingorhabdus | 1.0 | positive |
| Ellin6067 | Paracoccus | 1.0 | positive |
| Ellin6067 | Terrimonas | 1.0 | positive |
| Ellin6067 | Mariniradius | 1.0 | negative |
| Ellin6067 | Sphingopyxis | 1.0 | positive |
| Ellin6067 | Phreatobacter | 1.0 | positive |
| Ellin6067 | Gemmobacter | 1.0 | positive |
| Ellin6067 | Bdellovibrio | 1.0 | positive |
| Ellin6067 | Lactobacillus | 1.0 | positive |
| Ellin6067 | Novosphingobium | 1.0 | positive |
| Ellin6067 | Faecalibaculum | 1.0 | positive |
| Ellin6067 | Acinetobacter | 1.0 | positive |
| Ellin6067 | Arenimonas | 1.0 | positive |
| Ellin6067 | JGI\_0001001-H03 | 1.0 | positive |
| Ellin6067 | Alishewanella | 1.0 | negative |
| Ellin6067 | Reyranella | 1.0 | positive |
| Ellin6067 | Persicitalea | 1.0 | positive |
| Ellin6067 | Agathobacter | 1.0 | positive |
| Ellin6067 | Hirschia | 1.0 | positive |
| Ellin6067 | Sediminibacterium | 1.0 | positive |
| Ellin6067 | Sphingosinicella | 1.0 | positive |
| Ellin6067 | Streptococcus | 1.0 | positive |
| Ellin6067 | Methyloversatilis | 1.0 | negative |
| Ellin6067 | Longilinea | 1.0 | positive |
| Ellin6067 | OLB13 | 1.0 | negative |
| Ellin6067 | Lentimicrobium | 1.0 | positive |
| Ellin6067 | Clostridium\_sensu\_stricto\_1 | 1.0 | positive |
| Ellin6067 | Acidithiobacillus | 1.0 | positive |
| Ellin6067 | [Eubacterium]\_coprostanoligenes\_group | 1.0 | positive |
| Pseudofulvimonas | Bacteroidetes\_bacterium\_37-13 | 1.0 | positive |
| Pseudofulvimonas | Akkermansia | 1.0 | positive |
| Pseudofulvimonas | Sphingorhabdus | 1.0 | positive |
| Pseudofulvimonas | Paracoccus | 1.0 | positive |
| Pseudofulvimonas | Terrimonas | 1.0 | positive |
| Pseudofulvimonas | Mariniradius | 1.0 | negative |
| Pseudofulvimonas | Sphingopyxis | 1.0 | positive |
| Pseudofulvimonas | Phreatobacter | 1.0 | positive |
| Pseudofulvimonas | Gemmobacter | 1.0 | positive |
| Pseudofulvimonas | Bdellovibrio | 1.0 | positive |
| Pseudofulvimonas | Lactobacillus | 1.0 | positive |
| Pseudofulvimonas | Novosphingobium | 1.0 | positive |
| Pseudofulvimonas | Faecalibaculum | 1.0 | positive |
| Pseudofulvimonas | Acinetobacter | 1.0 | positive |
| Pseudofulvimonas | Arenimonas | 1.0 | positive |
| Pseudofulvimonas | JGI\_0001001-H03 | 1.0 | positive |
| Pseudofulvimonas | Alishewanella | 1.0 | negative |
| Pseudofulvimonas | Reyranella | 1.0 | positive |
| Pseudofulvimonas | Persicitalea | 1.0 | positive |
| Pseudofulvimonas | Agathobacter | 1.0 | positive |
| Pseudofulvimonas | Hirschia | 1.0 | positive |
| Pseudofulvimonas | Sediminibacterium | 1.0 | positive |
| Pseudofulvimonas | Sphingosinicella | 1.0 | positive |
| Pseudofulvimonas | Streptococcus | 1.0 | positive |
| Pseudofulvimonas | Methyloversatilis | 1.0 | negative |
| Pseudofulvimonas | Longilinea | 1.0 | positive |
| Pseudofulvimonas | OLB13 | 1.0 | negative |
| Pseudofulvimonas | Lentimicrobium | 1.0 | positive |
| Pseudofulvimonas | Clostridium\_sensu\_stricto\_1 | 1.0 | positive |
| Pseudofulvimonas | Acidithiobacillus | 1.0 | positive |
| Pseudofulvimonas | [Eubacterium]\_coprostanoligenes\_group | 1.0 | positive |
| Escherichia-Shigella | Bryobacter | 1.0 | negative |
| Escherichia-Shigella | Pseudomonas | 1.0 | negative |
| Escherichia-Shigella | Turneriella | 1.0 | positive |
| Escherichia-Shigella | Runella | 1.0 | negative |
| Escherichia-Shigella | Acetobacter | 1.0 | negative |
| Escherichia-Shigella | Hyphomicrobium | 1.0 | negative |
| Escherichia-Shigella | [Ruminococcus]\_torques\_group | 1.0 | positive |
| Escherichia-Shigella | Roseomonas | 1.0 | negative |
| Escherichia-Shigella | Anaerostipes | 1.0 | positive |
| Escherichia-Shigella | Rhodopseudomonas | 1.0 | negative |
| Escherichia-Shigella | Stella | 1.0 | negative |
| Escherichia-Shigella | Romboutsia | 1.0 | positive |
| Escherichia-Shigella | Sphingomonas | 1.0 | negative |
| Bacteroidetes\_bacterium\_37-13 | Akkermansia | 1.0 | positive |
| Bacteroidetes\_bacterium\_37-13 | Sphingorhabdus | 1.0 | positive |
| Bacteroidetes\_bacterium\_37-13 | Paracoccus | 1.0 | positive |
| Bacteroidetes\_bacterium\_37-13 | Terrimonas | 1.0 | positive |
| Bacteroidetes\_bacterium\_37-13 | Mariniradius | 1.0 | negative |
| Bacteroidetes\_bacterium\_37-13 | Sphingopyxis | 1.0 | positive |
| Bacteroidetes\_bacterium\_37-13 | Phreatobacter | 1.0 | positive |
| Bacteroidetes\_bacterium\_37-13 | Gemmobacter | 1.0 | positive |
| Bacteroidetes\_bacterium\_37-13 | Bdellovibrio | 1.0 | positive |
| Bacteroidetes\_bacterium\_37-13 | Lactobacillus | 1.0 | positive |
| Bacteroidetes\_bacterium\_37-13 | Novosphingobium | 1.0 | positive |
| Bacteroidetes\_bacterium\_37-13 | Faecalibaculum | 1.0 | positive |
| Bacteroidetes\_bacterium\_37-13 | Acinetobacter | 1.0 | positive |
| Bacteroidetes\_bacterium\_37-13 | Arenimonas | 1.0 | positive |
| Bacteroidetes\_bacterium\_37-13 | JGI\_0001001-H03 | 1.0 | positive |
| Bacteroidetes\_bacterium\_37-13 | Alishewanella | 1.0 | negative |
| Bacteroidetes\_bacterium\_37-13 | Reyranella | 1.0 | positive |
| Bacteroidetes\_bacterium\_37-13 | Persicitalea | 1.0 | positive |
| Bacteroidetes\_bacterium\_37-13 | Agathobacter | 1.0 | positive |
| Bacteroidetes\_bacterium\_37-13 | Hirschia | 1.0 | positive |
| Bacteroidetes\_bacterium\_37-13 | Sediminibacterium | 1.0 | positive |
| Bacteroidetes\_bacterium\_37-13 | Sphingosinicella | 1.0 | positive |
| Bacteroidetes\_bacterium\_37-13 | Streptococcus | 1.0 | positive |
| Bacteroidetes\_bacterium\_37-13 | Methyloversatilis | 1.0 | negative |
| Bacteroidetes\_bacterium\_37-13 | Longilinea | 1.0 | positive |
| Bacteroidetes\_bacterium\_37-13 | OLB13 | 1.0 | negative |
| Bacteroidetes\_bacterium\_37-13 | Lentimicrobium | 1.0 | positive |
| Bacteroidetes\_bacterium\_37-13 | Clostridium\_sensu\_stricto\_1 | 1.0 | positive |
| Bacteroidetes\_bacterium\_37-13 | Acidithiobacillus | 1.0 | positive |
| Bacteroidetes\_bacterium\_37-13 | [Eubacterium]\_coprostanoligenes\_group | 1.0 | positive |
| Akkermansia | Sphingorhabdus | 1.0 | positive |
| Akkermansia | Paracoccus | 1.0 | positive |
| Akkermansia | Terrimonas | 1.0 | positive |
| Akkermansia | Mariniradius | 1.0 | negative |
| Akkermansia | Sphingopyxis | 1.0 | positive |
| Akkermansia | Phreatobacter | 1.0 | positive |
| Akkermansia | Gemmobacter | 1.0 | positive |
| Akkermansia | Bdellovibrio | 1.0 | positive |
| Akkermansia | Lactobacillus | 1.0 | positive |
| Akkermansia | Novosphingobium | 1.0 | positive |
| Akkermansia | Faecalibaculum | 1.0 | positive |
| Akkermansia | Acinetobacter | 1.0 | positive |
| Akkermansia | Arenimonas | 1.0 | positive |
| Akkermansia | JGI\_0001001-H03 | 1.0 | positive |
| Akkermansia | Alishewanella | 1.0 | negative |
| Akkermansia | Reyranella | 1.0 | positive |
| Akkermansia | Persicitalea | 1.0 | positive |
| Akkermansia | Agathobacter | 1.0 | positive |
| Akkermansia | Hirschia | 1.0 | positive |
| Akkermansia | Sediminibacterium | 1.0 | positive |
| Akkermansia | Sphingosinicella | 1.0 | positive |
| Akkermansia | Streptococcus | 1.0 | positive |
| Akkermansia | Methyloversatilis | 1.0 | negative |
| Akkermansia | Longilinea | 1.0 | positive |
| Akkermansia | OLB13 | 1.0 | negative |
| Akkermansia | Lentimicrobium | 1.0 | positive |
| Akkermansia | Clostridium\_sensu\_stricto\_1 | 1.0 | positive |
| Akkermansia | Acidithiobacillus | 1.0 | positive |
| Akkermansia | [Eubacterium]\_coprostanoligenes\_group | 1.0 | positive |
| Blautia | Aquimonas | 1.0 | positive |
| Blautia | Dechloromonas | 1.0 | negative |
| Blautia | Subdoligranulum | 1.0 | positive |
| Blautia | Faecalibacterium | 1.0 | positive |
| Blautia | Fusicatenibacter | 1.0 | positive |
| Blautia | Enterobacter | 1.0 | positive |
| Blautia | Erysipelotrichaceae\_UCG-003 | 1.0 | positive |
| Sphingorhabdus | Paracoccus | 1.0 | positive |
| Sphingorhabdus | Terrimonas | 1.0 | positive |
| Sphingorhabdus | Mariniradius | 1.0 | negative |
| Sphingorhabdus | Sphingopyxis | 1.0 | positive |
| Sphingorhabdus | Phreatobacter | 1.0 | positive |
| Sphingorhabdus | Gemmobacter | 1.0 | positive |
| Sphingorhabdus | Bdellovibrio | 1.0 | positive |
| Sphingorhabdus | Lactobacillus | 1.0 | positive |
| Sphingorhabdus | Novosphingobium | 1.0 | positive |
| Sphingorhabdus | Faecalibaculum | 1.0 | positive |
| Sphingorhabdus | Acinetobacter | 1.0 | positive |
| Sphingorhabdus | Arenimonas | 1.0 | positive |
| Sphingorhabdus | JGI\_0001001-H03 | 1.0 | positive |
| Sphingorhabdus | Alishewanella | 1.0 | negative |
| Sphingorhabdus | Reyranella | 1.0 | positive |
| Sphingorhabdus | Persicitalea | 1.0 | positive |
| Sphingorhabdus | Agathobacter | 1.0 | positive |
| Sphingorhabdus | Hirschia | 1.0 | positive |
| Sphingorhabdus | Sediminibacterium | 1.0 | positive |
| Sphingorhabdus | Sphingosinicella | 1.0 | positive |
| Sphingorhabdus | Streptococcus | 1.0 | positive |
| Sphingorhabdus | Methyloversatilis | 1.0 | negative |
| Sphingorhabdus | Longilinea | 1.0 | positive |
| Sphingorhabdus | OLB13 | 1.0 | negative |
| Sphingorhabdus | Lentimicrobium | 1.0 | positive |
| Sphingorhabdus | Clostridium\_sensu\_stricto\_1 | 1.0 | positive |
| Sphingorhabdus | Acidithiobacillus | 1.0 | positive |
| Sphingorhabdus | [Eubacterium]\_coprostanoligenes\_group | 1.0 | positive |
| Aquimonas | Dechloromonas | 1.0 | negative |
| Aquimonas | Subdoligranulum | 1.0 | positive |
| Aquimonas | Faecalibacterium | 1.0 | positive |
| Aquimonas | Fusicatenibacter | 1.0 | positive |
| Aquimonas | Enterobacter | 1.0 | positive |
| Aquimonas | Erysipelotrichaceae\_UCG-003 | 1.0 | positive |
| Dechloromonas | Subdoligranulum | 1.0 | negative |
| Dechloromonas | Faecalibacterium | 1.0 | negative |
| Dechloromonas | Fusicatenibacter | 1.0 | negative |
| Dechloromonas | Enterobacter | 1.0 | negative |
| Dechloromonas | Erysipelotrichaceae\_UCG-003 | 1.0 | negative |
| Paracoccus | Terrimonas | 1.0 | positive |
| Paracoccus | Mariniradius | 1.0 | negative |
| Paracoccus | Sphingopyxis | 1.0 | positive |
| Paracoccus | Phreatobacter | 1.0 | positive |
| Paracoccus | Gemmobacter | 1.0 | positive |
| Paracoccus | Bdellovibrio | 1.0 | positive |
| Paracoccus | Lactobacillus | 1.0 | positive |
| Paracoccus | Novosphingobium | 1.0 | positive |
| Paracoccus | Faecalibaculum | 1.0 | positive |
| Paracoccus | Acinetobacter | 1.0 | positive |
| Paracoccus | Arenimonas | 1.0 | positive |
| Paracoccus | JGI\_0001001-H03 | 1.0 | positive |
| Paracoccus | Alishewanella | 1.0 | negative |
| Paracoccus | Reyranella | 1.0 | positive |
| Paracoccus | Persicitalea | 1.0 | positive |
| Paracoccus | Agathobacter | 1.0 | positive |
| Paracoccus | Hirschia | 1.0 | positive |
| Paracoccus | Sediminibacterium | 1.0 | positive |
| Paracoccus | Sphingosinicella | 1.0 | positive |
| Paracoccus | Streptococcus | 1.0 | positive |
| Paracoccus | Methyloversatilis | 1.0 | negative |
| Paracoccus | Longilinea | 1.0 | positive |
| Paracoccus | OLB13 | 1.0 | negative |
| Paracoccus | Lentimicrobium | 1.0 | positive |
| Paracoccus | Clostridium\_sensu\_stricto\_1 | 1.0 | positive |
| Paracoccus | Acidithiobacillus | 1.0 | positive |
| Paracoccus | [Eubacterium]\_coprostanoligenes\_group | 1.0 | positive |
| Bryobacter | Pseudomonas | 1.0 | positive |
| Bryobacter | Turneriella | 1.0 | negative |
| Bryobacter | Runella | 1.0 | positive |
| Bryobacter | Acetobacter | 1.0 | positive |
| Bryobacter | Hyphomicrobium | 1.0 | positive |
| Bryobacter | [Ruminococcus]\_torques\_group | 1.0 | negative |
| Bryobacter | Roseomonas | 1.0 | positive |
| Bryobacter | Anaerostipes | 1.0 | negative |
| Bryobacter | Rhodopseudomonas | 1.0 | positive |
| Bryobacter | Stella | 1.0 | positive |
| Bryobacter | Romboutsia | 1.0 | negative |
| Bryobacter | Sphingomonas | 1.0 | positive |
| Pseudomonas | Turneriella | 1.0 | negative |
| Pseudomonas | Runella | 1.0 | positive |
| Pseudomonas | Acetobacter | 1.0 | positive |
| Pseudomonas | Hyphomicrobium | 1.0 | positive |
| Pseudomonas | [Ruminococcus]\_torques\_group | 1.0 | negative |
| Pseudomonas | Roseomonas | 1.0 | positive |
| Pseudomonas | Anaerostipes | 1.0 | negative |
| Pseudomonas | Rhodopseudomonas | 1.0 | positive |
| Pseudomonas | Stella | 1.0 | positive |
| Pseudomonas | Romboutsia | 1.0 | negative |
| Pseudomonas | Sphingomonas | 1.0 | positive |
| Terrimonas | Mariniradius | 1.0 | negative |
| Terrimonas | Sphingopyxis | 1.0 | positive |
| Terrimonas | Phreatobacter | 1.0 | positive |
| Terrimonas | Gemmobacter | 1.0 | positive |
| Terrimonas | Bdellovibrio | 1.0 | positive |
| Terrimonas | Lactobacillus | 1.0 | positive |
| Terrimonas | Novosphingobium | 1.0 | positive |
| Terrimonas | Faecalibaculum | 1.0 | positive |
| Terrimonas | Acinetobacter | 1.0 | positive |
| Terrimonas | Arenimonas | 1.0 | positive |
| Terrimonas | JGI\_0001001-H03 | 1.0 | positive |
| Terrimonas | Alishewanella | 1.0 | negative |
| Terrimonas | Reyranella | 1.0 | positive |
| Terrimonas | Persicitalea | 1.0 | positive |
| Terrimonas | Agathobacter | 1.0 | positive |
| Terrimonas | Hirschia | 1.0 | positive |
| Terrimonas | Sediminibacterium | 1.0 | positive |
| Terrimonas | Sphingosinicella | 1.0 | positive |
| Terrimonas | Streptococcus | 1.0 | positive |
| Terrimonas | Methyloversatilis | 1.0 | negative |
| Terrimonas | Longilinea | 1.0 | positive |
| Terrimonas | OLB13 | 1.0 | negative |
| Terrimonas | Lentimicrobium | 1.0 | positive |
| Terrimonas | Clostridium\_sensu\_stricto\_1 | 1.0 | positive |
| Terrimonas | Acidithiobacillus | 1.0 | positive |
| Terrimonas | [Eubacterium]\_coprostanoligenes\_group | 1.0 | positive |
| Turneriella | Runella | 1.0 | negative |
| Turneriella | Acetobacter | 1.0 | negative |
| Turneriella | Hyphomicrobium | 1.0 | negative |
| Turneriella | [Ruminococcus]\_torques\_group | 1.0 | positive |
| Turneriella | Roseomonas | 1.0 | negative |
| Turneriella | Anaerostipes | 1.0 | positive |
| Turneriella | Rhodopseudomonas | 1.0 | negative |
| Turneriella | Stella | 1.0 | negative |
| Turneriella | Romboutsia | 1.0 | positive |
| Turneriella | Sphingomonas | 1.0 | negative |
| Mariniradius | Sphingopyxis | 1.0 | negative |
| Mariniradius | Phreatobacter | 1.0 | negative |
| Mariniradius | Gemmobacter | 1.0 | negative |
| Mariniradius | Bdellovibrio | 1.0 | negative |
| Mariniradius | Lactobacillus | 1.0 | negative |
| Mariniradius | Novosphingobium | 1.0 | negative |
| Mariniradius | Faecalibaculum | 1.0 | negative |
| Mariniradius | Acinetobacter | 1.0 | negative |
| Mariniradius | Arenimonas | 1.0 | negative |
| Mariniradius | JGI\_0001001-H03 | 1.0 | negative |
| Mariniradius | Alishewanella | 1.0 | positive |
| Mariniradius | Reyranella | 1.0 | negative |
| Mariniradius | Persicitalea | 1.0 | negative |
| Mariniradius | Agathobacter | 1.0 | negative |
| Mariniradius | Hirschia | 1.0 | negative |
| Mariniradius | Sediminibacterium | 1.0 | negative |
| Mariniradius | Sphingosinicella | 1.0 | negative |
| Mariniradius | Streptococcus | 1.0 | negative |
| Mariniradius | Methyloversatilis | 1.0 | positive |
| Mariniradius | Longilinea | 1.0 | negative |
| Mariniradius | OLB13 | 1.0 | positive |
| Mariniradius | Lentimicrobium | 1.0 | negative |
| Mariniradius | Clostridium\_sensu\_stricto\_1 | 1.0 | negative |
| Mariniradius | Acidithiobacillus | 1.0 | negative |
| Mariniradius | [Eubacterium]\_coprostanoligenes\_group | 1.0 | negative |
| Sphingopyxis | Phreatobacter | 1.0 | positive |
| Sphingopyxis | Gemmobacter | 1.0 | positive |
| Sphingopyxis | Bdellovibrio | 1.0 | positive |
| Sphingopyxis | Lactobacillus | 1.0 | positive |
| Sphingopyxis | Novosphingobium | 1.0 | positive |
| Sphingopyxis | Faecalibaculum | 1.0 | positive |
| Sphingopyxis | Acinetobacter | 1.0 | positive |
| Sphingopyxis | Arenimonas | 1.0 | positive |
| Sphingopyxis | JGI\_0001001-H03 | 1.0 | positive |
| Sphingopyxis | Alishewanella | 1.0 | negative |
| Sphingopyxis | Reyranella | 1.0 | positive |
| Sphingopyxis | Persicitalea | 1.0 | positive |
| Sphingopyxis | Agathobacter | 1.0 | positive |
| Sphingopyxis | Hirschia | 1.0 | positive |
| Sphingopyxis | Sediminibacterium | 1.0 | positive |
| Sphingopyxis | Sphingosinicella | 1.0 | positive |
| Sphingopyxis | Streptococcus | 1.0 | positive |
| Sphingopyxis | Methyloversatilis | 1.0 | negative |
| Sphingopyxis | Longilinea | 1.0 | positive |
| Sphingopyxis | OLB13 | 1.0 | negative |
| Sphingopyxis | Lentimicrobium | 1.0 | positive |
| Sphingopyxis | Clostridium\_sensu\_stricto\_1 | 1.0 | positive |
| Sphingopyxis | Acidithiobacillus | 1.0 | positive |
| Sphingopyxis | [Eubacterium]\_coprostanoligenes\_group | 1.0 | positive |
| Phreatobacter | Gemmobacter | 1.0 | positive |
| Phreatobacter | Bdellovibrio | 1.0 | positive |
| Phreatobacter | Lactobacillus | 1.0 | positive |
| Phreatobacter | Novosphingobium | 1.0 | positive |
| Phreatobacter | Faecalibaculum | 1.0 | positive |
| Phreatobacter | Acinetobacter | 1.0 | positive |
| Phreatobacter | Arenimonas | 1.0 | positive |
| Phreatobacter | JGI\_0001001-H03 | 1.0 | positive |
| Phreatobacter | Alishewanella | 1.0 | negative |
| Phreatobacter | Reyranella | 1.0 | positive |
| Phreatobacter | Persicitalea | 1.0 | positive |
| Phreatobacter | Agathobacter | 1.0 | positive |
| Phreatobacter | Hirschia | 1.0 | positive |
| Phreatobacter | Sediminibacterium | 1.0 | positive |
| Phreatobacter | Sphingosinicella | 1.0 | positive |
| Phreatobacter | Streptococcus | 1.0 | positive |
| Phreatobacter | Methyloversatilis | 1.0 | negative |
| Phreatobacter | Longilinea | 1.0 | positive |
| Phreatobacter | OLB13 | 1.0 | negative |
| Phreatobacter | Lentimicrobium | 1.0 | positive |
| Phreatobacter | Clostridium\_sensu\_stricto\_1 | 1.0 | positive |
| Phreatobacter | Acidithiobacillus | 1.0 | positive |
| Phreatobacter | [Eubacterium]\_coprostanoligenes\_group | 1.0 | positive |
| Runella | Acetobacter | 1.0 | positive |
| Runella | Hyphomicrobium | 1.0 | positive |
| Runella | [Ruminococcus]\_torques\_group | 1.0 | negative |
| Runella | Roseomonas | 1.0 | positive |
| Runella | Anaerostipes | 1.0 | negative |
| Runella | Rhodopseudomonas | 1.0 | positive |
| Runella | Stella | 1.0 | positive |
| Runella | Romboutsia | 1.0 | negative |
| Runella | Sphingomonas | 1.0 | positive |
| Acetobacter | Hyphomicrobium | 1.0 | positive |
| Acetobacter | [Ruminococcus]\_torques\_group | 1.0 | negative |
| Acetobacter | Roseomonas | 1.0 | positive |
| Acetobacter | Anaerostipes | 1.0 | negative |
| Acetobacter | Rhodopseudomonas | 1.0 | positive |
| Acetobacter | Stella | 1.0 | positive |
| Acetobacter | Romboutsia | 1.0 | negative |
| Acetobacter | Sphingomonas | 1.0 | positive |
| Gemmobacter | Bdellovibrio | 1.0 | positive |
| Gemmobacter | Lactobacillus | 1.0 | positive |
| Gemmobacter | Novosphingobium | 1.0 | positive |
| Gemmobacter | Faecalibaculum | 1.0 | positive |
| Gemmobacter | Acinetobacter | 1.0 | positive |
| Gemmobacter | Arenimonas | 1.0 | positive |
| Gemmobacter | JGI\_0001001-H03 | 1.0 | positive |
| Gemmobacter | Alishewanella | 1.0 | negative |
| Gemmobacter | Reyranella | 1.0 | positive |
| Gemmobacter | Persicitalea | 1.0 | positive |
| Gemmobacter | Agathobacter | 1.0 | positive |
| Gemmobacter | Hirschia | 1.0 | positive |
| Gemmobacter | Sediminibacterium | 1.0 | positive |
| Gemmobacter | Sphingosinicella | 1.0 | positive |
| Gemmobacter | Streptococcus | 1.0 | positive |
| Gemmobacter | Methyloversatilis | 1.0 | negative |
| Gemmobacter | Longilinea | 1.0 | positive |
| Gemmobacter | OLB13 | 1.0 | negative |
| Gemmobacter | Lentimicrobium | 1.0 | positive |
| Gemmobacter | Clostridium\_sensu\_stricto\_1 | 1.0 | positive |
| Gemmobacter | Acidithiobacillus | 1.0 | positive |
| Gemmobacter | [Eubacterium]\_coprostanoligenes\_group | 1.0 | positive |
| Bdellovibrio | Lactobacillus | 1.0 | positive |
| Bdellovibrio | Novosphingobium | 1.0 | positive |
| Bdellovibrio | Faecalibaculum | 1.0 | positive |
| Bdellovibrio | Acinetobacter | 1.0 | positive |
| Bdellovibrio | Arenimonas | 1.0 | positive |
| Bdellovibrio | JGI\_0001001-H03 | 1.0 | positive |
| Bdellovibrio | Alishewanella | 1.0 | negative |
| Bdellovibrio | Reyranella | 1.0 | positive |
| Bdellovibrio | Persicitalea | 1.0 | positive |
| Bdellovibrio | Agathobacter | 1.0 | positive |
| Bdellovibrio | Hirschia | 1.0 | positive |
| Bdellovibrio | Sediminibacterium | 1.0 | positive |
| Bdellovibrio | Sphingosinicella | 1.0 | positive |
| Bdellovibrio | Streptococcus | 1.0 | positive |
| Bdellovibrio | Methyloversatilis | 1.0 | negative |
| Bdellovibrio | Longilinea | 1.0 | positive |
| Bdellovibrio | OLB13 | 1.0 | negative |
| Bdellovibrio | Lentimicrobium | 1.0 | positive |
| Bdellovibrio | Clostridium\_sensu\_stricto\_1 | 1.0 | positive |
| Bdellovibrio | Acidithiobacillus | 1.0 | positive |
| Bdellovibrio | [Eubacterium]\_coprostanoligenes\_group | 1.0 | positive |
| Lactobacillus | Novosphingobium | 1.0 | positive |
| Lactobacillus | Faecalibaculum | 1.0 | positive |
| Lactobacillus | Acinetobacter | 1.0 | positive |
| Lactobacillus | Arenimonas | 1.0 | positive |
| Lactobacillus | JGI\_0001001-H03 | 1.0 | positive |
| Lactobacillus | Alishewanella | 1.0 | negative |
| Lactobacillus | Reyranella | 1.0 | positive |
| Lactobacillus | Persicitalea | 1.0 | positive |
| Lactobacillus | Agathobacter | 1.0 | positive |
| Lactobacillus | Hirschia | 1.0 | positive |
| Lactobacillus | Sediminibacterium | 1.0 | positive |
| Lactobacillus | Sphingosinicella | 1.0 | positive |
| Lactobacillus | Streptococcus | 1.0 | positive |
| Lactobacillus | Methyloversatilis | 1.0 | negative |
| Lactobacillus | Longilinea | 1.0 | positive |
| Lactobacillus | OLB13 | 1.0 | negative |
| Lactobacillus | Lentimicrobium | 1.0 | positive |
| Lactobacillus | Clostridium\_sensu\_stricto\_1 | 1.0 | positive |
| Lactobacillus | Acidithiobacillus | 1.0 | positive |
| Lactobacillus | [Eubacterium]\_coprostanoligenes\_group | 1.0 | positive |
| Novosphingobium | Faecalibaculum | 1.0 | positive |
| Novosphingobium | Acinetobacter | 1.0 | positive |
| Novosphingobium | Arenimonas | 1.0 | positive |
| Novosphingobium | JGI\_0001001-H03 | 1.0 | positive |
| Novosphingobium | Alishewanella | 1.0 | negative |
| Novosphingobium | Reyranella | 1.0 | positive |
| Novosphingobium | Persicitalea | 1.0 | positive |
| Novosphingobium | Agathobacter | 1.0 | positive |
| Novosphingobium | Hirschia | 1.0 | positive |
| Novosphingobium | Sediminibacterium | 1.0 | positive |
| Novosphingobium | Sphingosinicella | 1.0 | positive |
| Novosphingobium | Streptococcus | 1.0 | positive |
| Novosphingobium | Methyloversatilis | 1.0 | negative |
| Novosphingobium | Longilinea | 1.0 | positive |
| Novosphingobium | OLB13 | 1.0 | negative |
| Novosphingobium | Lentimicrobium | 1.0 | positive |
| Novosphingobium | Clostridium\_sensu\_stricto\_1 | 1.0 | positive |
| Novosphingobium | Acidithiobacillus | 1.0 | positive |
| Novosphingobium | [Eubacterium]\_coprostanoligenes\_group | 1.0 | positive |
| Faecalibaculum | Acinetobacter | 1.0 | positive |
| Faecalibaculum | Arenimonas | 1.0 | positive |
| Faecalibaculum | JGI\_0001001-H03 | 1.0 | positive |
| Faecalibaculum | Alishewanella | 1.0 | negative |
| Faecalibaculum | Reyranella | 1.0 | positive |
| Faecalibaculum | Persicitalea | 1.0 | positive |
| Faecalibaculum | Agathobacter | 1.0 | positive |
| Faecalibaculum | Hirschia | 1.0 | positive |
| Faecalibaculum | Sediminibacterium | 1.0 | positive |
| Faecalibaculum | Sphingosinicella | 1.0 | positive |
| Faecalibaculum | Streptococcus | 1.0 | positive |
| Faecalibaculum | Methyloversatilis | 1.0 | negative |
| Faecalibaculum | Longilinea | 1.0 | positive |
| Faecalibaculum | OLB13 | 1.0 | negative |
| Faecalibaculum | Lentimicrobium | 1.0 | positive |
| Faecalibaculum | Clostridium\_sensu\_stricto\_1 | 1.0 | positive |
| Faecalibaculum | Acidithiobacillus | 1.0 | positive |
| Faecalibaculum | [Eubacterium]\_coprostanoligenes\_group | 1.0 | positive |
| Subdoligranulum | Faecalibacterium | 1.0 | positive |
| Subdoligranulum | Fusicatenibacter | 1.0 | positive |
| Subdoligranulum | Enterobacter | 1.0 | positive |
| Subdoligranulum | Erysipelotrichaceae\_UCG-003 | 1.0 | positive |
| Acinetobacter | Arenimonas | 1.0 | positive |
| Acinetobacter | JGI\_0001001-H03 | 1.0 | positive |
| Acinetobacter | Alishewanella | 1.0 | negative |
| Acinetobacter | Reyranella | 1.0 | positive |
| Acinetobacter | Persicitalea | 1.0 | positive |
| Acinetobacter | Agathobacter | 1.0 | positive |
| Acinetobacter | Hirschia | 1.0 | positive |
| Acinetobacter | Sediminibacterium | 1.0 | positive |
| Acinetobacter | Sphingosinicella | 1.0 | positive |
| Acinetobacter | Streptococcus | 1.0 | positive |
| Acinetobacter | Methyloversatilis | 1.0 | negative |
| Acinetobacter | Longilinea | 1.0 | positive |
| Acinetobacter | OLB13 | 1.0 | negative |
| Acinetobacter | Lentimicrobium | 1.0 | positive |
| Acinetobacter | Clostridium\_sensu\_stricto\_1 | 1.0 | positive |
| Acinetobacter | Acidithiobacillus | 1.0 | positive |
| Acinetobacter | [Eubacterium]\_coprostanoligenes\_group | 1.0 | positive |
| Hyphomicrobium | [Ruminococcus]\_torques\_group | 1.0 | negative |
| Hyphomicrobium | Roseomonas | 1.0 | positive |
| Hyphomicrobium | Anaerostipes | 1.0 | negative |
| Hyphomicrobium | Rhodopseudomonas | 1.0 | positive |
| Hyphomicrobium | Stella | 1.0 | positive |
| Hyphomicrobium | Romboutsia | 1.0 | negative |
| Hyphomicrobium | Sphingomonas | 1.0 | positive |
| Faecalibacterium | Fusicatenibacter | 1.0 | positive |
| Faecalibacterium | Enterobacter | 1.0 | positive |
| Faecalibacterium | Erysipelotrichaceae\_UCG-003 | 1.0 | positive |
| Arenimonas | JGI\_0001001-H03 | 1.0 | positive |
| Arenimonas | Alishewanella | 1.0 | negative |
| Arenimonas | Reyranella | 1.0 | positive |
| Arenimonas | Persicitalea | 1.0 | positive |
| Arenimonas | Agathobacter | 1.0 | positive |
| Arenimonas | Hirschia | 1.0 | positive |
| Arenimonas | Sediminibacterium | 1.0 | positive |
| Arenimonas | Sphingosinicella | 1.0 | positive |
| Arenimonas | Streptococcus | 1.0 | positive |
| Arenimonas | Methyloversatilis | 1.0 | negative |
| Arenimonas | Longilinea | 1.0 | positive |
| Arenimonas | OLB13 | 1.0 | negative |
| Arenimonas | Lentimicrobium | 1.0 | positive |
| Arenimonas | Clostridium\_sensu\_stricto\_1 | 1.0 | positive |
| Arenimonas | Acidithiobacillus | 1.0 | positive |
| Arenimonas | [Eubacterium]\_coprostanoligenes\_group | 1.0 | positive |
| [Ruminococcus]\_torques\_group | Roseomonas | 1.0 | negative |
| [Ruminococcus]\_torques\_group | Anaerostipes | 1.0 | positive |
| [Ruminococcus]\_torques\_group | Rhodopseudomonas | 1.0 | negative |
| [Ruminococcus]\_torques\_group | Stella | 1.0 | negative |
| [Ruminococcus]\_torques\_group | Romboutsia | 1.0 | positive |
| [Ruminococcus]\_torques\_group | Sphingomonas | 1.0 | negative |
| JGI\_0001001-H03 | Alishewanella | 1.0 | negative |
| JGI\_0001001-H03 | Reyranella | 1.0 | positive |
| JGI\_0001001-H03 | Persicitalea | 1.0 | positive |
| JGI\_0001001-H03 | Agathobacter | 1.0 | positive |
| JGI\_0001001-H03 | Hirschia | 1.0 | positive |
| JGI\_0001001-H03 | Sediminibacterium | 1.0 | positive |
| JGI\_0001001-H03 | Sphingosinicella | 1.0 | positive |
| JGI\_0001001-H03 | Streptococcus | 1.0 | positive |
| JGI\_0001001-H03 | Methyloversatilis | 1.0 | negative |
| JGI\_0001001-H03 | Longilinea | 1.0 | positive |
| JGI\_0001001-H03 | OLB13 | 1.0 | negative |
| JGI\_0001001-H03 | Lentimicrobium | 1.0 | positive |
| JGI\_0001001-H03 | Clostridium\_sensu\_stricto\_1 | 1.0 | positive |
| JGI\_0001001-H03 | Acidithiobacillus | 1.0 | positive |
| JGI\_0001001-H03 | [Eubacterium]\_coprostanoligenes\_group | 1.0 | positive |
| Roseomonas | Anaerostipes | 1.0 | negative |
| Roseomonas | Rhodopseudomonas | 1.0 | positive |
| Roseomonas | Stella | 1.0 | positive |
| Roseomonas | Romboutsia | 1.0 | negative |
| Roseomonas | Sphingomonas | 1.0 | positive |
| Anaerostipes | Rhodopseudomonas | 1.0 | negative |
| Anaerostipes | Stella | 1.0 | negative |
| Anaerostipes | Romboutsia | 1.0 | positive |
| Anaerostipes | Sphingomonas | 1.0 | negative |
| Alishewanella | Reyranella | 1.0 | negative |
| Alishewanella | Persicitalea | 1.0 | negative |
| Alishewanella | Agathobacter | 1.0 | negative |
| Alishewanella | Hirschia | 1.0 | negative |
| Alishewanella | Sediminibacterium | 1.0 | negative |
| Alishewanella | Sphingosinicella | 1.0 | negative |
| Alishewanella | Streptococcus | 1.0 | negative |
| Alishewanella | Methyloversatilis | 1.0 | positive |
| Alishewanella | Longilinea | 1.0 | negative |
| Alishewanella | OLB13 | 1.0 | positive |
| Alishewanella | Lentimicrobium | 1.0 | negative |
| Alishewanella | Clostridium\_sensu\_stricto\_1 | 1.0 | negative |
| Alishewanella | Acidithiobacillus | 1.0 | negative |
| Alishewanella | [Eubacterium]\_coprostanoligenes\_group | 1.0 | negative |
| Reyranella | Persicitalea | 1.0 | positive |
| Reyranella | Agathobacter | 1.0 | positive |
| Reyranella | Hirschia | 1.0 | positive |
| Reyranella | Sediminibacterium | 1.0 | positive |
| Reyranella | Sphingosinicella | 1.0 | positive |
| Reyranella | Streptococcus | 1.0 | positive |
| Reyranella | Methyloversatilis | 1.0 | negative |
| Reyranella | Longilinea | 1.0 | positive |
| Reyranella | OLB13 | 1.0 | negative |
| Reyranella | Lentimicrobium | 1.0 | positive |
| Reyranella | Clostridium\_sensu\_stricto\_1 | 1.0 | positive |
| Reyranella | Acidithiobacillus | 1.0 | positive |
| Reyranella | [Eubacterium]\_coprostanoligenes\_group | 1.0 | positive |
| Rhodopseudomonas | Stella | 1.0 | positive |
| Rhodopseudomonas | Romboutsia | 1.0 | negative |
| Rhodopseudomonas | Sphingomonas | 1.0 | positive |
| Stella | Romboutsia | 1.0 | negative |
| Stella | Sphingomonas | 1.0 | positive |
| Persicitalea | Agathobacter | 1.0 | positive |
| Persicitalea | Hirschia | 1.0 | positive |
| Persicitalea | Sediminibacterium | 1.0 | positive |
| Persicitalea | Sphingosinicella | 1.0 | positive |
| Persicitalea | Streptococcus | 1.0 | positive |
| Persicitalea | Methyloversatilis | 1.0 | negative |
| Persicitalea | Longilinea | 1.0 | positive |
| Persicitalea | OLB13 | 1.0 | negative |
| Persicitalea | Lentimicrobium | 1.0 | positive |
| Persicitalea | Clostridium\_sensu\_stricto\_1 | 1.0 | positive |
| Persicitalea | Acidithiobacillus | 1.0 | positive |
| Persicitalea | [Eubacterium]\_coprostanoligenes\_group | 1.0 | positive |
| Agathobacter | Hirschia | 1.0 | positive |
| Agathobacter | Sediminibacterium | 1.0 | positive |
| Agathobacter | Sphingosinicella | 1.0 | positive |
| Agathobacter | Streptococcus | 1.0 | positive |
| Agathobacter | Methyloversatilis | 1.0 | negative |
| Agathobacter | Longilinea | 1.0 | positive |
| Agathobacter | OLB13 | 1.0 | negative |
| Agathobacter | Lentimicrobium | 1.0 | positive |
| Agathobacter | Clostridium\_sensu\_stricto\_1 | 1.0 | positive |
| Agathobacter | Acidithiobacillus | 1.0 | positive |
| Agathobacter | [Eubacterium]\_coprostanoligenes\_group | 1.0 | positive |
| Hirschia | Sediminibacterium | 1.0 | positive |
| Hirschia | Sphingosinicella | 1.0 | positive |
| Hirschia | Streptococcus | 1.0 | positive |
| Hirschia | Methyloversatilis | 1.0 | negative |
| Hirschia | Longilinea | 1.0 | positive |
| Hirschia | OLB13 | 1.0 | negative |
| Hirschia | Lentimicrobium | 1.0 | positive |
| Hirschia | Clostridium\_sensu\_stricto\_1 | 1.0 | positive |
| Hirschia | Acidithiobacillus | 1.0 | positive |
| Hirschia | [Eubacterium]\_coprostanoligenes\_group | 1.0 | positive |
| Fusicatenibacter | Enterobacter | 1.0 | positive |
| Fusicatenibacter | Erysipelotrichaceae\_UCG-003 | 1.0 | positive |
| Romboutsia | Sphingomonas | 1.0 | negative |
| Enterobacter | Erysipelotrichaceae\_UCG-003 | 1.0 | positive |
| Sediminibacterium | Sphingosinicella | 1.0 | positive |
| Sediminibacterium | Streptococcus | 1.0 | positive |
| Sediminibacterium | Methyloversatilis | 1.0 | negative |
| Sediminibacterium | Longilinea | 1.0 | positive |
| Sediminibacterium | OLB13 | 1.0 | negative |
| Sediminibacterium | Lentimicrobium | 1.0 | positive |
| Sediminibacterium | Clostridium\_sensu\_stricto\_1 | 1.0 | positive |
| Sediminibacterium | Acidithiobacillus | 1.0 | positive |
| Sediminibacterium | [Eubacterium]\_coprostanoligenes\_group | 1.0 | positive |
| Sphingosinicella | Streptococcus | 1.0 | positive |
| Sphingosinicella | Methyloversatilis | 1.0 | negative |
| Sphingosinicella | Longilinea | 1.0 | positive |
| Sphingosinicella | OLB13 | 1.0 | negative |
| Sphingosinicella | Lentimicrobium | 1.0 | positive |
| Sphingosinicella | Clostridium\_sensu\_stricto\_1 | 1.0 | positive |
| Sphingosinicella | Acidithiobacillus | 1.0 | positive |
| Sphingosinicella | [Eubacterium]\_coprostanoligenes\_group | 1.0 | positive |
| Streptococcus | Methyloversatilis | 1.0 | negative |
| Streptococcus | Longilinea | 1.0 | positive |
| Streptococcus | OLB13 | 1.0 | negative |
| Streptococcus | Lentimicrobium | 1.0 | positive |
| Streptococcus | Clostridium\_sensu\_stricto\_1 | 1.0 | positive |
| Streptococcus | Acidithiobacillus | 1.0 | positive |
| Streptococcus | [Eubacterium]\_coprostanoligenes\_group | 1.0 | positive |
| Methyloversatilis | Longilinea | 1.0 | negative |
| Methyloversatilis | OLB13 | 1.0 | positive |
| Methyloversatilis | Lentimicrobium | 1.0 | negative |
| Methyloversatilis | Clostridium\_sensu\_stricto\_1 | 1.0 | negative |
| Methyloversatilis | Acidithiobacillus | 1.0 | negative |
| Methyloversatilis | [Eubacterium]\_coprostanoligenes\_group | 1.0 | negative |
| Longilinea | OLB13 | 1.0 | negative |
| Longilinea | Lentimicrobium | 1.0 | positive |
| Longilinea | Clostridium\_sensu\_stricto\_1 | 1.0 | positive |
| Longilinea | Acidithiobacillus | 1.0 | positive |
| Longilinea | [Eubacterium]\_coprostanoligenes\_group | 1.0 | positive |
| OLB13 | Lentimicrobium | 1.0 | negative |
| OLB13 | Clostridium\_sensu\_stricto\_1 | 1.0 | negative |
| OLB13 | Acidithiobacillus | 1.0 | negative |
| OLB13 | [Eubacterium]\_coprostanoligenes\_group | 1.0 | negative |
| Lentimicrobium | Clostridium\_sensu\_stricto\_1 | 1.0 | positive |
| Lentimicrobium | Acidithiobacillus | 1.0 | positive |
| Lentimicrobium | [Eubacterium]\_coprostanoligenes\_group | 1.0 | positive |
| Clostridium\_sensu\_stricto\_1 | Acidithiobacillus | 1.0 | positive |
| Clostridium\_sensu\_stricto\_1 | [Eubacterium]\_coprostanoligenes\_group | 1.0 | positive |
| Acidithiobacillus | [Eubacterium]\_coprostanoligenes\_group | 1.0 | positive |

注：Source 为第一个节点名称；Target为第二个节点名称；weight为两个节点的相关性，值越大相关性越强；color为相关性类型，positive为正相关，negative为负相关。

网络中节点具有很多重要的性质，包括度、聚类系数、紧密中心性、中介中心性、Zi（within-module connectivity）与 Pi（among-module connectivity）[10]等。通常度、聚类系数、紧密中心性、中介中心性值越大，说明节点重要性越高；此外通过Zi和Pi值可以将节点分为四类：Peripheral nodes（zi ≤ 2.5, Pi ≤ 0.62，仅有少量的边并且通常只与模块内部的节点相连）、Connectors (zi ≤ 2.5, Pi > 0.62，通常连接不同的模块)、Module hubs (zi > 2.5, Pi ≤ 0.62，与自身所在模块中的许多节点高度连接)和Network hubs (zi > 2.5, Pi > 0.62，即能与自身所在模块中的许多节点高度连接又能连接不同的模块),通过这些性质可以说明网络中节点的重要性。节点各特性统计结果见下表：

表格7 节点属性表

| ID | Abudance | ModuleClass | Degree | Closeness Centrality | Betweenness Centrality | Custering Coefficient | Zi | Pi | NodeType |
| --- | --- | --- | --- | --- | --- | --- | --- | --- | --- |
| Acetobacter | 0.002 | Module1 | 22 | 0.2785 | 0.0 | 1.0 | 0.0 | 0.0 | Peripherals |
| Acidithiobacillus | 0.0007 | Module0 | 47 | 0.5949 | 0.0 | 1.0 | 0.0 | 0.0 | Peripherals |
| Acinetobacter | 0.0015 | Module0 | 47 | 0.5949 | 0.0 | 1.0 | 0.0 | 0.0 | Peripherals |
| Agathobacter | 0.001 | Module0 | 47 | 0.5949 | 0.0 | 1.0 | 0.0 | 0.0 | Peripherals |
| Akkermansia | 0.0027 | Module0 | 47 | 0.5949 | 0.0 | 1.0 | 0.0 | 0.0 | Peripherals |
| Algoriphagus | 0.009 | Module1 | 22 | 0.2785 | 0.0 | 1.0 | 0.0 | 0.0 | Peripherals |
| Alishewanella | 0.0012 | Module0 | 47 | 0.5949 | 0.0 | 1.0 | 0.0 | 0.0 | Peripherals |
| Anaerostipes | 0.0012 | Module1 | 22 | 0.2785 | 0.0 | 1.0 | 0.0 | 0.0 | Peripherals |
| Aquimonas | 0.0026 | Module2 | 8 | 0.1013 | 0.0 | 1.0 | 0.0 | 0.0 | Peripherals |
| Arenimonas | 0.0014 | Module0 | 47 | 0.5949 | 0.0 | 1.0 | 0.0 | 0.0 | Peripherals |
| Azoarcus | 0.0146 | Module0 | 47 | 0.5949 | 0.0 | 1.0 | 0.0 | 0.0 | Peripherals |
| Bacillus | 0.0041 | Module0 | 47 | 0.5949 | 0.0 | 1.0 | 0.0 | 0.0 | Peripherals |
| Bacteroidetes\_bacterium\_37-13 | 0.0028 | Module0 | 47 | 0.5949 | 0.0 | 1.0 | 0.0 | 0.0 | Peripherals |
| Bacteroidetes\_bacterium\_OLB9 | 0.0109 | Module0 | 47 | 0.5949 | 0.0 | 1.0 | 0.0 | 0.0 | Peripherals |
| Bdellovibrio | 0.002 | Module0 | 47 | 0.5949 | 0.0 | 1.0 | 0.0 | 0.0 | Peripherals |
| Bifidobacterium | 0.004 | Module2 | 8 | 0.1013 | 0.0 | 1.0 | 0.0 | 0.0 | Peripherals |
| Blautia | 0.0027 | Module2 | 8 | 0.1013 | 0.0 | 1.0 | 0.0 | 0.0 | Peripherals |
| Brevifollis | 0.0194 | Module1 | 22 | 0.2785 | 0.0 | 1.0 | 0.0 | 0.0 | Peripherals |
| Brevundimonas | 0.0059 | Module0 | 47 | 0.5949 | 0.0 | 1.0 | 0.0 | 0.0 | Peripherals |
| Bryobacter | 0.0025 | Module1 | 22 | 0.2785 | 0.0 | 1.0 | 0.0 | 0.0 | Peripherals |
| Candidatus\_Competibacter | 0.021 | Module1 | 22 | 0.2785 | 0.0 | 1.0 | 0.0 | 0.0 | Peripherals |
| Clostridium\_sensu\_stricto\_1 | 0.0007 | Module0 | 47 | 0.5949 | 0.0 | 1.0 | 0.0 | 0.0 | Peripherals |
| Dechloromonas | 0.0026 | Module2 | 8 | 0.1013 | 0.0 | 1.0 | 0.0 | 0.0 | Peripherals |
| Denitratisoma | 0.0053 | Module1 | 22 | 0.2785 | 0.0 | 1.0 | 0.0 | 0.0 | Peripherals |
| Ellin6067 | 0.0033 | Module0 | 47 | 0.5949 | 0.0 | 1.0 | 0.0 | 0.0 | Peripherals |
| Enterobacter | 0.0009 | Module2 | 8 | 0.1013 | 0.0 | 1.0 | 0.0 | 0.0 | Peripherals |
| Erysipelotrichaceae\_UCG-003 | 0.0007 | Module2 | 8 | 0.1013 | 0.0 | 1.0 | 0.0 | 0.0 | Peripherals |
| Escherichia-Shigella | 0.0029 | Module1 | 22 | 0.2785 | 0.0 | 1.0 | 0.0 | 0.0 | Peripherals |
| Faecalibacterium | 0.0014 | Module2 | 8 | 0.1013 | 0.0 | 1.0 | 0.0 | 0.0 | Peripherals |
| Faecalibaculum | 0.0017 | Module0 | 47 | 0.5949 | 0.0 | 1.0 | 0.0 | 0.0 | Peripherals |
| Flavobacterium | 0.0165 | Module1 | 22 | 0.2785 | 0.0 | 1.0 | 0.0 | 0.0 | Peripherals |
| Fusicatenibacter | 0.0009 | Module2 | 8 | 0.1013 | 0.0 | 1.0 | 0.0 | 0.0 | Peripherals |
| Gemmatimonas | 0.0037 | Module1 | 22 | 0.2785 | 0.0 | 1.0 | 0.0 | 0.0 | Peripherals |
| Gemmobacter | 0.002 | Module0 | 47 | 0.5949 | 0.0 | 1.0 | 0.0 | 0.0 | Peripherals |
| Hirschia | 0.0009 | Module0 | 47 | 0.5949 | 0.0 | 1.0 | 0.0 | 0.0 | Peripherals |
| Hydrogenophaga | 0.009 | Module0 | 47 | 0.5949 | 0.0 | 1.0 | 0.0 | 0.0 | Peripherals |
| Hyphomicrobium | 0.0015 | Module1 | 22 | 0.2785 | 0.0 | 1.0 | 0.0 | 0.0 | Peripherals |
| JGI\_0001001-H03 | 0.0013 | Module0 | 47 | 0.5949 | 0.0 | 1.0 | 0.0 | 0.0 | Peripherals |
| Lactobacillus | 0.0017 | Module0 | 47 | 0.5949 | 0.0 | 1.0 | 0.0 | 0.0 | Peripherals |
| Lentimicrobium | 0.0007 | Module0 | 47 | 0.5949 | 0.0 | 1.0 | 0.0 | 0.0 | Peripherals |
| Longilinea | 0.0007 | Module0 | 47 | 0.5949 | 0.0 | 1.0 | 0.0 | 0.0 | Peripherals |
| Mariniradius | 0.0021 | Module0 | 47 | 0.5949 | 0.0 | 1.0 | 0.0 | 0.0 | Peripherals |
| Methyloversatilis | 0.0008 | Module0 | 47 | 0.5949 | 0.0 | 1.0 | 0.0 | 0.0 | Peripherals |
| Nitrosomonas | 0.0114 | Module0 | 47 | 0.5949 | 0.0 | 1.0 | 0.0 | 0.0 | Peripherals |
| Nitrospira | 0.009 | Module0 | 47 | 0.5949 | 0.0 | 1.0 | 0.0 | 0.0 | Peripherals |
| Novosphingobium | 0.0017 | Module0 | 47 | 0.5949 | 0.0 | 1.0 | 0.0 | 0.0 | Peripherals |
| OLB12 | 0.0068 | Module1 | 22 | 0.2785 | 0.0 | 1.0 | 0.0 | 0.0 | Peripherals |
| OLB13 | 0.0007 | Module0 | 47 | 0.5949 | 0.0 | 1.0 | 0.0 | 0.0 | Peripherals |
| OLB17 | 0.1061 | Module0 | 47 | 0.5949 | 0.0 | 1.0 | 0.0 | 0.0 | Peripherals |
| OLB8 | 0.0469 | Module1 | 22 | 0.2785 | 0.0 | 1.0 | 0.0 | 0.0 | Peripherals |
| Paracoccus | 0.0025 | Module0 | 47 | 0.5949 | 0.0 | 1.0 | 0.0 | 0.0 | Peripherals |
| Persicitalea | 0.001 | Module0 | 47 | 0.5949 | 0.0 | 1.0 | 0.0 | 0.0 | Peripherals |
| Phreatobacter | 0.0021 | Module0 | 47 | 0.5949 | 0.0 | 1.0 | 0.0 | 0.0 | Peripherals |
| Plasticicumulans | 0.1053 | Module0 | 47 | 0.5949 | 0.0 | 1.0 | 0.0 | 0.0 | Peripherals |
| Prosthecobacter | 0.0063 | Module1 | 22 | 0.2785 | 0.0 | 1.0 | 0.0 | 0.0 | Peripherals |
| Pseudofulvimonas | 0.0033 | Module0 | 47 | 0.5949 | 0.0 | 1.0 | 0.0 | 0.0 | Peripherals |
| Pseudomonas | 0.0024 | Module1 | 22 | 0.2785 | 0.0 | 1.0 | 0.0 | 0.0 | Peripherals |
| Pseudoxanthomonas | 0.0042 | Module0 | 47 | 0.5949 | 0.0 | 1.0 | 0.0 | 0.0 | Peripherals |
| Reyranella | 0.0012 | Module0 | 47 | 0.5949 | 0.0 | 1.0 | 0.0 | 0.0 | Peripherals |
| Rhodobacter | 0.0064 | Module0 | 47 | 0.5949 | 0.0 | 1.0 | 0.0 | 0.0 | Peripherals |
| Rhodopseudomonas | 0.0011 | Module1 | 22 | 0.2785 | 0.0 | 1.0 | 0.0 | 0.0 | Peripherals |
| Romboutsia | 0.0009 | Module1 | 22 | 0.2785 | 0.0 | 1.0 | 0.0 | 0.0 | Peripherals |
| Roseomonas | 0.0012 | Module1 | 22 | 0.2785 | 0.0 | 1.0 | 0.0 | 0.0 | Peripherals |
| Runella | 0.0021 | Module1 | 22 | 0.2785 | 0.0 | 1.0 | 0.0 | 0.0 | Peripherals |
| SM1A02 | 0.0074 | Module0 | 47 | 0.5949 | 0.0 | 1.0 | 0.0 | 0.0 | Peripherals |
| SWB02 | 0.0085 | Module0 | 47 | 0.5949 | 0.0 | 1.0 | 0.0 | 0.0 | Peripherals |
| Sediminibacterium | 0.0008 | Module0 | 47 | 0.5949 | 0.0 | 1.0 | 0.0 | 0.0 | Peripherals |
| Sphingomonas | 0.0007 | Module1 | 22 | 0.2785 | 0.0 | 1.0 | 0.0 | 0.0 | Peripherals |
| Sphingopyxis | 0.0021 | Module0 | 47 | 0.5949 | 0.0 | 1.0 | 0.0 | 0.0 | Peripherals |
| Sphingorhabdus | 0.0026 | Module0 | 47 | 0.5949 | 0.0 | 1.0 | 0.0 | 0.0 | Peripherals |
| Sphingosinicella | 0.0008 | Module0 | 47 | 0.5949 | 0.0 | 1.0 | 0.0 | 0.0 | Peripherals |
| Stella | 0.0011 | Module1 | 22 | 0.2785 | 0.0 | 1.0 | 0.0 | 0.0 | Peripherals |
| Streptococcus | 0.0008 | Module0 | 47 | 0.5949 | 0.0 | 1.0 | 0.0 | 0.0 | Peripherals |
| Subdoligranulum | 0.0016 | Module2 | 8 | 0.1013 | 0.0 | 1.0 | 0.0 | 0.0 | Peripherals |
| Terrimonas | 0.0022 | Module0 | 47 | 0.5949 | 0.0 | 1.0 | 0.0 | 0.0 | Peripherals |
| Thauera | 0.0692 | Module0 | 47 | 0.5949 | 0.0 | 1.0 | 0.0 | 0.0 | Peripherals |
| Turneriella | 0.0021 | Module1 | 22 | 0.2785 | 0.0 | 1.0 | 0.0 | 0.0 | Peripherals |
| Zoogloea | 0.137 | Module0 | 47 | 0.5949 | 0.0 | 1.0 | 0.0 | 0.0 | Peripherals |
| [Eubacterium]\_coprostanoligenes\_group | 0.0006 | Module0 | 47 | 0.5949 | 0.0 | 1.0 | 0.0 | 0.0 | Peripherals |
| [Ruminococcus]\_torques\_group | 0.0013 | Module1 | 22 | 0.2785 | 0.0 | 1.0 | 0.0 | 0.0 | Peripherals |

注：ID 为节点名称；Abudance为节点平均丰度；ModuleClass为节点所在的模块类别；Degree为节点度；Closeness Centrality为紧密中心性；Betweenness Centrality为中介中心性；Custering Coefficient为聚类系数；Zi为模块内连通性；Pi为模块间连通性；NodeType为根据Zi和Pi值划分的类别。

图19 节点Zi-Pi分布图

注：横坐标为Pi值，纵坐标为Zi值，不同颜色的点表示不同类型的节点。

网络自身也具有很多不同的特性，例如节点数据量、边数量、模块性与模块数量、网络直径与密度、平均最短路径与平均聚类系数等，网络属性统计结果见下表：

表格8 网络属性表

| Network properties | Value |
| --- | --- |
| Number of nodes | 80 |
| Number of edges | 1,417 |
| Modularity | 0.334 |
| Number of communities | 3 |
| Network diameter | 1 |
| Network Density | 0.448 |
| Average shortest path length | 1.0 |
| Average clustering coefficient | 1.0 |

注：Number of nodes表示节点数量；Number of edges表示边数量；Modularity表示模块性；Number of communities表示模块数量；Network diameter表示网络直径；Network Density表示网络密度；Average shortest path length为平均最短路径；Average clustering coefficient为平均聚类系数。

基于python绘制物种相关性网络图。此处展示相关性最高的前50个属，如下：

图20 属水平各物种网络图

注：圆圈代表物种，圆圈大小代表物种平均丰度大小；线条代表两物种间相关，线的粗细代表相关性的强弱，线的颜色：橙色代表正相关，绿色代表负相关。

结果文件：Net\_work

#### 4.7 功能基因预测分析

##### 4.7.1 PICRUSt2功能预测

使用PICRUSt2软件采用将待预测的特征序列与软件中已有的系统发育树中进行物种注释，使用 IMG 微生物基因组数据进行功能信息的输出进而推测样本中的功能基因组成，从而分析不同样本或分组之间在功能上的差异[9]。对不同样本间的功能丰度使用STAMP中的G-TEST（大样本：注释到的功能基因数目大于20）和Fisher（小样本：注释到的功能基因数目小于20）检验方法进行两两样本间的显著性差异检验，对不同组间进行两两T-test检验，P-value阈值为0.05（<0.05表示显著）。

通过KEGG代谢途径的组成及差异分析，可以观测不同分组的样品之间微生物群落的功能基因在代谢途径上的差异和变化，是研究群落样本为适应环境变化发生的代谢功能改变的有效手段。

图21 KEGG代谢通路柱状图

注：横坐标为物种,纵坐标为代谢通路相对丰度百分比。

图22 组间KEGG代谢途径差异分析图

上图为第二层级下KEGG代谢途径的差异分析图(也可以针对第三或第一层的分级进行分析)：图中不同颜色代表不同的分组。注：图片中左图所示为不同功能在两个样品或者两组样品中的丰度比例，中间所示为95%置信度区间内功能丰度的差异比例，最右边的值为p值。

结果文件：KEGG

COG（Clusters of Orthologous Groups of proteins）即原核生物同源蛋白簇数据库，是原核生物常用的蛋白功能分类数据库。COG功能预测分析方法与KEGG基本相同，分析结果如下图所示，反应了样品中序列的功能分布及所占丰度。

图23 COG代谢通路柱状图

注：横坐标为物种,纵坐标为代谢通路相对丰度百分比。

图24 COG功能分类统计图

注：左图所示为不同功能在两个样品或者两组样品中的丰度比例，中间所示为95%置信度区间内功能丰度的差异比例，最右边的值为p值。

结果文件：COG

在获得标准报告后如果希望单独修改分组或对某些组之间进行显著性差异分析，可以使用 STAMP 软件自行进行数据分析。STAMP提供了丰富的统计检验方法和图形化结果的输出。在使用STAMP之前需要首先准备需要的spf格式文件和样品分组信息表。在我们的报告中已经将KEGG和COG的结果文件转换成了适用于STAMP软件打开的spf格式文件，并提供了对应的分组信息表文件groupfile.txt。

##### 4.7.2 BugBase表型预测

BugBase是一种预测复杂微生物组内功能途径的生物水平覆盖以及生物可解释表型的方法。BugBase首先通过预测的16S拷贝数对OTU进行归一化，然后使用提供的预先计算的文件预测微生物表型[11]。首先，针对生物学数据集中的每个样本，在覆盖阈值的整个范围（0到1，以0.01为增量）中估计性状相对丰度。然后，BugBase为用户数据中的每个特征选择所有样本中方差最高的覆盖率阈值。设置阈值后，BugBase会生成最终的生物体水平性状预测表，其中包含每个样本的预测性状相对丰度。根据用户指定的元数据，选择性地对差异性状进行自动假设测试并可视化，并生成描述具有特征性状类群相对丰度的类群贡献图, 输出包括非参数微分检验（Mann-Whitney U或Kruskal Wallis）统计摘要文件。

图25 BugBase表型预测图

注：横坐标为组名,纵坐标为相对丰度百分比,三条线自下而上分别为下四分位,平均值和上四分位。

图26 BugBase物种柱状图

注：横坐标为组名,纵坐标为物种相对丰度百分比。图片展示了科水平的9种表型,更多分类水平的柱状图在结果文件内查看

结果文件：BugBase

### 5 使用建议

微生物多样性生物信息分析部分是在Linux平台上完成的，数据结果或被压缩成zip、tar.gz、tar等格式，以下介绍了常见的操作方法：

1)文件解压：linux系统解压：zip包解压命令“unzip file.zip”，tar包解压命令“tar -xvf file.tar”，tar.gz包解压命令“tar -xzvf file.tar.gz”；windows系统可以使用WINRAR或7-zip解压。

2)查看文本文件：linux或unix用户可以用more、less等命令查看，Windows 用户，可用文本编辑器或写字板打开并编辑文本文件，比如gedit或editplus。

3)图片查看与编辑：数据中可能包含部分图像文件，一般图像文件后缀名为.png、.pdf、tiff、svg等，对于图像文件，Windows用户可以使用图片浏览器打开，Linux/Unix用户使用display命令打开；如果需要重新修改图片，则建议使用AI编辑器。具体操作：首先，在windows上安装Adobe Illustrator（Adobe Illustrator CS6）软件，双击打开软件；第二步，使用快捷键“Ctrl+o”或者选择“文件-打开”找到pdf文件打开PDF如果是单页的PDF，可以直接确定。多页PDF，选择需要修改的页面，在确定后，直接打开要修改的PDF页面，选择左侧工具栏进行编辑；第三步，保存。要存储回原始的多页PDF,直接使用“存储”命令即可，快捷键是Ctrl+S,如果想将当前编辑的页面存储为单页的PDF，可以直接选择“存储为”命令，快捷键是“Shift+Ctrl+S”。

4)表格打开方式：Linux下的表格均为制表符(Tab)分割的文本，可直接用less命令查看（less -SN \*.xls）也可使用excel或openoffice等办公软件打开。

(注意：当文件比较大时，打开文件可能导致Windows系统死机，建议使用性能较好的计算机或者使用更适合处理大量数据的Unix/Linux系统打开。)

### 6 附录

#### 6.1 分析方法

方法描述：Method.pdf

#### 6.2 结果文件列表

结果目录结构：customer\_backup介绍

样品信息：sample\_information.xls

测序数据质量评估：customer\_backup/data\_assement

OTU分析：customer\_backup/otus

物种注释及分类学分析：customer\_backup/taxa\_summary

Alpha多样性分析：customer\_backup/alpha\_diversity

Beta多样性分析：customer\_backup/beta\_diversity

相关性与关联分析：customer\_backup/association\_analysis

功能基因预测分析：customer\_backup/function\_analysis

#### 6.3 结果文件说明

文件说明：Result.pdf

### 参考文献

1. Edgar R C. UPARSE: highly accurate OTU sequences from microbial amplicon reads[J]. Nature methods, 2013, 10(10): 996.
2. Hanbo C, Paul CB. (2011). VennDiagram: a package for the generation of highly-customizable Venn and Euler diagrams in R. BMC Bioinformatics, 12 (1): 35.
3. Huson DH, Auch AF, Qi J, et al. (2007). MEGAN analysis of metagenomic data. Genome research, 17(3): 377-386.
4. Grice EA, Kong HH, et al. (2009). Topographical and temporal diversity of the human skin microbiome. Science, 324(5931): 1190–1192.
5. Wang Y, Sheng H-F, He Y, Wu J-Y, Jiang Y-X, Tam NF-Y, Zhou H-W: Comparison of the levels of bacterial diversity in freshwater, intertidal wetland, and marine sediments by using millions of illumina tags. Applied and environmental microbiology 2012, 78(23):8264-8271.
6. Kõljalg U, Nilsson RH, Abarenkov K, Tedersoo L, Taylor AF, Bahram M, Bates ST, Bruns TD, Bengtsson‐Palme J, Callaghan TM: Towards a unified paradigm for sequence‐based identification of fungi. Molecular Ecology 2013, 22(21):5271-5277.
7. Lozupone C, Knight R. (2005). UniFrac: a new phylogenetic method for comparing microbial communities. Appl Environ Microbiol, 71 (12): 8228-8235.
8. Gower J C . Some Distance Properties of Latent Root and Vector Methods Used in Multivariate Analysis[J]. Biometrika, 1966, 53(3-4):325-338.
9. Donovan H. Parks1, Gene W. Tyson, STAMP: statistical analysis of taxonomic and functional profiles, Bioinformatics (2014) 30(21): 3123-3124: 10.1093
10. Deng et al. (2012) Molecular ecological network analyses. BMC Bioinformatics 13:113.
11. Ward T, Larson J, Meulemans J, Hillmann B, Lynch J, Sidiropoulos D,Spear J, Caporaso G, Blekhman R, Knight R, Fink R, Knights D. 2017.BugBase predicts organism level microbiome phenotypes. bioRxiv

- 摘要
- 1 背景介绍
- 2 项目概况
  - 2.1 结果概述
  - 2.2 样品基本信息
- 3 工作流程
  - 3.1 工作流程图
  - 3.2 实验流程
  - 3.3 信息分析流程
- 4 分析结果
  - 4.1 测序数据质量评估
  - 4.2 OTU/ASV分析
  - 4.3 物种注释及分类学分析
    - 4.3.1 聚类结果展示及说明
    - 4.3.2 物种分布柱状(饼)图
    - 4.3.3 物种丰度聚类热图
    - 4.3.4 系统进化树
    - 4.3.5 MEGAN分类学树状图
    - 4.3.6 KRONA物种注释
  - 4.4 Alpha多样性分析
    - 4.4.1 Alpha多样性指数统计
    - 4.4.2 稀释性曲线(Rarefaction Curve)
    - 4.4.3 香农指数曲线(Shannon Index)
    - 4.4.4 等级丰度曲线(Rank Abundance Curve)
  - 4.5 Beta多样性分析
    - 4.5.1 PCoA分析
    - 4.5.2 UPGMA分析
    - 4.5.3 UPGMA聚类树与柱状图结合绘图
    - 4.5.4 样品热图分析
  - 4.6 相关性与关联分析
    - 4.6.1 相关性网络分析
  - 4.7 功能基因预测分析
    - 4.7.1 PICRUSt2功能预测
    - 4.7.2 BugBase表型预测
- 5 使用建议
- 6 附录
  - 6.1 分析方法
  - 6.2 结果文件列表
  - 6.3 结果文件说明
- 参考文献

Copyright © 2009-2015 北京百迈客生物科技有限公司版权所有 京ICP备10042835号

公司地址：北京市顺义区南法信府前街12号顺捷大厦5层

- Tel:400-600-3186
- Fax:010-57045001
- Tel:400-600-3186
- E-mail:tech@biomarker.com.cn
- 微信:biomarker\_tech
- 百迈客生物云平台
- 关于我们
