# Supplementary material for: Effect of tetracycline on nitrogen removal in Moving Bed Biofilm Reactor (MBBR) System
Source: PLoS One. 2022 Jan 10;17(1):e0261306. doi: 10.1371/journal.pone.0261306 (PMC8746769; doi:10.1371/journal.pone.0261306)
Supplement: S2 Data — (ZIP) [file pone.0261306.s002.zip › src/Method.pdf]

微生物多样性分析方法介绍 (v2.6)

北京百迈客生物科技有限公司

2020 年 10 月 12 日

目录

1 分析方法........1
1.1 测序数据质量评估........1
1.2 OTU / ASV 分析........3
1.3 物种注释及分类学分析........4
1.3.1 数据库选择........4
1.3.2 物种注释方法........4
1.4 多样性分析........5
1.4.1 Alpha 指数分析........5
1.4.2 Beta 多样性分析（n ≥ 3）........6
1.5 组间差异显著性分析........9
1.5.1 LefSe 分析........9
1.5.2 Metastats 分析........10
1.6 相关性与关联分析........10
1.6.1 RDA/CCA 分析........10
1.6.2 相关性网络分析........10
1.7 功能预测分析........11
1.7.1 picrust2 功能预测........11
1.7.2 BugBase 表型预测........12
1.7.3 真菌表型预测........13
【参考文献】........14

1 分析方法

1.1 测序数据质量评估

二代数据处理流程：首先使用 Trimmomatic[1] (version 0.33)对原始数据进行质量过滤，然后使用 Cutadapt[2]（version 1.9.1）进行引物序列的识别与去除，其后使用 FLASH [3]（version 1.2.11）对双端 reads 进行拼接并去除嵌合体（UCHIME [4] , version 8.1）,最终得到高质量的序列用于后续分析。具体参数如下：

1） Trimmomatic 原理：Trimmomatic 是一个针对 Illumina 高通量测序的 reads 质控过滤工具。它能对碱基质量格式为 phred 33 或 phred 64（取决于 Illumina 测序的机器）的 FASTQ 格式双端测序或单端测序数据进行过滤。单端测序文件需要一个输入文件和一个输出文件名称，加上参数；双端测序数据需要两个输入文件（forward 和 reverse 的 fastq 数据）。参数为设置：50bp 的窗口，如果窗口内的平均质量值低于 20，从窗口开始截去后端碱基。

2） 引物序列识别与去除：使用 Cutadapt 软件，按照允许最大错配率 20%，最小覆盖度 80%的参数进行引物序列的识别。

3） PE reads 拼接：使用 FLASH v1.2.11 软件，按照最小 overlap 长度为 10bp，overlap 区允许的最大错配比率为 0.2（Default），对每个样品的 reads 进行拼接。

4） UCHIME 原理如下图：第一步，将 query sequence 拆分成不存在 overlap 的 chunks，然后比对数据库；第二步，选取每个 chunk 在数

据库中最好的 match，最终选择两条最好的 parents 序列；第三步，将待检测序列 query 与两条 parents 序列进行比对，如果两条 parents 分别有一段序列与 query 序列相似度在 80%以上,则判断该 query 为嵌合体。

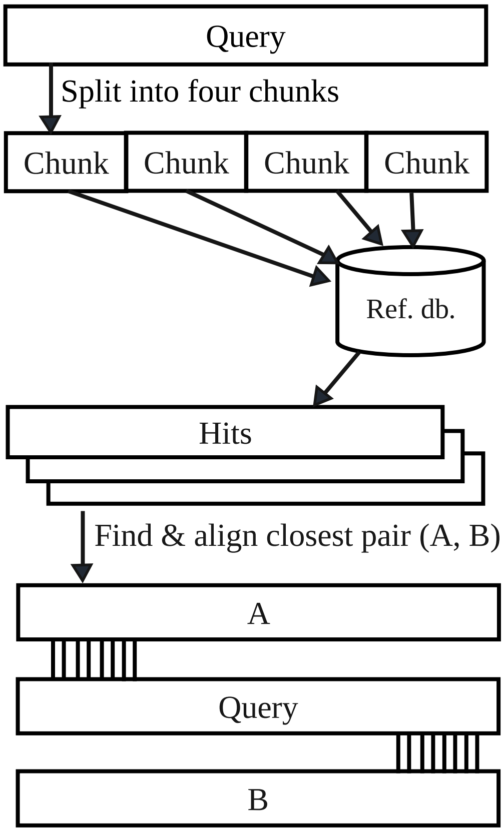

三代数据处理流程：原始下机 subreads 进行校正得到 CCS（Circular Consensus Sequencing）序列（SMRT Link，version8.0），然后使用 lima (v1.7.0)软件，通过 barcode 序列识别不同样品的 CCS 序列并去除嵌合体,得到高质量的 CCS 序列。具体参数如下：

1） CCS 序列获取：在全长多样性测序中，由于测序片段的大小远远小于测序长度，因此目标片段会重复测多次（passes）, CCS 通过对来自单个 ZMW 中的 subreads 互相校正而产生，通常当 passes 数目大于 4 时，CCS

的准确性就可以达到 99%以上。使用 SMRT Link v8.0 软件，按照 minPasses ≥5，minPredictedAccuracy≥0.9 识别 CCS 序列。

2） barcode 识别与引物识别：然后使用 lima v1.7.0 软件根据默认参数识别不同样品，然后使用 cutadapt v2.7（错误率 20%）识别正向引物与反向引物，丢弃掉不包含引物的 CCS 序列，最后对 CCS 长度进行过滤，丢弃不满足长度阈值的序列（16S：1200bp-1650bp；18S：1200bp-2000bp；ITS：300bp-1000bp）。

3） 去除嵌合体：去除嵌合体方法同上（二代数据处理流程）。

1.2 OTU / ASV 分析

OTU 聚类：使用 USEARCH[5]（version 10.0）在相似性 97% （默认）的水平上对序列进行聚类,默认以测序所有序列数的 0.005%作为阈值过滤 OTUs[6]。

ASV 分析：使用 QIIME2[7]（versoin 2020.6）中 DADA2[8]方法对质控后的数据进行去噪处理，默认以测序所有序列数的 0.005%作为阈值过滤 ASVs。

1.3 物种注释及分类学分析

1.3.1 数据库选择

Silva[9]（Release132, http://www.arb-silva.de）

Unite[10]（Release 8.0, https://unite.ut.ee/）

Greengenes[11]（version 13.5, http://greengenes.secondgenome.com/）

NCBI（ftp://ftp.ncbi.nlm.nih.gov/refseq/TargetedLoci/）

fungene[12]（http://fungene.cme.msu.edu/）

MaarjAM[13] (http://www.maarjam.botany.ut.ee)

1.3.2 物种注释方法

物种注释方法：

1) 基于比对方方法：QIIME2 中 classify-consensus-blast 是基于比对的方法，可以在 N（默认 3 个）个最好的比对结果中找一致最高的用于分类，最小序列相似性 90%，最小覆盖度 90%，最小一致性 51%。

2) 基于朴素贝斯分类器：通过 QIIME2 中 classify-sklearn 实现，使用前必须训练分类器，以便让软件“学会”哪些特征可以最好地区分每个分类组，分类器置信度（confidence）：0.7。

3) 比对结合分类器方法：先使用 classify-consensus-blast 将特性序列与参考数据库比对，不能精确比对上参考数据库的使用 classify-sklearn 分类器分类。

1.4 多样性分析

1.4.1 Alpha 指数分析

Alpha 多样性是指一个特定区域或者生态系统内的多样性，常用的度量菌群丰的指标有 Chao 丰富度估计量（Chao1 richness estimator）、Ace 丰富度估计量（Ace richness estimator）；度量菌群多样性的指标有：香农-威纳多样性指数（Shannon-wiener diversity index）、辛普森多样性指数（Simpson diversity index）、系统发育多样性指数（Phylogenetic diversity），。

Alpha 指数分析软件：QIIME2（https://qiime2.org/）。

1.4.1.1 稀释性曲线

稀释性曲线（Rarefaction Curve），稀释曲线是用来评价测序量是否足以覆盖所有类群，并间接反映样品中物种的丰富程度。稀释曲线是用已测得的 rDNA 序列中已知的各 OTU 的相对比例计算抽取 n 个（n 小于测得 reads 序列总数）reads 时出现 OTU 数量的期望值，然后根据一组 n 值（一般为一组小于总序列数的等差数列）与其相对应的 OTU 数量的期望值做出曲线来。当曲线趋于平缓或者达到平台期时也就可以认为测序深度已经基本覆盖到样品中所有的物种；反之，则表示样品中物种多样性较高，还存在较多未被测序检测到的物种。

1.4.1.2 Shannon 多样性曲线

Shannon 多样性曲线是以 shannon 指数来估算群落多样性的高低。利用各样品的测序量在不同测序深度时的微生物多样性指数构建曲线，反映各样本在不同测序数量时的微生物多样性。当曲线趋向平坦时，说明测序

数据量足够大，可以反映样品中绝大多数的微生物物种信息。

1.4.1.3 等级丰度曲线

等级丰度曲线（Rank Abundance Curve）用于同时解释样品所含物种的丰富度和均匀度。物种的丰富程度由曲线在横轴上的长度来反映，曲线越宽，表示物种的组成越丰富；物种组成的均匀程度由曲线的形状来反映，曲线越平坦，表示物种组成的均匀程度越高。

1.4.2 Beta 多样性分析（n ≥ 3）

Beta 多样性分析用于分析时间、空间尺度上的物种组成变化。方法：基于 binary jaccard、bray curtis、(un)weighted unifrac（限细菌）多种算法呈现物种多样性矩阵。基于 R 语言平台绘制样本主成分分析（PCA）、主坐标分析（PCoA）以及环境因子与样本组成相关性分析（RDA/CCA）。

PCA，PcoA，NMDS 分析都属于排序分析（Ordination analysis）。排序的过程就是在一个可视化的低维空间或平面重新排列这些样本，使得样本之间的距离最大程度地反映出平面散点图内样本之间的关系信息。

1.4.2.1 PCA 分析

主成分分析 PCA（ Principal component analysis）是通过一系列的特征值和特征向量进行排序，选择主要的前几位特征值，采取降维的思想，PCA 可以找到距离矩阵中最主要的坐标，从而观察个体或群体间的差异。

1.4.2.2 PcoA 分析

主坐标分析（principal coordinat analysis）是对特征值和特征向量进行排序，选择主要排在前几位的特征值，PCoA 可以找到距离矩阵中最主要

的坐标。通过 PCoA 可以观察个体或群体间的差异。PCoA 有多张图，分别代表的 PCoA1-2，PCoA2-3，PCoA3-1。

PCA 与 PcoA 分析的区别：PCA 分析是基于原始的物种组成矩阵所做的排序分析，PCoA 分析是基于由物种组成计算得到的距离矩阵进行分析。在 PCoA 分析中，计算距离矩阵的方法有多种，其中 weighted/unweighted Unifrac 是利用各样品序列间的进化信息来计算样品间距离，其中 weighted 考虑物种的丰度，unweighted 没有对物种丰度进行加权处理。

1.4.2.3 NMDS 分析

非度量多维尺度法，即 NMDS（Nonmetric Multidimensional Scaling）。是一种将多维空间的研究对象（样本或变量）简化到低维空间进行定位、分析和归类，同时又保留对象间原始关系的数据分析方法。适用于无法获得研究对象间精确的相似性或相异性数据，仅能得到他们之间等级关系数据的情形。其基本特征是将对象间的相似性或相异性数据看成点间距离的单调函数，在保持原始数据次序关系的基础上，用新的相同次序的数据列替换原始数据进行度量型多维尺度分析。

NMDS 与 PCA 分析的主要差异在于考量了进化方面的信息。

1.4.2.4 UPGMA 分析

非加权组平均法（UPGMA，unweighted pair-group method with arithmetic means）是一种较常用的聚类分析方法。其算法原理是：假设在进化过程中，每一世系发生趋异的次数相同，即核苷酸（或氨基酸）的替

换速率是均等且恒定的。首先将距离最小的 2 个 OTU 聚在一起，并形成一个新的 OTU，其分支点位于 2 个 OTU 间距离的 1/2 处；然后计算新的 OTU 与其它 OTU 间的平均距离，再找出其中的最小 2 个 OTU 进行聚类；如此反复，直到所有的 OTU 都聚到一起，最终得到一个完整的系统发生树。

1.4.2.5 样品热图分析

根据 OUT 数据进行标准化处理（取对数）之后，选取数目最多的前 80 个物种，基于 R heatmap 进行作图，热图中的每一个色块代表一个样品的一个属的丰度，样品横向排列，物种纵向排列，从聚类中可以了解样品之间的相似性以及各分类水平上的群落构成相似性。

1.4.2.6 Anosim 和 Adonis 分析

相似性分析(ANOSIM)是一种非参数检验，用来检验组间（两组或多组）的差异是否显著大于组内差异，从而判断分组是否有意义。Anosim 组间相似性分析的统计量 R 值取值范围为[-1,1]，R 值越接近 1 表示组间差异越大于组内差异，R 值越接近-1 表示组内差异越大于组间差异，R 值接近 0 表示组间和组内没有明显差异。

Adonis 又称置换多因素方差分析（permutational MANOVA）距离矩阵(如 Euclidean)对总方差进行分解，分析不同分组因素对样品差异的解释度，并使用置换检验对划分的统计学意义进行显著性分析。R2 取值范围[0,1]，越接近 1 说明分组因素对样品差异的解释度就越高。

1.5 组间差异显著性分析

1.5.1 LefSe 分析

LefSe[14]分析（http://huttenhower.sph.harvard.edu/lefse/），即组间差异显著物种分析（可以称作生物标记物（ biomarkers）分析），采用线性判别分析（LDA）来估算每个组分（物种）丰度对差异效果影响的大小，该分析主要是想找到组间在丰度上有显著差异的物种。

LDA 值：使用这种方法能够使投影后模式样本的类间散布矩阵最大，并且同时类内散布矩阵最小。就是说，它能够保证投影后模式样本在新的空间中有最小的类内距离和最大的类间距离，即模式在该空间中有最佳的可分离性。

设定显著差异的 logarithmic LDA score 为 4.0；若分组数> 2，LEfSe 分析寻找在所有组间具有显著性差异的物种。

PCA 和 LDA 的差别在于，PCA 只是将整组数据整体映射到最方便表示这组数据的坐标轴上，映射时没有利用任何数据内部的分类信息，是无监督的；LDA 增加了种属之间的信息关系后，结合显著性差异标准测试（克鲁斯卡尔-沃利斯检验和两两 Wilcoxon 测试）和线性判别分析的方法进行特征选择，是有监督的。除了可以检测重要特征，LefSe 分析还可以根据效应值进行功能特性排序，这些功能特性可以解释顶部的大部分生物学差异。

注：只对分组进行 LefSe 分析，分组数目 ≥ 2，且各分组所含的样品

数目 ≥2。

1.5.2 Metastats 分析

Metastats[15]（http://metastats.cbcb.umd.edu/）。

对组间的物种丰度数据进行 T 检验得到 p 值,并对 p 值进行校正得到 q 值；最后根据 p 值（或 q 值）筛选出导致两组样品组成差异的物种，默认 P≥0.05。

1.6 相关性与关联分析

1.6.1 RDA/CCA 分析

RDA(Redundancy analysis)/CCA(Canonical Correspondence analysis)是基于对应分析发展的一种排序方法，RDA 分析基于线性模型，CCA 分析基于单峰模型，主要用来反映菌群或样品与环境因子之间的关系。根据物种分布变化，选择最佳的分析模型。RDA 或 CCA 模型的选择原则：先用 species-sample 丰度数据做 DCA 分析，看分析结果中 Lengths of gradient 的第一轴的大小，如果大于 4.0，就应该选 CCA，如果 3.0-4.0 之间，选 RDA 和 CCA 均可，如果小于 3.0，RDA 的结果要好于 CCA。使用 R 语言 vegan（v2.3）包中 rda 或者 cca 分析和作图。

1.6.2 相关性网络分析

根据各个物种在各个样品中的丰度以及变化情况，进行斯皮尔曼(Spearman，默认方法)秩相关分析并筛选相关性大于 0.1 且 p 值小于 0.05 的数据构建相关性网络。网络分析常见度量如下：

度（Degree）:与节点相连的边的数量；

聚类系数（Custering Coefficient）：用于定义图中的“聚集性”（clustering）概念，反映了网络的凝聚性特征；

紧密中心性（Closeness Centrality）：反映在网络中某一节点与其他节点之间的接近程度；

中介中心性（Betweenness Centrality）：以经过某个节点的最短路径数目来刻画节点重要性的指标；

模块度（modularity）：是社团发现中用来衡量社团划分质量的一种方法，一个相对好的结果在社团（community）内部的节点连接度较高，而在社团外部节点的连接度较低；

网络直径（diameter）：网络图中最长的距离的值被称为图的“直径”，网络直径可反映网络的规模；

密度（Density）：是指实际出现的边与可能的边的频数之比，反映了网络的凝聚性特征；

平均路径长度（Average shortest path length）：网络中所有节点对之间的平均最短距离。

1.7 功能预测分析

1.7.1 picrust2 功能预测

PICRUSt2[16]一种利用标记基因数据和一个参考基因组数据库来预测环境微生物功能组成的计算方法，基于 IMG 微生物基因组数据通过系统

发育与功能的相关性预测微生物群落在系统发育过程中的功能潜力。其工作原理如图：

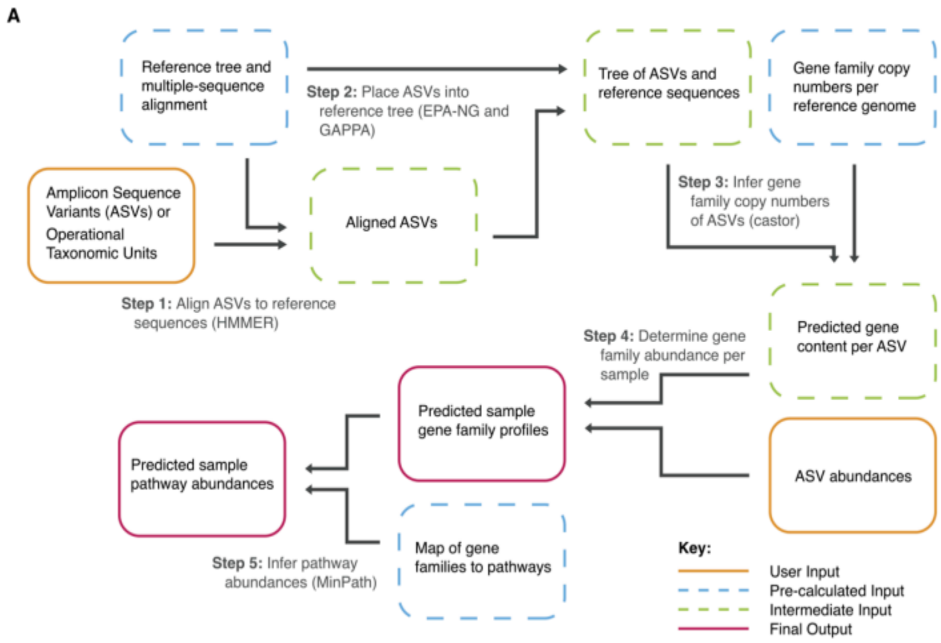

1.7.2 BugBase 表型预测

BugBase[17]是一种预测复杂微生物组内功能途径的生物水平覆盖以及生物可解释表型的方法。BugBase 首先通过预测的 16S 拷贝数对 OTU 进行归一化，然后使用提供的预先计算的文件预测微生物表型。首先，针对生物学数据集中的每个样本，在覆盖阈值的整个范围（0 到 1，以 0.01 为增量）中估计性状相对丰度。然后，BugBase 为用户数据中的每个特征选择所有样本中方差最高的覆盖率阈值。设置阈值后，BugBase 会生成最终的生物体水平性状预测表，其中包含每个样本的预测性状相对丰度。

预测表型类型：革兰氏阳性（Gram Positive）、革兰氏阴性（Gram Negative）、生物膜形成（Biofilm Forming）致病性（Pathogenic）、移动元件（Mobile Element Containing）、氧需求（Oxygen Utilizing）、氧化胁

迫耐受（Oxidative Stress Tolerant）。

1.7.3 真菌表型预测

FUNGuild[18](Fungi Functional Guild)是一种可用于由生态协会分类学解析真菌的工具，用简单而一致的方法将大型序列库分类为具有生态意义的类别。根据营养方式将真菌分为 12 类，然后构建了一个真菌分类和功能分组（guild）之间的数据库，通过这个数据库你就可以对真菌进行功能分类。

病理营养型（pathotroph）：通过损害宿主细胞而获取营养（包括吞噬型真菌 phagotrophs）。

共生营养型（symbiotroph）：通过与宿主细胞交换资源来获取营养。

腐生营养型（saprotroph）：通过降解死亡的宿主细胞来获取营养。包括动物病原菌（animal pathogens）、丛枝菌根真菌（arbuscular mycorrhizal fungi）、外生菌根真菌（ectomycorrhizal fungi）、杜鹃花类菌根真菌（ericoid mycorrhizal fungi）、叶内生真菌（foliar endophytes）、地衣寄生真菌（lichenicolous fungi）、地衣共生真菌（lichenized fungi）、菌寄生真菌（mycoparasites）、植物病原菌（plantpathogens）、未定义根内生真菌（undefined root endophytes）、未定义腐生真菌（undefined saprotrophs）和木质腐生真菌（wood saprotrophs）。

以上分析内容由 BMKCloud（www.biocloud.net）完成

注：若合同中未签某种分析或样品不适合做某种分析，则结题报告及结果文件中不会提供相应的结果。

【参考文献】

1. Bolger AM, Lohse M, Usadel B: Trimmomatic: a flexible trimmer for Illumina sequence data. Bioinformatics 2014:btu170. Bolger AM, Lohse M, Usadel B: Trimmomatic: a flexible trimmer for Illumina sequence data. Bioinformatics 2014:btu170.
2. Martin M.MartinM.Cut adapt removes adapter sequences from high-throughput sequencing reads, EMBnet. EMBnet 17:10-12[J]. Embnet Journal, 2011, 17(1).
3. Magoč T, Salzberg SL: FLASH: fast length adjustment of short reads to improve genome assemblies. Bioinformatics 2011, 27(21):2957-2963.
4. Edgar RC, Haas BJ, Clemente JC, Quince C, Knight R: UCHIME improves sensitivity and speed of chimera detection. Bioinformatics 2011, 27(16):2194-2200.
5. Edgar Robert C. UPARSE: highly accurate OTU sequences from microbial amplicon reads.[J] .Nat. Methods, 2013, 10(10): 996-8.
6. Bokulich NA, Subramanian S, Faith JJ, Gevers D, Gordon JI, Knight R, Mills DA, Caporaso JG: Quality-filtering vastly improves diversity estimates from Illumina amplicon sequencing. Nature methods 2013, 10(1):57-59.
7. Bolyen E, Rideout JR, Dillon MR, Bokulich NA, Abnet CC, Al-Ghalith GA, Alexander H, Alm EJ, Arumugam M, Asnicar F, Bai Y, Bisanz JE,

Bittinger K, Brejnrod A, Brislawn CJ, Brown CT, Callahan BJ, Caraballo-Rodríguez AM, Chase J, Cope EK, Da Silva R, Diener C, Dorrestein PC, Douglas GM, Durall DM, Duvallet C, Edwardson CF, Ernst M, Estaki M, Fouquier J, Gauglitz JM, Gibbons SM, Gibson DL, Gonzalez A, Gorlick K, Guo J, Hillmann B, Holmes S, Holste H, Huttenhower C, Huttley GA, Janssen S, Jarmusch AK, Jiang L, Kaehler BD, Kang KB, Keefe CR, Keim P, Kelley ST, Knights D, Koester I, Kosciolek T, Kreps J, Langille MGI, Lee J, Ley R, Liu YX, Loftfield E, Lozupone C, Maher M, Marotz C, Martin BD, McDonald D, McIver LJ, Melnik AV, Metcalf JL, Morgan SC, Morton JT, Naimey AT, Navas-Molina JA, Nothias LF, Orchanian SB, Pearson T, Peoples SL, Petras D, Preuss ML, Pruesse E, Rasmussen LB, Rivers A, Robeson MS, Rosenthal P, Segata N, Shaffer M, Shiffer A, Sinha R, Song SJ, Spear JR, Swafford AD, Thompson LR, Torres PJ, Trinh P, Tripathi A, Turnbaugh PJ, Ul-Hasan S, van der Hooft JJJ, Vargas F, Vázquez-Baeza Y, Vogtmann E, von Hippel M, Walters W, Wan Y, Wang M, Warren J, Weber KC, Williamson CHD, Willis AD, Xu ZZ, Zaneveld JR, Zhang Y, Zhu Q, Knight R, and Caporaso JG. 2019. Reproducible, interactive, scalable and extensible microbiome data science using QIIME 2. Nature Biotechnology 37: 852–857.

8. Callahan, B., McMurdie, P., Rosen, M. et al. DADA2: High-resolution

sample inference from Illumina amplicon data. Nat Methods 13, 581–583 (2016). https://doi.org/10.1038/nmeth.3869.

9. Callahan, B., McMurdie, P., Rosen, M. et al. DADA2: High-resolution sample inference from Illumina amplicon data. Nat Methods 13, 581–583 (2016). https://doi.org/10.1038/nmeth.3869.

10. Kõljalg U, Larsson KH, Abarenkov K, Nilsson RH, Alexander IJ, Eberhardt U, Erland S, Høiland K, Kjøller R, Larsson E, Pennanen T, Sen R, Taylor AF, Tedersoo L, Vrålstad T, Ursing BM.UNITE: a database providing web-based methods for the molecular identification of ectomycorrhizal fungi. [PMID: 15869663].

11. DeSantis T Z, Hugenholtz P, Larsen N, et al. Greengenes, a chimera-checked 16SrRNA gene database and workbench compatible with ARB[J]. Applied andenvironmental microbiology, 2006, 72(7): 5069-5072.

12. Fish, J. A., B. Chai, Q. Wang, Y. Sun, C. T. Brown, J. M. Tiedje, and J. R. Cole. 2013. FunGene: the Functional Gene Pipeline and Repository. Front. Microbiol. 4:291; doi: 10.3389/fmicb.2013.00291.

13. Öpik, M., Vanatoa, A., Vanatoa, E., Moora, M., Davison, J., Kalwij, J.M., Reier, Ü., Zobel, M. 2010. The online database MaarjAM reveals global and ecosystemic distribution patterns in arbuscular mycorrhizal fungi (Glomeromycota). New Phytologist 188: 223-241.

14. Segata N, Izard J, Waldron L, Gevers D, Miropolsky L, Garrett WS, Huttenhower C: Metagenomic biomarker discovery and explanation. Genome Biol 2011, 12(6):R60.

15. White J R, Nagarajan N, Pop M. Statistical methods for detecting differentially abundant features in clinical metagenomic samples[J]. PLoSComput Biol, 2009, 5(4): e1000352.

16. Douglas G M, Maffei V J, Zaneveld J, et al. PICRUSt2: An improved and extensible approach for metagenome inference. bioRxiv, 2019.

17. Ward T, Larson J, Meulemans J, Hillmann B, Lynch J, SidiropoulosD,Spear J, Caporaso G, Blekhman R, Knight R, Fink R, Knights D. 2017.BugBase predicts organism level microbiome phenotypes. bioRxiv.

18. Nguyen NH, Song Z, Bates ST, Branco, S, Tedersoo L, Menke J, Schilling JS, Kennedy PG. 2016. FUNGuild: an open annotation tool for parsing fungal community datasets by ecological guild. Fungal Ecology 20:241-248.
