# Supplementary material for: Effect of tetracycline on nitrogen removal in Moving Bed Biofilm Reactor (MBBR) System
Source: PLoS One. 2022 Jan 10;17(1):e0261306. doi: 10.1371/journal.pone.0261306 (PMC8746769; doi:10.1371/journal.pone.0261306)
Supplement: S2 Data — (ZIP) [file pone.0261306.s002.zip › src/Result.pdf]

微生物多样性结果文件说明
(v2.6)

北京百迈客生物科技有限公司

2020 年 10 月 14 日

目录

1 结果目录......1
1.1 测序数据质量评估：./data_assement/......1
1.2 OTU 分析：./otus/......1
1.3 物种注释及分类学分析： ./taxa_summary/......1
1.3.1 物种分布分析： ./taxa_summary/Taxa_dis/*/......1
1.3.2 物种丰度聚类分析：./taxa_summary/heatmap/*/......1
1.3.3 MEGAN 分类学分析：./taxa_summary/taxa_tree/和 krona/......1
1.3.4 系统进化树：./taxa_summary/Phylogenetic_tree/*/......2
1.4 Alpha 多样性分析：./alpha_div/......2
1.4.1 Alpha 多样性指数统计：./alpha_div/alpha_index_stat/*/......2
1.4.2 稀释性曲线：./alpha_div/rarefaction_curve/*/......2
1.4.3 Shannon 曲线：./alpha_div/shannon_index_curve/*/......2
1.4.4 等级丰度曲线：./alpha_div/rank_abund_curve/*/......2
1.4.5 物种累计曲线：./alpha_div/specaccum/*/......3
1.5 Beta 多样性分析：./Beta_diversity/......3
1.5.1 PCA 分析：./Beta_diversity/pca/*/......3
1.5.2 PCoA 分析：./Beta_diversity/pcoa/*/......3
1.5.3 NMDS 分析：./Beta_diversity/nmds/*/......3
1.5.4 层次聚类分析：./Beta_diversity/upgma_tree/*/......3
1.5.5 层次聚类与柱状图分析：./Beta_diversity/ClusterTree_bar/*/......4
1.5.6 样品热图分析：./Beta_diversity/sample_heatmap/*/......4
1.5.7 PERMANOVA/Anosim 分析: ./Beta_diversity/permanova_anosim_Dis_box/*/......4
1.5.8 RDA/CCA：./RDA_or_CCA/*/......错误!未定义书签。
1.6 组间差异显著性分析：./Significant_features/......4
1.6.1 Lefse 分析：./Significant_features/LEfSe/*/......4
1.6.2 Metastats 分析：./Significant_features/Metastats/*/......4
1.7 统计检验：./Statistical/......错误!未定义书签。
1.7.1 方差分析：./Statistical/Anova/*/......5
1.7.2 秩和检验：./Statistical/Wilcox/*/......5
1.7.3 三元相图：./Statistical/Ternary/*/......5
1.8 16S 功能基因预测分析：./Function_prediction/......5
1.8.1 KEGG 功能预测：./Function_prediction/KEGG/......5
1.8.2 COG 功能预测：./Function_prediction/COG/......6
1.9 相关性分析：./Network/*/......错误!未定义书签。
1.10 丰度表：./Abundance_dir/*/......6

1 结果目录

1.1 测序数据质量评估：./data_assement/

AllSample_GC_Q.stat.xls：	各样品测序数据处理结果统计表
sample_information.xls：	样品编号对应表
*_reads_length.(pdf|png)：	单样品 Effective Tags 长度分布图

1.2 OTU 分析：./otus/

otu_distribution.xls：	样品各等级 OTU 聚类结果统计表
classfy_distribution.xls：	样品各等级 OTU 物种统计表
rep_set.fasta：	OTU 代表序列
allsample.txt：	OTU 物种注释结果
allsample.biom：	biom 格式 OTU 表格
OTU_distri/*/*.otu_stat.xls：	样品 OTU 信息统计表
OTU_distri/*/*.otu.bar.(svg|png)：	样品 OTU 分布图
venn/*/*_venn.(pdf|png)：	不同样品组合的韦恩图
venn/*/*_new_otu_tab.xls：	韦恩图对应的 OTU 表
flower/*/*_flower.(pdf|png)：	不同样品组合的花瓣图
flower/*/*_flower_stat.xls：	花瓣图对应的 OTU 表

1.3 物种注释及分类学分析： ./taxa_summary/

1.3.1 物种分布分析： ./taxa_summary/Taxa_dis/*/

sample/*.sample.*.bar.(svg|pdf|png)：	样品间各分类学水平的物种分布柱状图
group/*group.*.bar.(svg|pdf|png)：	组间各分类学水平的物种分布柱状图

1.3.2 物种丰度聚类分析：./taxa_summary/heatmap/*/

*.reabundance.heatmap.(pdf|png)：	各分类学水平的物种丰度聚类热图

1.3.3 MEGAN 分类学分析：./taxa_summary/taxa_tree/和 krona/

taxa_tree/samples/Single.*.tx.(pdf|png)：	单样品分类学树状图
taxa_tree/groups/*/Group.tx.(png|pdf)：	多样品分类学树状图
krona/samples/*.Krona.html：	样品物种注释结果的 KRONA 图

krona/groups/*/GroupKrona.html： 分组物种注释结果的 KRONA 图

1.3.4 系统进化树：./taxa_summary/Phylogenetic_tree/*/

genus.rep.(pdf|png)： 属水平物种系统进化树

1.4 Alpha 多样性分析：./alpha_div/

alpha_diversity.xls： Alpha 多样性指数计算结果(包含所有样品)

1.4.1 Alpha 多样性指数统计：./alpha_div/alpha_index_stat/*/

*.alpha_diversity.xls： Alpha 多样性指数结果表格
*.(ACE|Chao1|Shannon|Simpson).(pdf|svg|png) Alpha 多样性指数柱状图
*.p_value_table.txt Alpha 多样性指数组间差异比较 p_value 结果
*.(ACE|Chao1|Shannon|Simpson)_index_diff.(pdf|svg|png) Alpha 多样性指数组间差异比较柱状图
*.alpha_index_stats.xls Alpha 多样性指数组内统计结果(含有各指数平均值和标准误)

1.4.2 稀释性曲线：./alpha_div/rarefaction_curve/*/

*.rarefaction_curve_data.xls 稀释性曲线数据
*.Rarefaction.curve_group.(png|pdf|svg) 稀释性曲线图

1.4.3 Shannon 曲线：./alpha_div/shannon_index_curve/*/

*.shannon_curve_data.xls： Shannon 曲线数据
*.shannon.curve_group.svg: Shannon 曲线图

1.4.4 等级丰度曲线：./alpha_div/rank_abund_curve/*/

*.rank.abund.curve.(png|pdf|svg)： 各主成分的贡献率

1.4.5 物种累计曲线：./alpha_div/specaccum/*/

..specaccum.(png|svg)：	不同分类等级的物种丰度曲线

1.5 Beta 多样性分析：./Beta_diversity/

1.5.1 PCA 分析：./Beta_diversity/pca/*/

*.userLabel.pca.loadings：	各主成分的贡献率
*.userLabel.pca.axes：	各样品的坐标
PCA.*.(pdf|png)：	PCA 图

1.5.2 PCoA 分析：./Beta_diversity/pcoa/*/

*.loadings：	各主坐标的贡献率
*.axes：	各主坐标的坐标
.binary_jaccard..(svg|png)：	基于 binary jaccard 的 PCoA 图
.bray_curtis..(svg|png)：	基于 bray_curtis 的 PCoA 图
.unweighted_unifrac..(svg|png)：	基于 unweighted_unifrac 的 PCoA 图(限细菌)
.weighted_unifrac..(svg|png)：	基于 weighted_unifrac 的 PCoA 图(限细菌)

1.5.3 NMDS 分析：./Beta_diversity/nmds/*/

*.axes：	各 nmds 的坐标
.binary_jaccard..(svg|png)：	基于 binary jaccard 的 NMDS 图
.bray_curtis..(svg|png)：	基于 bray_curtis 的 NMDS 图
.unweighted_unifrac..(svg|png)：	基于 unweighted_unifrac 的 NMDS 图(限细菌)
.weighted_unifrac..(svg|png)：	基于 weighted_unifrac 的 NMDS 图(限细菌)

1.5.4 层次聚类分析：./Beta_diversity/upgma_tree/*/

.binary_jaccard..(svg|png)：	基于 binary jaccard 的 UPGMA 聚类树
.bray_curtis..(svg|png)：	基于 bray_curtis 的 UPGMA 聚类树
.unweighted_unifrac..(svg|png)：	基于 unweighted_unifrac 的 UPGMA 聚类树(限细菌)
.weighted_unifrac..(svg|png)：	基于 weighted_unifrac 的 UPGMA 聚类树(限细菌)
.binary_jaccard..tre：	基于 binary jaccard 的 UPGMA 聚类树
.bray_curtis..tre：	基于 bray_curtis 的 UPGMA 聚类树
.unweighted_unifrac..tre：	基于 unweighted_unifrac 的 UPGMA 聚类树(限细菌)
.weighted_unifrac..tre：	基于 weighted_unifrac 的 UPGMA 聚类树(限细菌)

1.5.5 层次聚类与柱状图分析：./Beta_diversity/ClusterTree_bar/*/

.binary_jaccard..(svg|png)：	基于 binary jaccard 的树柱图
.bray_curtis..(svg|png)：	基于 bray_curtis 的树柱图
.unweighted_unifrac..(svg|png)：	基于 unweighted_unifrac 的树柱图
.weighted_unifrac..(svg|png)：	基于 weighted_unifrac 的树柱图

1.5.6 样品热图分析：./Beta_diversity/sample_heatmap/*/

*.binary_jaccard_dm.heatmap.(pdf|png)：	基于 binary jaccard 的热图
*.bray_curtis_dm.heatmap.(pdf|png)：	基于 bray_curtis 的热图
*.unweighted_unifrac_dm.heatmap.(pdf|png)：	基于 unweighted_unifrac 的热图(限细菌)
*.weighted_unifrac_dm.heatmap.(pdf|png)：	基于 weighted_unifrac 的热图(限细菌)

1.5.7 PERMANOVA/Anosim 分析: ./Beta_diversity/permanova_anosim_Dis_box/*/

*.binary_jaccard.(anosim|permanova).DistMatrixBoxplot.(svg|png)：	基于 binary jaccard 的距离箱线图
*.bray_curtis.(anosim|permanova).DistMatrixBoxplot.(svg|png)：	基于 bray_curtis 的距离箱线图
*.unweighted_unifrac.(anosim|permanova).DistMatrixBoxplot.(svg|png)：	基于 unweighted_unifrac 的距离箱线图(限细菌)
*.weighted_unifrac.(anosim|permanova).DistMatrixBoxplot.(svg|png)：	基于 weighted_unifrac 的距离箱线图(限细菌)

1.6 组间差异显著性分析：./ difference_analysis/

1.6.1 Lefse 分析：./ difference_analysis /LEfSe/*/

species_lefse_LDA*_less_strict.rep：	Lefse 分析结果
lefse_biomarkers_less_strict.(pdf|png)：	LDA 值分布柱状图
lefse_biomarkers_cladogram_less_strict.(pdf|png)：	LEfSe 分析进化分枝图
1_*.(pdf|png)：	样品丰度比较图

1.6.2 Metastats 分析：./ difference_analysis /Metastats/*/

*.diffAb.xls：	Metastats 结果统计表

1.6.3 方差分析：./ difference_analysis /Anova/*/

*.ANOVA.xls：	各分类学水平的方差分析结果
*.ANOVA.(svg|png)：	各分类学水平的相对丰度柱状图

1.6.4 秩和检验：./ difference_analysis /*/

*.rank_sum.xls：	各分类学水平的秩和检验结果
*.rank_sum.(svg|png)：	各分类学水平的相对丰度柱状图

1.6.5 三元相图：./ difference_analysis /Ternary/*/

.all.abundance..(svg|png)：	三样品的三元相图

1.7 相关性与关系分析：./ association_analysis/

1.7.1 RDA/CCA：. /association_analysis /RDA_or_CCA/*/

*.*_compontents.1.(pdf|png)：	RDA/CCA 物种和环境因子图
*.*_samples.1.(pdf|png)	RDA/CCA 样本和环境因子图
biplot.txt	环境因子坐标值表格
sample_pos.xls	样本坐标值表格
species_pos.xls	物种坐标值表格

1.7.2 相关性网络分析：. /association_analysis / Network/*/

*edge.txt：	边属性统计表
*node.txt：	节点属性统计表
*network.property.txt：	网络属性统计表
*.genus.network.(svg|png)：	属水平网络图

1.8 功能基因预测分析：./ function_analysis/

1.8.1 KEGG 功能预测：./ function_analysis/KEGG/

KEGG.Kocount.spf：	KEGG 功能丰度表

/2samples/KEGG.Kocount.spf.All_Class2..xls： 两个样品间 KEGG 代谢途径差异分析结果
/2samples/KEGG.Kocount.spf.All_Class2..(svg|png)： 两个样品间 KEGG 代谢途径差异分析图
/2groups/KEGG.Kocount.spf.All_Class2..xls： 两组间 KEGG 代谢途径差异分析结果
/2groups/KEGG.Kocount.spf.All_Class2..(svg|png)： 两组间 KEGG 代谢途径差异分析图

1.8.2 COG 功能预测：./ function_analysis/COG/

COG.Kocount.spf： COG 功能丰度表
/2samples/COG.Kocount.spf.All_Class2..xls： 两个样品间 COG 功能分类差异分析结果
/2samples/COG.Kocount.spf.All_Class2..(svg|png)： 两个样品间 COG 功能分类差异分析图
/2groups/COG.Kocount.spf.All_Class2..xls： 两组间 COG 功能分类差异分析结果
/2groups/COG.Kocount.spf.All_Class2..(svg|png)： 两组间 COG 功能分类差异分析图

1.8.3 BugBase 表型预测：./ function_analysis/ BugBase/*/

normalized_otus 标准化后的 OTU 表
otu_contributions 各水平下 9 种表型或功能预测结果表
predicted_phenotypes 各水平下 9 种表型或功能预测结果表
thresholds 包含分析使用的阈值和不同阈值下的结果

1.9 丰度表：./Abundance_dir/*/

*.abundance： 各分类学水平的绝对丰度表
*.reabundance： 各分类学水平的相对丰度表
*.biom： biom 格式的 OTU 丰度表
*.txt： OTU 丰度表
*.group： 分组表

注：若合同中未签某种分析或样品不适合做某种分析，则结题报告及结果文件中不会提供相应的结果。
