# Supplementary material for: Effect of tetracycline on nitrogen removal in Moving Bed Biofilm Reactor (MBBR) System
Source: PLoS One. 2022 Jan 10;17(1):e0261306. doi: 10.1371/journal.pone.0261306 (PMC8746769; doi:10.1371/journal.pone.0261306)
Supplement: S2 Data — (ZIP) [file pone.0261306.s002.zip › customer_backup/taxa_summary/krona/samples/B1.Krona.html]

Javascript must be enabled to view this page.

members
magnitude
magnitudeUnassigned

B1.krona

63847

63847

9000

7908

4008

99

35

35

64

64

211

10

10

3

3

113

113

71

71

14

14

3698

81

81

3617

3617

1191

332

327

327

5

5

831

11

11

22

22

798

684

114

26

26

26

2

2

2

766

2

1

1

1

1

1

1

1

2

2

2

636

636

636

82

2

2

8

8

1

1

71

71

9

9

2

6

1

30

30

30

2

1

1

1

1

2

2

2

349

45

45

45

1

1

1

43

6

6

37

37

154

154

154

6

6

6

100

100

100

45

45

45

45

1549

906

202

202

704

52

652

109

41

41

68

68

91

91

91

443

443

443

1092

1023

1023

1023

1023

35

10

10

10

25

25

25

27

27

27

27

7

7

7

7

90

88

88

88

88

88

2

2

2

2

2

61

43

43

43

43

43

18

18

18

18

18

13

13

13

13

13

13

4029

3789

3789

3789

42

42

3686

3686

61

61

6

6

6

6

6

110

110

110

110

110

107

4

4

4

4

103

103

2

2

1

1

100

31

69

15

15

15

15

15

2

2

2

2

2

423

215

215

215

215

215

67

45

45

45

45

22

22

22

22

1

1

1

1

1

140

140

129

129

129

11

3

3

8

8

2

2

2

2

2

2

906

906

906

906

906

906

80

80

80

80

77

77

3

3

1046

1046

1046

1046

1046

1002

44

2

2

2

2

2

2

238

27

27

23

23

23

3

3

3

1

1

1

197

2

2

2

2

6

6

3

3

1

1

2

2

1

1

1

1

3

3

3

3

7

6

6

6

1

1

1

167

167

167

35

2

130

11

3

3

3

4

4

4

1

1

1

1

1

1

1

1

1

1

1

1

14

14

7

7

7

2

1

1

1

1

1

1

1

4

3

3

1

1

38902

1406

20

20

20

20

28

28

28

28

34

26

26

26

8

8

8

80

80

2

2

25

25

20

20

13

13

7

7

1

1

12

12

8

8

8

8

32

32

32

32

52

52

36

36

2

2

14

14

281

13

8

8

2

2

3

3

107

107

107

30

30

30

26

11

9

2

15

15

3

3

3

75

23

23

1

1

50

50

1

1

12

7

7

2

2

3

3

15

15

15

400

285

9

9

247

247

29

29

115

7

7

3

3

105

105

7

6

6

6

1

1

1

10

10

10

10

391

391

93

93

122

122

143

143

31

31

2

2

63

63

63

63

37306

11888

11888

524

524

11364

11364

1

1

1

1

39

39

39

39

47

47

47

47

228

228

228

228

8

8

8

8

124

124

124

124

316

217

79

79

23

23

4

4

111

111

99

1

1

24

24

5

5

67

65

2

2

2

24287

607

67

67

34

34

506

5

501

22362

102

102

1002

1002

207

207

7752

7752

13027

13027

28

28

156

156

9

9

79

79

9

9

9

2

2

2

1307

328

328

11

11

7

7

1

1

1

1

2

2

926

926

27

27

2

2

1

1

1

1

1

1

1

1

5

5

5

5

278

278

58

58

31

31

189

189

1

1

1

1

2

2

2

2

81

42

42

42

39

39

39

190

69

1

1

1

19

19

19

14

14

14

34

34

34

1

1

1

15

9

9

9

6

3

3

3

3

4

4

4

4

100

100

100

51

49

2

1

1

1

1

1

1

531

1

1

1

1

1

206

206

206

206

206

324

324

324

324

324

93

65

65

65

65

65

28

28

28

25

25

3

3

5650

27

27

27

27

27

63

59

59

59

59

4

4

4

4

12

12

12

12

12

5517

94

94

94

94

23

23

23

23

14

14

14

14

95

95

95

95

20

20

20

20

57

57

57

57

132

132

9

9

101

101

1

1

21

2

19

5082

4974

4876

4876

98

98

108

108

108

3

3

3

3

3

28

28

28

28

28

1001

561

561

125

2

2

1

1

1

1

1

1

12

12

58

58

9

9

41

41

4

4

4

5

1

1

4

4

53

5

5

1

1

47

47

6

6

6

352

24

24

75

75

35

35

9

9

2

2

25

25

84

84

71

71

6

6

5

5

16

16

5

5

5

11

1

1

10

10

384

28

11

10

10

1

1

1

1

1

16

16

16

356

2

2

2

354

354

354

28

28

2

2

2

26

18

18

8

8

28

28

28

2

2

25

25

1

1

22

22

22

22

12

12

1

1

9

9

395

395

395

395

395

395

4

4

4

4

4

4

1359

1359

2

2

2

2

51

51

8

8

31

31

5

5

7

7

1088

926

545

545

319

319

62

62

146

146

146

14

14

14

2

2

2

218

218

1

1

52

52

156

156

9

9
