# Supplementary material for: Effect of tetracycline on nitrogen removal in Moving Bed Biofilm Reactor (MBBR) System
Source: PLoS One. 2022 Jan 10;17(1):e0261306. doi: 10.1371/journal.pone.0261306 (PMC8746769; doi:10.1371/journal.pone.0261306)
Supplement: S2 Data — (ZIP) [file pone.0261306.s002.zip › customer_backup/taxa_summary/krona/groups/treat/B3.Krona.html]

Javascript must be enabled to view this page.

members
magnitude
magnitudeUnassigned

B3.krona

60017

60017

9778

7353

2845

16

16

16

376

5

5

371

371

2091

60

60

55

55

1617

1617

1

1

260

260

98

98

362

23

23

339

339

1397

1180

1163

1163

17

17

201

2

2

1

1

198

76

30

92

6

6

4

2

10

10

10

496

10

7

7

3

3

1

1

1

250

250

250

33

33

33

27

6

6

12

12

7

7

2

2

1

1

1

78

78

1

72

5

9

6

6

3

3

62

62

62

25

1

1

14

14

10

10

1576

55

55

55

16

14

14

1

1

1

1

47

3

3

44

44

31

31

31

9

9

9

1418

1418

1418

31

31

31

31

1008

168

24

24

144

9

135

336

86

86

250

250

23

23

23

481

477

477

4

4

2425

2253

2253

2253

2253

50

34

34

34

16

16

16

87

87

87

87

35

35

35

35

646

646

646

646

646

646

0

0

0

0

0

65

4

4

4

4

4

24

24

24

24

24

37

37

37

37

37

4

4

4

4

4

4

12526

12100

8

8

8

8

12092

12092

46

46

11944

11944

102

102

11

11

11

11

11

66

66

66

66

66

296

5

5

5

5

291

291

6

6

4

4

281

22

259

3

3

3

3

3

1

1

1

1

1

0

0

0

0

0

49

48

48

48

48

1

1

1

1

196

79

79

79

79

79

9

6

6

6

6

1

1

1

1

2

2

2

2

22

5

5

5

5

17

17

17

17

86

86

83

83

83

3

2

2

1

1

3

3

3

3

3

3

0

0

0

0

0

0

20

20

20

20

20

20

583

583

583

583

540

540

43

43

281

281

281

281

281

131

150

3

3

3

3

3

3

563

44

44

12

12

12

17

4

4

13

13

15

15

15

3

1

1

1

1

2

2

2

2

404

0

0

0

0

9

9

9

9

8

8

7

7

0

0

1

1

0

0

0

0

5

5

5

5

261

261

256

18

87

151

5

5

24

21

21

21

3

3

3

39

37

37

37

2

2

2

15

15

15

15

40

6

6

6

1

1

1

6

6

6

22

2

2

19

19

1

1

3

3

3

2

2

2

3

0

0

0

3

3

3

112

112

31

31

31

15

15

15

36

30

30

6

6

30

13

12

1

13

13

4

4

1

1

1

1

1

1

24964

9692

175

14

14

14

161

161

161

155

155

155

155

106

50

50

50

56

56

56

1319

1319

47

47

314

314

266

266

113

113

441

441

42

42

96

96

14

14

14

14

310

310

310

310

624

624

61

61

367

367

196

3

193

2282

416

347

347

6

6

1

1

8

8

54

54

22

22

22

439

439

439

197

197

197

70

70

70

322

262

31

231

5

5

55

55

304

74

74

6

6

215

215

9

9

397

153

153

204

204

40

40

115

8

8

3

3

32

32

72

72

1957

1159

57

57

1000

1000

102

102

798

46

46

9

9

743

743

6

6

6

6

0

0

0

39

39

39

39

2504

2504

4

4

217

217

849

849

1212

1212

218

218

4

4

201

201

201

201

14914

4281

4281

3006

3006

1275

1275

12

12

12

12

5

5

5

5

5

5

5

5

4

4

4

4

34

34

34

34

1660

779

87

87

535

535

49

49

108

108

881

2

2

179

176

3

42

42

614

587

27

44

44

7876

15

15

15

1596

24

24

7

7

509

509

1056

32

59

965

3247

73

73

751

751

180

180

941

941

2

2

550

550

8

8

681

681

1

1

46

46

14

14

13

13

13

446

446

446

11

11

11

2548

940

940

2

2

5

5

5

5

9

9

1

1

1398

1398

1

1

6

6

8

8

7

7

4

4

2

2

4

4

10

10

1

1

8

8

3

3

4

4

21

21

78

78

6

6

25

25

56

56

56

56

108

108

5

5

41

41

62

62

538

363

363

363

175

175

175

3

3

3

3

102

102

102

102

88

88

88

88

4

4

4

4

0

0

3

3

3

3

3

3

3

3

1

1

1

1

118

118

118

118

13

13

13

13

358

107

1

1

1

17

17

17

0

0

0

5

5

5

15

15

15

48

48

48

20

20

20

1

1

1

65

45

45

45

20

19

19

1

1

17

17

17

17

6

2

2

2

4

4

4

163

163

163

118

45

1123

0

0

0

0

0

47

15

15

15

15

32

32

32

32

866

866

866

866

866

0

0

210

210

210

210

210

3

3

3

3

3

3

304

54

54

54

54

54

250

250

250

250

250

0

0

0

0

0

0

0

4758

62

62

62

62

62

229

228

5

5

5

223

223

223

1

1

1

1

0

0

0

0

0

4405

86

86

86

86

25

25

25

25

119

119

119

119

623

623

623

623

4

4

4

4

545

545

545

545

471

471

24

24

348

348

13

13

0

0

86

0

86

2532

2431

2421

2421

10

10

101

101

101

38

38

38

38

38

24

24

24

24

24

2088

1158

3

3

3

3

62

62

62

62

1093

423

19

19

69

69

4

4

54

54

3

3

46

46

19

19

4

4

84

84

8

8

113

113

2

2

2

19

12

12

0

0

7

7

47

1

1

4

4

42

42

17

17

14

3

441

14

14

21

21

46

46

27

27

8

8

79

79

180

180

15

15

11

1

10

17

17

23

23

17

17

17

123

3

2

1

116

116

4

4

4

4

4

478

385

107

106

106

1

1

3

3

3

12

12

12

263

263

193

65

5

93

5

5

5

88

1

1

87

87

31

31

1

1

1

30

3

3

18

18

9

9

421

421

421

7

7

72

72

29

29

10

10

303

303

57

57

57

57

3

3

2

2

50

50

2

2

214

214

214

214

214

214

0

0

0

0

0

0

29

29

23

23

23

23

6

6

6

6

9

9

9

8

8

8

1

1

1

1799

1799

22

22

22

22

105

105

15

15

11

11

71

71

8

8

1623

365

101

101

246

246

18

18

776

776

776

2

2

2

480

480

480

49

49

0

0

9

9

7

7

33

33
