# Supplementary material for: Effect of tetracycline on nitrogen removal in Moving Bed Biofilm Reactor (MBBR) System
Source: PLoS One. 2022 Jan 10;17(1):e0261306. doi: 10.1371/journal.pone.0261306 (PMC8746769; doi:10.1371/journal.pone.0261306)
Supplement: S2 Data — (ZIP) [file pone.0261306.s002.zip › customer_backup/taxa_summary/krona/groups/treat/B1.Krona.html]

Javascript must be enabled to view this page.

members
magnitude
magnitudeUnassigned

B1.krona

63847

63847

9000

7908

4008

0

0

0

99

35

35

64

64

211

10

10

3

3

113

113

0

0

71

71

14

14

3698

81

81

3617

3617

1191

332

327

327

5

5

831

11

11

22

22

798

684

0

114

26

26

0

26

2

2

2

766

2

1

1

1

1

1

1

1

2

2

2

636

636

636

82

2

2

8

8

1

1

71

71

0

0

0

9

9

2

6

1

2

1

1

1

1

30

30

30

2

2

2

0

0

0

0

349

1

1

1

45

45

45

0

0

0

0

43

6

6

37

37

154

154

154

6

6

6

100

100

100

45

45

45

45

1549

906

202

202

704

52

652

109

41

41

68

68

91

91

91

443

443

443

0

0

1092

1023

1023

1023

1023

35

10

10

10

25

25

25

27

27

27

27

7

7

7

7

90

88

88

88

88

88

2

2

2

2

2

61

0

0

0

0

0

43

43

43

43

43

18

18

18

18

18

13

13

13

13

13

13

4029

3789

0

0

0

0

3789

3789

42

42

3686

3686

61

61

6

6

6

6

6

110

110

110

110

110

107

4

4

4

4

103

103

2

2

1

1

100

31

69

0

0

0

0

0

0

0

0

0

0

15

15

15

15

15

2

2

2

2

2

0

0

0

0

423

215

215

215

215

215

67

45

45

45

45

0

0

0

0

22

22

22

22

1

1

1

1

1

0

0

0

0

140

140

129

129

129

11

3

3

8

8

0

0

0

0

0

0

2

2

2

2

2

2

906

906

906

906

906

906

80

80

80

80

77

77

3

3

1046

1046

1046

1046

1046

1002

44

2

2

2

2

2

2

238

27

27

23

23

23

3

0

0

3

3

1

1

1

0

0

0

0

0

0

0

0

0

197

0

0

0

0

2

2

2

2

6

6

0

0

3

3

1

1

0

0

2

2

1

1

1

1

167

167

167

35

2

130

0

0

0

0

0

0

0

0

0

7

6

6

6

1

1

1

3

3

3

3

11

3

3

3

4

4

4

1

1

1

1

0

0

0

0

1

1

1

1

1

1

1

1

0

0

0

0

0

0

0

14

14

7

7

7

1

1

1

4

3

3

1

1

2

1

1

0

1

1

0

0

0

0

0

0

0

0

38902

1406

20

0

0

0

20

20

20

28

28

28

28

34

26

26

26

8

8

8

80

80

2

2

25

25

20

20

13

13

7

7

1

1

12

12

8

8

8

8

32

32

32

32

52

52

36

36

2

2

14

0

14

281

13

8

8

0

0

0

0

2

2

3

3

0

0

0

107

107

107

30

30

30

3

3

3

26

11

9

2

0

0

15

15

75

23

23

1

1

50

50

1

1

12

7

7

2

2

3

3

15

0

0

0

0

0

0

15

15

400

285

9

9

247

247

29

29

115

7

7

3

3

105

105

7

6

6

6

1

1

1

10

10

10

10

391

391

0

0

93

93

122

122

143

143

31

31

2

2

63

63

63

63

37306

11888

11888

524

524

11364

11364

0

0

0

0

1

1

1

1

39

39

39

39

8

8

8

8

124

124

124

124

316

217

79

79

23

23

4

4

111

111

99

1

1

24

24

0

5

5

67

65

2

2

2

24287

0

0

0

607

67

67

0

0

34

34

506

0

5

501

22362

102

102

1002

1002

207

207

7752

7752

0

0

13027

13027

28

28

156

156

0

0

9

9

79

79

0

0

0

9

9

9

2

2

2

1307

328

328

0

0

1

1

1

1

0

0

0

0

926

926

1

1

0

0

0

0

0

0

0

0

0

0

0

0

11

11

0

0

7

7

0

0

2

2

27

27

2

2

0

0

1

1

5

5

5

5

278

278

58

58

31

31

189

189

81

42

42

42

39

39

39

0

0

0

0

47

47

47

47

228

228

228

228

0

0

0

0

0

0

0

0

0

0

1

1

1

1

0

0

0

0

1

1

1

1

2

2

2

2

190

69

0

0

0

34

34

34

1

1

1

0

0

0

1

1

1

19

19

19

14

14

14

0

0

0

15

9

9

9

6

3

3

3

3

4

4

4

4

2

1

1

1

1

1

1

100

100

100

51

49

531

1

1

1

1

1

0

0

0

0

0

0

0

0

0

206

206

206

206

206

0

0

324

324

324

324

324

0

0

0

0

0

0

93

65

65

65

65

65

28

28

28

25

25

3

3

0

0

0

0

0

5650

27

27

27

27

27

63

59

0

0

0

59

59

59

4

4

4

4

12

12

12

12

12

5517

94

94

94

94

23

23

23

23

14

14

14

14

95

95

95

95

20

20

20

20

57

57

57

57

132

132

9

9

101

101

1

1

0

0

21

2

19

5082

4974

4876

4876

98

98

108

108

108

3

3

3

3

3

28

28

28

28

28

1001

561

0

0

0

0

0

0

0

0

561

125

0

0

0

0

0

0

2

2

1

1

1

1

12

12

1

1

58

58

9

9

41

41

4

4

4

5

0

0

1

1

4

4

53

5

5

1

1

47

47

6

6

6

0

352

24

24

75

75

35

35

9

9

2

2

25

25

84

84

71

71

6

0

6

5

5

16

16

5

5

5

11

0

0

0

1

1

10

10

0

0

0

384

28

11

10

10

1

1

0

0

0

1

1

1

16

16

16

0

0

356

2

2

2

354

0

0

354

354

28

28

2

2

2

26

0

0

18

18

8

8

28

28

28

0

0

2

2

25

25

1

1

0

0

22

22

22

22

12

12

1

1

0

0

9

9

395

395

395

395

395

395

0

0

0

0

0

0

4

4

4

4

4

4

0

0

0

0

0

0

0

0

0

0

0

0

0

1359

1359

2

2

2

2

51

51

8

8

31

31

5

5

7

7

1088

926

545

545

319

319

62

62

146

146

146

14

14

14

2

2

2

218

218

1

1

52

52

156

156

9

9
