# Supplementary material for: Effect of tetracycline on nitrogen removal in Moving Bed Biofilm Reactor (MBBR) System
Source: PLoS One. 2022 Jan 10;17(1):e0261306. doi: 10.1371/journal.pone.0261306 (PMC8746769; doi:10.1371/journal.pone.0261306)
Supplement: S2 Data — (ZIP) [file pone.0261306.s002.zip › customer_backup/taxa_summary/krona/groups/treat/B2.Krona.html]

Javascript must be enabled to view this page.

members
magnitude
magnitudeUnassigned

B2.krona

54707

54707

9446

9020

4616

0

0

0

96

34

34

62

62

182

21

21

7

7

48

48

0

0

68

68

38

38

4338

41

41

4297

4297

2461

451

441

441

10

10

1979

31

31

101

101

1847

1494

2

351

30

30

0

30

1

1

1

322

3

2

2

1

1

0

0

0

6

6

6

216

216

216

46

2

2

6

6

4

4

34

34

0

0

0

16

16

9

7

0

4

2

2

2

2

14

14

14

17

7

7

5

5

5

5

242

0

0

0

44

44

44

0

0

0

0

49

9

9

40

40

91

91

91

9

9

9

49

49

49

53

53

53

53

1326

887

149

149

738

118

620

105

53

53

52

52

38

38

38

296

296

296

0

0

426

395

395

395

395

20

3

3

3

17

17

17

7

7

7

7

4

4

4

4

122

120

120

120

120

120

2

2

2

2

2

71

0

0

0

0

0

41

41

41

41

41

30

30

30

30

30

5

5

5

5

5

5

3634

3460

2

2

2

2

3458

3458

29

29

3368

3368

61

61

0

0

0

0

0

71

71

71

71

71

80

4

4

4

4

76

76

5

5

10

10

61

8

53

2

2

2

2

2

0

0

0

0

0

15

15

15

15

15

6

6

6

6

6

0

0

0

0

839

592

592

592

592

592

109

68

68

68

68

0

0

0

0

41

41

41

41

7

2

2

2

2

5

5

5

5

131

131

112

112

112

19

9

9

10

10

20

20

20

20

20

20

0

0

0

0

0

0

599

599

599

599

599

599

62

62

62

62

56

56

6

6

327

327

327

327

327

319

8

0

0

0

0

0

0

397

48

48

36

36

36

6

0

0

6

6

6

6

6

0

0

0

0

0

0

0

0

0

324

1

1

1

1

4

4

4

4

10

10

3

3

5

5

0

0

2

2

0

0

0

0

0

0

286

286

286

61

1

224

0

0

1

0

0

0

1

1

1

4

4

4

4

0

0

0

0

0

0

0

17

2

2

2

7

7

7

3

3

3

5

2

2

2

2

1

1

0

0

0

0

0

0

1

1

1

1

0

0

0

25

25

14

14

14

2

2

2

3

1

1

2

2

6

1

1

0

3

3

2

2

0

0

0

0

0

0

27753

1758

18

2

2

2

16

16

16

25

25

25

25

16

12

12

12

4

4

4

118

118

9

9

33

33

22

22

21

21

20

20

3

3

10

10

12

12

12

12

32

32

32

32

59

59

48

48

0

0

11

0

11

243

20

16

16

0

0

0

0

0

0

4

4

4

4

4

71

71

71

47

47

47

3

3

3

17

1

1

0

1

1

15

15

63

11

11

2

2

47

47

3

3

2

1

1

1

1

0

0

16

0

0

0

0

1

1

15

15

508

311

9

9

274

274

28

28

197

1

1

3

3

193

193

3

0

0

0

3

3

3

5

5

5

5

625

625

0

0

126

126

173

173

217

217

109

109

0

0

94

94

94

94

25604

6632

6632

254

254

6378

6378

1

1

1

1

0

0

0

0

1

1

1

1

18

18

18

18

62

62

62

62

516

405

277

277

31

31

2

2

95

95

111

0

0

43

43

0

2

2

66

64

2

0

0

17626

0

0

0

525

8

8

0

0

48

48

469

0

1

468

15862

73

73

849

849

78

78

3850

3850

0

0

10813

10813

25

25

120

120

0

0

9

9

45

45

4

4

4

8

8

8

6

6

6

1221

338

338

1

1

0

0

0

0

1

1

1

1

833

833

0

0

10

10

2

2

1

1

3

3

0

0

0

0

19

19

0

0

5

5

0

0

1

1

4

4

1

1

0

0

1

1

0

0

0

0

417

417

75

75

79

79

263

263

93

33

33

33

60

60

60

0

0

0

0

38

38

38

38

193

193

193

193

4

4

1

1

3

3

0

0

0

0

0

0

0

0

0

0

0

0

1

1

1

1

2

2

2

2

391

265

0

0

0

226

226

226

0

0

0

0

0

0

1

1

1

29

29

29

9

9

9

0

0

0

19

8

8

8

11

6

6

5

5

8

8

8

8

8

7

7

7

1

1

1

91

91

91

39

52

713

0

0

0

0

0

1

0

0

0

0

1

1

1

1

242

242

242

241

241

1

1

470

470

470

470

470

0

0

0

0

0

0

282

243

243

243

243

243

34

34

34

34

34

0

0

5

5

5

5

5

4033

18

18

18

18

18

29

29

0

0

0

29

29

29

0

0

0

0

24

24

24

24

24

3940

90

90

90

90

16

16

16

16

7

7

7

7

140

140

140

140

5

5

5

5

34

34

34

34

176

176

7

7

140

140

7

7

2

2

20

1

19

3472

3419

3399

3399

20

20

53

53

53

1

1

1

1

1

21

21

21

21

21

1655

1165

0

0

0

0

1

1

1

1

1164

314

0

0

40

40

0

0

1

1

1

1

0

0

23

23

4

4

108

108

18

18

119

119

18

18

18

11

2

2

1

1

8

8

72

2

2

4

4

66

66

6

6

5

1

705

21

21

126

126

73

73

27

27

1

1

64

64

206

206

127

127

9

2

7

14

14

37

37

14

14

14

24

0

0

0

5

5

19

19

0

0

0

362

68

26

24

24

2

2

3

3

3

7

7

7

32

32

31

0

1

294

2

2

2

292

0

0

292

292

52

52

4

4

4

48

0

0

32

32

16

16

76

76

76

0

0

6

6

65

65

1

1

4

4

66

66

66

66

21

21

34

34

0

0

11

11

571

571

571

571

571

571

1

1

1

1

1

1

5

5

5

5

5

5

0

0

0

0

1

1

1

1

1

1

0

0

0

4105

4105

5

5

5

5

47

47

11

11

24

24

7

7

5

5

3903

3386

2622

2622

533

533

231

231

464

464

464

45

45

45

8

8

8

150

150

2

2

16

16

128

128

4

4
