# Supplementary material for: Effect of tetracycline on nitrogen removal in Moving Bed Biofilm Reactor (MBBR) System
Source: PLoS One. 2022 Jan 10;17(1):e0261306. doi: 10.1371/journal.pone.0261306 (PMC8746769; doi:10.1371/journal.pone.0261306)
Supplement: S2 Data — (ZIP) [file pone.0261306.s002.zip › customer_backup/taxa_summary/krona/groups/treat/Group.Krona.html]

Javascript must be enabled to view this page.

members
magnitude
magnitudeUnassigned

B1.krona
B2.krona
B3.krona

638475470760017

638475470760017

900094469778

790890207353

400846162845

0016

0016

0016

9996376

35345

35345

6462371

6462371

2111822091

102160

102160

3755

3755

113481617

113481617

001

001

7168260

7168260

143898

143898

36984338362

814123

814123

36174297339

36174297339

119124611397

3324511180

3274411163

3274411163

51017

51017

8311979201

11312

11312

221011

221011

7981847198

684149476

0230

11435192

26306

26306

004

26302

2110

2110

2110

766322496

2310

127

127

113

113

101

101

101

26250

26250

26250

63621633

63621633

63621633

824627

226

226

8612

8612

147

147

71342

71342

001

001

001

91678

91678

291

6772

105

249

126

126

123

123

301462

301462

301462

21725

271

271

0514

0514

0510

0510

3492421576

1055

1055

1055

454416

454414

454414

001

001

001

001

434947

693

693

374044

374044

1549131

1549131

1549131

699

699

699

100491418

100491418

100491418

455331

455331

455331

455331

154913261008

906887168

20214924

20214924

704738144

521189

652620135

109105336

415386

415386

6852250

6852250

913823

913823

913823

443296481

443296477

443296477

004

004

10924262425

10233952253

10233952253

10233952253

10233952253

352050

10334

10334

10334

251716

251716

251716

27787

27787

27787

27787

7435

7435

7435

7435

90122646

88120646

88120646

88120646

88120646

88120646

220

220

220

220

220

617165

004

004

004

004

004

434124

434124

434124

434124

434124

183037

183037

183037

183037

183037

1354

1354

1354

1354

1354

1354

4029363412526

3789346012100

028

028

028

028

3789345812092

3789345812092

422946

422946

3686336811944

3686336811944

6161102

6161102

6011

6011

6011

6011

6011

1107166

1107166

1107166

1107166

1107166

10780296

445

445

445

445

10376291

10376291

256

256

1104

1104

10061281

31822

6953259

023

023

023

023

023

001

001

001

001

001

15150

15150

15150

15150

15150

2649

2648

2648

2648

2648

001

001

001

001

423839196

21559279

21559279

21559279

21559279

21559279

671099

45686

45686

45686

45686

001

001

001

001

22412

22412

22412

22412

1722

125

125

125

125

0517

0517

0517

0517

14013186

14013186

12911283

12911283

12911283

11193

392

392

8101

8101

0203

0203

0203

0203

0203

0203

200

200

200

200

200

200

90659920

90659920

90659920

90659920

90659920

90659920

8062583

8062583

8062583

8062583

7756540

7756540

3643

3643

1046327281

1046327281

1046327281

1046327281

1046327281

1002319131

448150

203

203

203

203

203

203

238397563

274844

274844

233612

233612

233612

3617

004

004

3613

3613

1615

1615

1615

003

001

001

001

001

002

002

002

002

197324404

010

010

010

010

249

249

249

249

6108

6108

037

037

350

350

101

101

020

020

200

200

105

105

105

105

167286261

167286261

167286256

356118

2187

130224151

005

005

0124

0021

0021

0021

013

013

013

7439

6437

6437

6437

102

102

102

3015

3015

3015

3015

111740

326

326

326

471

471

471

136

136

136

1522

022

022

0219

0219

111

111

103

103

103

102

102

102

013

010

010

010

003

003

003

1425112

1425112

71431

71431

71431

1215

1215

1215

4336

3130

3130

126

126

2630

1113

1112

001

1313

1313

024

024

001

001

001

001

001

001

389022775324964

140617589692

2018175

0214

0214

0214

2016161

2016161

2016161

2825155

2825155

2825155

2825155

3416106

261250

261250

261250

8456

8456

8456

801181319

801181319

2947

2947

2533314

2533314

2022266

2022266

1321113

1321113

720441

720441

1342

1342

121096

121096

81214

81214

81214

81214

3232310

3232310

3232310

3232310

5259624

5259624

364861

364861

20367

20367

1411196

003

1411193

2812432282

1320416

816347

816347

006

006

001

001

208

208

3454

3454

0422

0422

0422

10771439

10771439

10771439

3047197

3047197

3047197

3370

3370

3370

2617322

111262

9131

20231

015

015

151555

151555

7563304

231174

231174

126

126

5047215

5047215

139

139

122397

71153

71153

21204

21204

3040

3040

1516115

008

008

003

003

0132

0132

151572

151572

4005081957

2853111159

9957

9957

2472741000

2472741000

2928102

2928102

115197798

7146

7146

339

339

105193743

105193743

736

606

606

606

130

130

130

10539

10539

10539

10539

3916252504

3916252504

004

004

93126217

93126217

122173849

122173849

1432171212

1432171212

31109218

31109218

204

204

6394201

6394201

6394201

6394201

373062560414914

1188866324281

1188866324281

5242543006

5242543006

1136463781275

1136463781275

0112

0112

0112

0112

105

105

105

105

3915

3915

3915

3915

8184

8184

8184

8184

1246234

1246234

1246234

1246234

3165161660

217405779

7927787

7927787

2331535

2331535

4249

4249

11195108

11195108

99111881

102

102

2443179

2443176

003

5242

5242

6766614

6564587

2227

2044

2044

24287176267876

0015

0015

0015

6075251596

67824

67824

007

007

3448509

3448509

5064691056

0032

5159

501468965

22362158623247

1027373

1027373

1002849751

1002849751

20778180

20778180

77523850941

77523850941

002

002

1302710813550

1302710813550

28258

28258

156120681

156120681

001

001

9946

9946

794514

794514

0413

0413

0413

98446

98446

98446

2611

2611

2611

130712212548

328338940

328338940

012

012

105

105

105

105

019

019

011

011

9268331398

9268331398

101

101

0106

0106

028

028

017

017

034

034

002

002

004

004

111910

111910

001

001

758

758

003

003

214

214

27421

27421

2178

2178

006

006

1125

1125

5056

5056

5056

5056

278417108

278417108

58755

58755

317941

317941

18926362

18926362

8193538

4233363

4233363

4233363

3960175

3960175

3960175

003

003

003

003

4738102

4738102

4738102

4738102

22819388

22819388

22819388

22819388

044

044

014

014

030

030

003

003

003

003

103

103

103

103

001

001

001

001

11118

11118

11118

11118

2213

2213

2213

2213

190391358

69265107

001

001

001

3422617

3422617

3422617

100

100

100

005

005

005

1115

1115

1115

192948

192948

192948

14920

14920

14920

001

001

001

151965

9845

9845

9845

61120

3619

3619

351

351

4817

4817

4817

4817

286

172

172

172

114

114

114

10091163

10091163

10091163

5139118

495245

5317131123

100

100

100

100

100

0147

0015

0015

0015

0015

0132

0132

0132

0132

206242866

206242866

206242866

206241866

206241866

010

010

324470210

324470210

324470210

324470210

324470210

003

003

003

003

003

003

93282304

6524354

6524354

6524354

6524354

6524354

2834250

2834250

2834250

2534250

2534250

300

300

050

050

050

050

050

565040334758

271862

271862

271862

271862

271862

6329229

5929228

005

005

005

5929223

5929223

5929223

401

401

401

401

12240

12240

12240

12240

12240

551739404405

949086

949086

949086

949086

231625

231625

231625

231625

147119

147119

147119

147119

95140623

95140623

95140623

95140623

2054

2054

2054

2054

5734545

5734545

5734545

5734545

132176471

132176471

9724

9724

101140348

101140348

1713

1713

020

020

212086

210

191986

508234722532

497434192431

487633992421

487633992421

982010

982010

10853101

10853101

10853101

3138

3138

3138

3138

3138

282124

282124

282124

282124

282124

100116552088

56111651158

003

003

003

003

0162

0162

0162

0162

56111641093

125314423

0019

0019

04069

04069

004

004

2154

2154

113

113

1046

1046

122319

122319

144

144

5810884

5810884

9188

9188

41119113

41119113

4182

4182

4182

51119

0212

0212

110

110

487

487

537247

521

521

144

144

476642

476642

6617

6617

6514

013

352705441

242114

242114

7512621

7512621

357346

357346

92727

92727

218

218

256479

256479

84206180

84206180

7112715

7112715

6911

021

6710

51417

51417

163723

163723

51417

51417

51417

1124123

003

002

001

15116

15116

10194

10194

004

004

004

384362478

2868385

1126107

1024106

1024106

121

121

033

033

033

1712

1712

1712

1632263

1632263

1631193

0065

015

35629493

225

225

225

35429288

001

001

35429287

35429287

285231

285231

241

241

241

264830

003

003

183218

183218

8169

8169

2876421

2876421

2876421

007

007

2672

2672

256529

256529

1110

1110

04303

04303

226657

226657

226657

226657

12213

12213

1342

1342

0050

0050

9112

9112

395571214

395571214

395571214

395571214

395571214

395571214

010

010

010

010

010

010

4529

4529

4523

4523

4523

4523

006

006

006

006

019

019

019

018

018

018

001

001

001

135941051799

135941051799

2522

2522

2522

2522

5147105

5147105

81115

81115

312411

312411

5771

5771

758

758

108839031623

9263386365

5452622101

5452622101

319533246

319533246

6223118

6223118

146464776

146464776

146464776

14452

14452

14452

28480

28480

28480

21815049

21815049

120

120

52169

52169

1561287

1561287

9433

9433
