# Supplementary figures and images for: Effect of tetracycline on nitrogen removal in Moving Bed Biofilm Reactor (MBBR) System
Source: PLoS One. 2022 Jan 10;17(1):e0261306. doi: 10.1371/journal.pone.0261306 (PMC8746769; doi:10.1371/journal.pone.0261306)

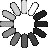

Supplement: S2 Data — (ZIP) [file pone.0261306.s002.zip › src/js/fancyBox/fancybox_loading@2x.gif]

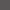

Supplement: S2 Data — (ZIP) [file pone.0261306.s002.zip › src/js/fancyBox/fancybox_overlay.png]

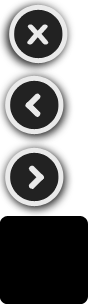

Supplement: S2 Data — (ZIP) [file pone.0261306.s002.zip › src/js/fancyBox/fancybox_sprite@2x.png]

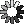

Supplement: S2 Data — (ZIP) [file pone.0261306.s002.zip › src/js/fancyBox/fancybox_loading.gif]

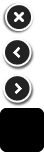

Supplement: S2 Data — (ZIP) [file pone.0261306.s002.zip › src/js/fancyBox/fancybox_sprite.png]

Supplement: S2 Data — (ZIP) [file pone.0261306.s002.zip › src/js/fancyBox/blank.gif]

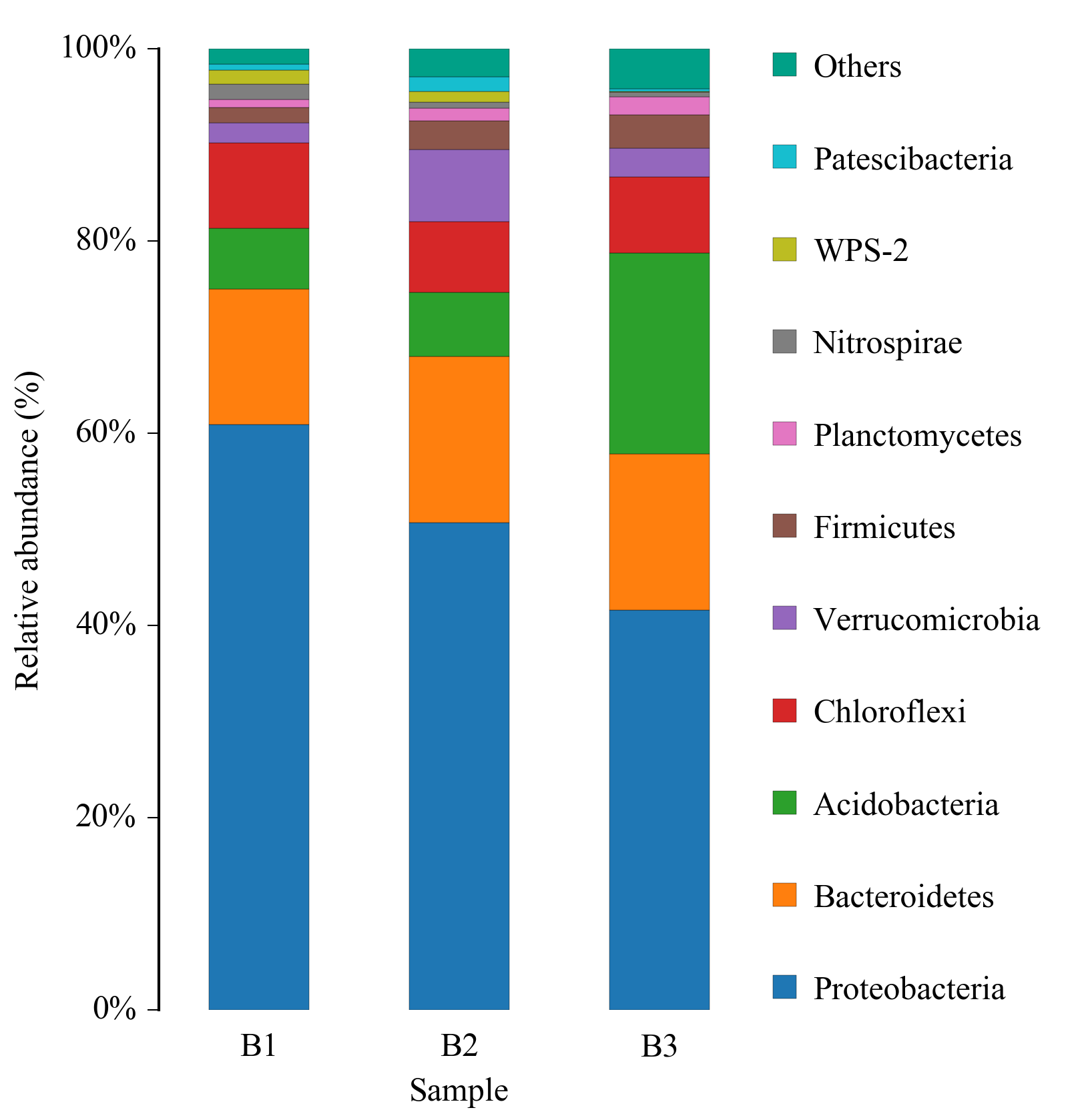

Supplement: S2 Data — (ZIP) [file pone.0261306.s002.zip › customer_backup/taxa_summary/Taxa_dis/treat/sample/treat.sample.phylum.bar.png]

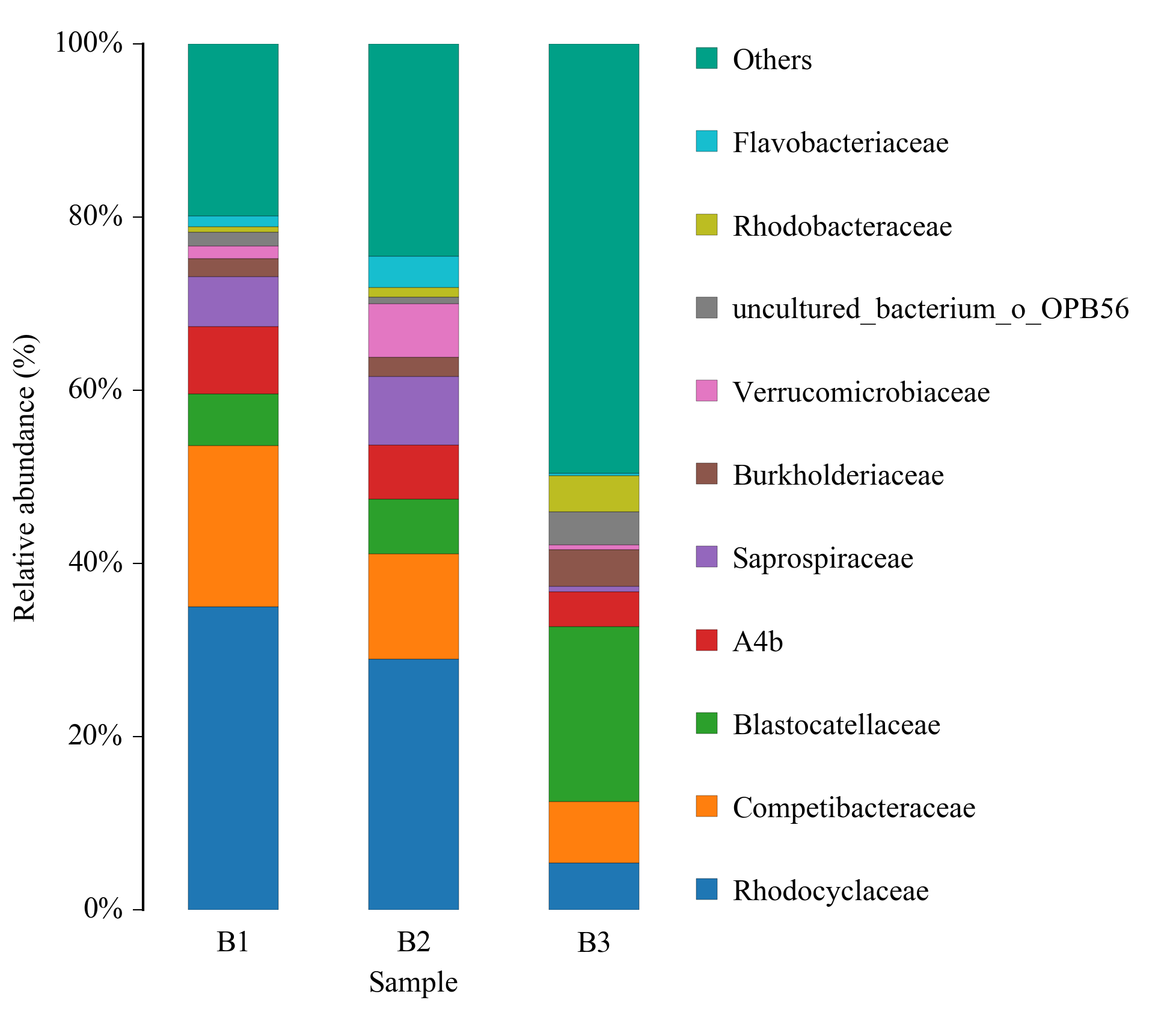

Supplement: S2 Data — (ZIP) [file pone.0261306.s002.zip › customer_backup/taxa_summary/Taxa_dis/treat/sample/treat.sample.family.bar.png]

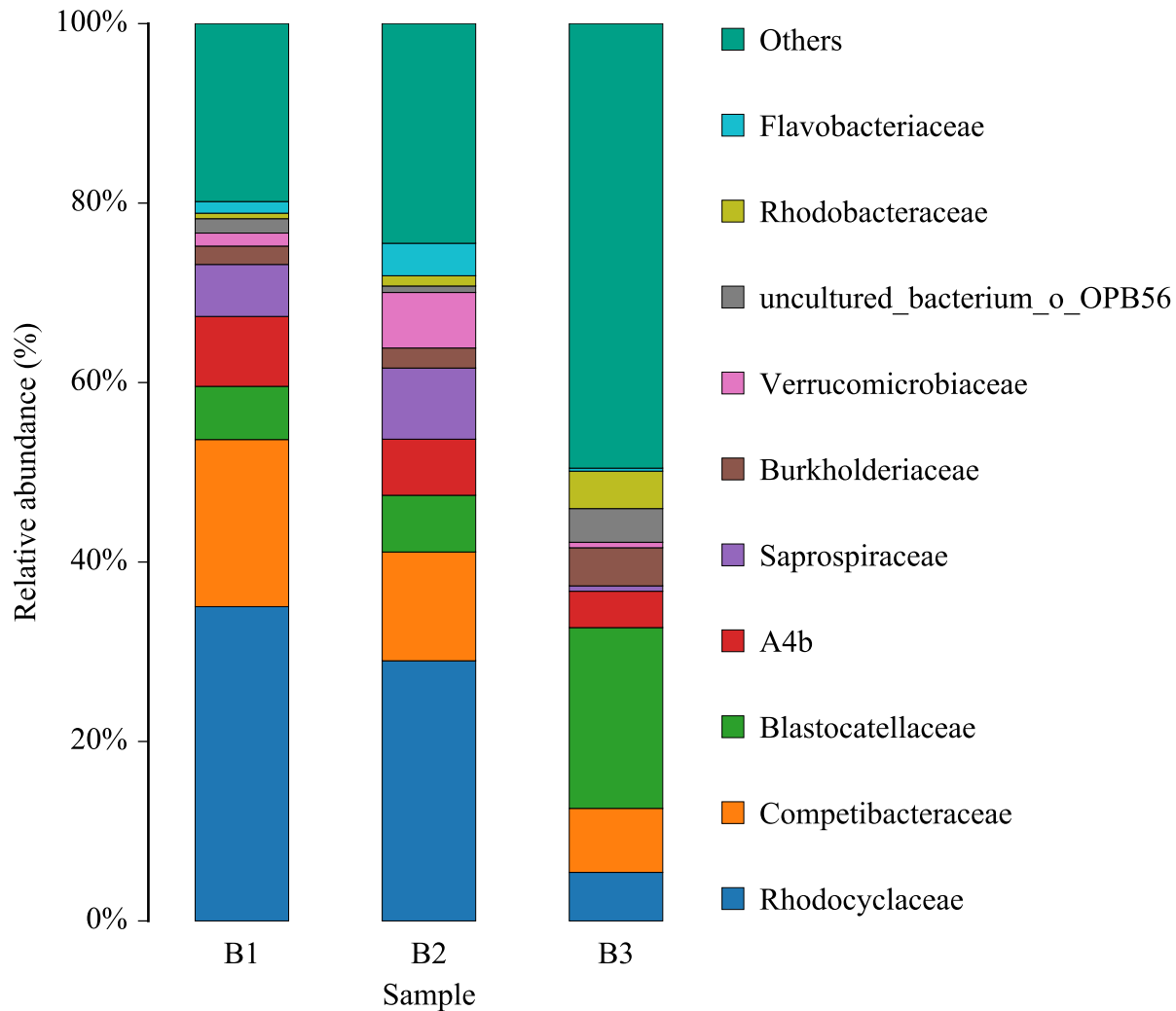

Supplement: S2 Data — (ZIP) [file pone.0261306.s002.zip › customer_backup/taxa_summary/Taxa_dis/treat/sample/treat.sample.family.bar.pdf]

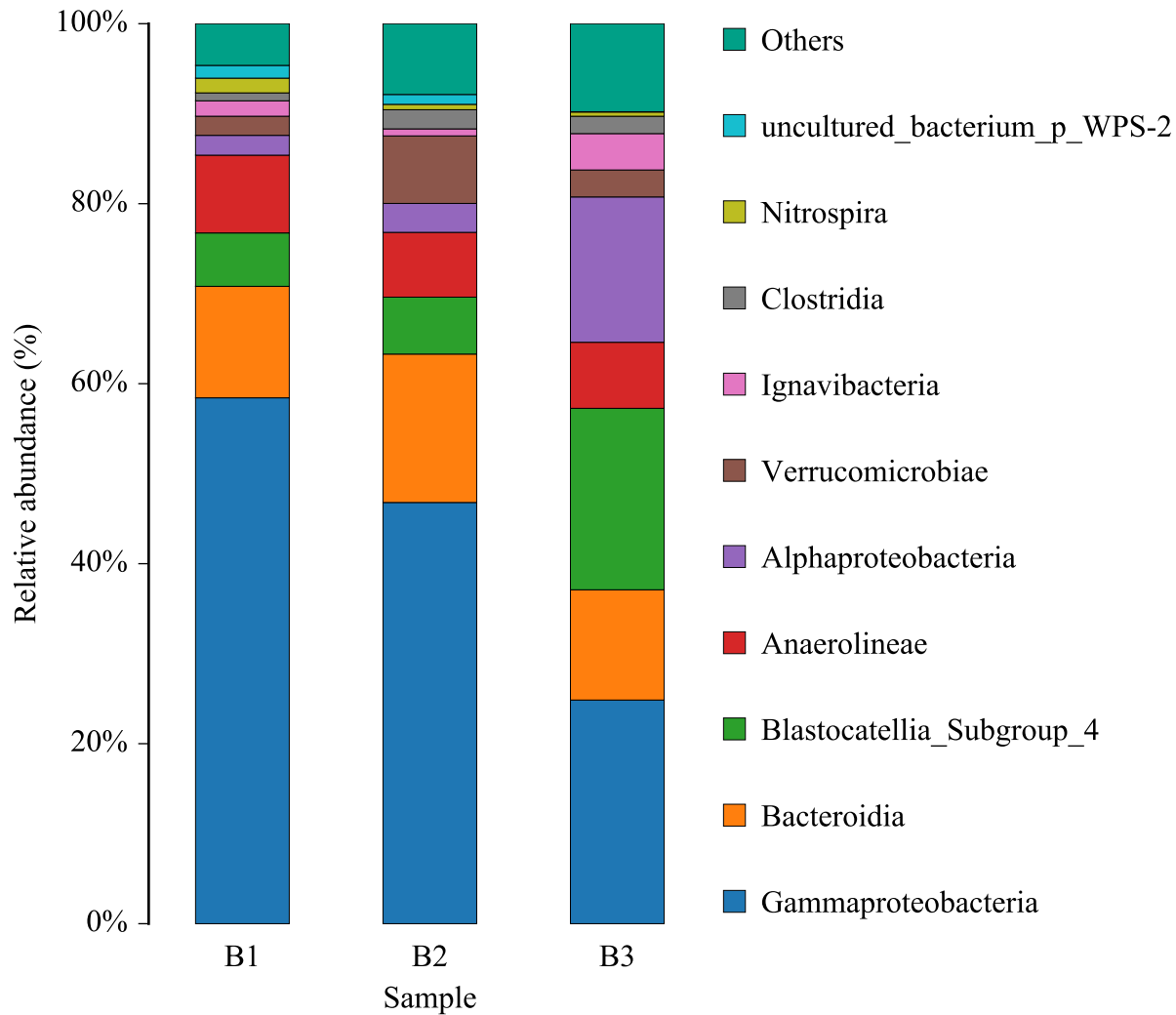

Supplement: S2 Data — (ZIP) [file pone.0261306.s002.zip › customer_backup/taxa_summary/Taxa_dis/treat/sample/treat.sample.class.bar.pdf]

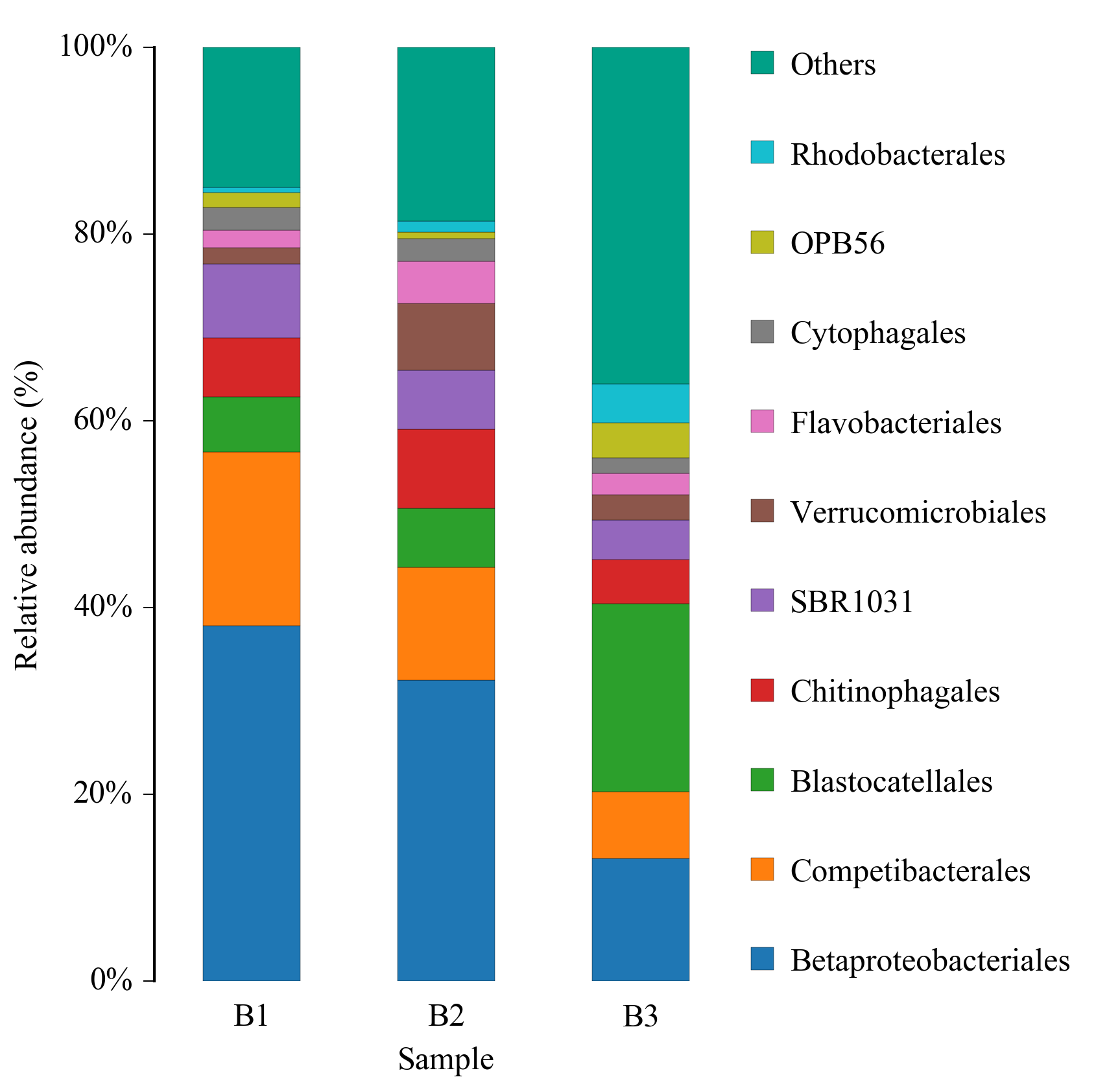

Supplement: S2 Data — (ZIP) [file pone.0261306.s002.zip › customer_backup/taxa_summary/Taxa_dis/treat/sample/treat.sample.order.bar.png]

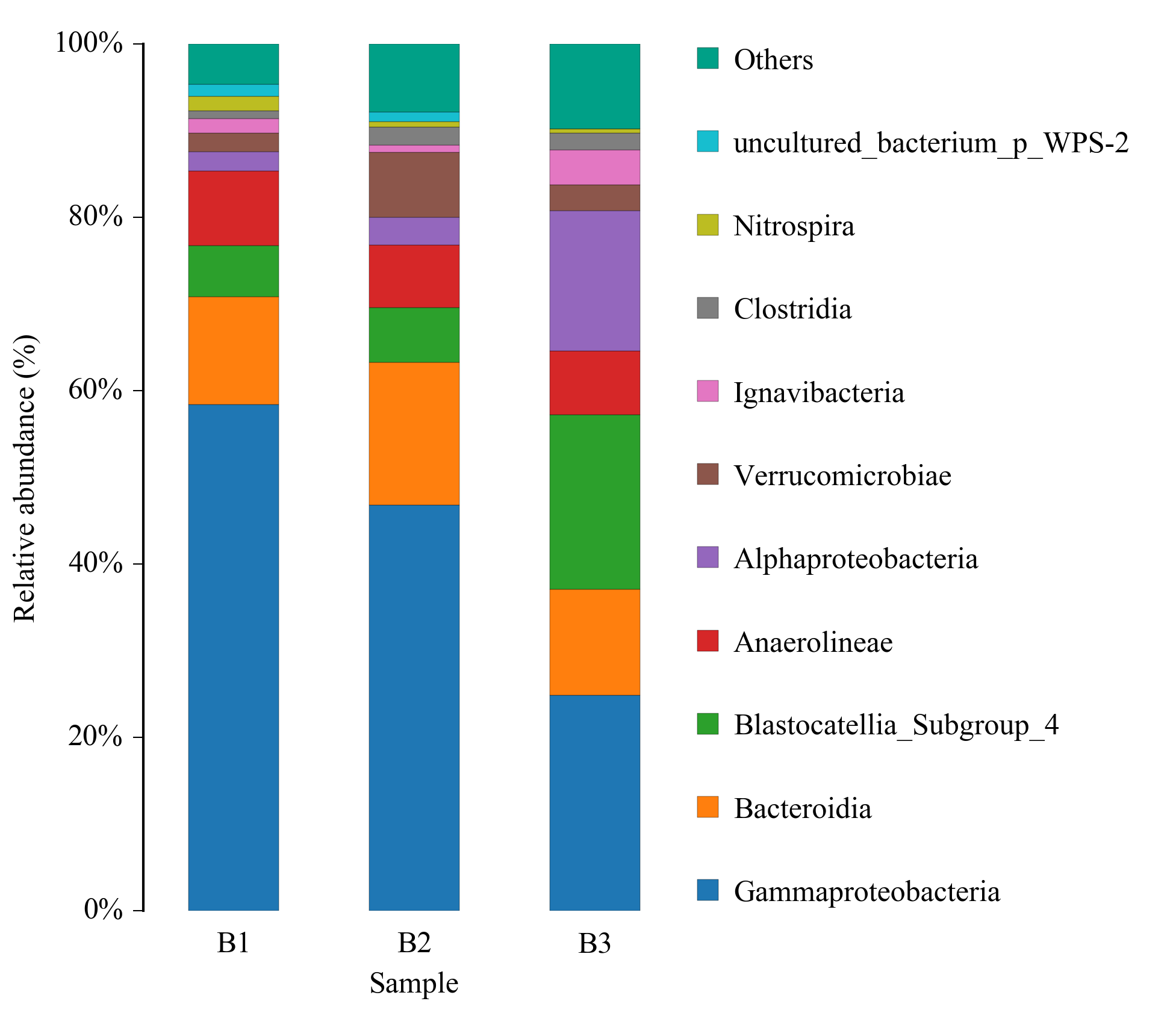

Supplement: S2 Data — (ZIP) [file pone.0261306.s002.zip › customer_backup/taxa_summary/Taxa_dis/treat/sample/treat.sample.class.bar.png]

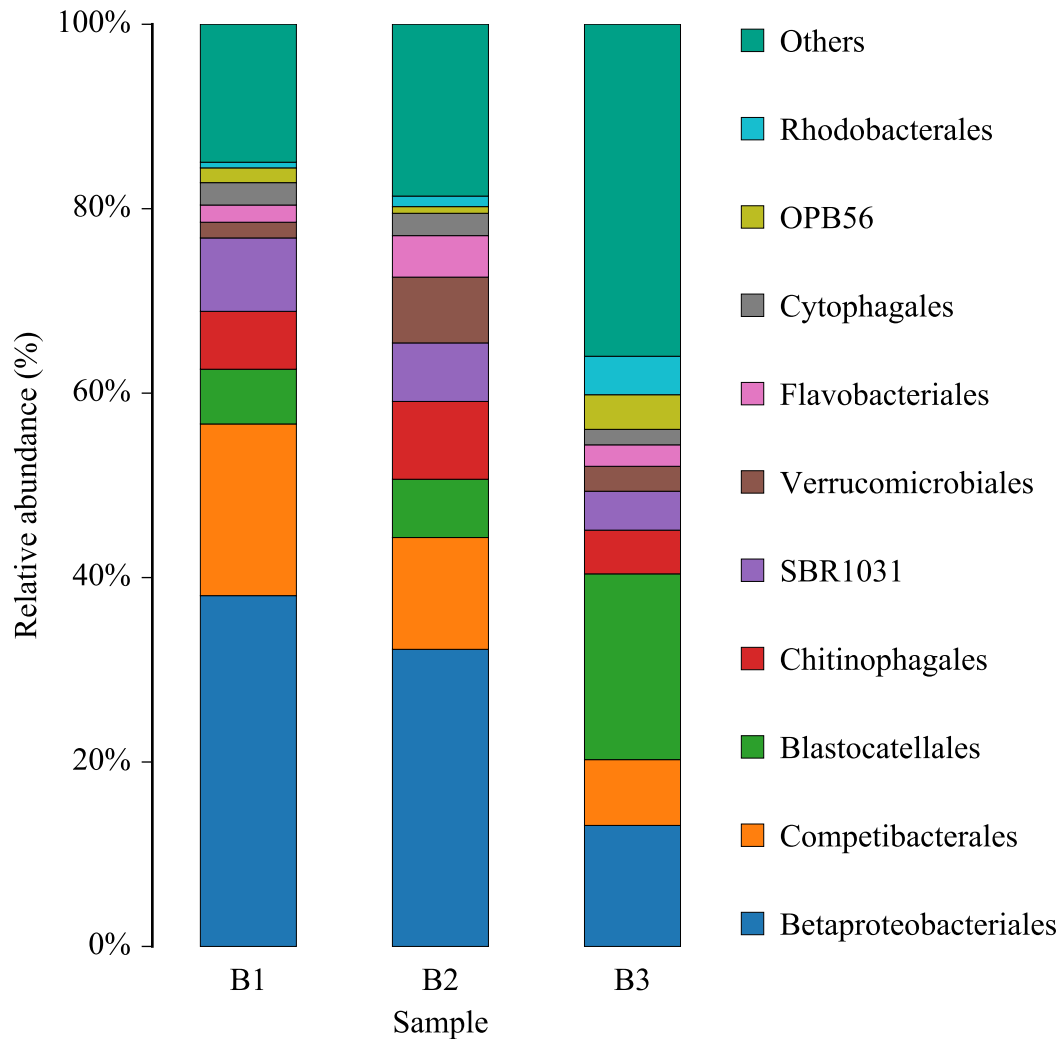

Supplement: S2 Data — (ZIP) [file pone.0261306.s002.zip › customer_backup/taxa_summary/Taxa_dis/treat/sample/treat.sample.order.bar.pdf]

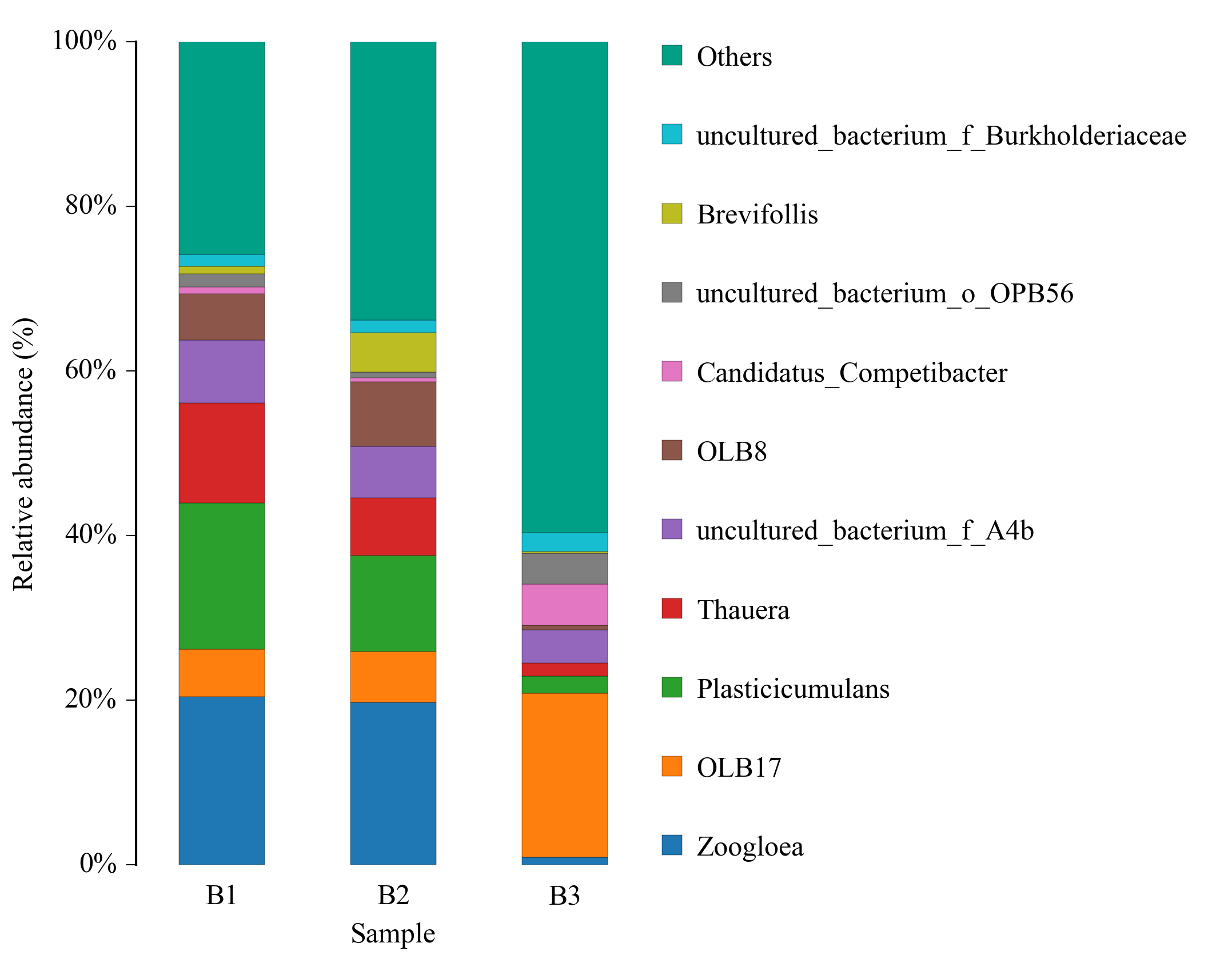

Supplement: S2 Data — (ZIP) [file pone.0261306.s002.zip › customer_backup/taxa_summary/Taxa_dis/treat/sample/treat.sample.genus.bar.png]

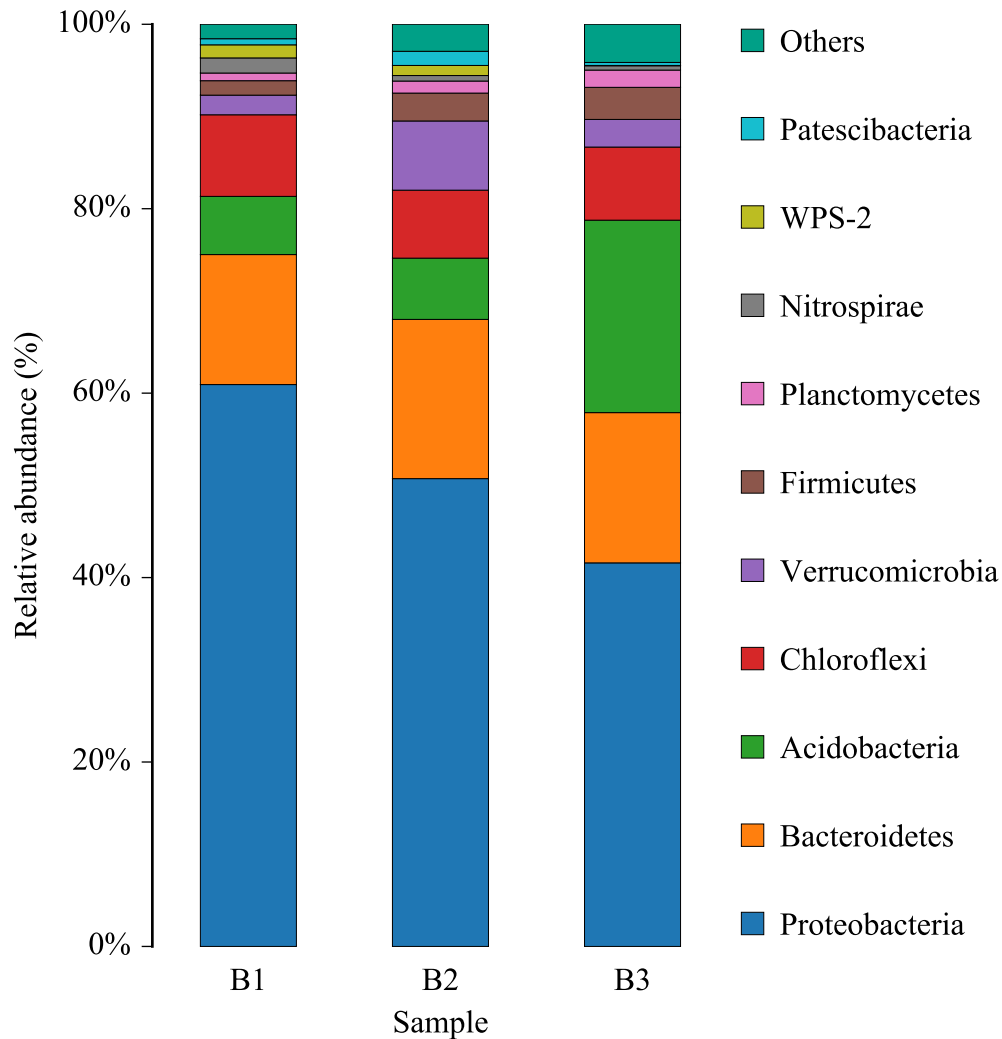

Supplement: S2 Data — (ZIP) [file pone.0261306.s002.zip › customer_backup/taxa_summary/Taxa_dis/treat/sample/treat.sample.phylum.bar.pdf]

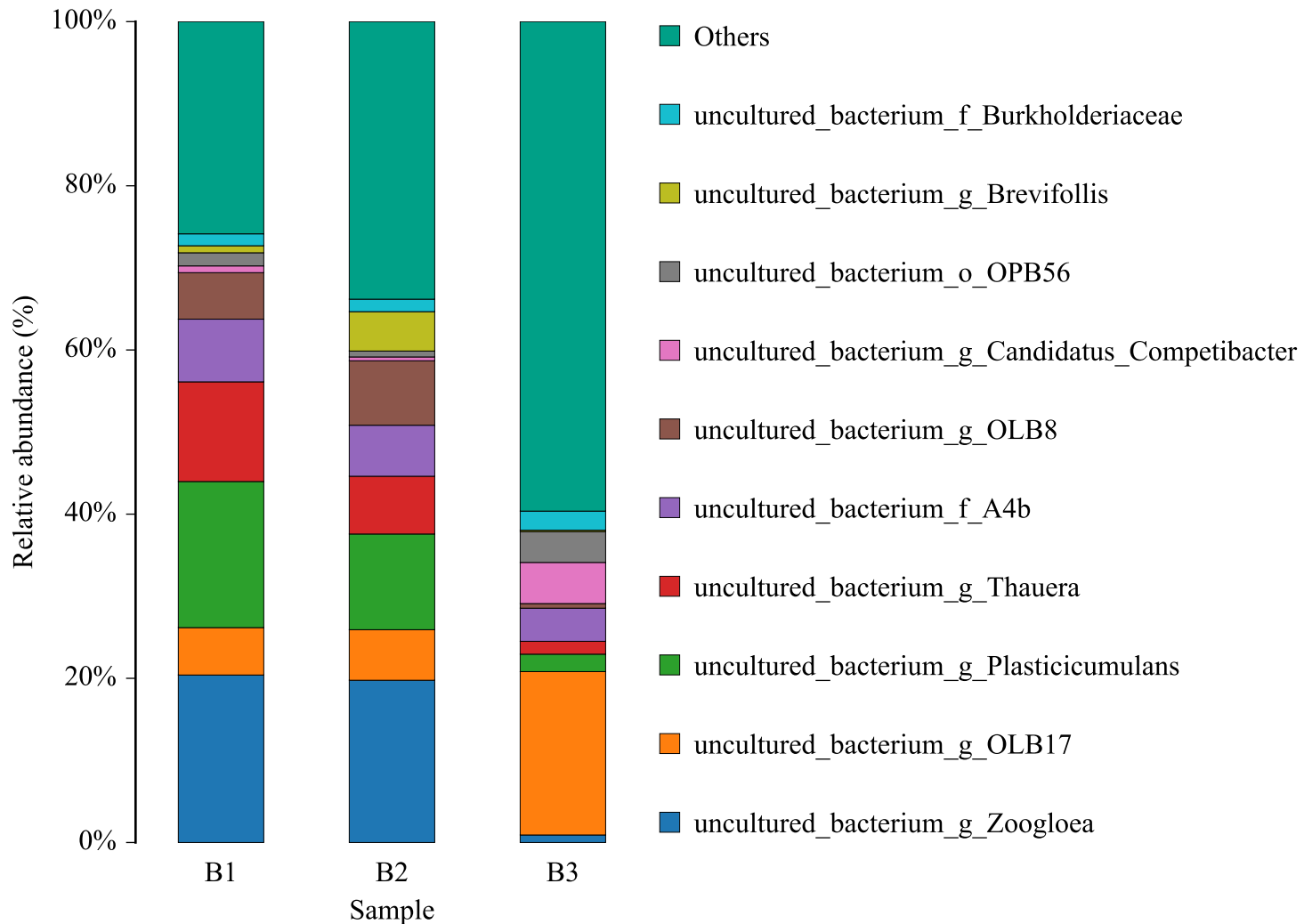

Supplement: S2 Data — (ZIP) [file pone.0261306.s002.zip › customer_backup/taxa_summary/Taxa_dis/treat/sample/treat.sample.species.bar.pdf]

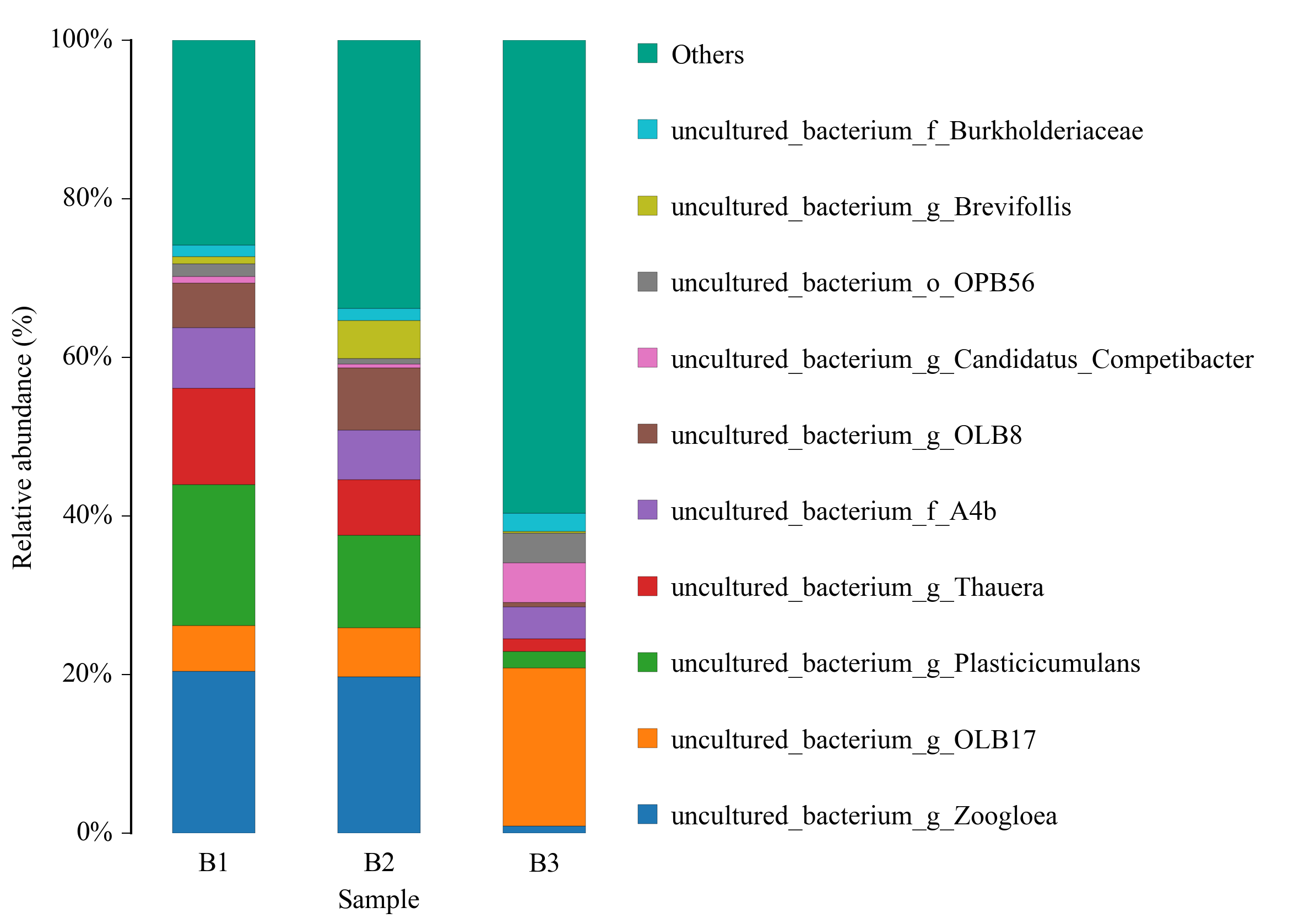

Supplement: S2 Data — (ZIP) [file pone.0261306.s002.zip › customer_backup/taxa_summary/Taxa_dis/treat/sample/treat.sample.species.bar.png]

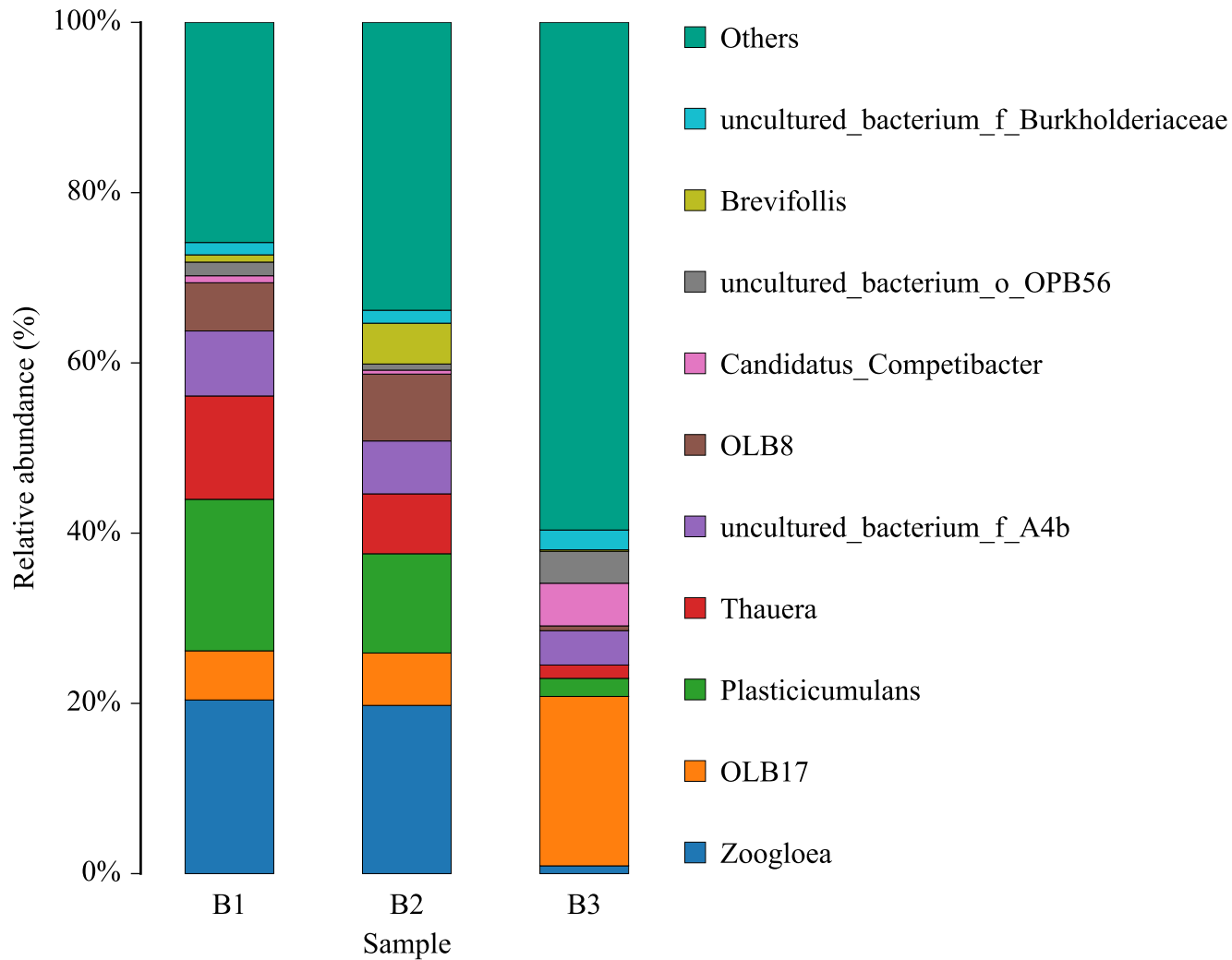

Supplement: S2 Data — (ZIP) [file pone.0261306.s002.zip › customer_backup/taxa_summary/Taxa_dis/treat/sample/treat.sample.genus.bar.pdf]

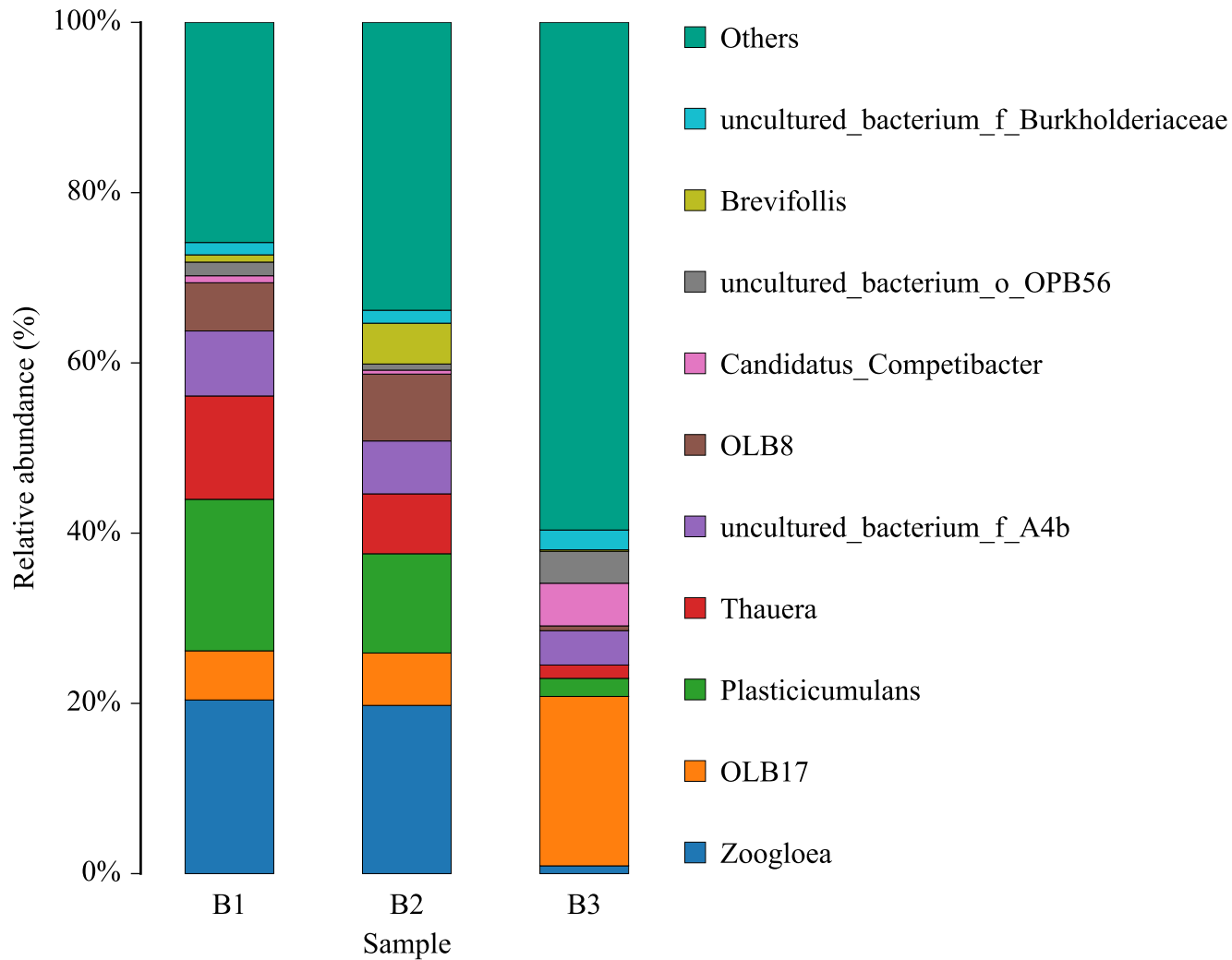

Supplement: S2 Data — (ZIP) [file pone.0261306.s002.zip › customer_backup/taxa_summary/Taxa_dis/allsample/sample/allsample.sample.genus.bar.pdf]

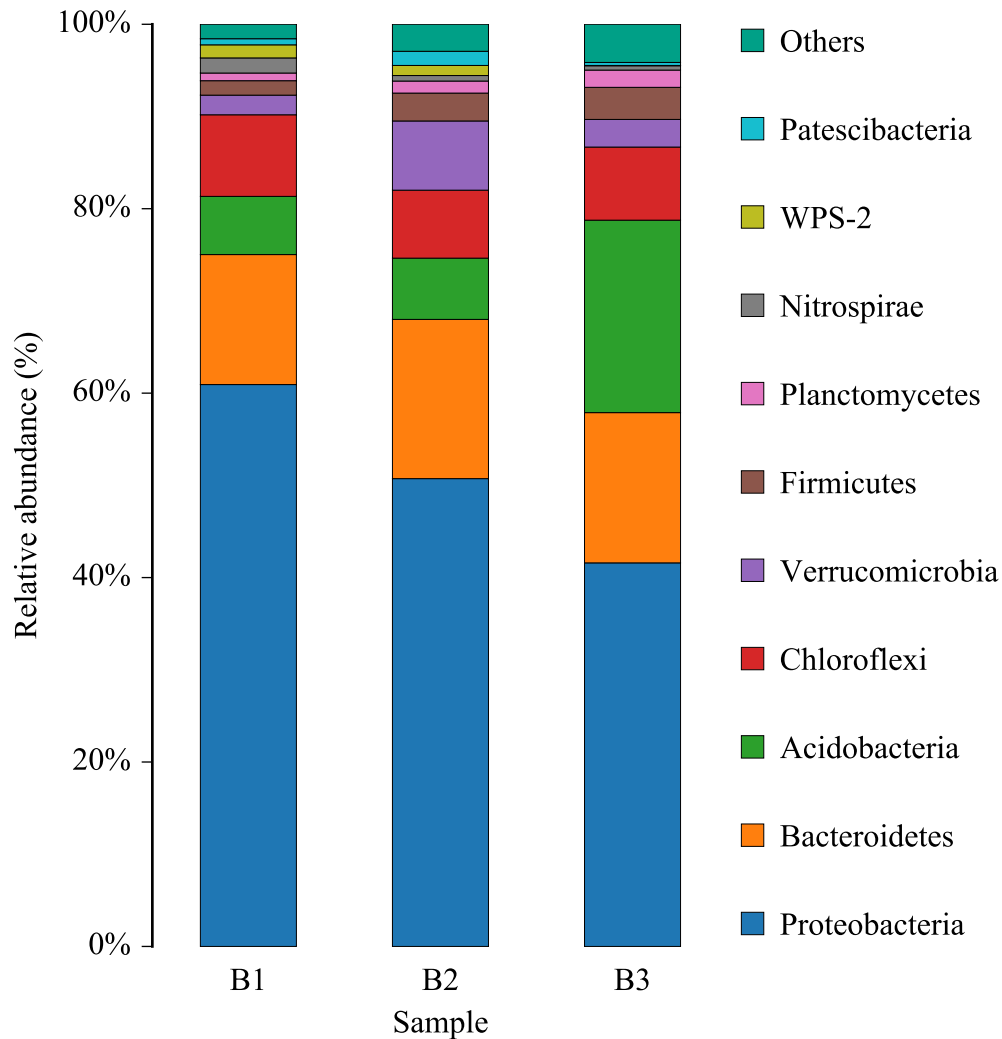

Supplement: S2 Data — (ZIP) [file pone.0261306.s002.zip › customer_backup/taxa_summary/Taxa_dis/allsample/sample/allsample.sample.phylum.bar.pdf]

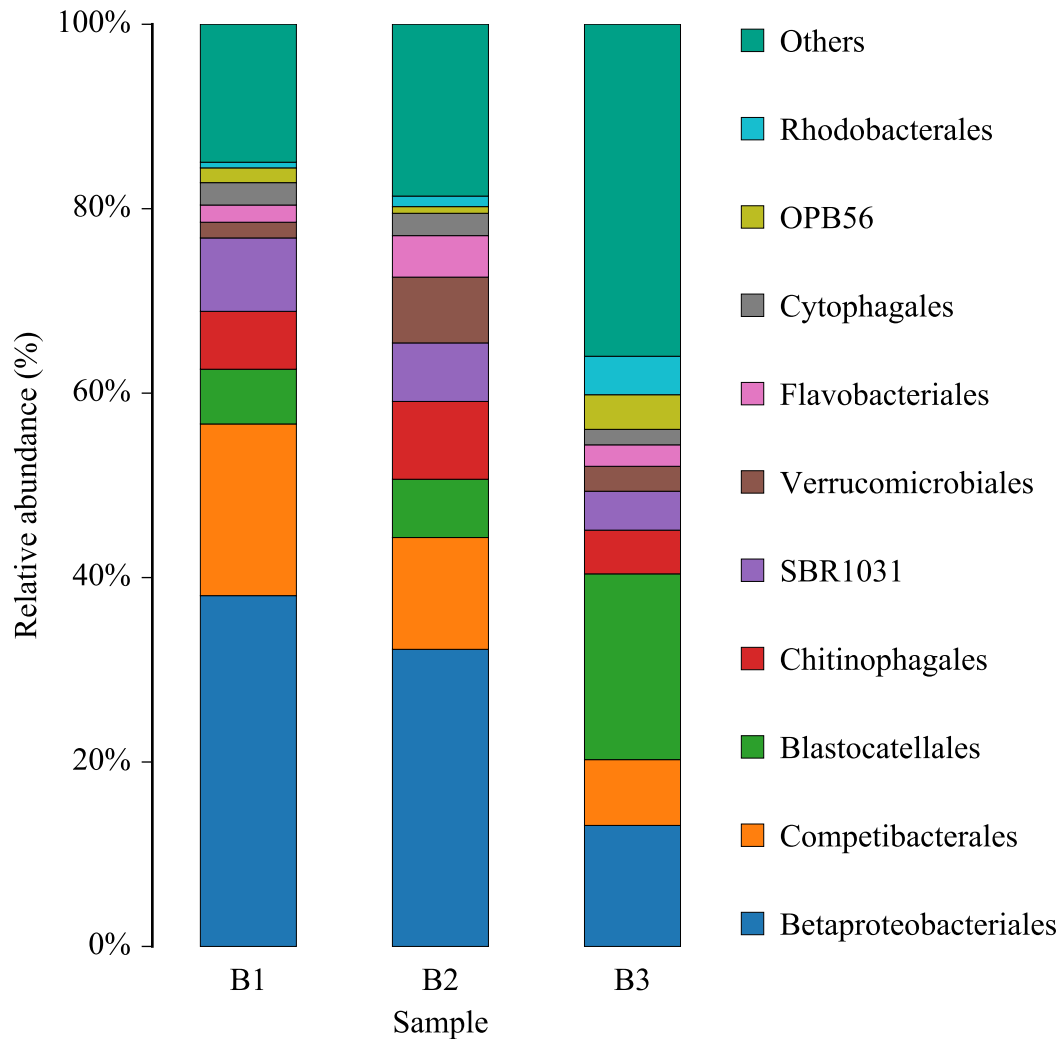

Supplement: S2 Data — (ZIP) [file pone.0261306.s002.zip › customer_backup/taxa_summary/Taxa_dis/allsample/sample/allsample.sample.order.bar.pdf]

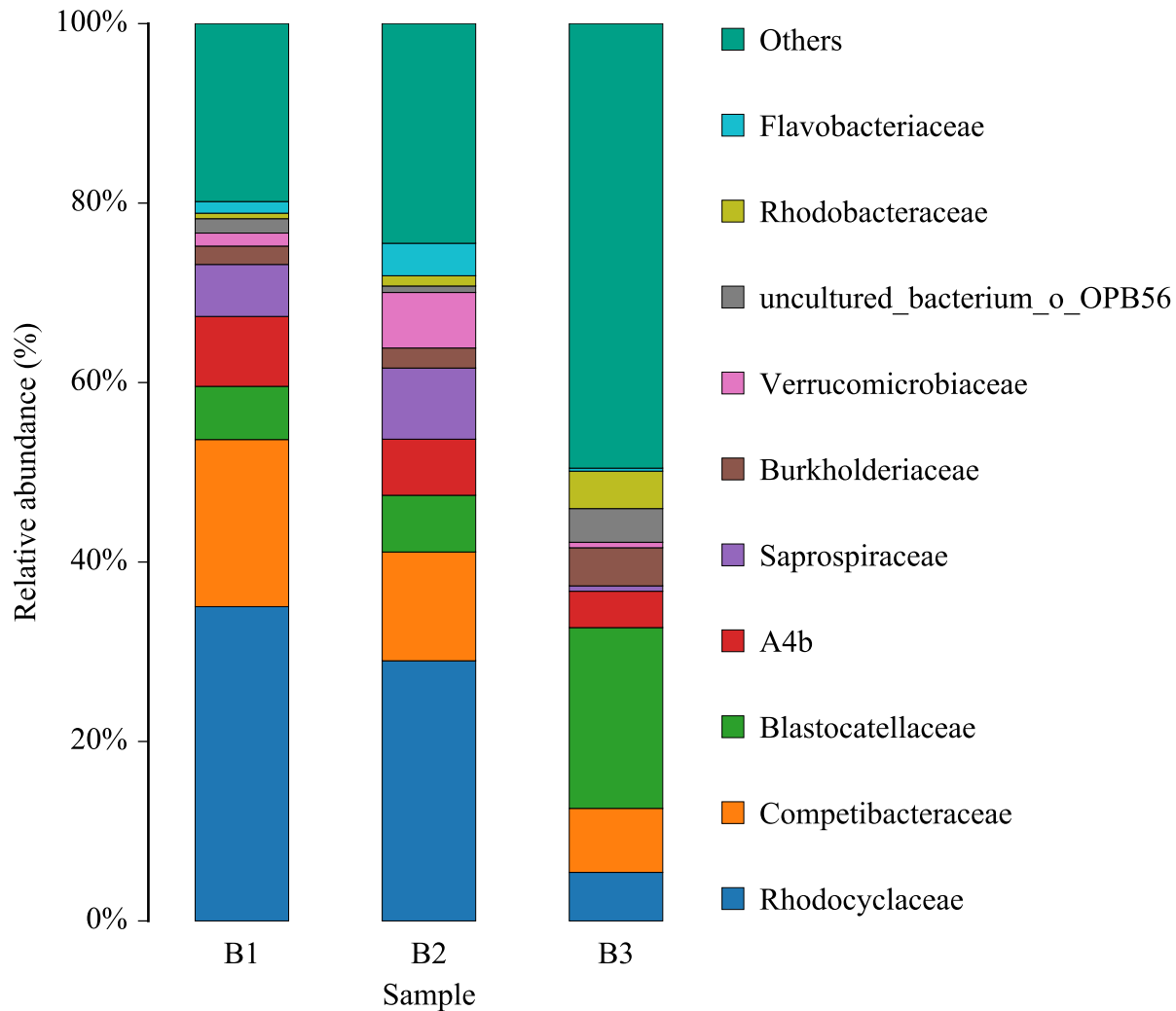

Supplement: S2 Data — (ZIP) [file pone.0261306.s002.zip › customer_backup/taxa_summary/Taxa_dis/allsample/sample/allsample.sample.family.bar.pdf]

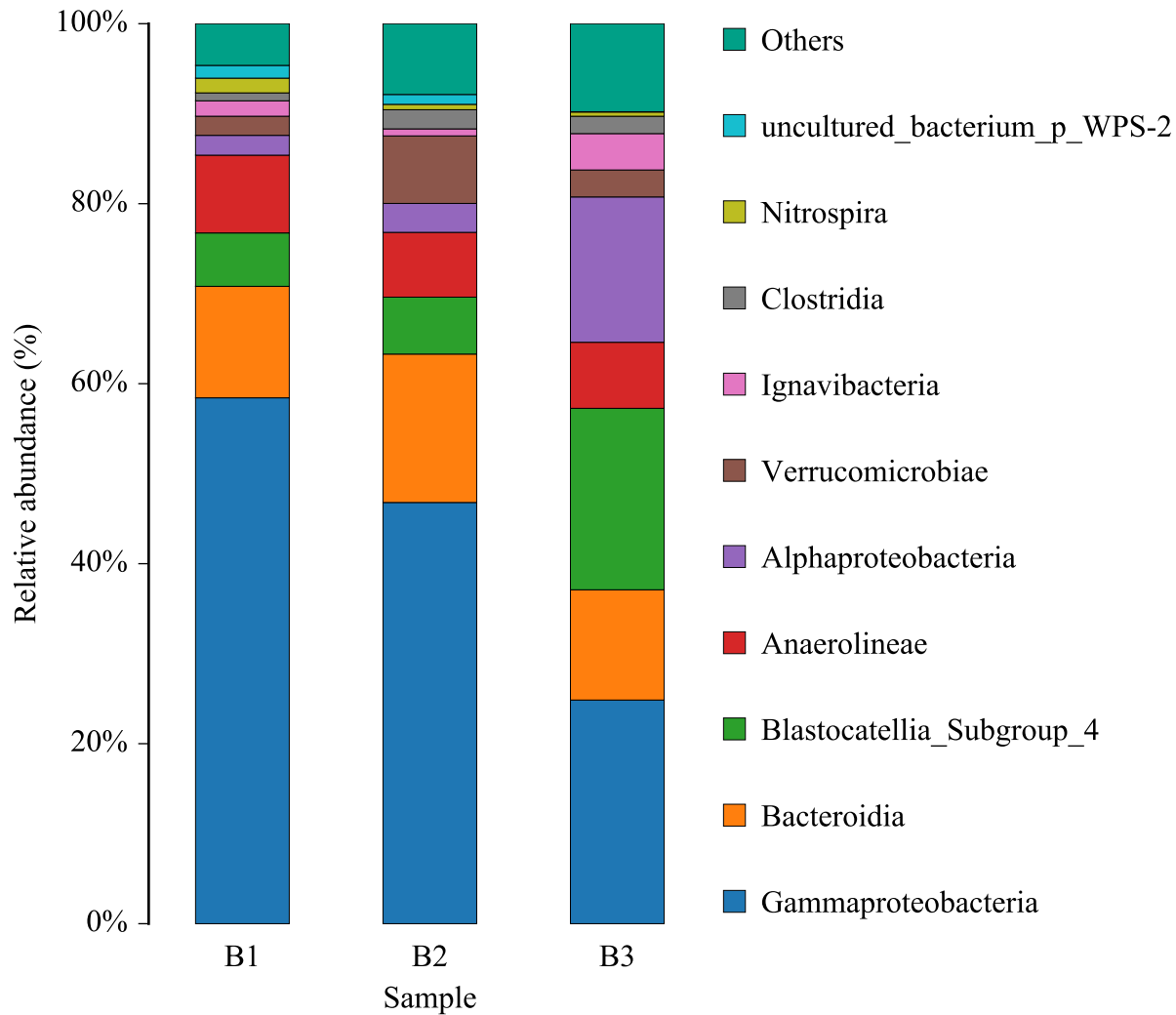

Supplement: S2 Data — (ZIP) [file pone.0261306.s002.zip › customer_backup/taxa_summary/Taxa_dis/allsample/sample/allsample.sample.class.bar.pdf]

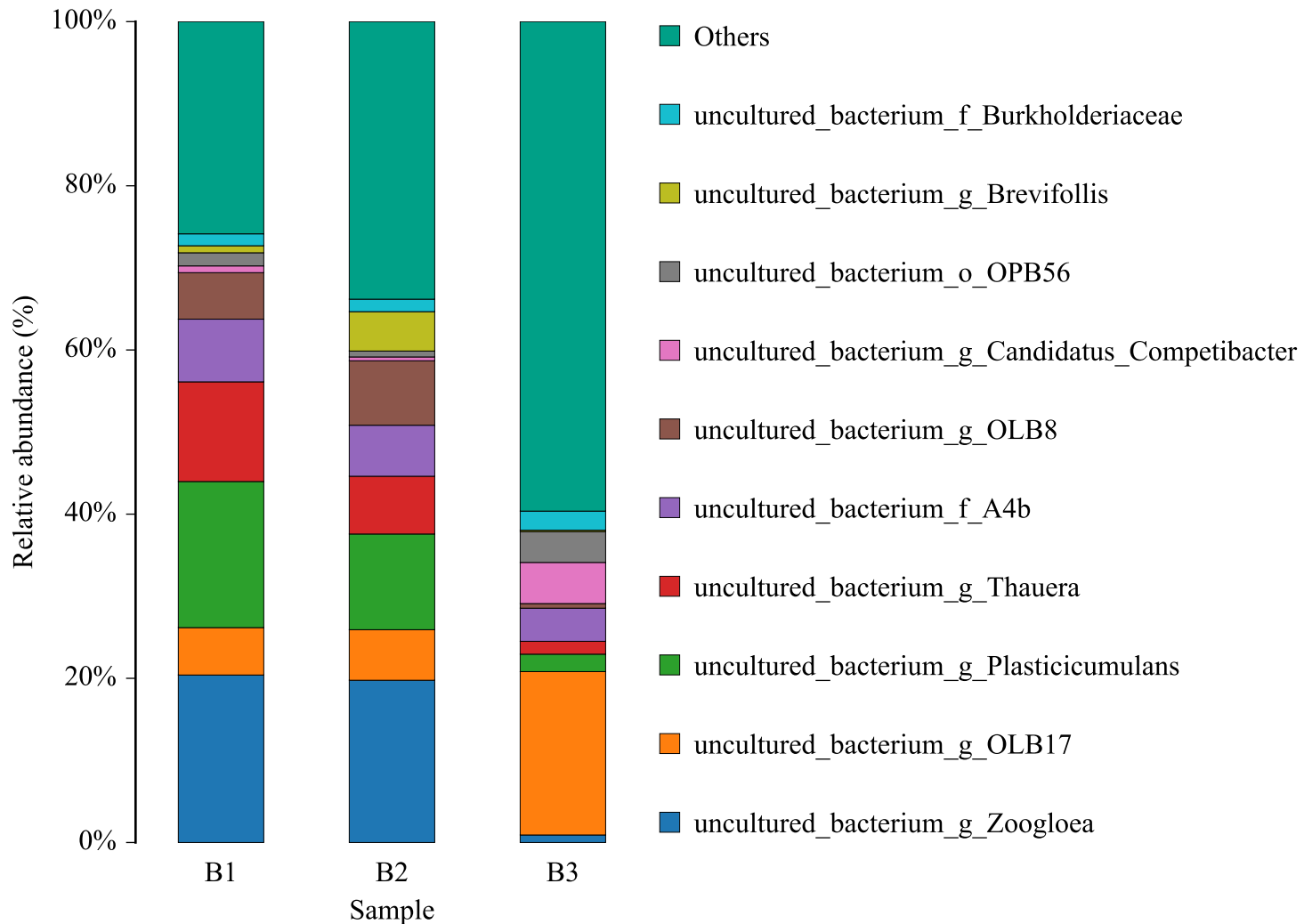

Supplement: S2 Data — (ZIP) [file pone.0261306.s002.zip › customer_backup/taxa_summary/Taxa_dis/allsample/sample/allsample.sample.species.bar.pdf]

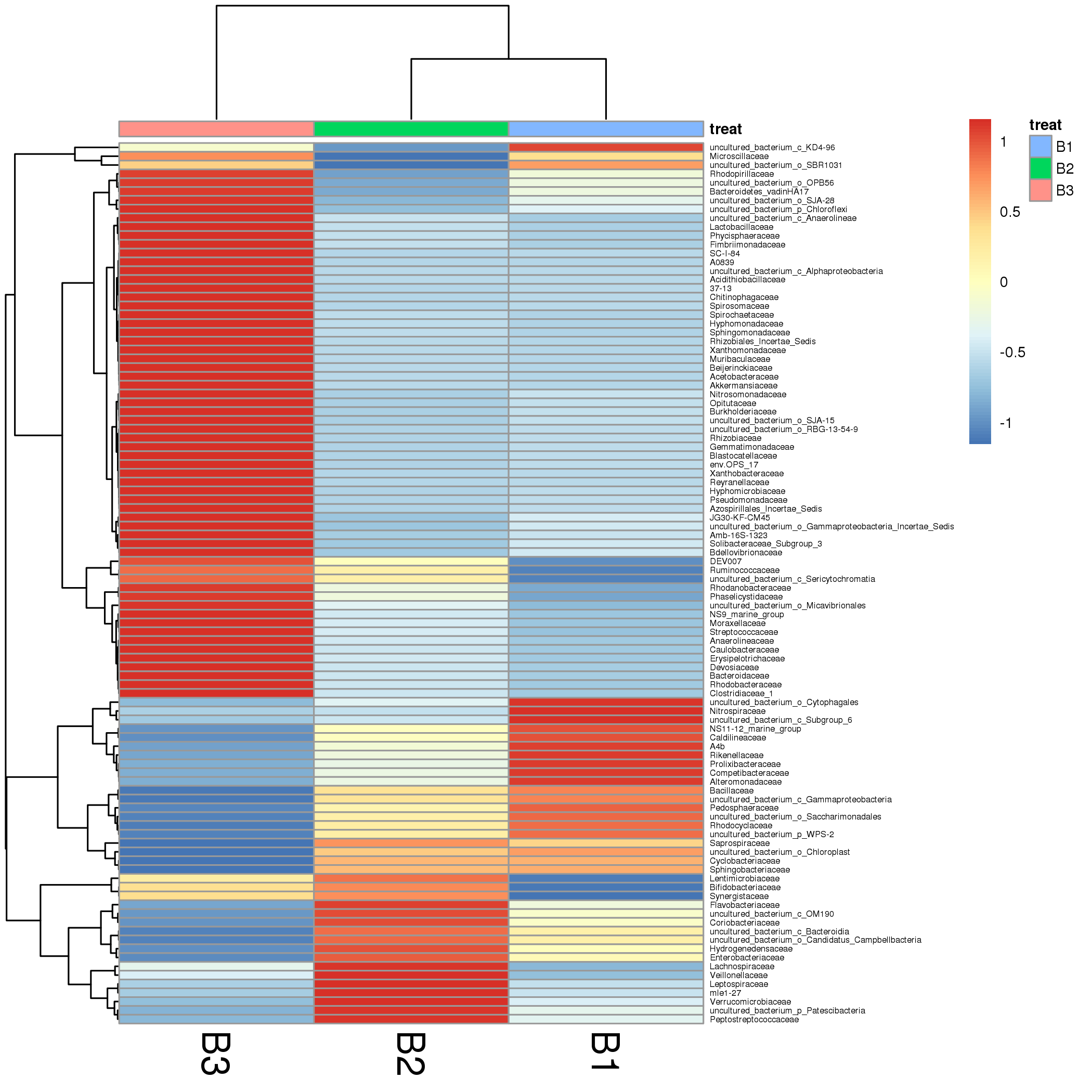

Supplement: S2 Data — (ZIP) [file pone.0261306.s002.zip › customer_backup/taxa_summary/heatmap/treat/treat.family.reabundance.heatmap.png]

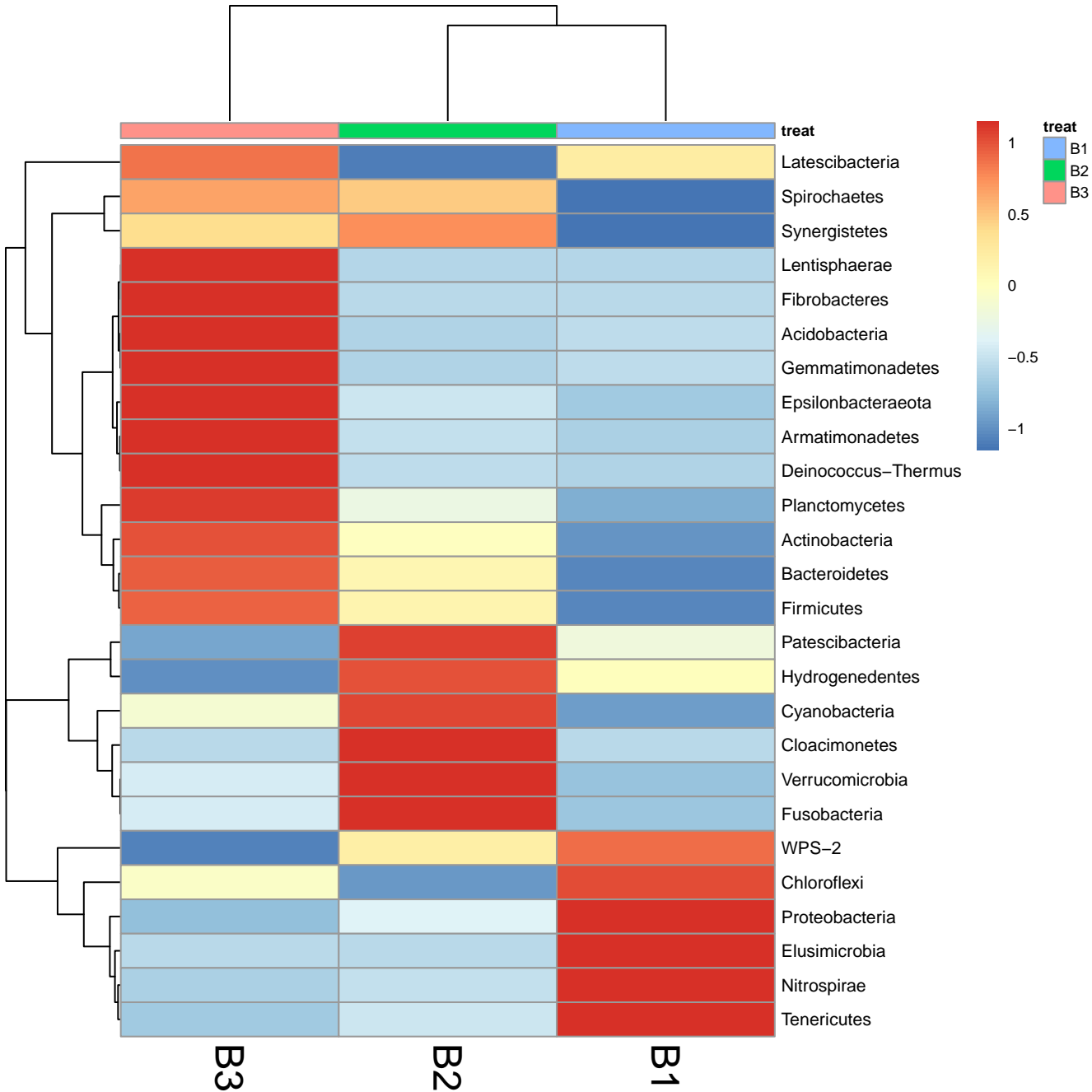

Supplement: S2 Data — (ZIP) [file pone.0261306.s002.zip › customer_backup/taxa_summary/heatmap/treat/treat.phylum.reabundance.heatmap.pdf]

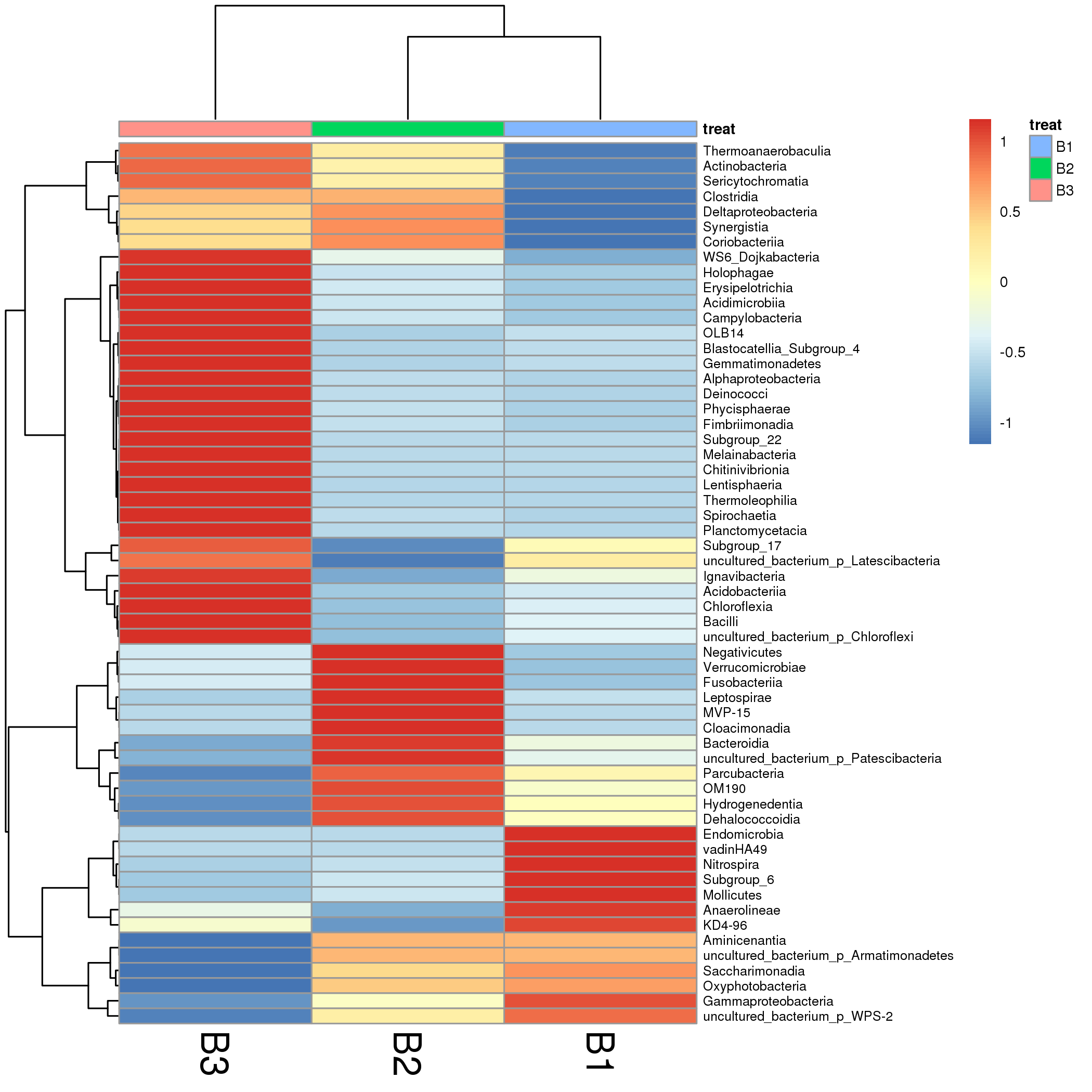

Supplement: S2 Data — (ZIP) [file pone.0261306.s002.zip › customer_backup/taxa_summary/heatmap/treat/treat.class.reabundance.heatmap.png]

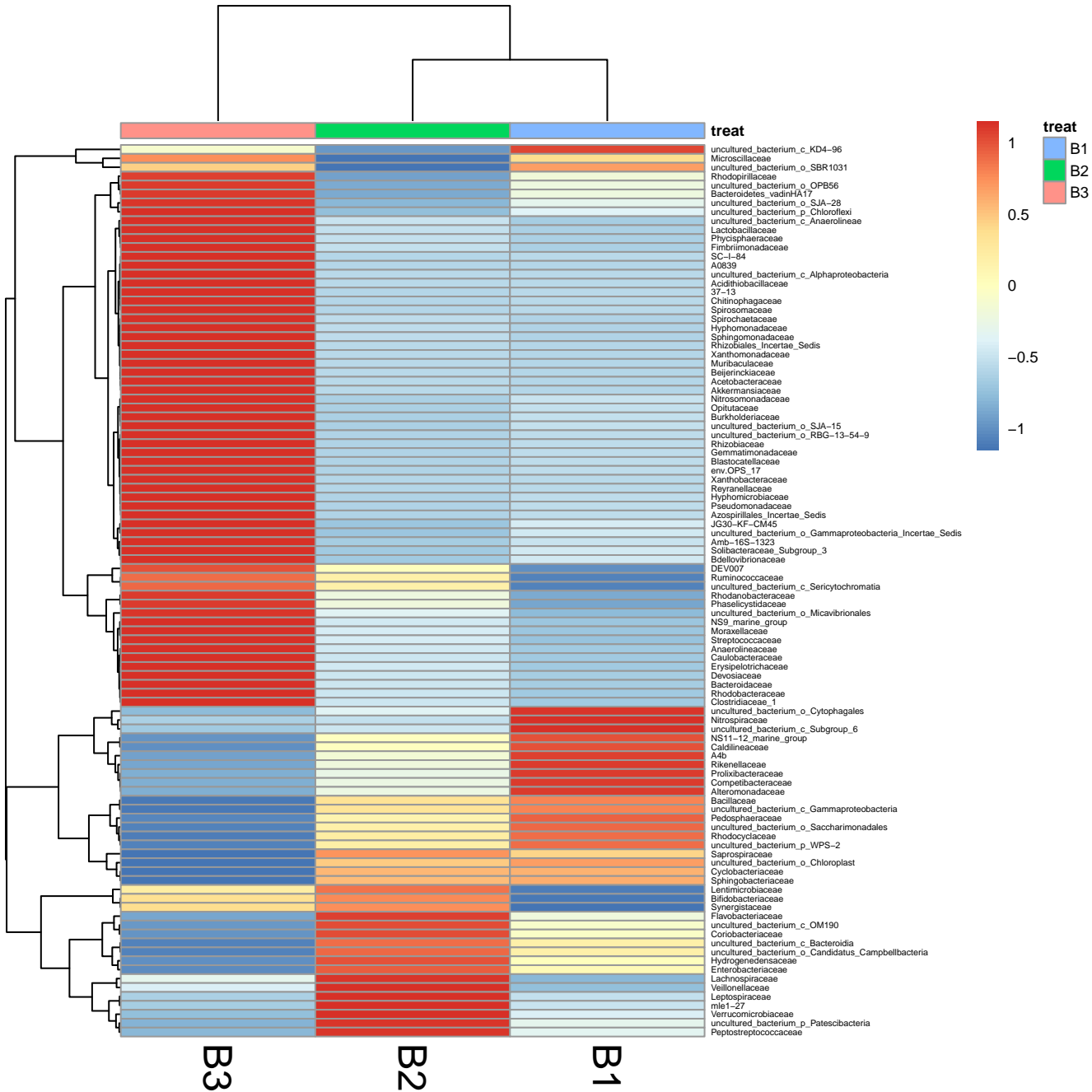

Supplement: S2 Data — (ZIP) [file pone.0261306.s002.zip › customer_backup/taxa_summary/heatmap/treat/treat.family.reabundance.heatmap.pdf]

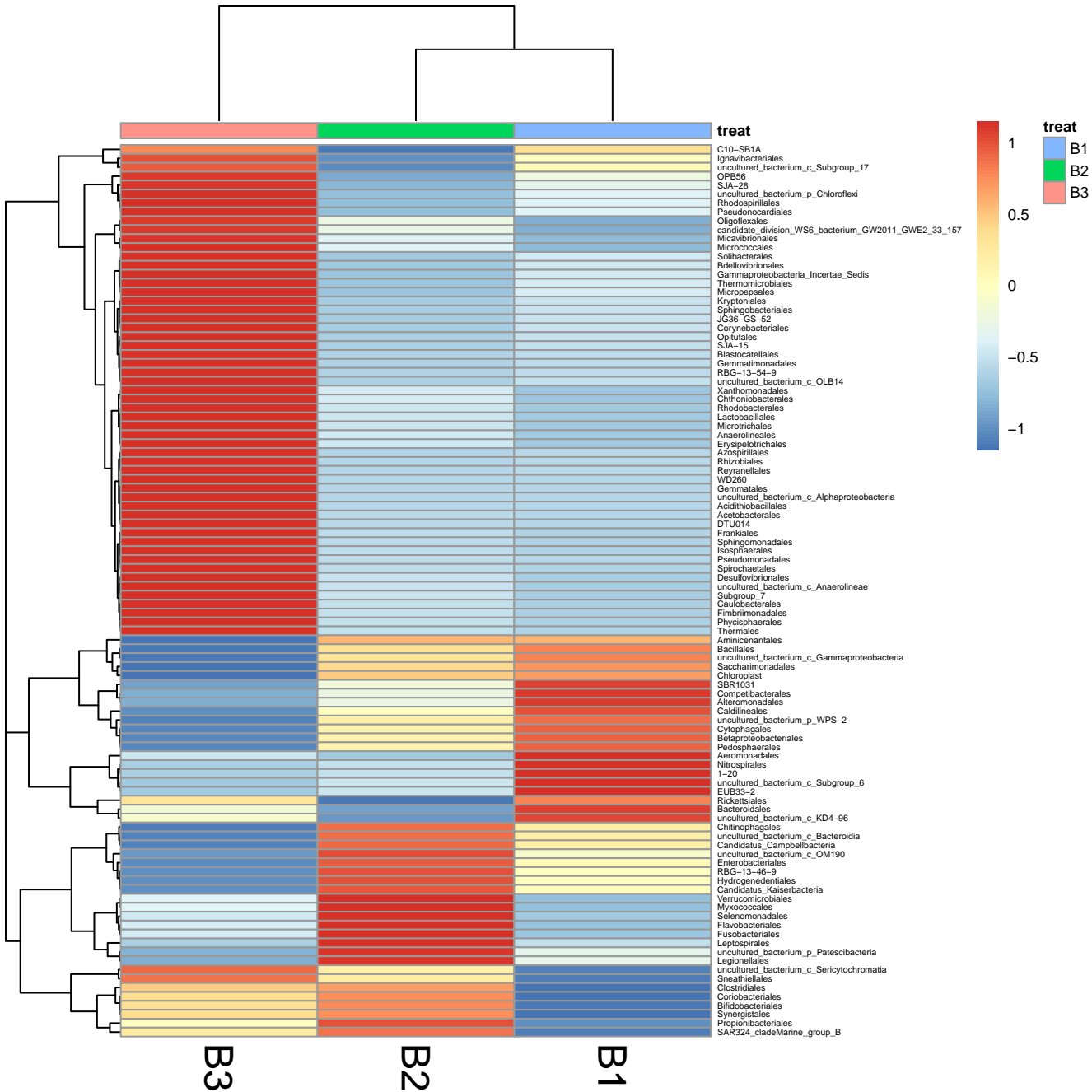

Supplement: S2 Data — (ZIP) [file pone.0261306.s002.zip › customer_backup/taxa_summary/heatmap/treat/treat.order.reabundance.heatmap.pdf]

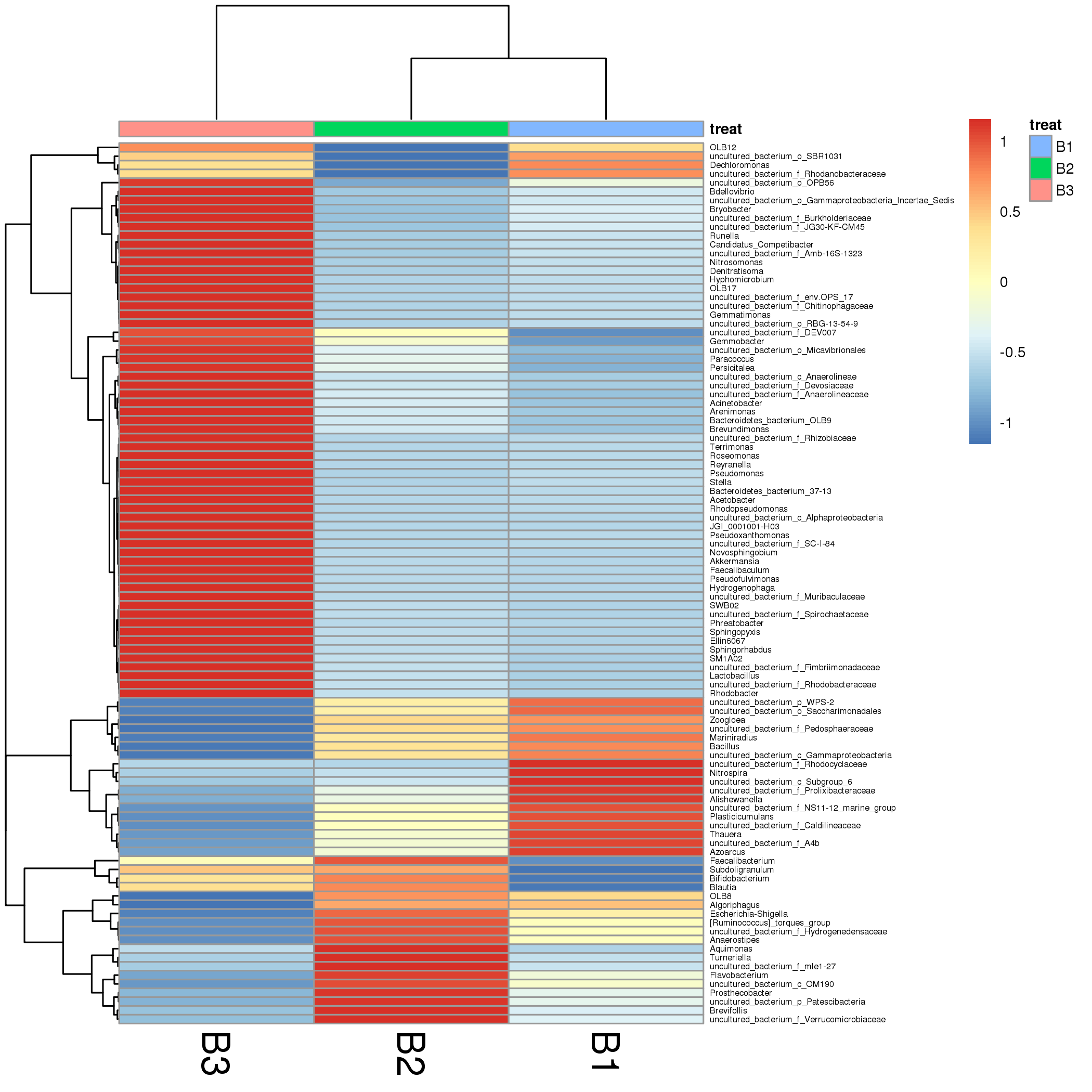

Supplement: S2 Data — (ZIP) [file pone.0261306.s002.zip › customer_backup/taxa_summary/heatmap/treat/treat.genus.reabundance.heatmap.png]

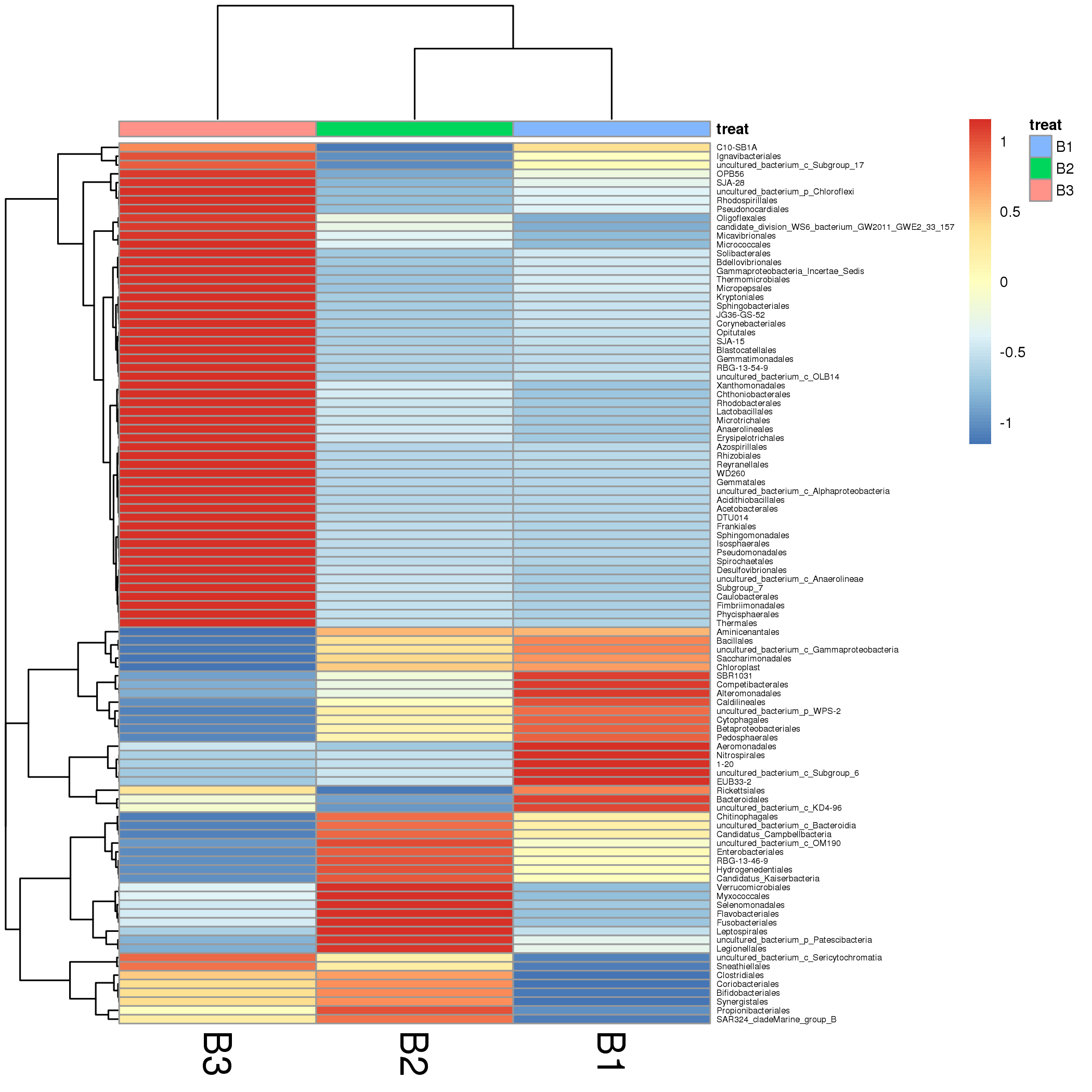

Supplement: S2 Data — (ZIP) [file pone.0261306.s002.zip › customer_backup/taxa_summary/heatmap/treat/treat.order.reabundance.heatmap.png]

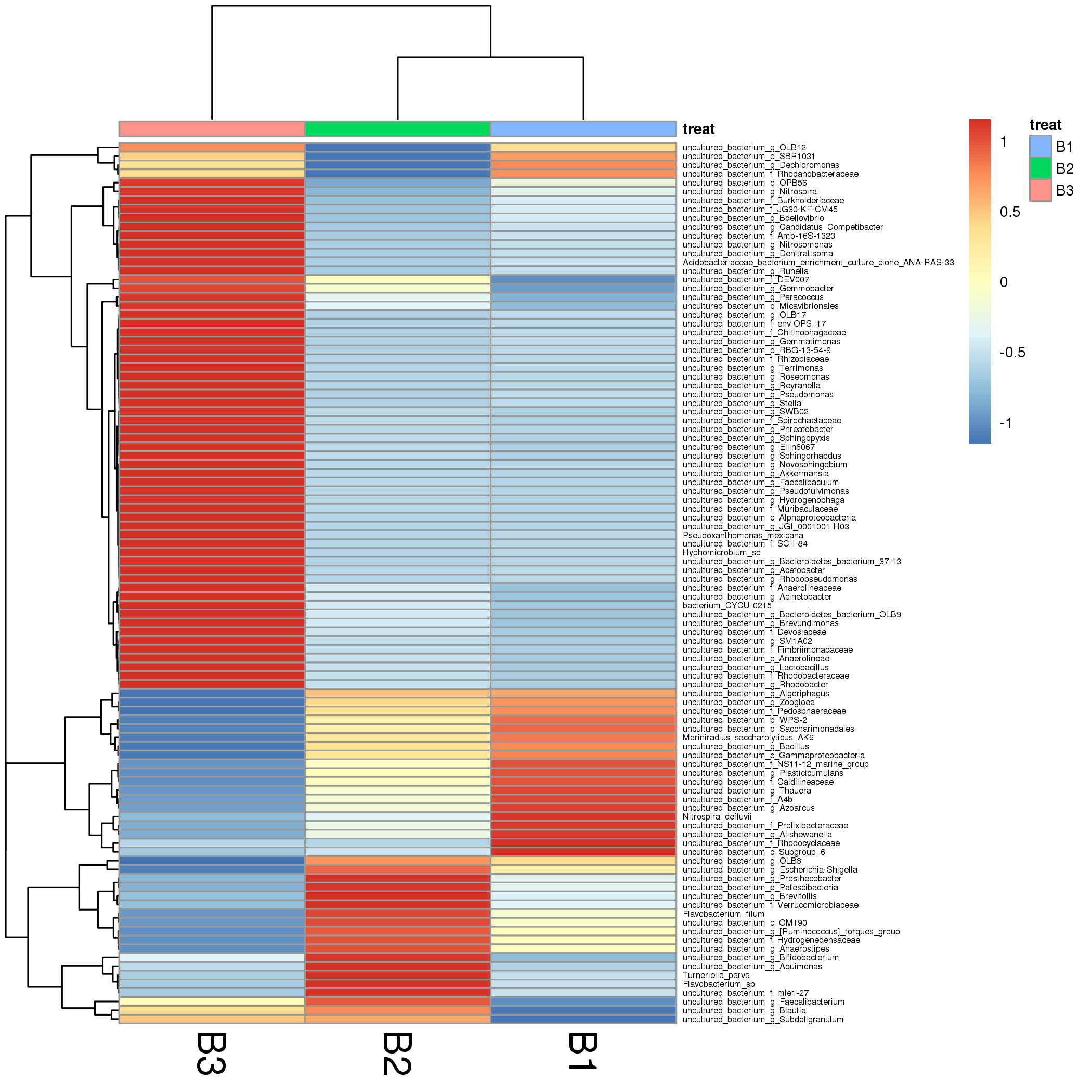

Supplement: S2 Data — (ZIP) [file pone.0261306.s002.zip › customer_backup/taxa_summary/heatmap/treat/treat.species.reabundance.heatmap.png]

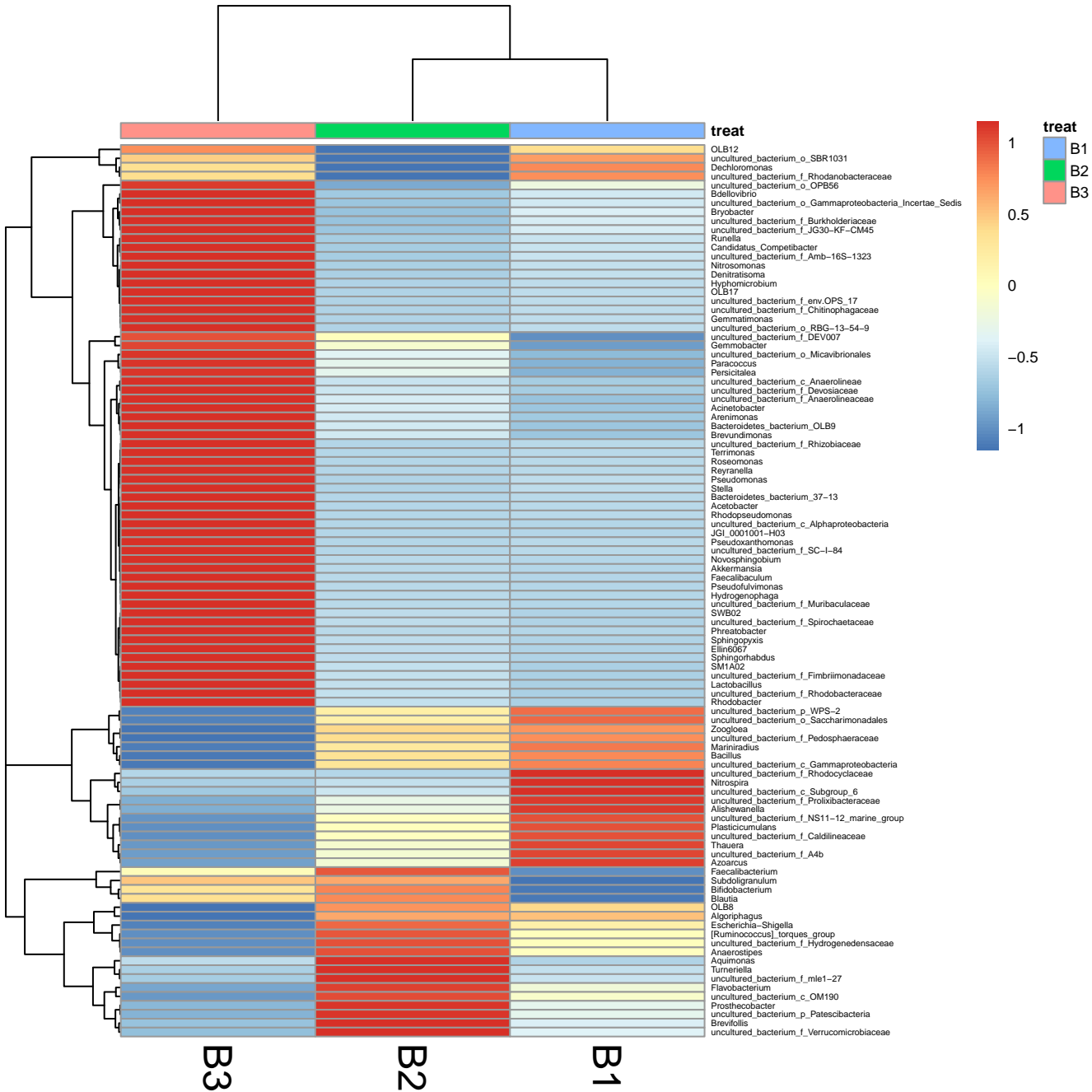

Supplement: S2 Data — (ZIP) [file pone.0261306.s002.zip › customer_backup/taxa_summary/heatmap/treat/treat.genus.reabundance.heatmap.pdf]

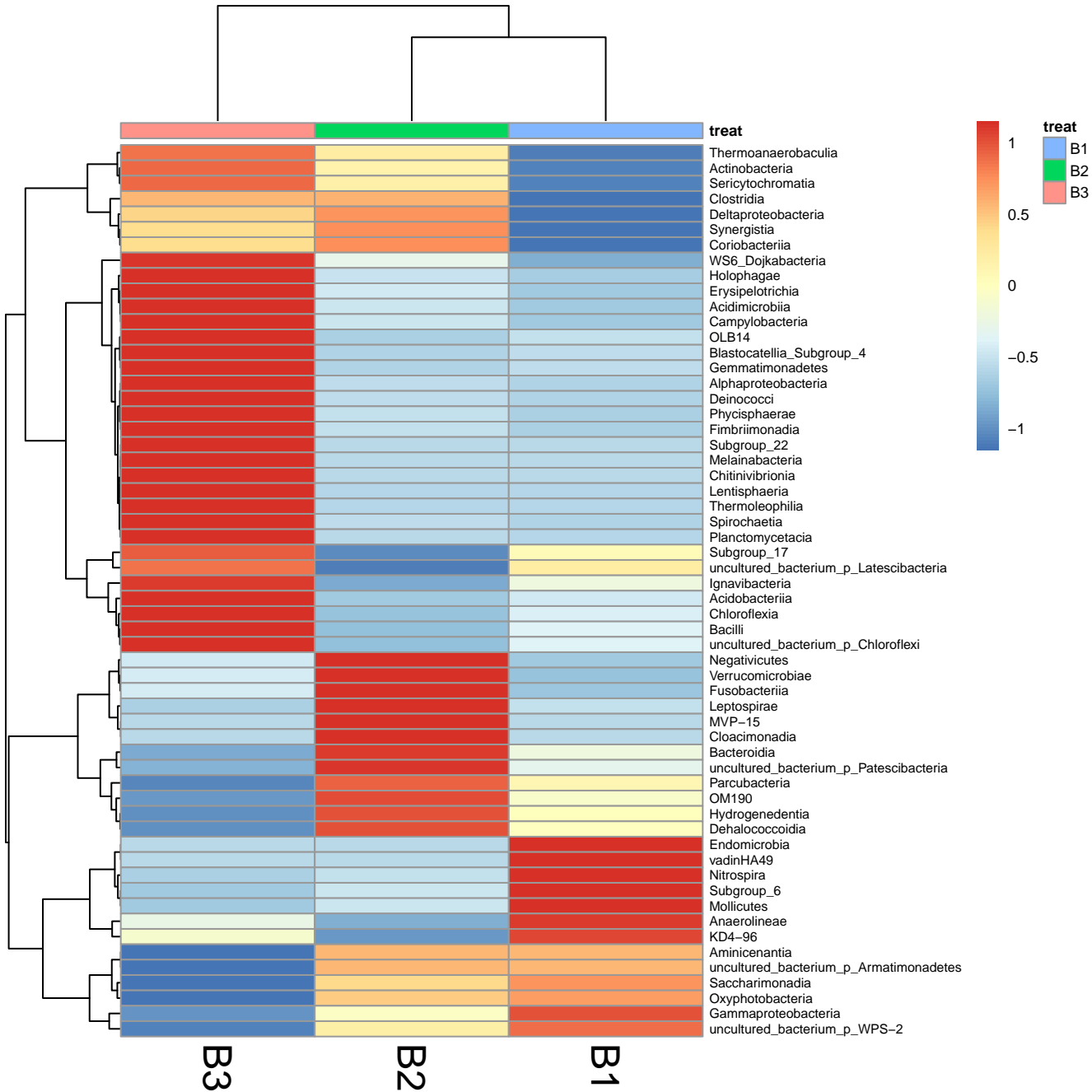

Supplement: S2 Data — (ZIP) [file pone.0261306.s002.zip › customer_backup/taxa_summary/heatmap/treat/treat.class.reabundance.heatmap.pdf]

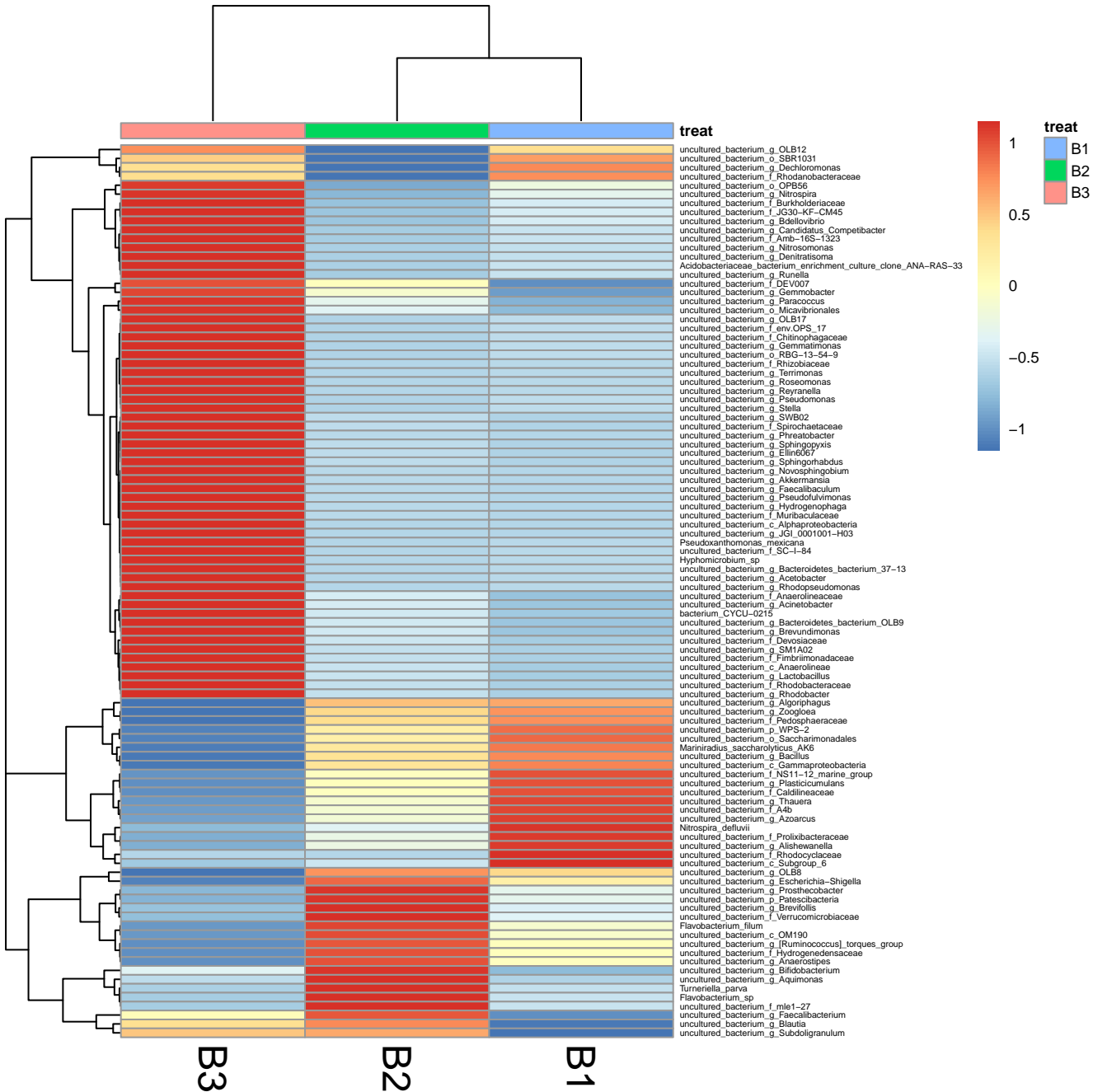

Supplement: S2 Data — (ZIP) [file pone.0261306.s002.zip › customer_backup/taxa_summary/heatmap/treat/treat.species.reabundance.heatmap.pdf]

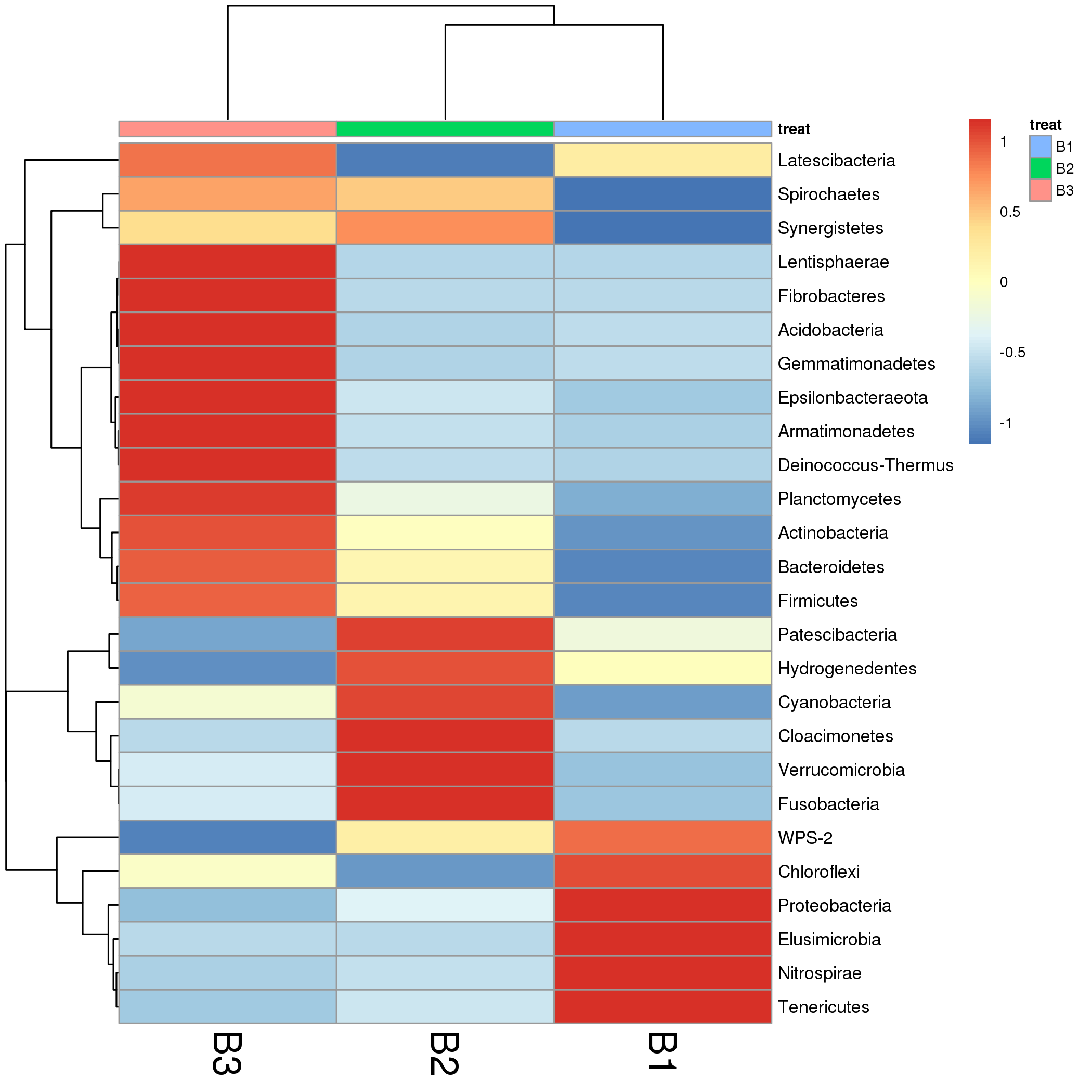

Supplement: S2 Data — (ZIP) [file pone.0261306.s002.zip › customer_backup/taxa_summary/heatmap/treat/treat.phylum.reabundance.heatmap.png]

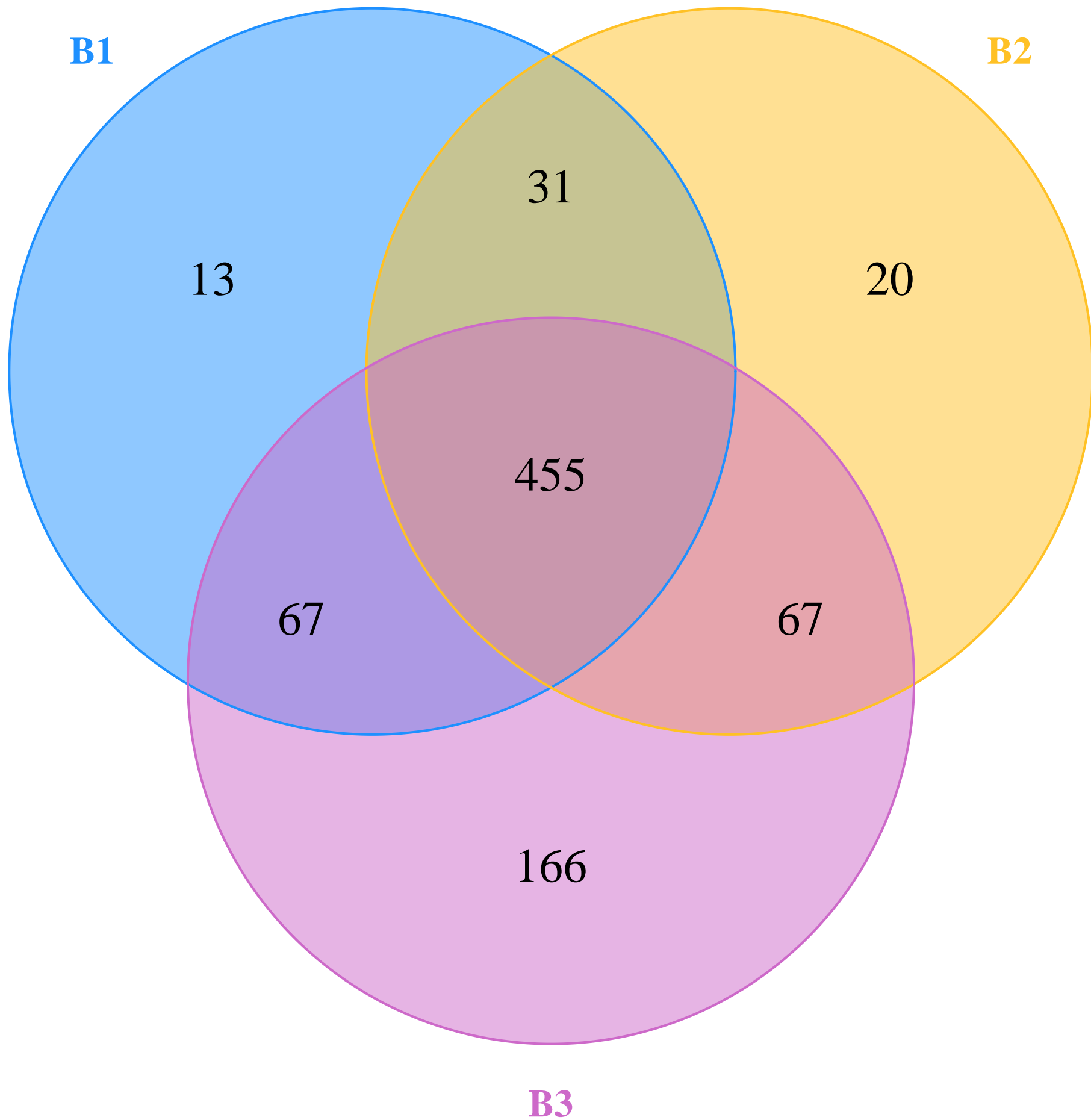

Supplement: S2 Data — (ZIP) [file pone.0261306.s002.zip › customer_backup/otus/venn/treat/treat_venn.pdf]

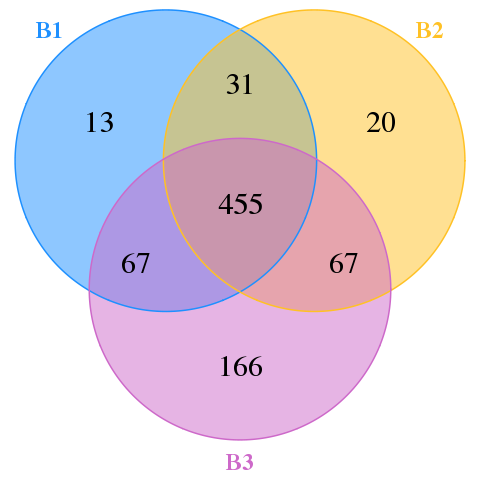

Supplement: S2 Data — (ZIP) [file pone.0261306.s002.zip › customer_backup/otus/venn/treat/treat_venn.png]

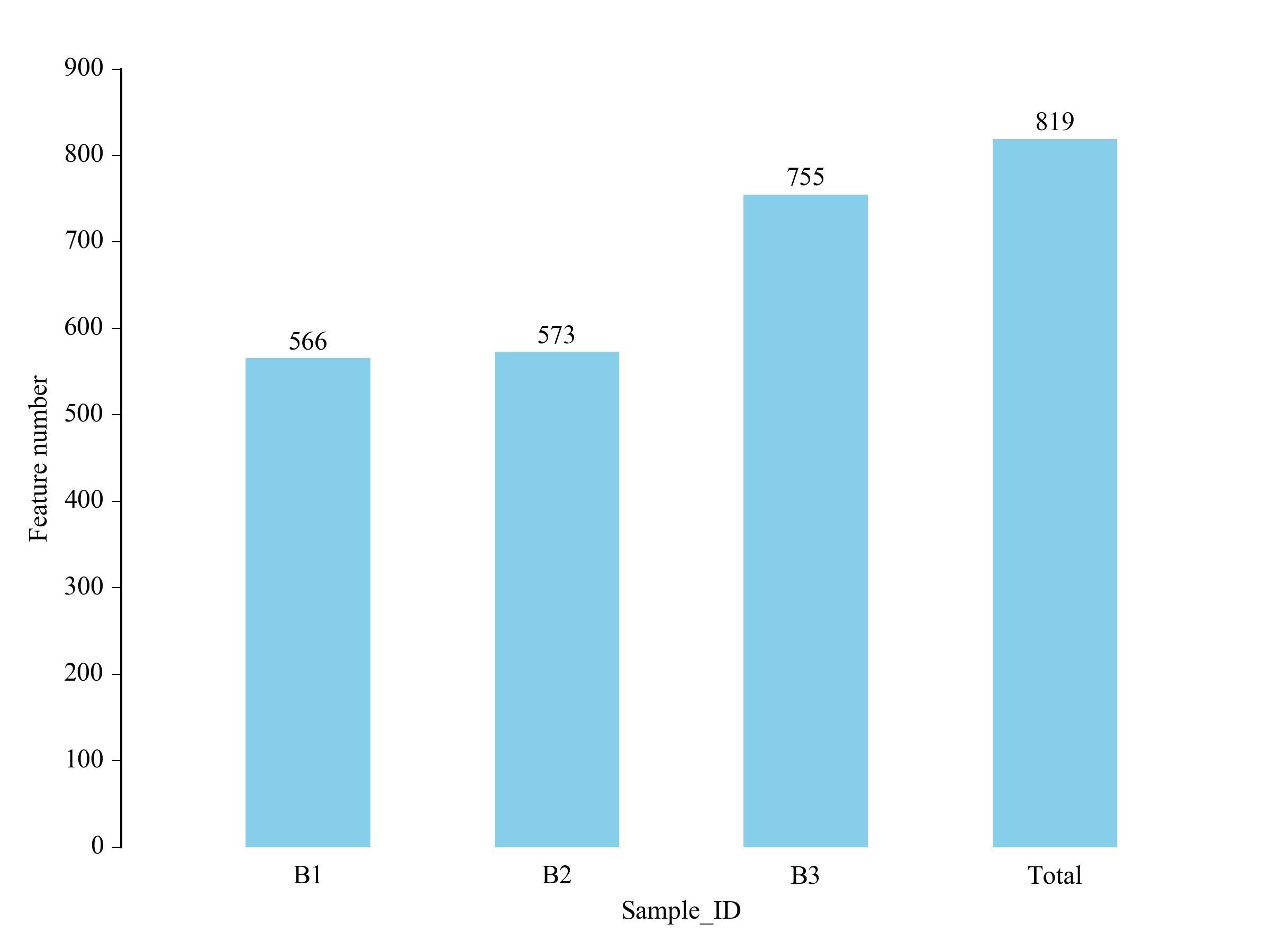

Supplement: S2 Data — (ZIP) [file pone.0261306.s002.zip › customer_backup/otus/OTU_distri/treat/treat.otu.bar.png]

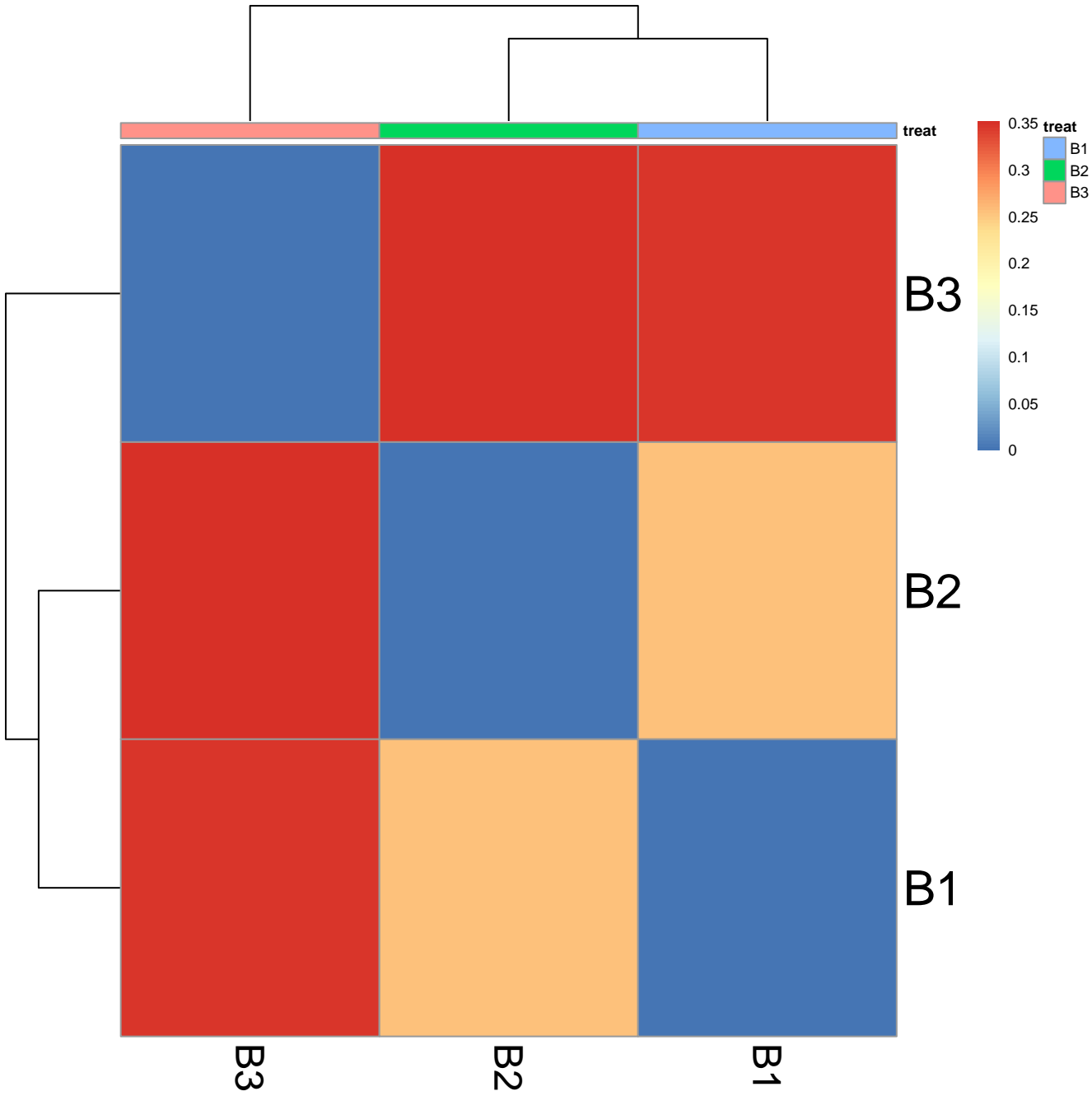

Supplement: S2 Data — (ZIP) [file pone.0261306.s002.zip › customer_backup/beta_diversity/sample_heatmap/treat/treat.binary_jaccard_dm.heatmap.pdf]

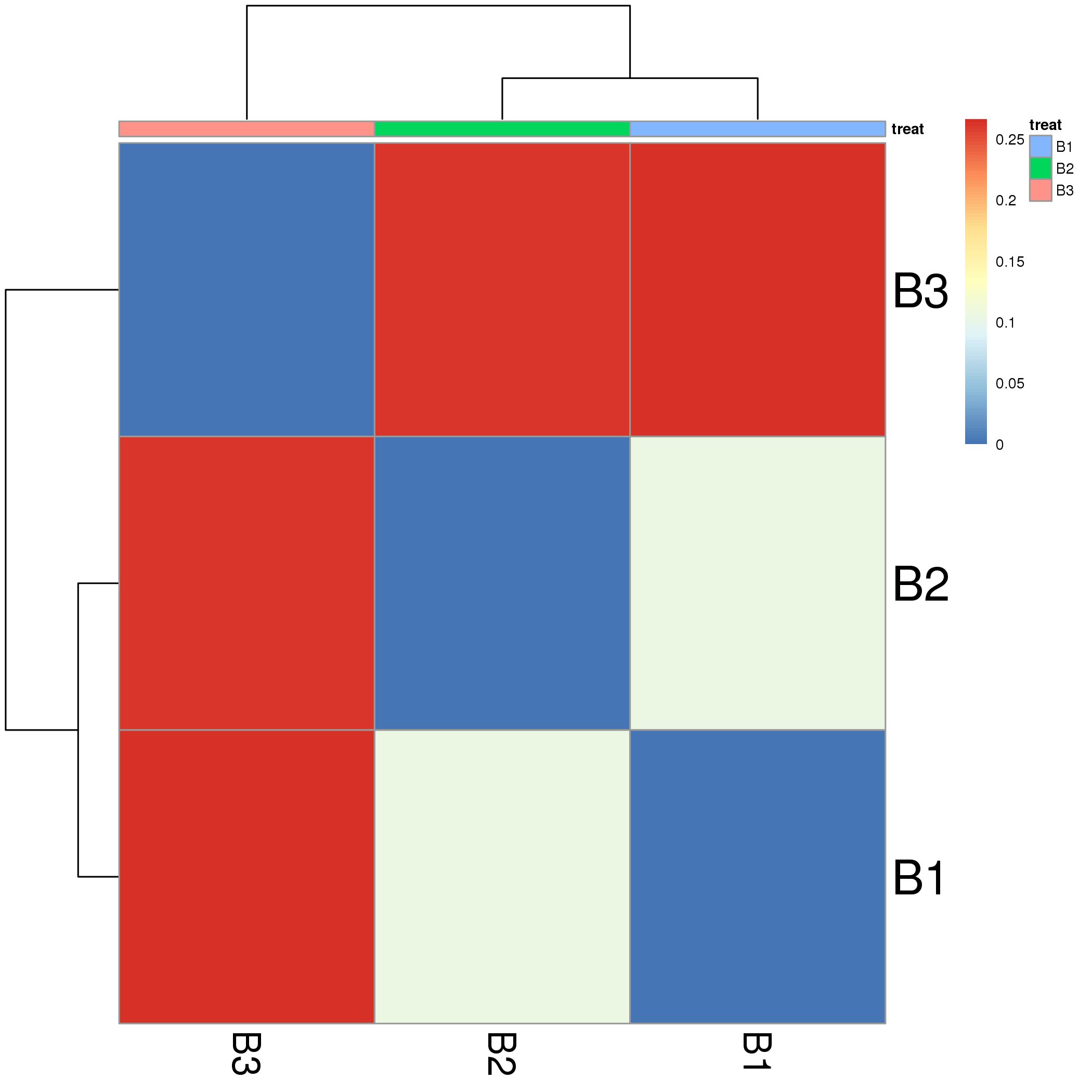

Supplement: S2 Data — (ZIP) [file pone.0261306.s002.zip › customer_backup/beta_diversity/sample_heatmap/treat/treat.weighted_unifrac_dm.heatmap.png]

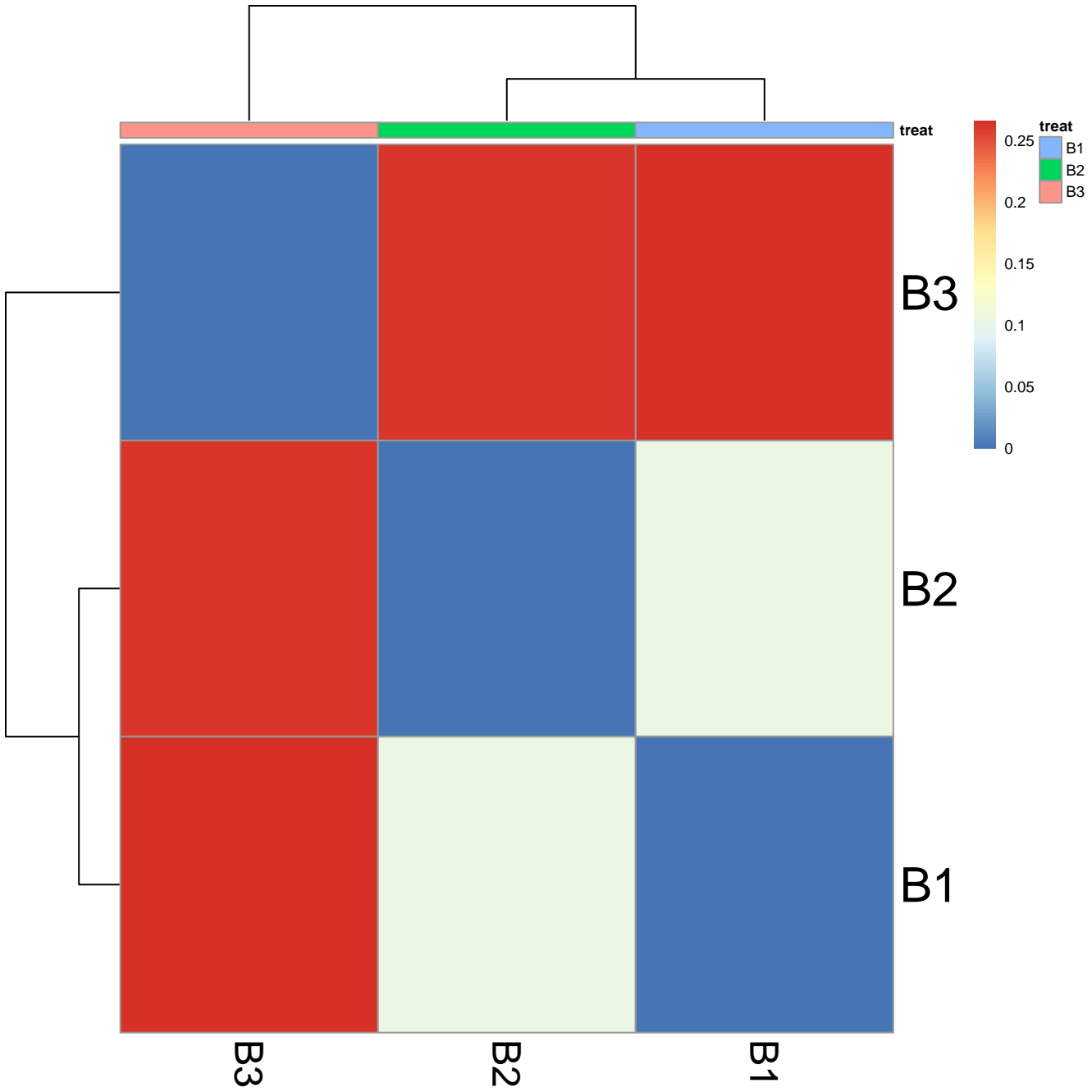

Supplement: S2 Data — (ZIP) [file pone.0261306.s002.zip › customer_backup/beta_diversity/sample_heatmap/treat/treat.weighted_unifrac_dm.heatmap.pdf]

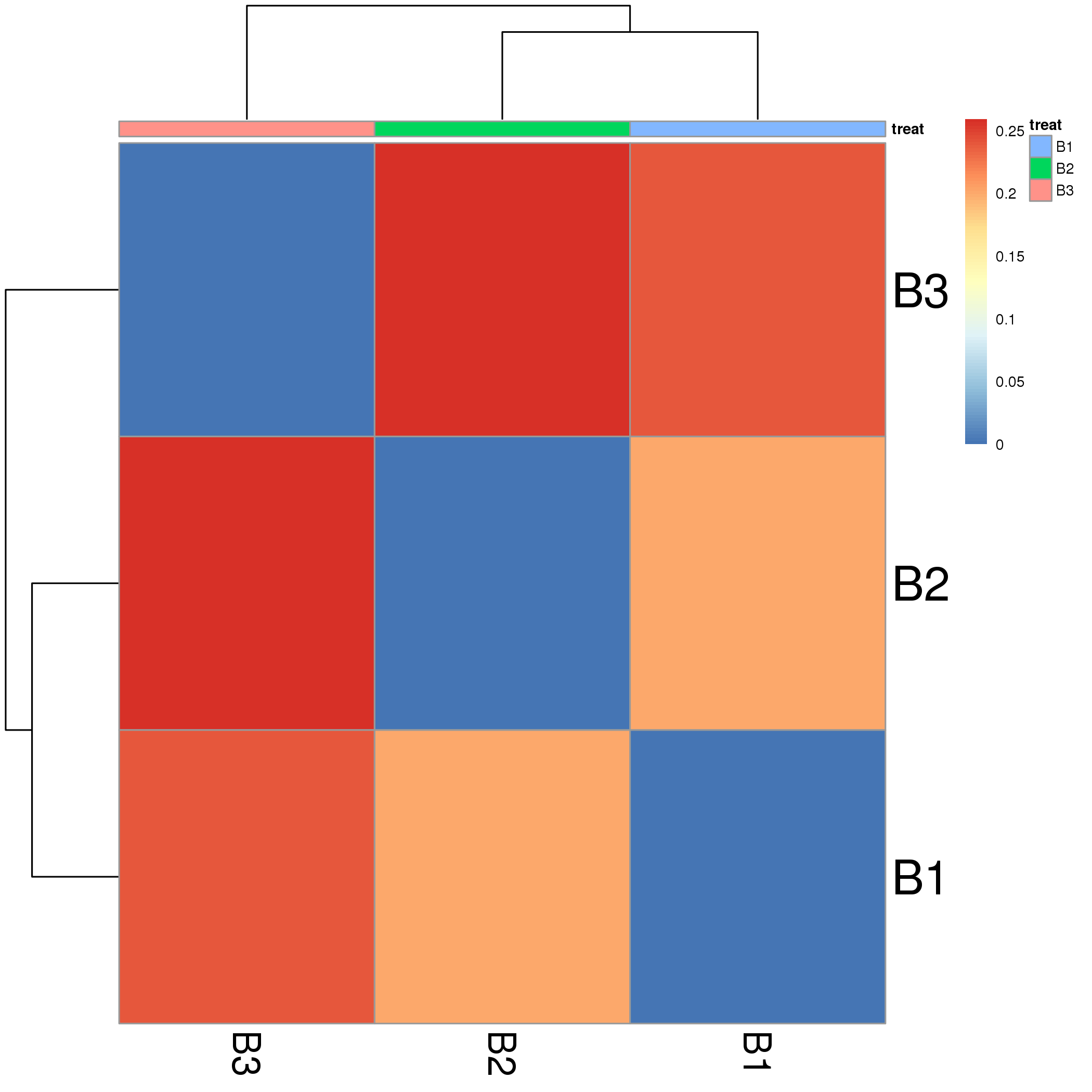

Supplement: S2 Data — (ZIP) [file pone.0261306.s002.zip › customer_backup/beta_diversity/sample_heatmap/treat/treat.unweighted_unifrac_dm.heatmap.png]

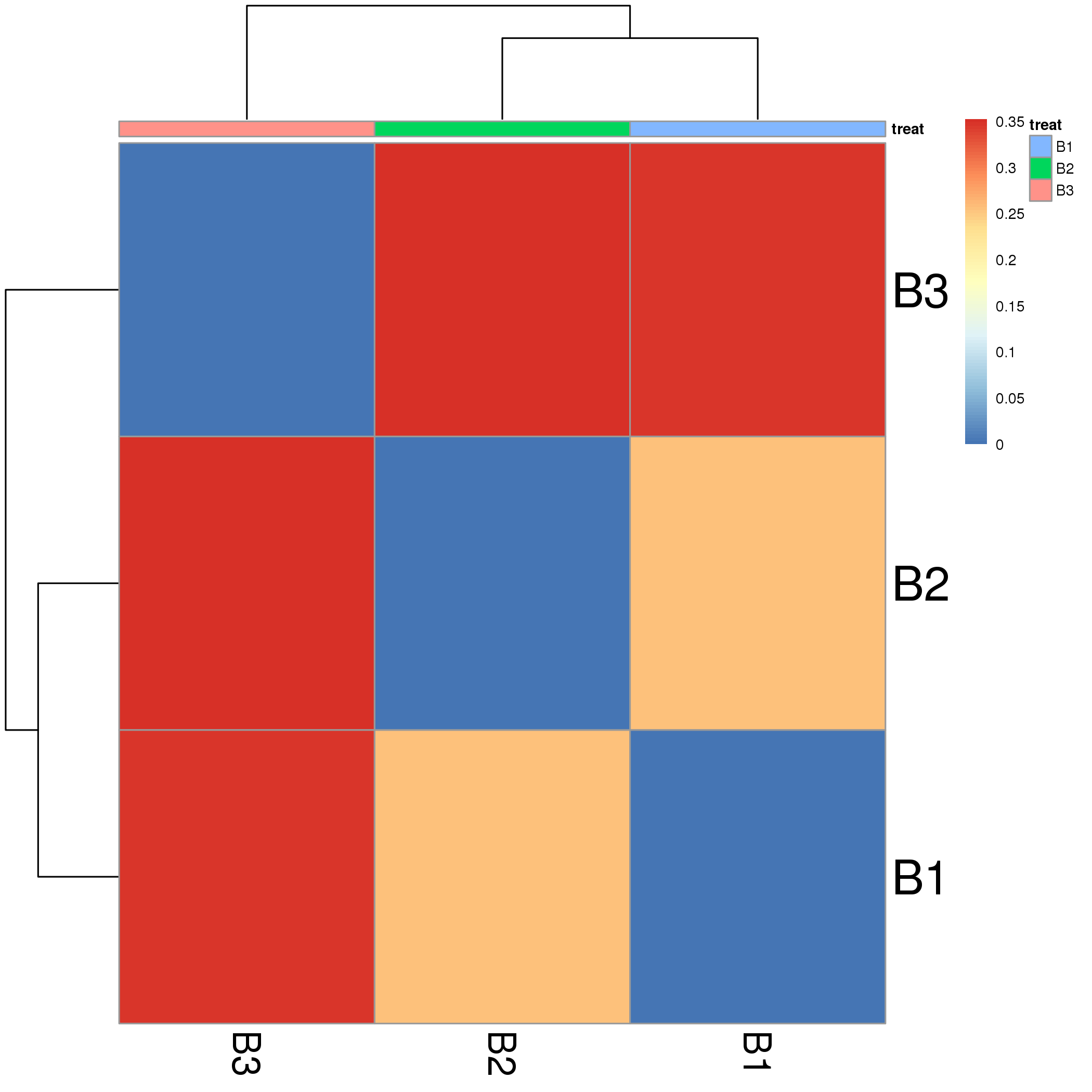

Supplement: S2 Data — (ZIP) [file pone.0261306.s002.zip › customer_backup/beta_diversity/sample_heatmap/treat/treat.binary_jaccard_dm.heatmap.png]

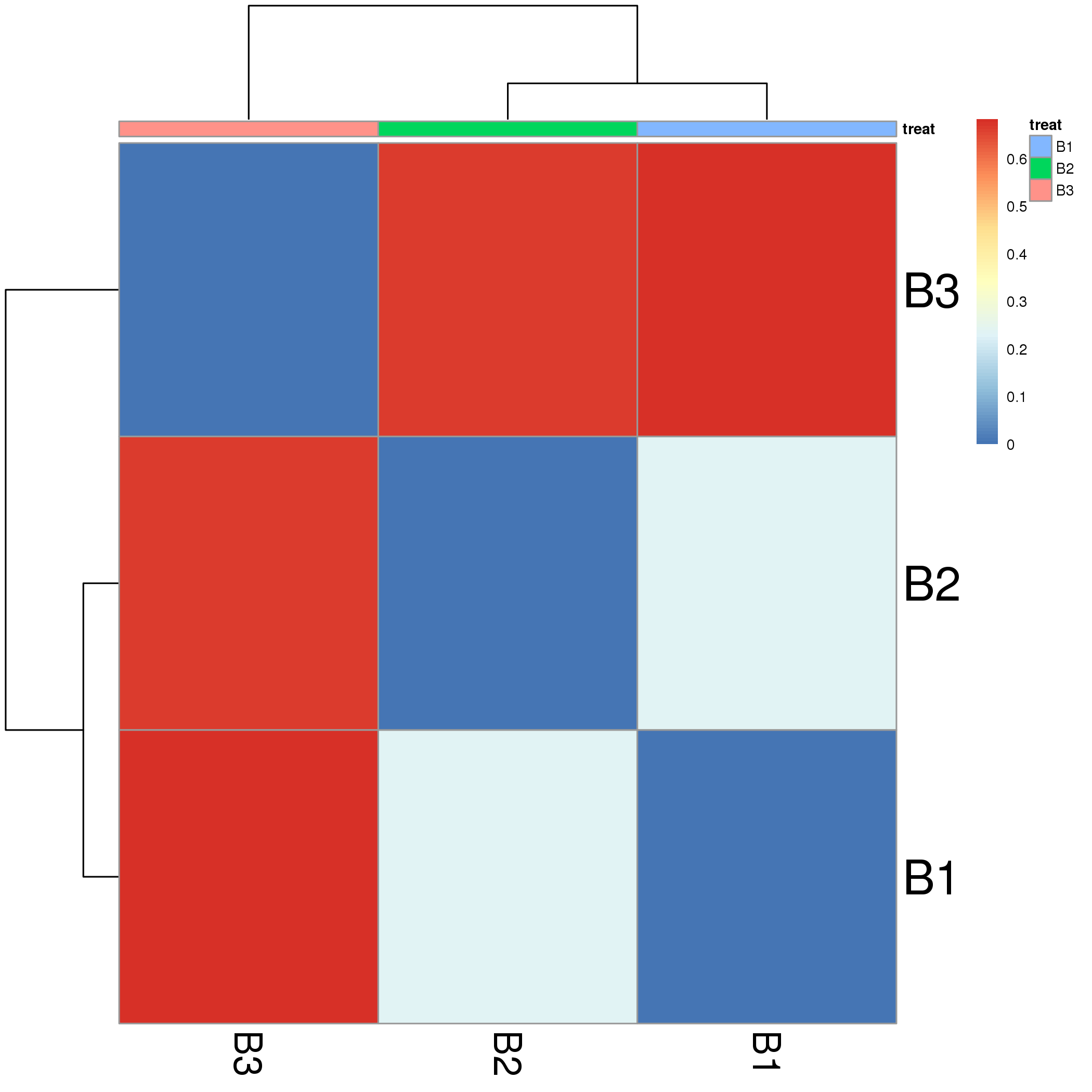

Supplement: S2 Data — (ZIP) [file pone.0261306.s002.zip › customer_backup/beta_diversity/sample_heatmap/treat/treat.bray_curtis_dm.heatmap.png]

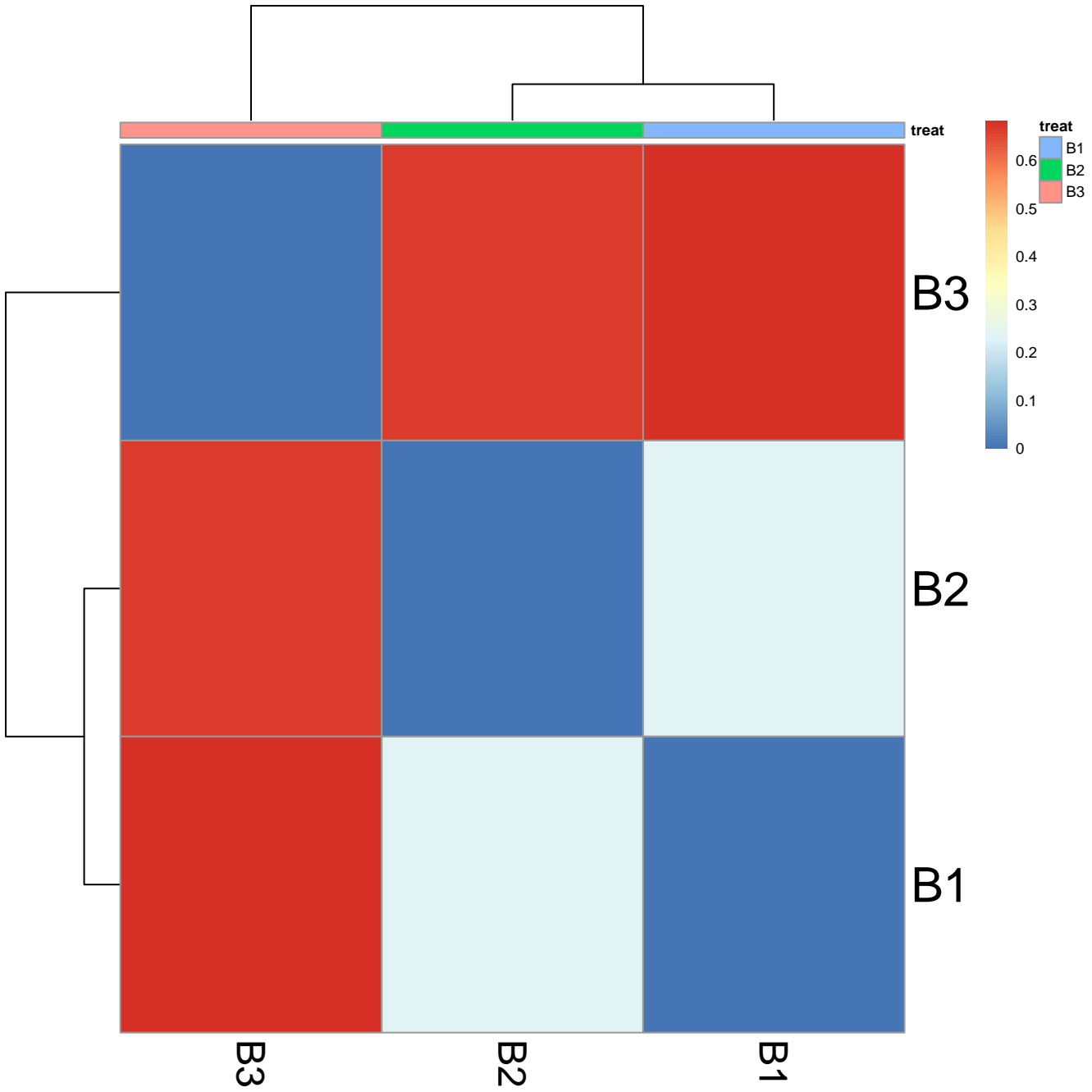

Supplement: S2 Data — (ZIP) [file pone.0261306.s002.zip › customer_backup/beta_diversity/sample_heatmap/treat/treat.bray_curtis_dm.heatmap.pdf]

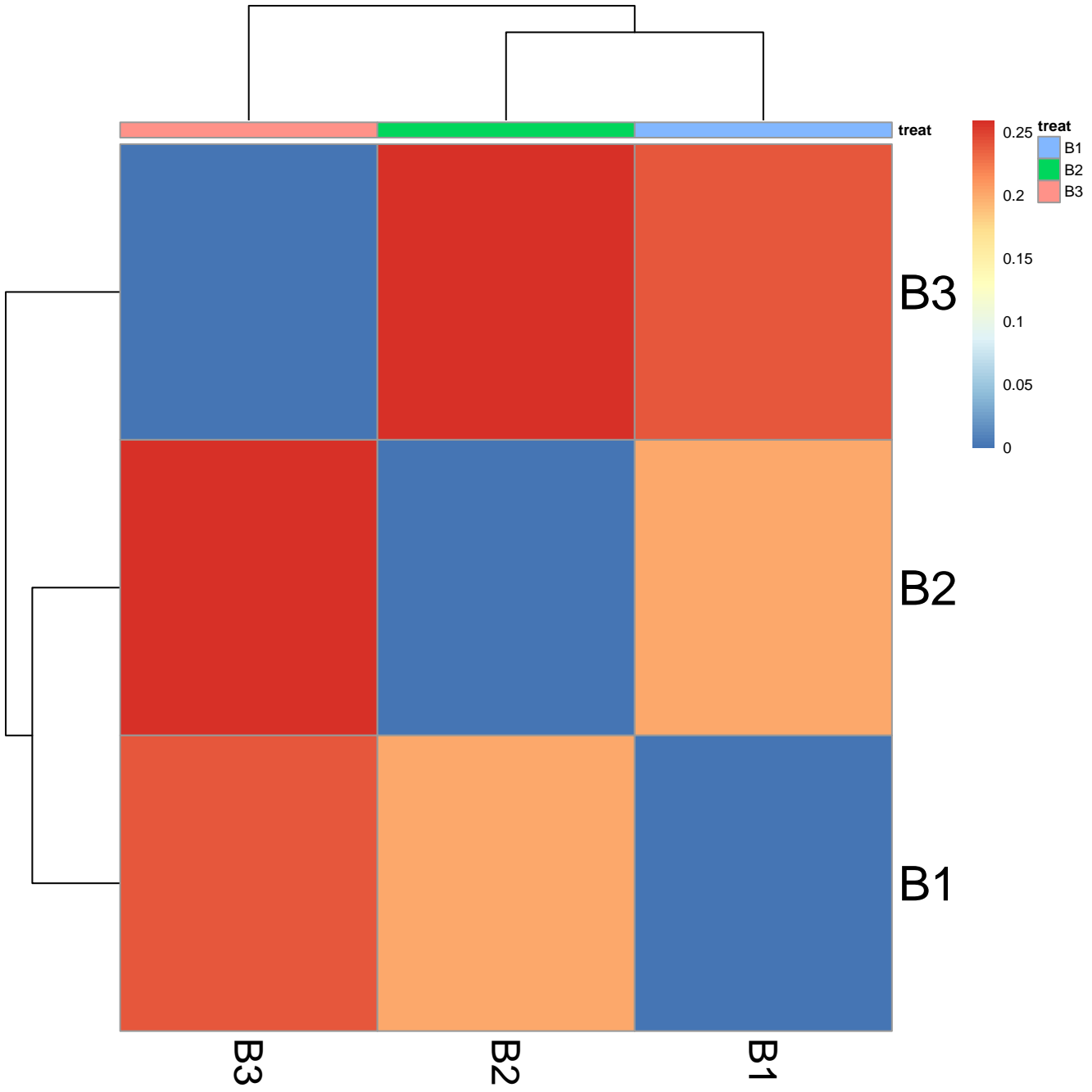

Supplement: S2 Data — (ZIP) [file pone.0261306.s002.zip › customer_backup/beta_diversity/sample_heatmap/treat/treat.unweighted_unifrac_dm.heatmap.pdf]

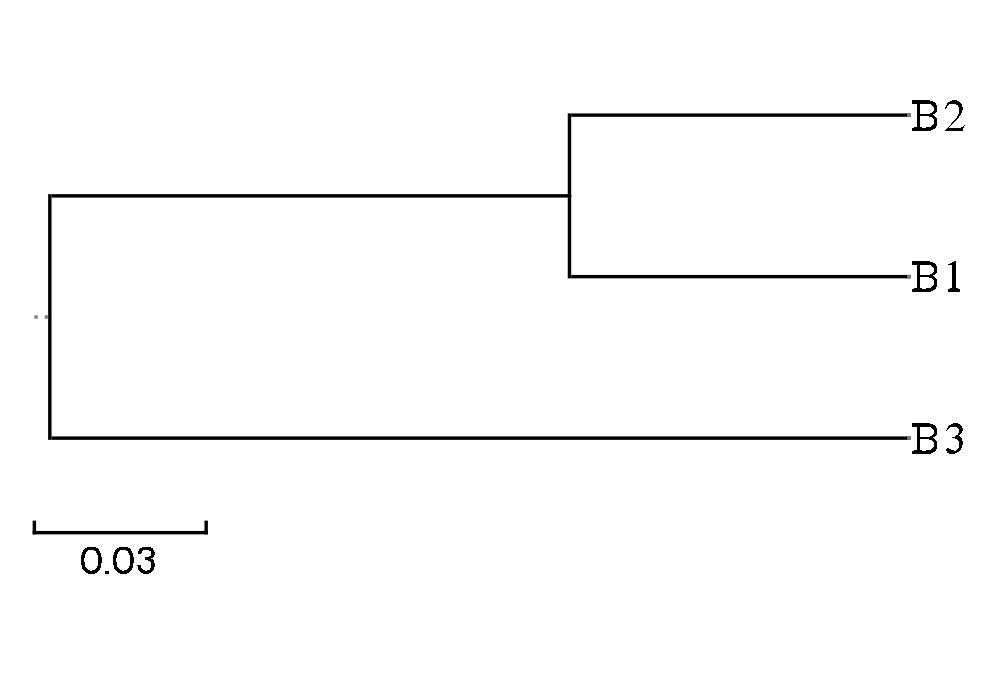

Supplement: S2 Data — (ZIP) [file pone.0261306.s002.zip › customer_backup/beta_diversity/upgma_tree/treat/treat.weighted_unifrac.png]

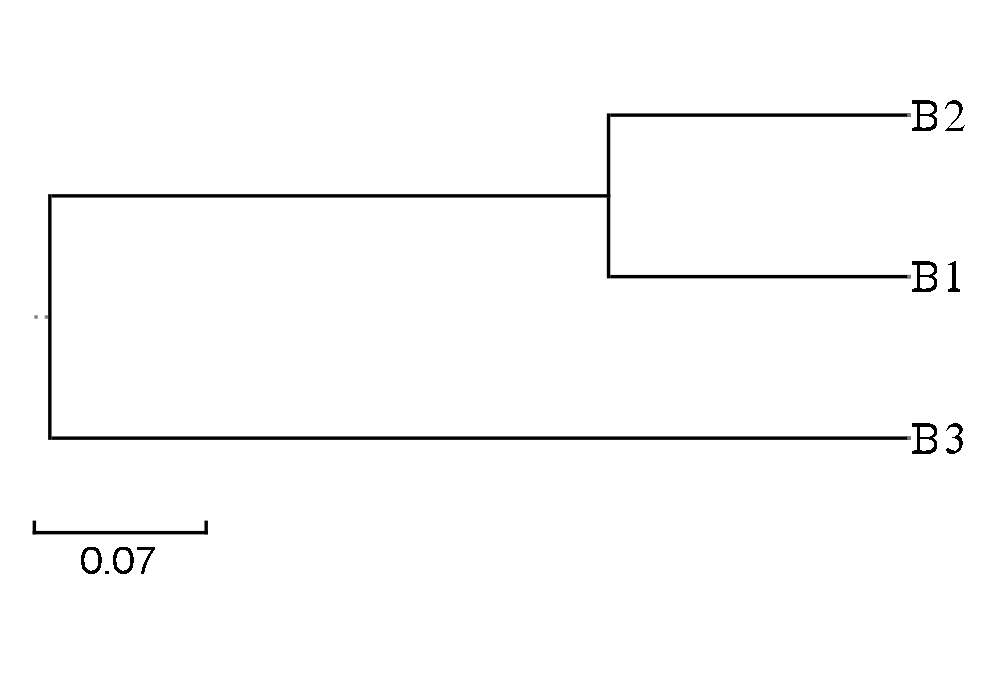

Supplement: S2 Data — (ZIP) [file pone.0261306.s002.zip › customer_backup/beta_diversity/upgma_tree/treat/treat.bray_curtis.png]

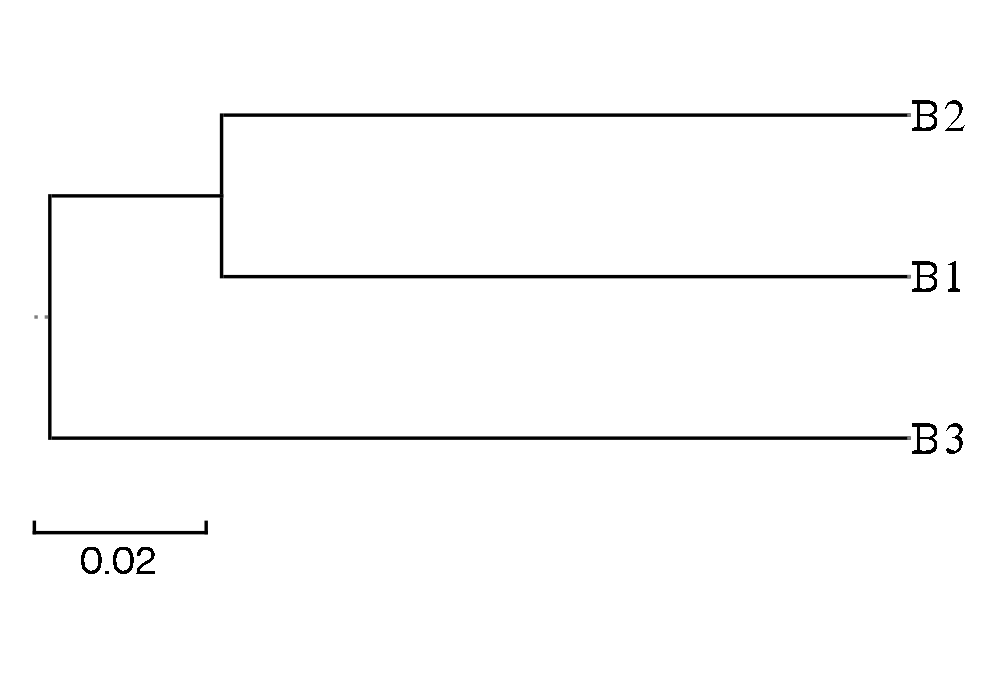

Supplement: S2 Data — (ZIP) [file pone.0261306.s002.zip › customer_backup/beta_diversity/upgma_tree/treat/treat.unweighted_unifrac.png]

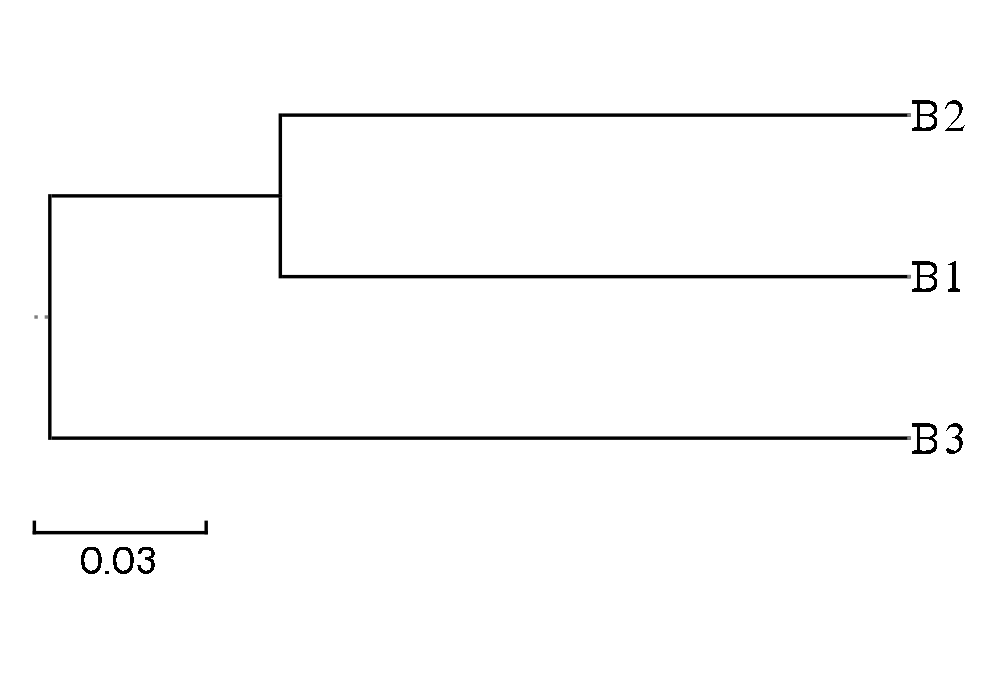

Supplement: S2 Data — (ZIP) [file pone.0261306.s002.zip › customer_backup/beta_diversity/upgma_tree/treat/treat.binary_jaccard.png]

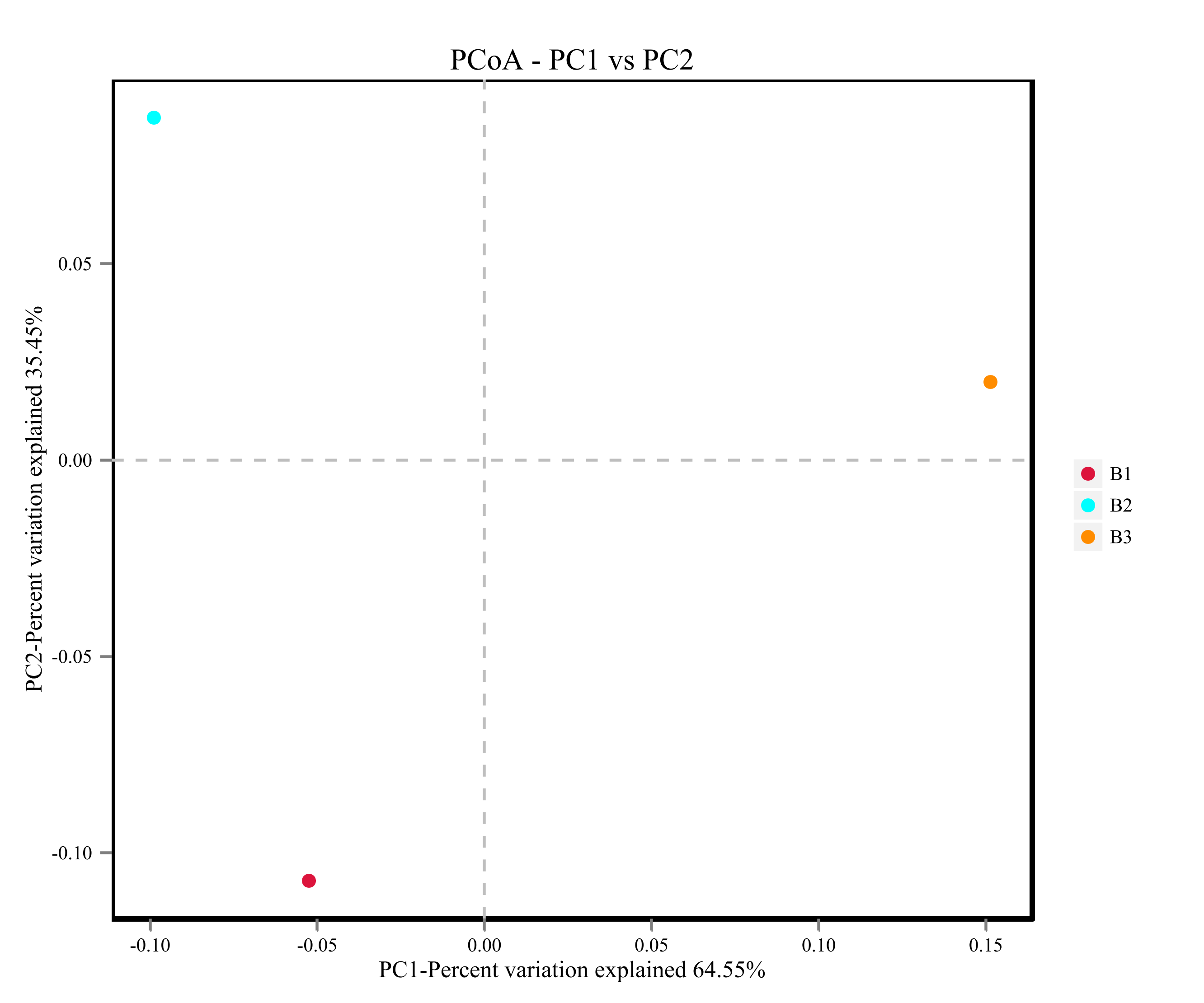

Supplement: S2 Data — (ZIP) [file pone.0261306.s002.zip › customer_backup/beta_diversity/pcoa/treat/treat.unweighted_unifrac.PC1_PC2.png]

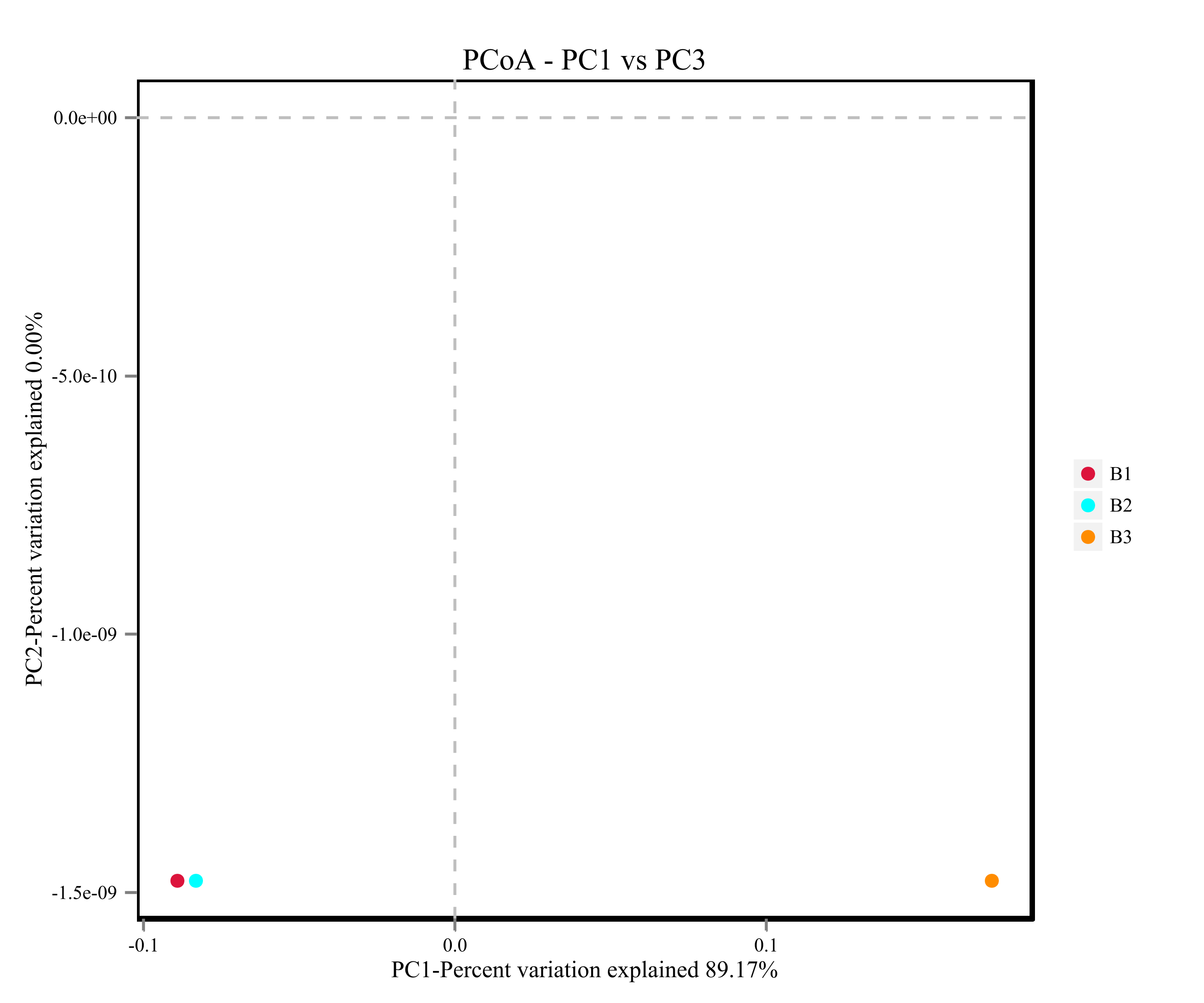

Supplement: S2 Data — (ZIP) [file pone.0261306.s002.zip › customer_backup/beta_diversity/pcoa/treat/treat.weighted_unifrac.PC1_PC3.png]

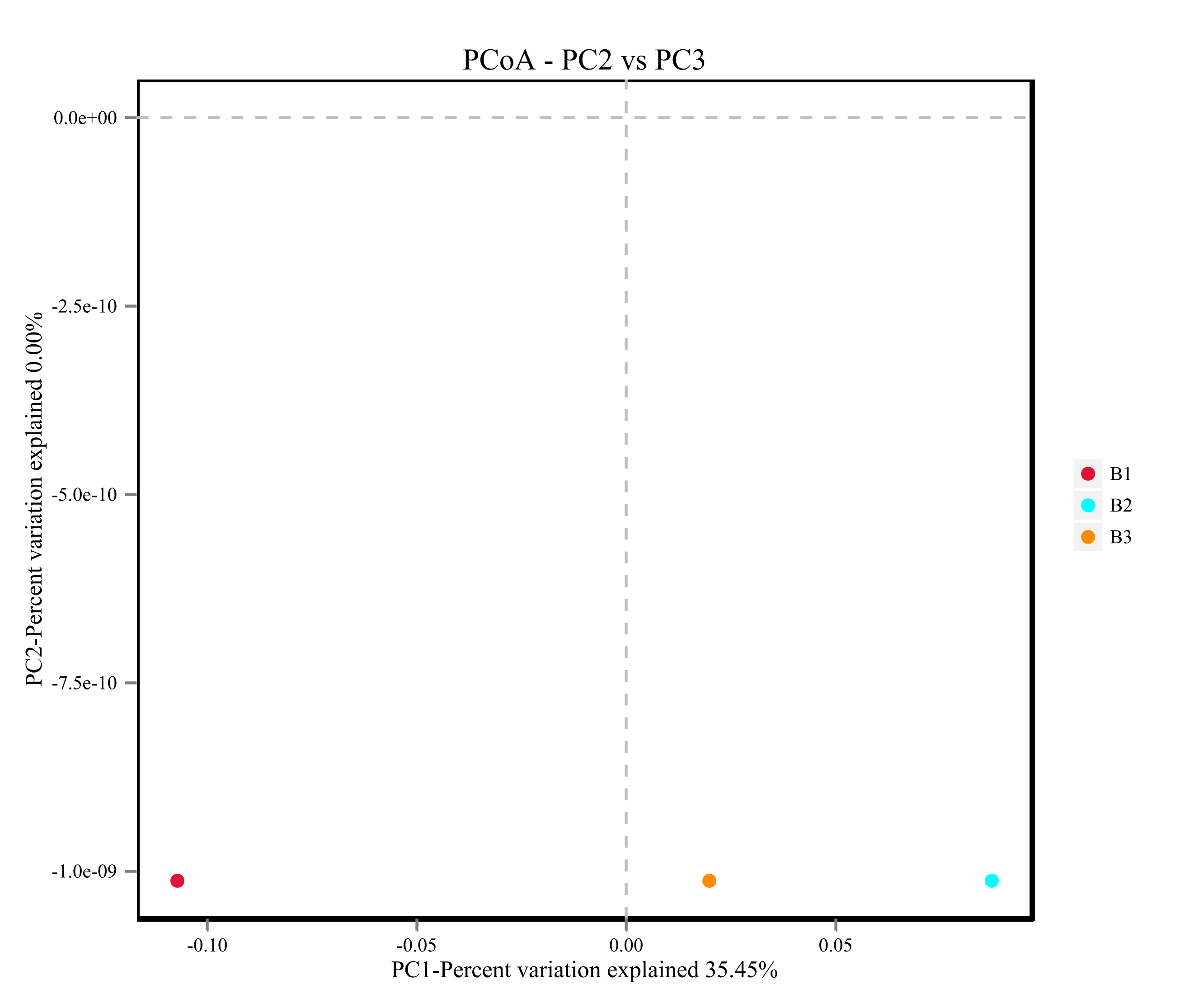

Supplement: S2 Data — (ZIP) [file pone.0261306.s002.zip › customer_backup/beta_diversity/pcoa/treat/treat.unweighted_unifrac.PC2_PC3.png]

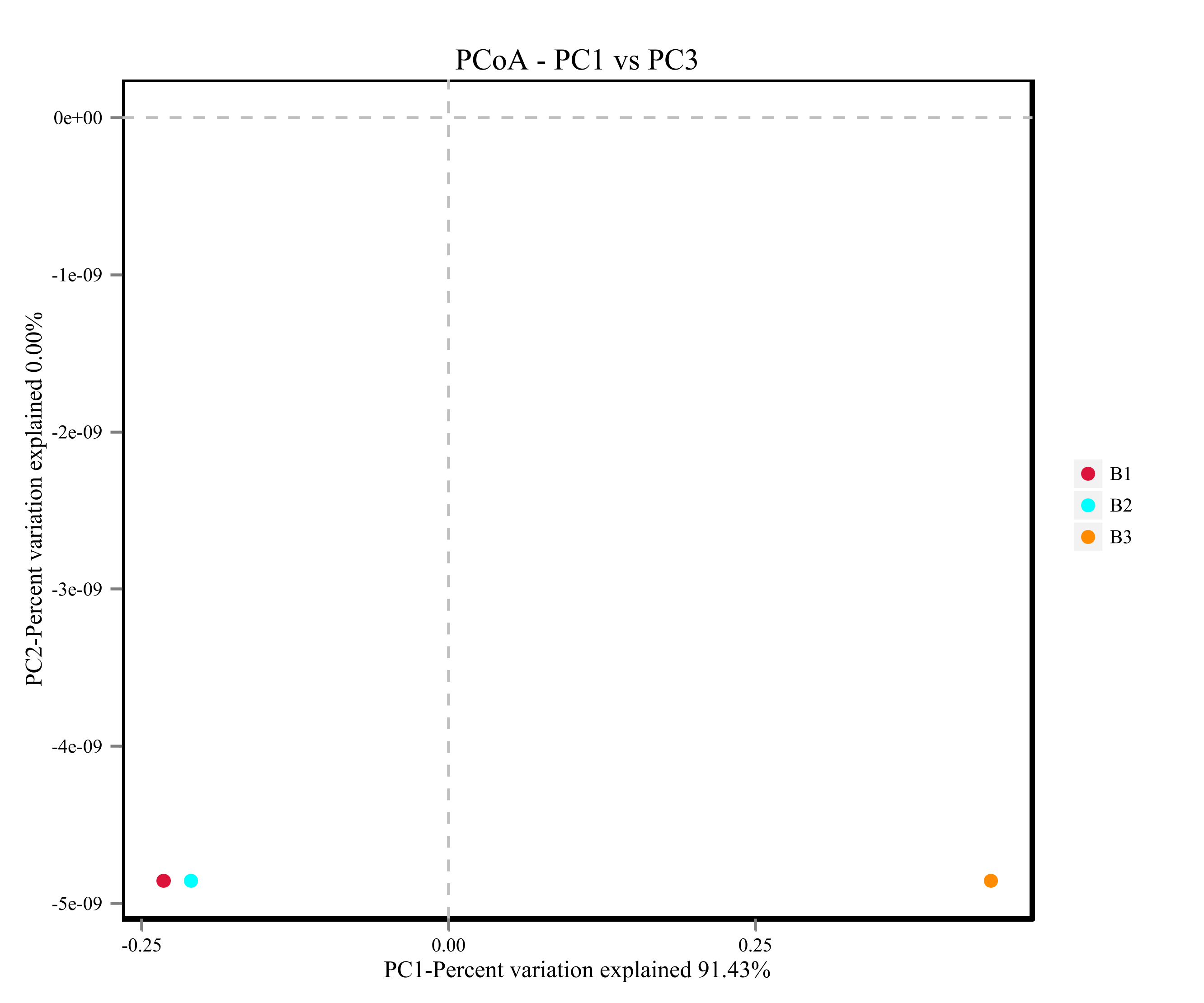

Supplement: S2 Data — (ZIP) [file pone.0261306.s002.zip › customer_backup/beta_diversity/pcoa/treat/treat.bray_curtis.PC1_PC3.png]

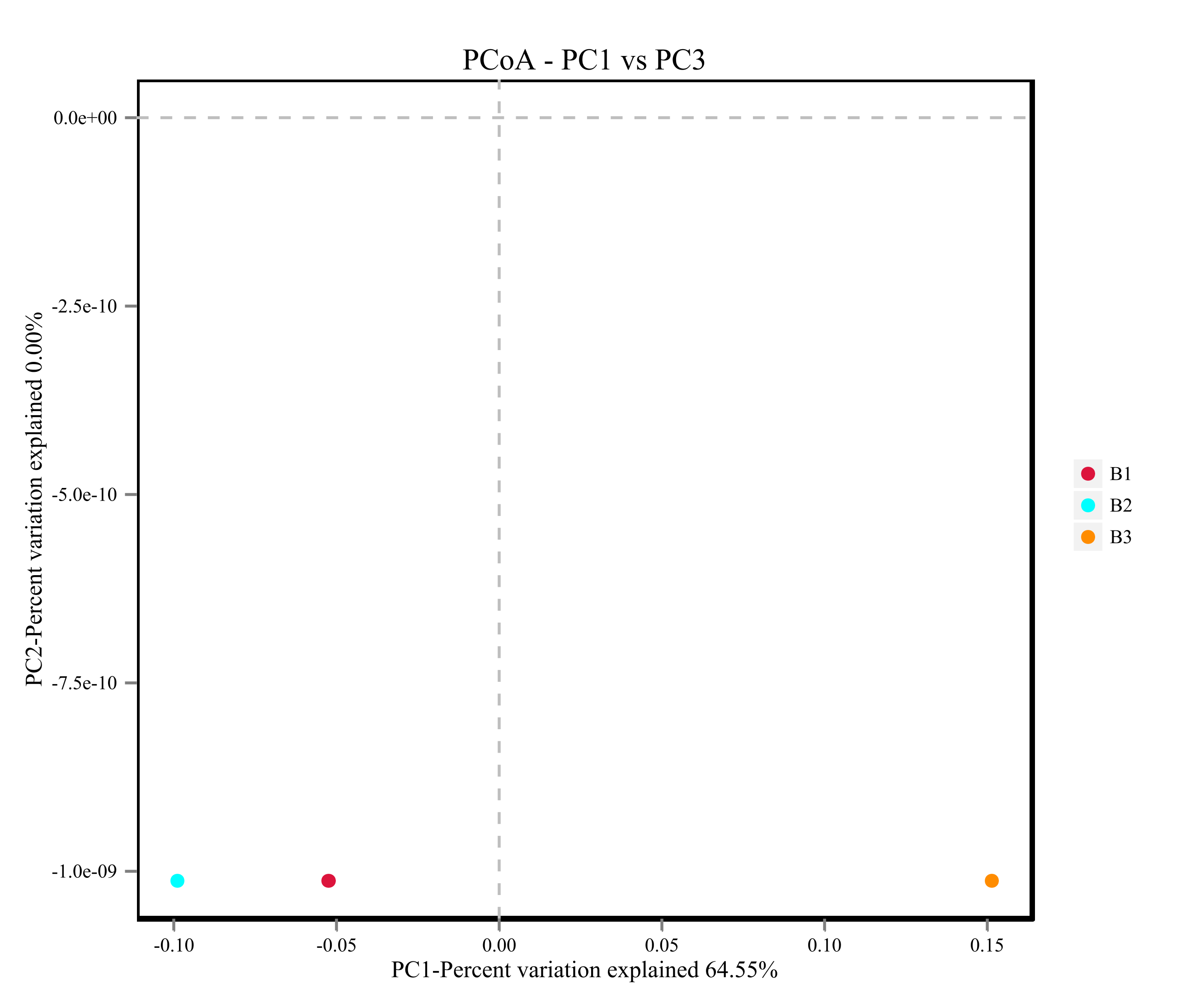

Supplement: S2 Data — (ZIP) [file pone.0261306.s002.zip › customer_backup/beta_diversity/pcoa/treat/treat.unweighted_unifrac.PC1_PC3.png]

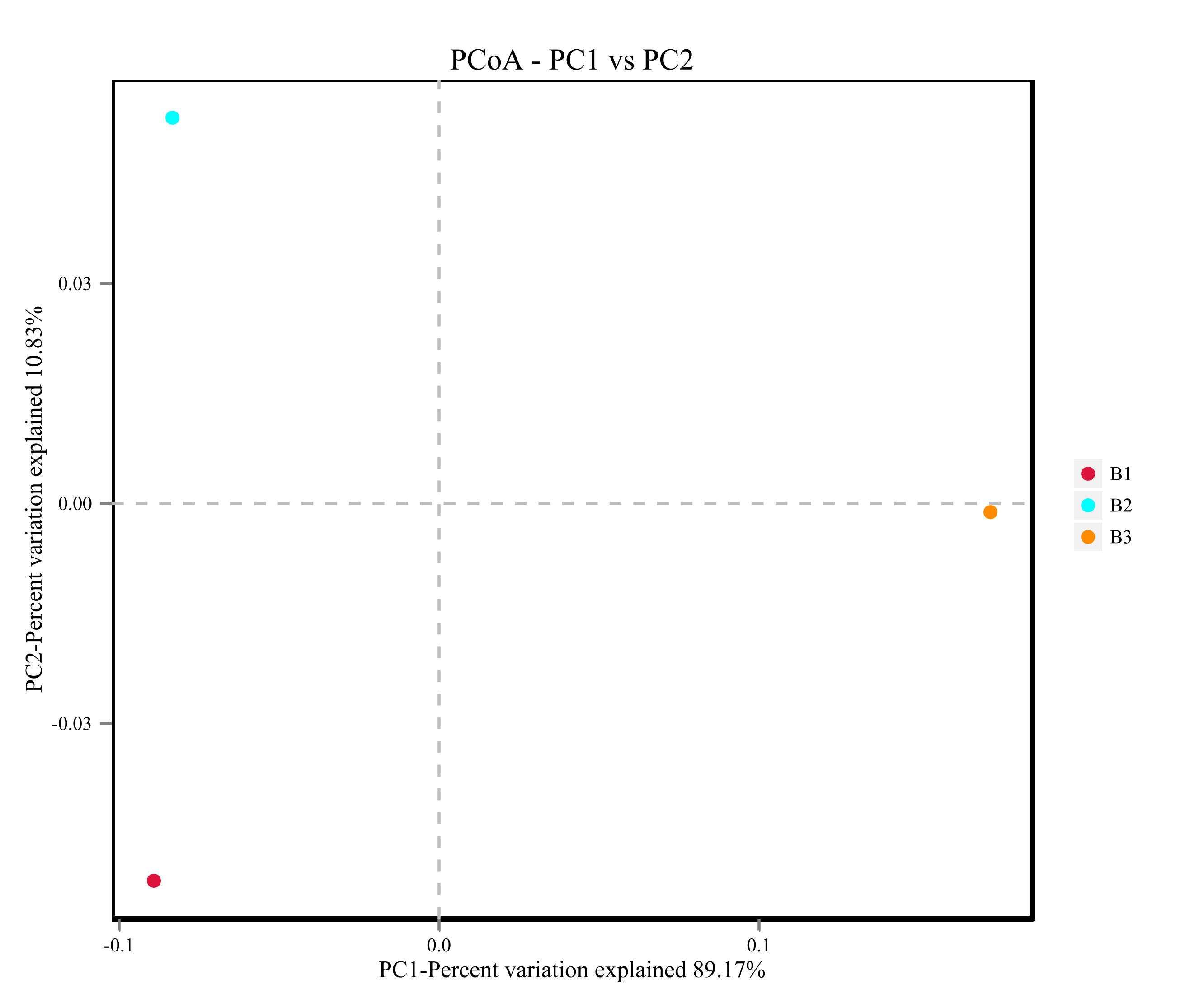

Supplement: S2 Data — (ZIP) [file pone.0261306.s002.zip › customer_backup/beta_diversity/pcoa/treat/treat.weighted_unifrac.PC1_PC2.png]

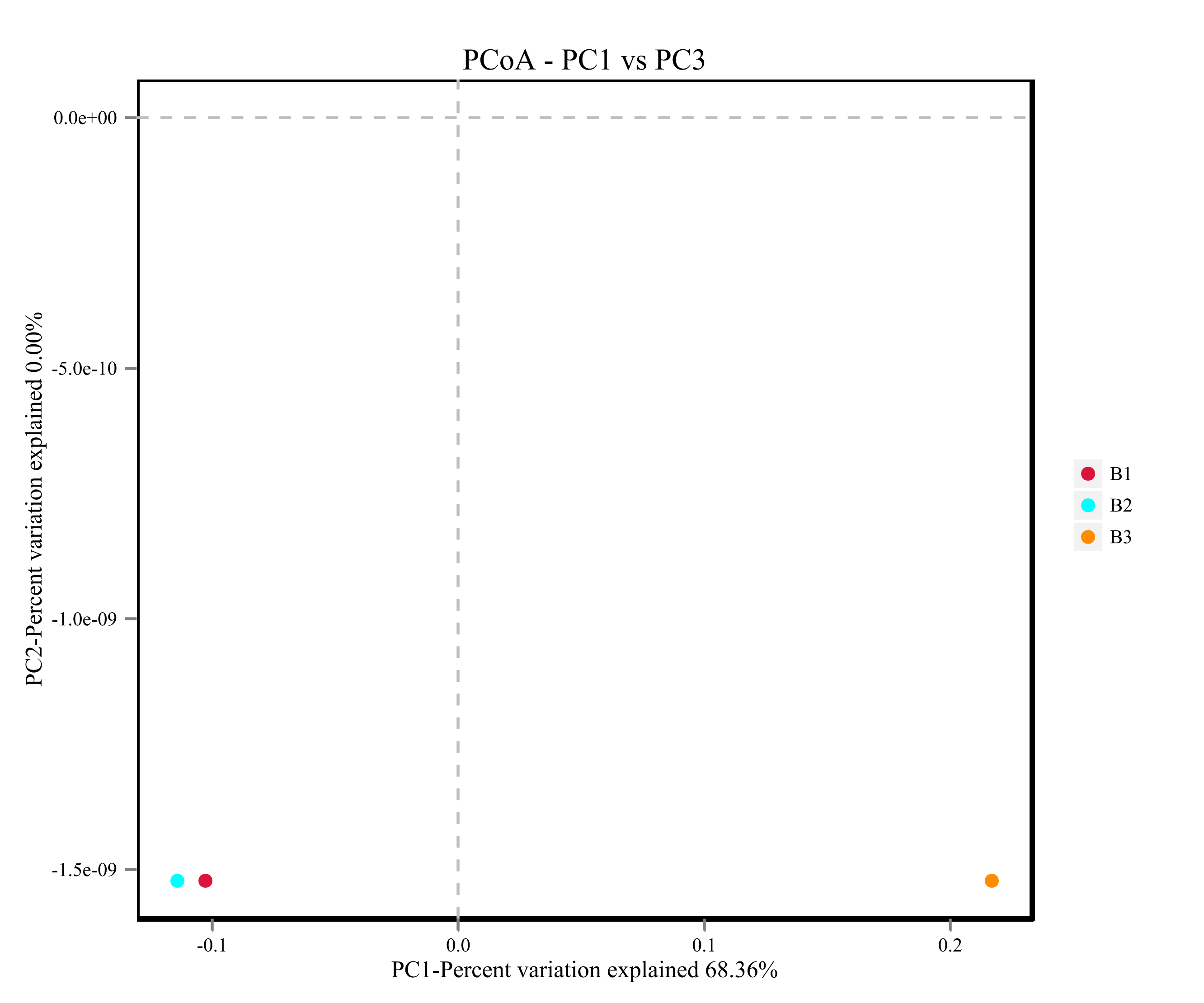

Supplement: S2 Data — (ZIP) [file pone.0261306.s002.zip › customer_backup/beta_diversity/pcoa/treat/treat.binary_jaccard.PC1_PC3.png]

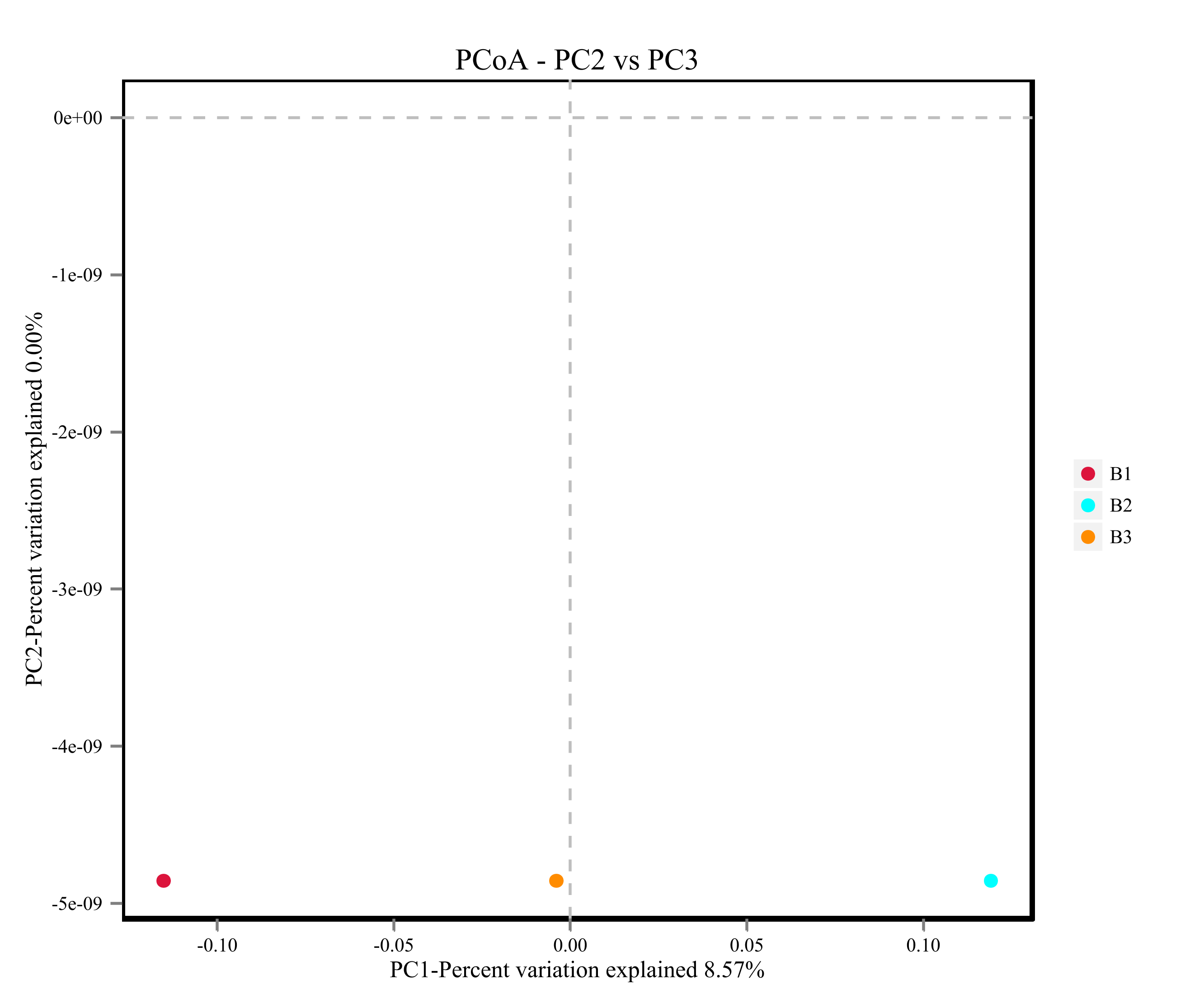

Supplement: S2 Data — (ZIP) [file pone.0261306.s002.zip › customer_backup/beta_diversity/pcoa/treat/treat.bray_curtis.PC2_PC3.png]

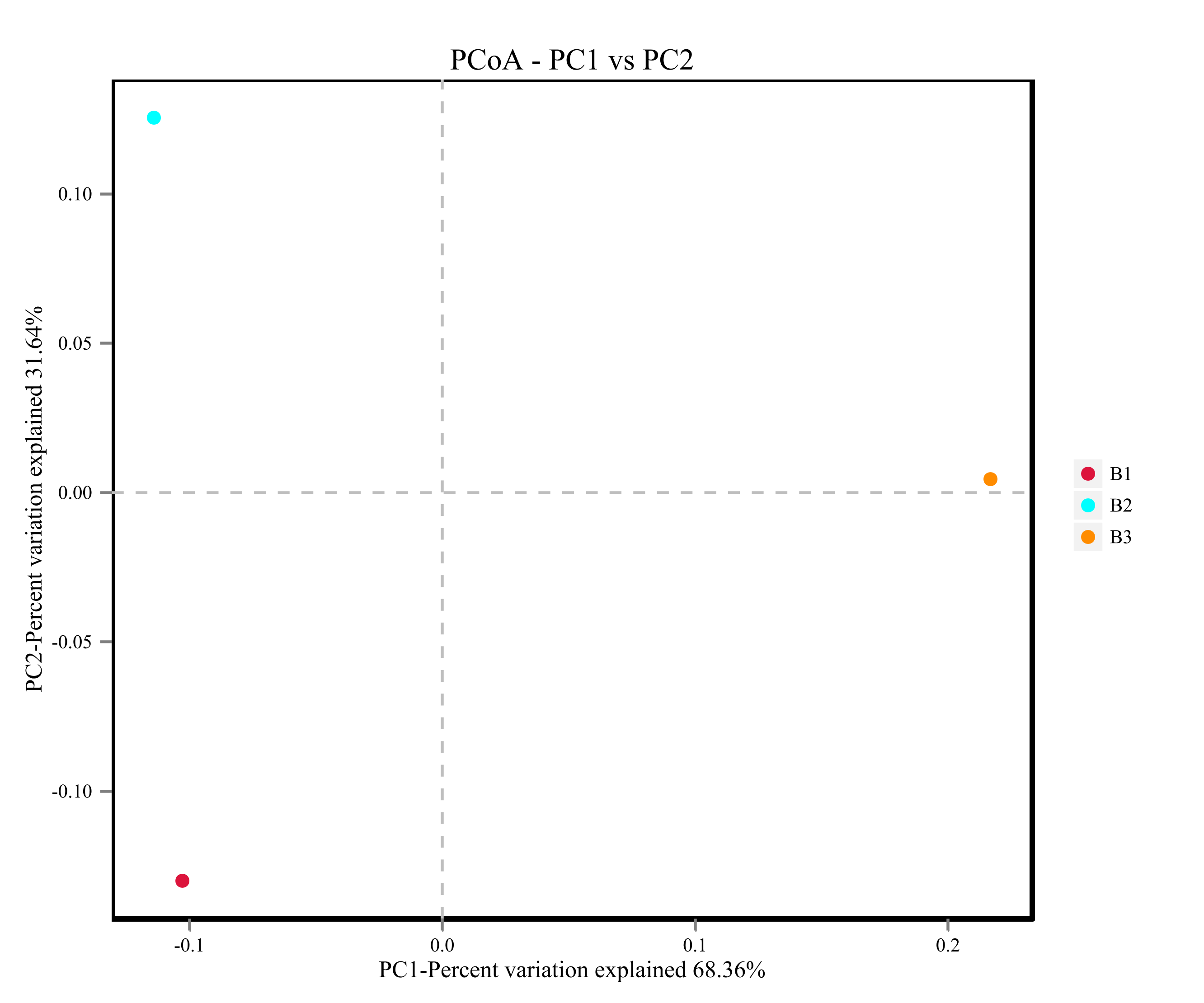

Supplement: S2 Data — (ZIP) [file pone.0261306.s002.zip › customer_backup/beta_diversity/pcoa/treat/treat.binary_jaccard.PC1_PC2.png]

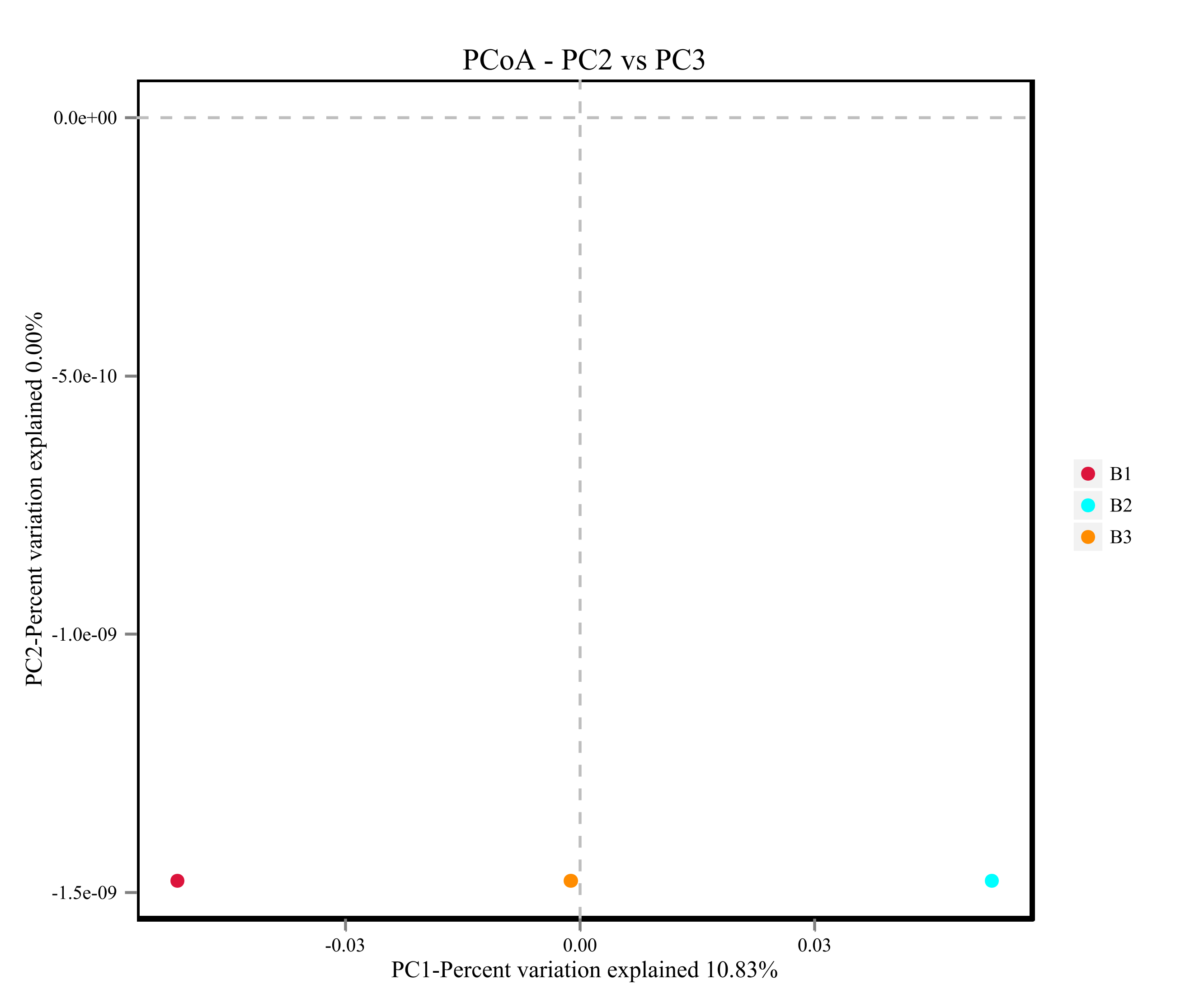

Supplement: S2 Data — (ZIP) [file pone.0261306.s002.zip › customer_backup/beta_diversity/pcoa/treat/treat.weighted_unifrac.PC2_PC3.png]

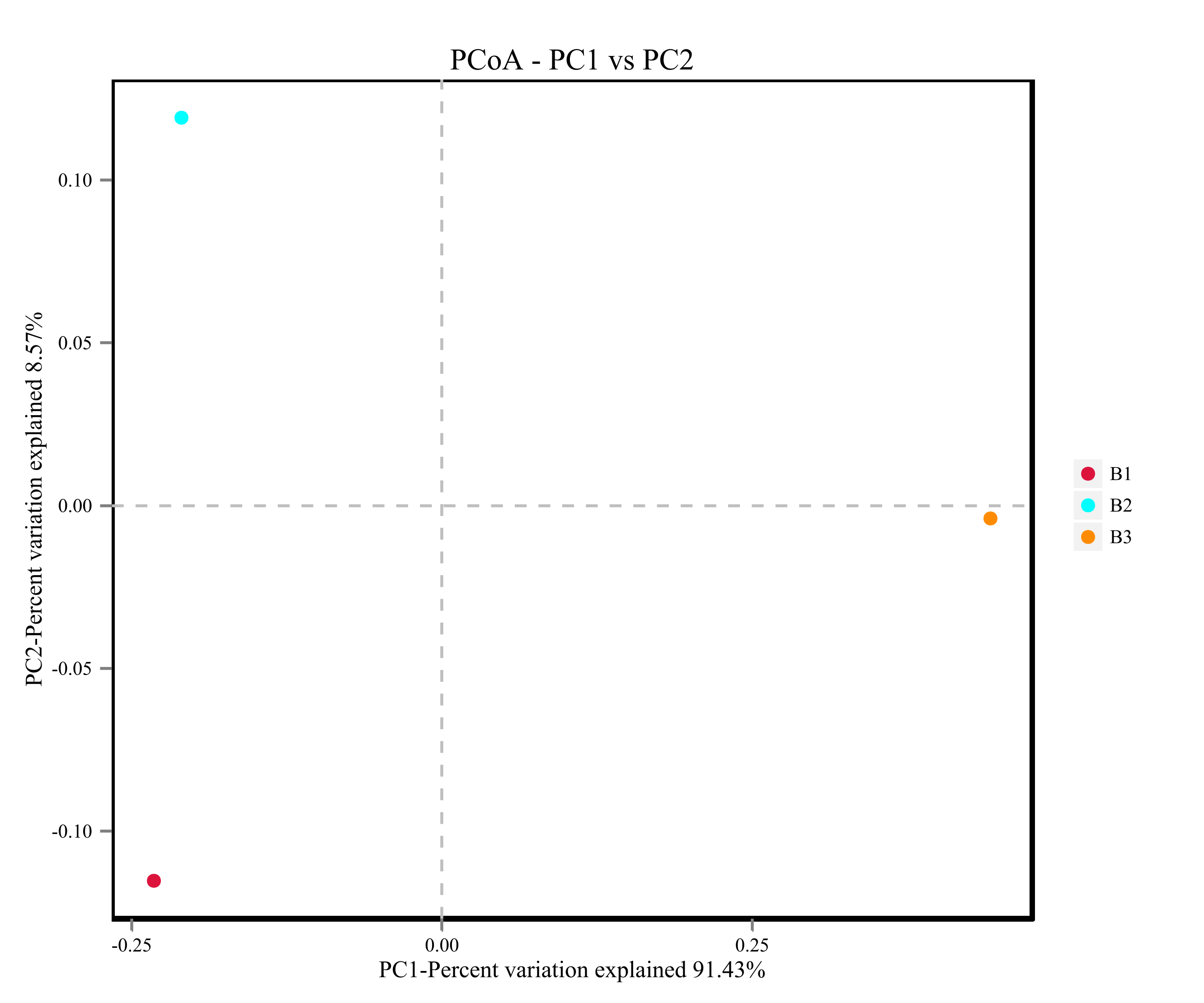

Supplement: S2 Data — (ZIP) [file pone.0261306.s002.zip › customer_backup/beta_diversity/pcoa/treat/treat.bray_curtis.PC1_PC2.png]

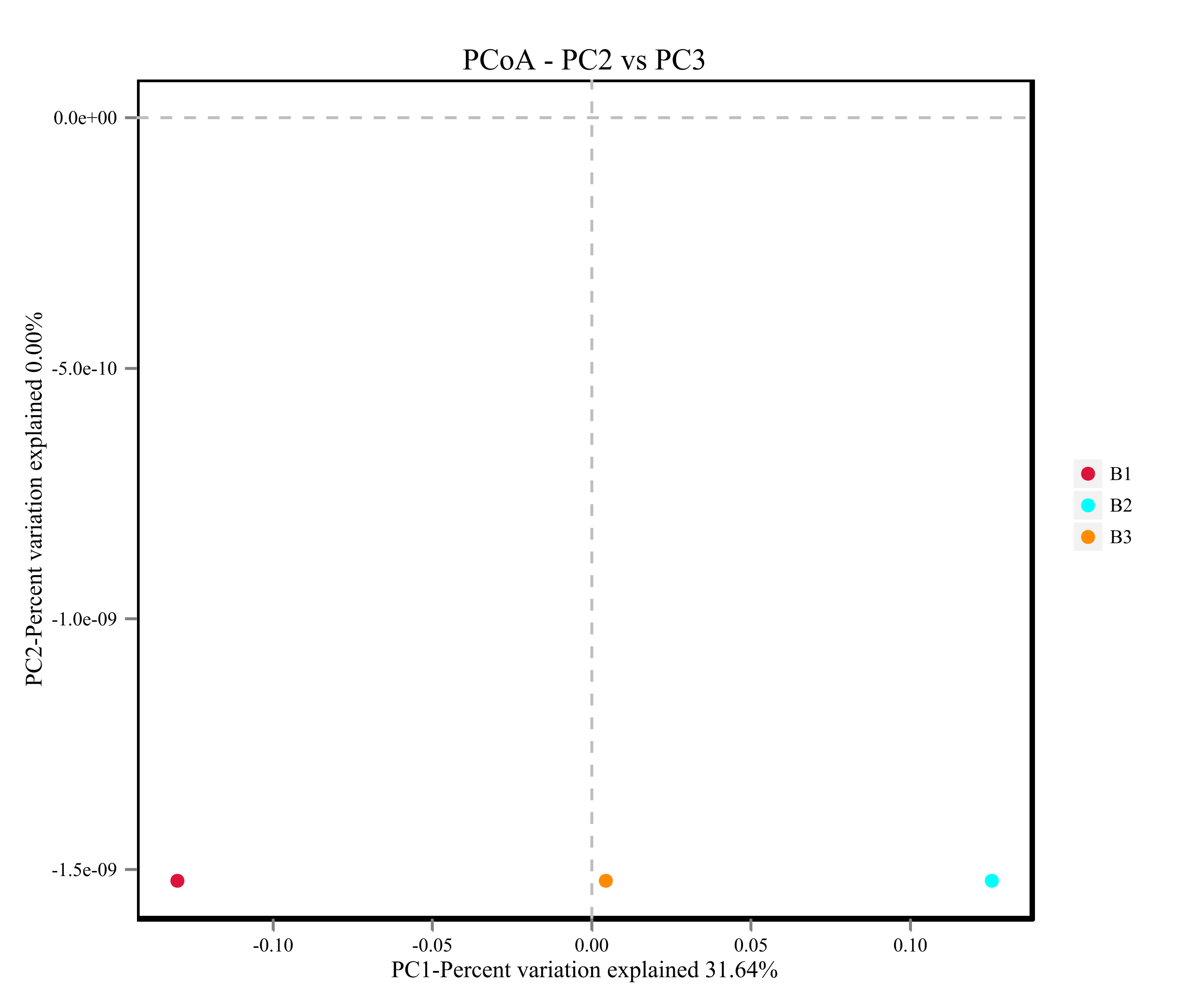

Supplement: S2 Data — (ZIP) [file pone.0261306.s002.zip › customer_backup/beta_diversity/pcoa/treat/treat.binary_jaccard.PC2_PC3.png]

Multy samples Rarefaction Curves

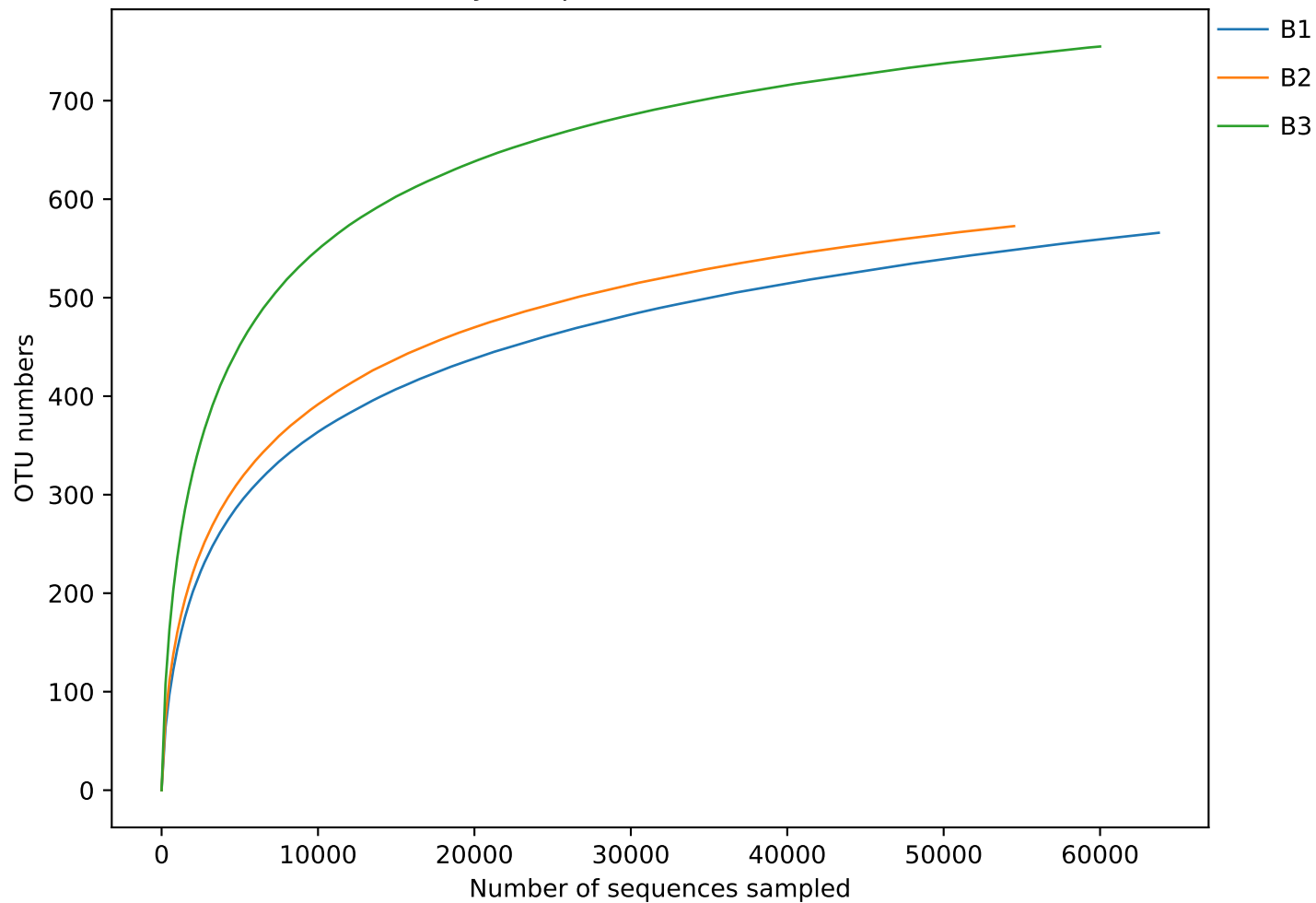

Supplement: S2 Data — (ZIP) [file pone.0261306.s002.zip › customer_backup/alpha_diversity/rarefaction_curve/treat/treat.Rarefaction.curve_group.pdf]

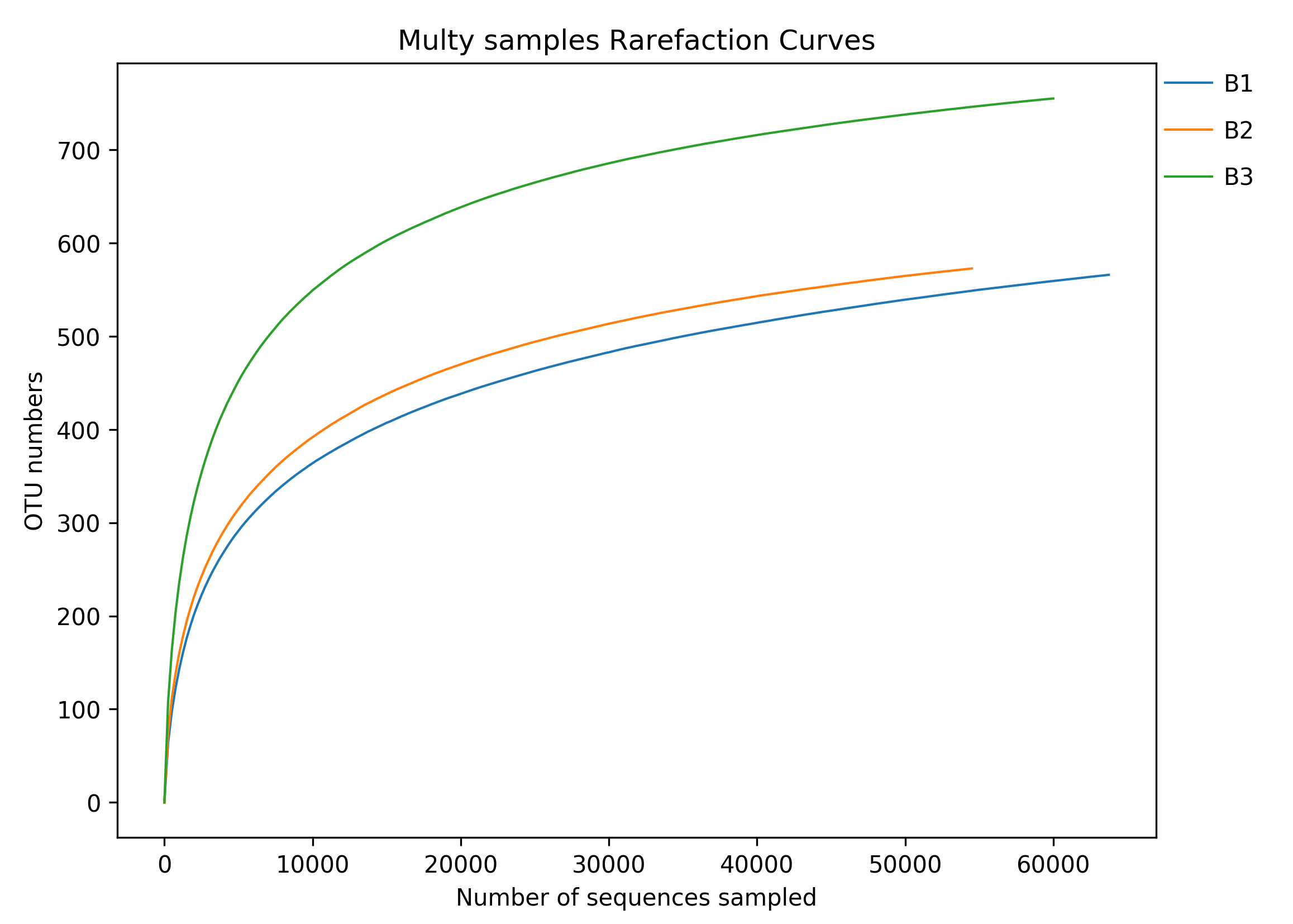

Supplement: S2 Data — (ZIP) [file pone.0261306.s002.zip › customer_backup/alpha_diversity/rarefaction_curve/treat/treat.Rarefaction.curve_group.png]

Multy samples Rarefaction Curves

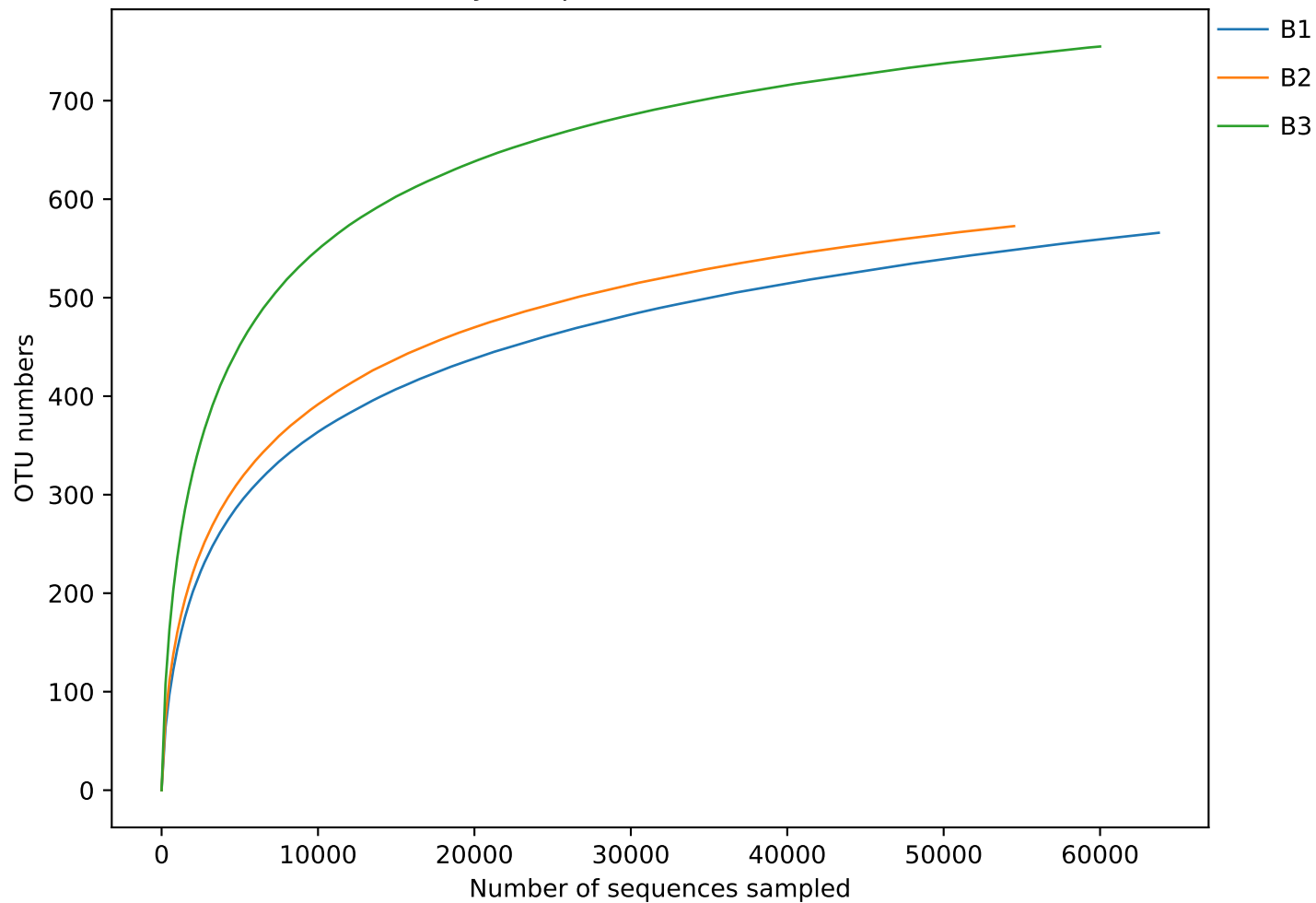

Supplement: S2 Data — (ZIP) [file pone.0261306.s002.zip › customer_backup/alpha_diversity/rarefaction_curve/allsample/allsample.Rarefaction.curve_group.pdf]

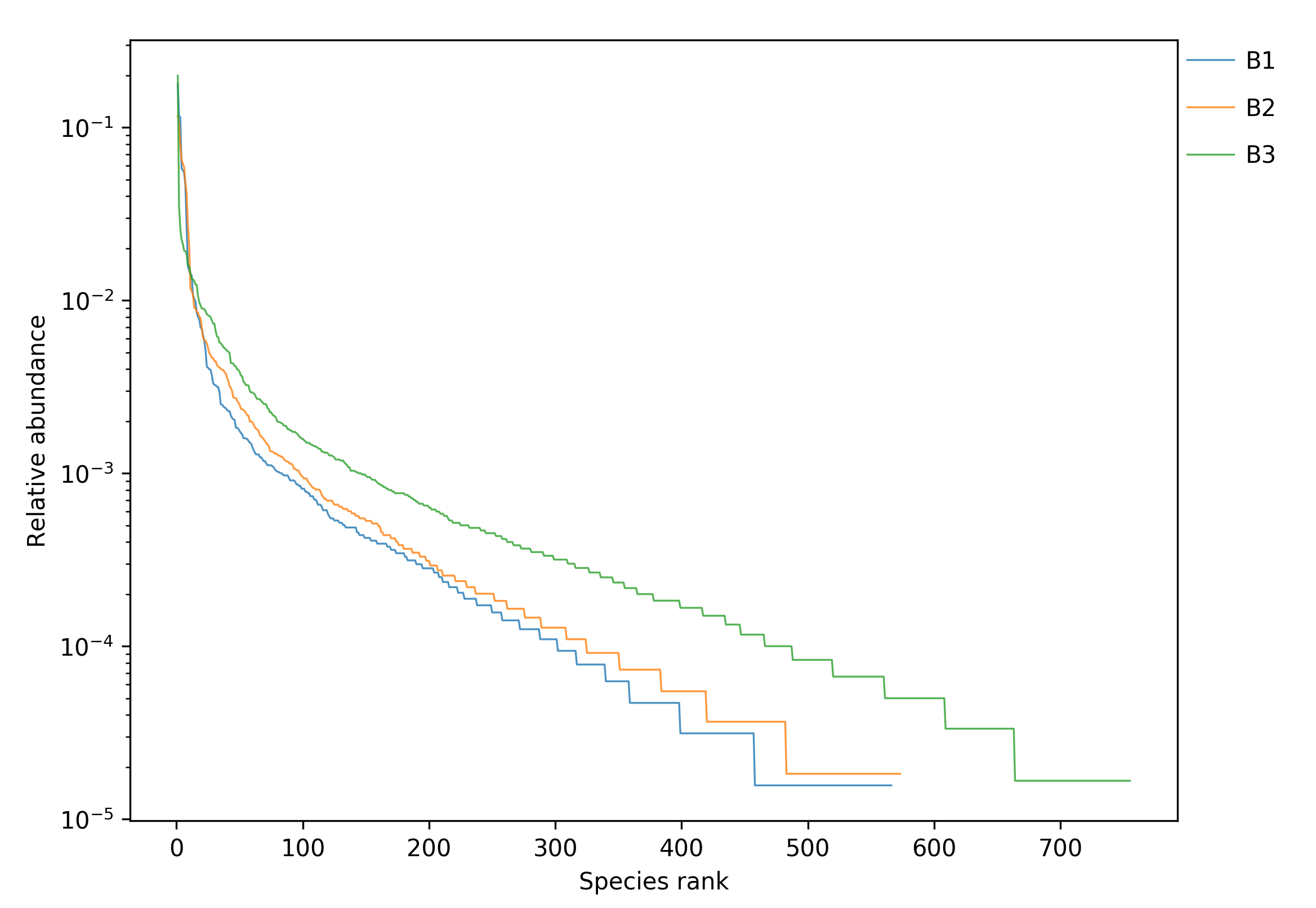

Supplement: S2 Data — (ZIP) [file pone.0261306.s002.zip › customer_backup/alpha_diversity/rank_abund_curve/treat/treat.rank.abund.curve.png]

Relative abundance

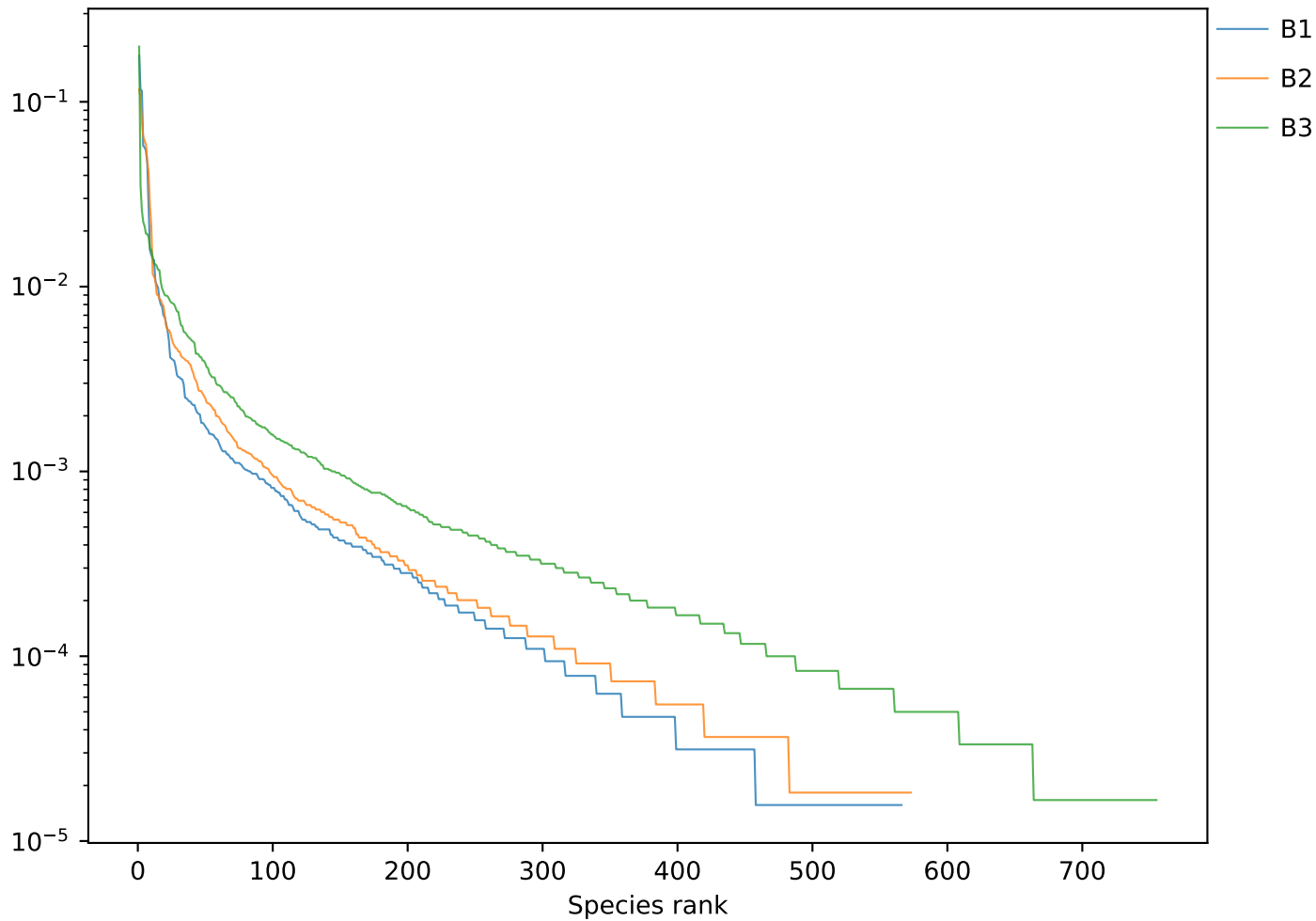

Supplement: S2 Data — (ZIP) [file pone.0261306.s002.zip › customer_backup/alpha_diversity/rank_abund_curve/treat/treat.rank.abund.curve.pdf]

Relative abundance

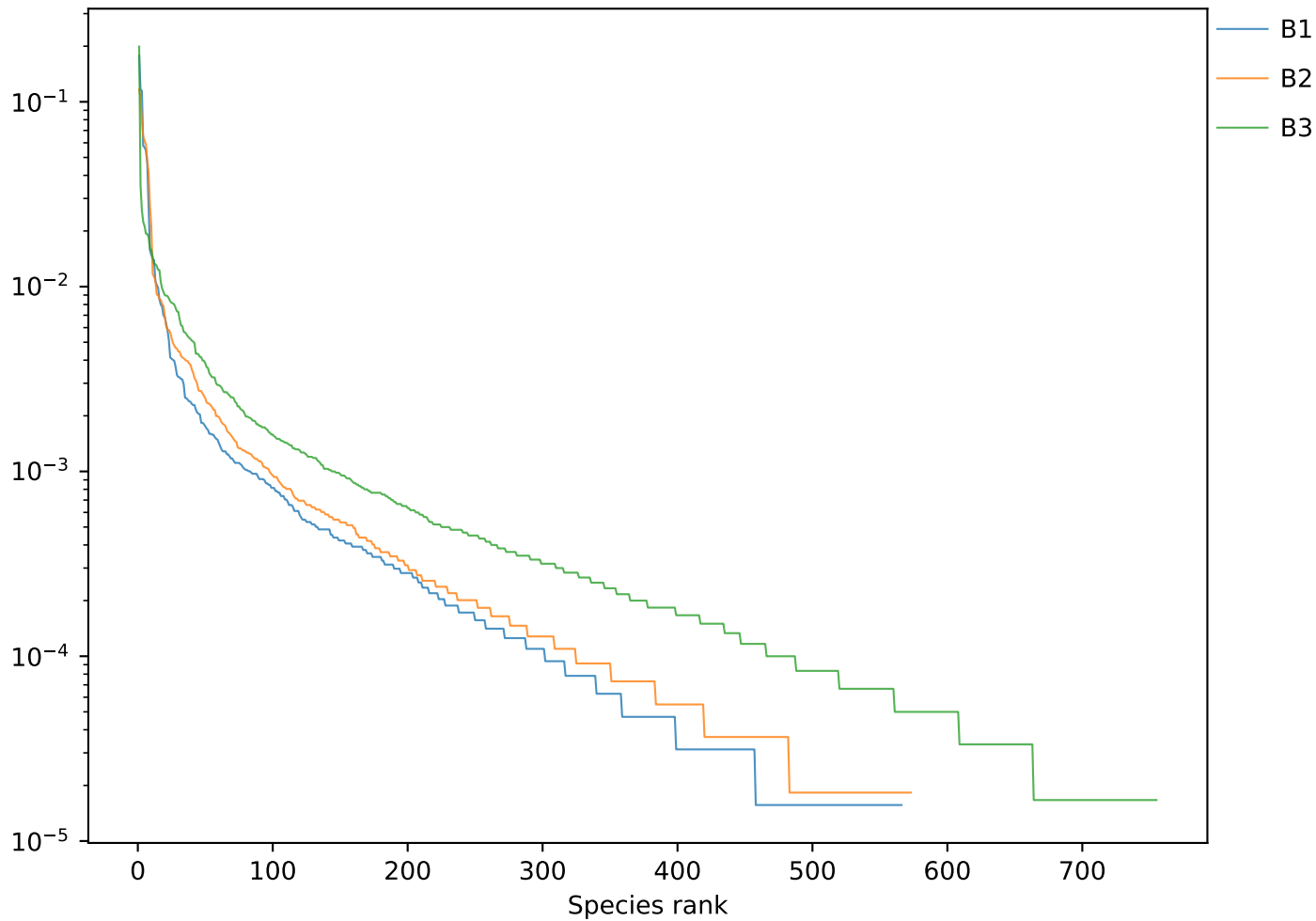

Supplement: S2 Data — (ZIP) [file pone.0261306.s002.zip › customer_backup/alpha_diversity/rank_abund_curve/allsample/allsample.rank.abund.curve.pdf]

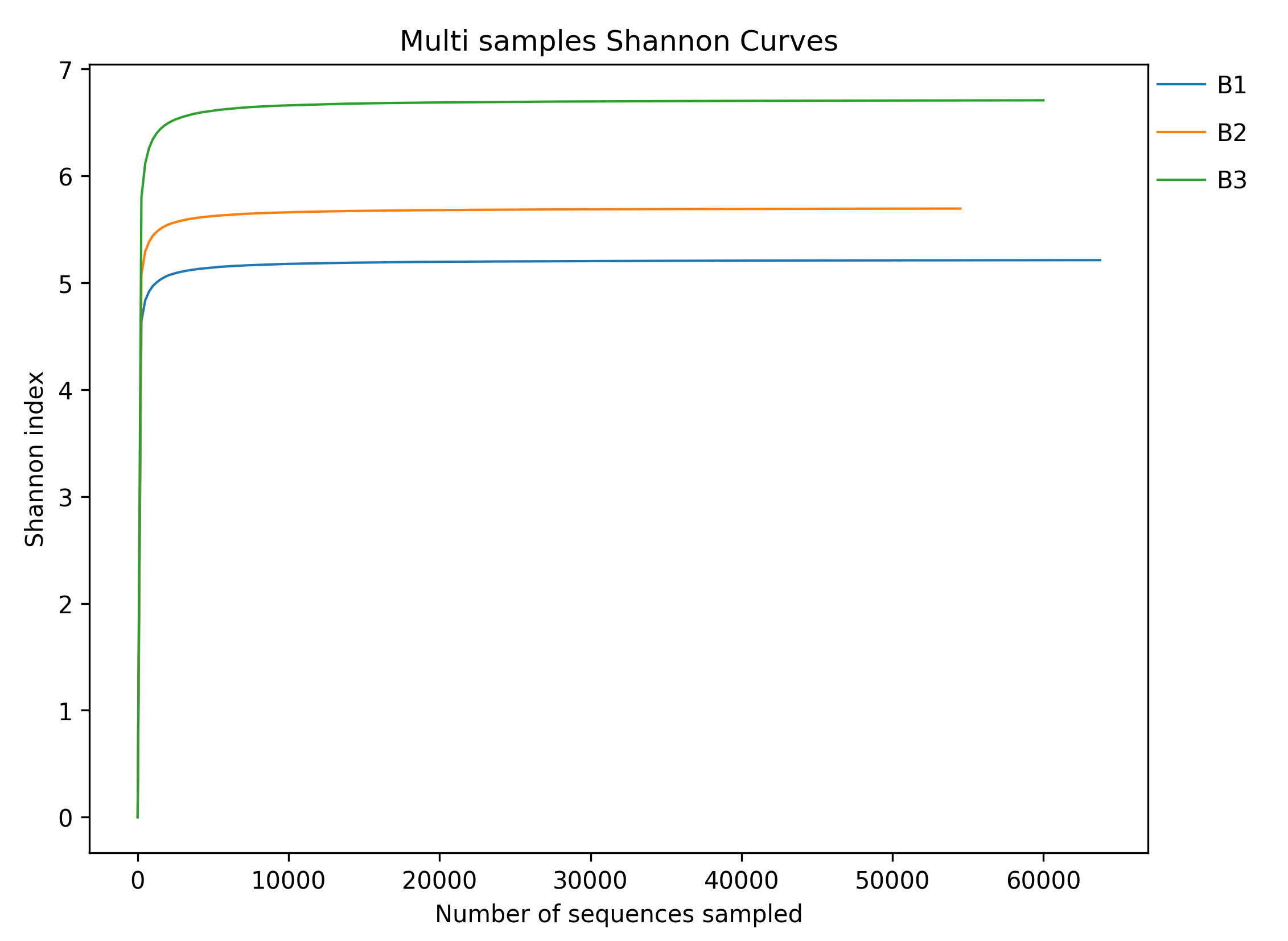

Supplement: S2 Data — (ZIP) [file pone.0261306.s002.zip › customer_backup/alpha_diversity/shannon_index_curve/treat/treat.shannon.curve_group.png]

Multi samples Shannon Curves

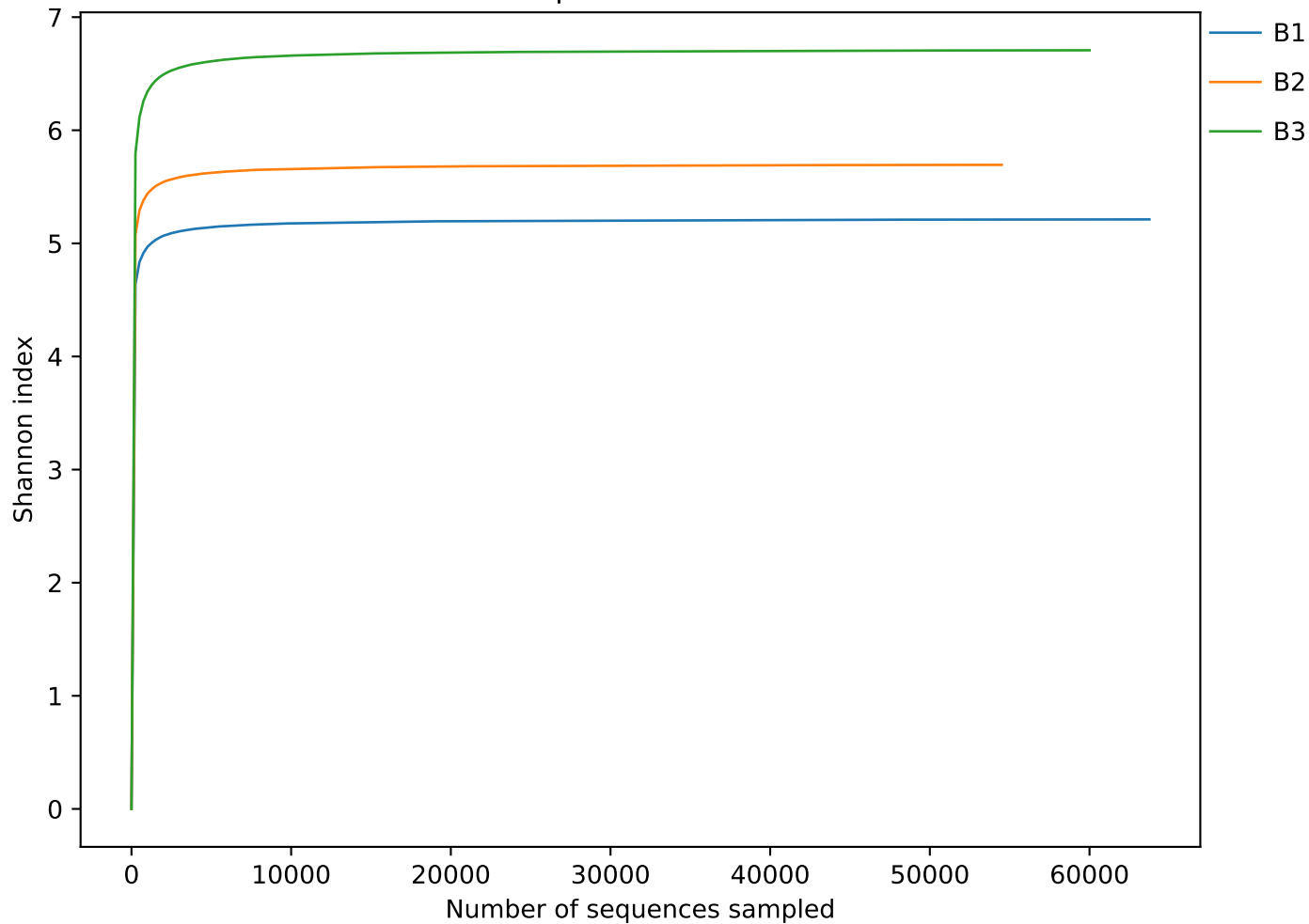

Supplement: S2 Data — (ZIP) [file pone.0261306.s002.zip › customer_backup/alpha_diversity/shannon_index_curve/treat/treat.shannon.curve_group.pdf]

Multi samples Shannon Curves

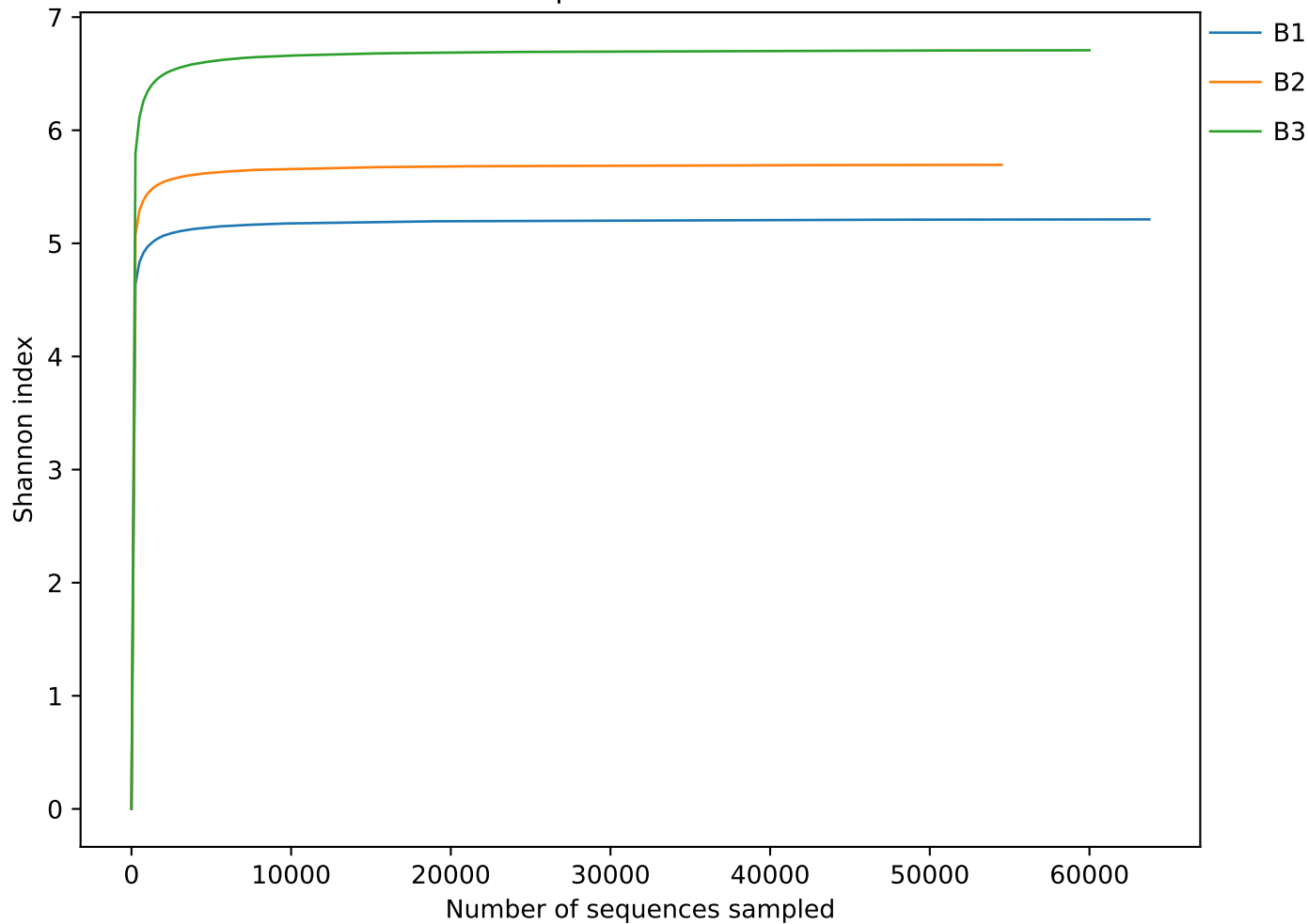

Supplement: S2 Data — (ZIP) [file pone.0261306.s002.zip › customer_backup/alpha_diversity/shannon_index_curve/allsample/allsample.shannon.curve_group.pdf]
